# Differential early diagnosis of benign versus malignant lung cancer using systematic pathway flux analysis of peripheral blood leukocytes

**DOI:** 10.1038/s41598-022-08890-x

**Published:** 2022-03-24

**Authors:** Jian Li, Xiaoyu Li, Ming Li, Hong Qiu, Christian Saad, Bo Zhao, Fan Li, Xiaowei Wu, Dong Kuang, Fengjuan Tang, Yaobing Chen, Hongge Shu, Jing Zhang, Qiuxia Wang, He Huang, Shankang Qi, Changkun Ye, Amy Bryant, Xianglin Yuan, Christian Kurts, Guangyuan Hu, Weiting Cheng, Qi Mei

**Affiliations:** 1 Institute of Molecular Medicine and Experimental Immunology, University Clinic of Rheinische Friedrich-Wilhelms-University, Bonn, Germany; 2 Department of Oncology, Tongji Hospital, Tongji Medical College, Huazhong University of Science and Technology, Wuhan, Hubei People’s Republic of China; 3 Department of Oncology, Wuhan Pulmonary Hospital, Wuhan, Hubei People’s Republic of China; 4 Department of Computer Science, University of Augsburg, Augsburg, Germany; 5 Department of Thoracic Surgery, Tongji Hospital, Tongji Medical College, Huazhong University of Science and Technology, Wuhan, Hubei People’s Republic of China; 6 Institute of Pathology, Tongji Hospital, Tongji Medical College, Huazhong University of Science and Technology, Wuhan, Hubei People’s Republic of China; 7 Department of Pathology, School of Basic Medicine, Tongji Medical College, Huazhong University of Science and Technology, Wuhan, Hubei People’s Republic of China; 8 Radiology Department, Tongji Hospital, Tongji Medical College, Huazhong University of Science and Technology, Wuhan, Hubei People’s Republic of China; 9 Shanghai Institute of Materia Medica, Chinese Academy of Sciences, Shanghai, People’s Republic of China; 10 Medical Research Center of Yu Huang Hospital, Yu Huang, Zhejiang People’s Republic of China; 11 Department of Biochemical and Pharmaceutical Sciences, College of Pharmacy, Idaho State University, Pocatello, USA; 12 Department of Oncology, Wuhan No. 1 Hospital, Wuhan, Hubei People’s Republic of China

**Keywords:** Immune evasion, Diagnostic markers, Computational science

## Abstract

Early diagnosis of lung cancer is critically important to reduce disease severity and improve overall survival. Newer, minimally invasive biopsy procedures often fail to provide adequate specimens for accurate tumor subtyping or staging which is necessary to inform appropriate use of molecular targeted therapies and immune checkpoint inhibitors. Thus newer approaches to diagnosis and staging in early lung cancer are needed. This exploratory pilot study obtained peripheral blood samples from 139 individuals with clinically evident pulmonary nodules (benign and malignant), as well as ten healthy persons. They were divided into three cohorts: original cohort (n = 99), control cohort (n = 10), and validation cohort (n = 40). Average RNAseq sequencing of leukocytes in these samples were conducted. Subsequently, data was integrated into artificial intelligence (AI)-based computational approach with system-wide gene expression technology to develop a rapid, effective, non-invasive immune index for early diagnosis of lung cancer. An immune-related index system, IM-Index, was defined and validated for the diagnostic application. IM-Index was applied to assess the malignancies of pulmonary nodules of 109 participants (original + control cohorts) with high accuracy (AUC: *0.822* [95% CI: 0.75–0.91, *p* < 0.001]), and to differentiate between phases of cancer immunoediting concept (odds ratio: 1.17 [95% CI: 1.1–1.25, *p* < 0.001]). The predictive ability of IM-Index was validated in a validation cohort with a AUC: 0.883 (95% CI: 0.73–1.00, *p* < 0.001). The difference between molecular mechanisms of adenocarcinoma and squamous carcinoma histology was also determined via the IM-Index (OR: 1.2 [95% CI 1.14–1.35, *p* = 0.019]). In addition, a structural metabolic behavior pattern and signaling property in host immunity were found (bonferroni correction, *p* = 1.32e − 16). Taken together our findings indicate that this AI-based approach may be used for “Super Early” cancer diagnosis and amend the current immunotherpay for lung cancer.

## Introduction

Lung cancer is associated with high mortality worldwidely^1,2^. Although newer diagnosis and treatment modalities for lung cancer have been substantially improved over the past 5 years, survival rates remain extremely low (5%)^3^. Early diagnosis is of particular importance since it can identify patients who would benefit from these newer therapeutic strategies and thus improve survival. Currently, definitive diagnosis and tumor staging rely on surgical biopsy. However, these invasive procedures often fail to provide adequate tissue specimens for tumor subtyping/characterization and patients with benign lesions are put at unnecessary risk. Thus, a non-invasive method that can differentiate between benign and malignant lung cancer at the earliest stages of disease would be of great clinical benefit.

Accumulating evidence has suggested that the total lymphocyte count, neutrophil–lymphocyte ratio, and platelet-lymphocyte ratio can be used to predict treatment outcomes of diverse therapies such as chemotherapy^4^, targeted therapy^5^, surgery^6^ and immunotherapy^7,8^. Other evidence clearly indicates that peripheral blood leukocytes play an essential role in diverse malignant transformation processes such as tumor angiogenesis^9^, proliferation^10^, metastasis^11^ and treatment resistant^12^. These findings suggest that peripheral blood leukocytes, being critical components of the immune system, may contain valuable and stable information for diverse clinical applications including early lung cancer diagnosis.

A recent paradigm for cancer development is the cancer immunoediting concept (CIC). This concept is an extension of the “immune surveillance” hypothesis^13^ and describes the host immune system’s essential, but contradicting, roles in tumor growth and metastasis^14^. It embodies the fundamental struggle between host and tumor, and consists of three interdependent phases of cancer progression: elimination, equilibrium, escape^15^. The CIC has been unequivocally established as a prominent paradigm in tumor immunology^16^, yet there are three remaining questions: i) how to differentiate between these three phases in real time, ii) how to assess the overall functional state of the host immune system, and iii) whether tumor cells can modulate these phases to alter host immunity and effect its own survival and progression.

Any activation of the immune system is a energy-demanding process and is accompanied by a dramatic metabolic remodeling response^17^. The levels of metabolic nutrients including glucose, fatty acids and amino acids are strongly correlated to the functional status of immune cells^18–20^. Moreover, activation of the immune system is also an intensive signaling process, in which diverse signaling pathways function to sense antigen^21^, to invoke development and differentiation of immune-cells^22–24^ and to conduct immune functions and tolerance^25,26^. Therefore, metabolism and signaling “collaborate” in an inseparable manner to ensure appropriate and efficient functionality of the immune system^27^. Fluxes in intracellular metabolic networks reflect the magnitude and direction of cellular responses to external stimuli or conditions. Though powerful, metabolic flux predictions can be improved by incorporating absolute gene expression data. Indeed, many studies have shown that transcriptomic data can be applied to calculate the pathway flux to describe quantitatively the intensity of metabolism and signaling^28–30^. We hypothesize that metabolic pathway flux analysis applied over a genome-scale signaling pathway network during cancer development might provide more accurate insight into the functional status of the immune system.

Recently developed machine-learning based approaches have been applied to pathological images, including computed tomography and immunostained tissue sections, in an effort to improve cancer diagnosis^31–33^. However, this approach demonstrated only limited ability to differentiate between benign and malignant lesions, likely because of a relative scarcity of machine training data for benign lesions. Other studies tried to identify molecular signatures at the DNA, RNA and epigenetic levels by comparing tumor and normal tissues. However, the resultant molecular signatures varied significantly due to the heterogeneity of the tumor tissue and the applied statistical approaches^34–36^.

In the current prospective study, we utilized immune-related information to define an index system (IM-Index) in an AI model and subsequently applied the IM-Index to analyze the RNAseq of peripheral blood leukocytes of 99 participants with unknown pulmonary nodules and 10 healthy participants. Using this approach, our results show that RNAseq data from peripheral leukocytes distinguishes between benign versus malignant pulmonary nodules with high accuracy. Further, our technique could differentiate between adenocarcinoma and squamous cell carcinoma in the malignant nodules. This non-invasive, AI-based approach creates a unique opportunity to significantly improve early cancer diagnosis and staging of disease.

## Patients and methods

### Study objective and design

The aim of this study was to develop a highly accurate, non-invasive method for early cancer diagnosis that exploited immune information contained in peripheral blood leukocytes. Peripheral blood samples (3–5 mLs) were obtained through evacuated blood collection tubes (BD, New Jersey, USA) from 99 individuals with clinically evident pulmonary nodules (benign and malignant), as well as 10 healthy persons (Table 1). Additionally, a validation cohort consisting of 40 patients were recruited (Table 1 & Supplement Table 1). Participants were from Wuhan Tongji Hospital, Wuhan Pulmonary Hospital and Wuhan No.1 Hospital (PR China) in 2019. Due to the exploratory nature of this study no prospective sample size calculation was done. The number of participants to be studied was estimated from past experience. A clinical and histological diagnosis was available for all individuals. The inclusion criteria included: i) age between 18 and 80 years, ii) diagnosis of pulmonary nodule requiring surgery and subsequent histopathological analysis, iii) availability of blood specimen for sequencing, iv) willing to participate in this project. Patients were excluded if they: i) were pregnant or lactating; ii) had received treatments before diagnosis; iii) had a prior medical history of cancer or other related malignancy including chronic inflammation-related lung diseases, tuberculosis, and autoimmune disease; or vi) were not willing to participate in this study. Blood samples were not studied if: i) resultant plasma specimens were contaminated; or ii) blood specimens were not prepared for analysis within 24 h. According to the World Health Organization (WHO), a current smoker is someone who smokes daily at least one cigarette.

**Table 1:** Clinical characteristics and laboratory findings.

	Malignant	Benign	Validation	*p*-value
Number of patients	78	21	40	…
Age, years	59.0 [53.2–64.8]	53.0 [43.0–64.0]	55.6 [47.3–62.7]	0.040
**Sex**	…	…	…	0.524
Male	45 (57.7)	12 (57.1)	27 (67.5)	…
Female	33 (42.3)	9 (42.9)	13 (32.5)	…
**Clinical stages**	…	…	…	0.419
I	52 (66.7)	…	23 (57.5)	…
II	10 (12.8)	…	4 (10.0)	…
III	13 (16.7)	…	3 (7.5)	…
IV	3 (3.8)	…	0 (0.0)	…
**T stage**	…	…	…	0.325
1	36 (46.2)	…	14 (35.0)	…
2	33 (42.3)	…	13 (32.5)	…
3	5 (6.4)	…	2 (5.0)	…
4	4 (5.1)	…	1 (2.5)	…
**N stage**	…	…	…	0.076
0	62 (79.5)	…	25 (52.5)	…
1	3 (3.8)	…	2 (5.0)	…
2	12 (15.4)	…	2 (5.0)	…
3	1 (1.3)	…	1 (2.5)	…
**M stage**	…	…	…	< .001
0	75 (96.2)	…	28 (70.0)	…
1	3 (3.8)	…	2 (5.0)	…
**Pathological type**	…	…	…	0.110
Adenocarcinoma	52 (66.7)	…	20 (50.0)	…
Squamous carcinoma	19 (24.4)	…	7 (17.5)	…
Other carcinoma	7 (9.0)	…	3 (7.5)	…
**Invasive depth**	…	…	…	0.003
0	1 (1.3)	…	2 (5.0)	…
1	8 (10.3)	…	2 (5.0)	…
2	69 (88.5)	…	26 (65.0)	…
**Differentiation grade**	…	…	…	0.020
1	6/63 (9.5)	…	4 (10.0)	…
2	52/63 (82.5)	…	24 (60.0)	…
3	5/63 (7.9)	…	2 (5.0)	…
**No. of pulmonary nodules**	…	…	…	0.699
1	63 (80.8)	19 (90.5)	35 (62.5)	…
2	13 (16.7)	2 (9.5)	4 (10.0)	…
3	2 (2.6)	0	1 (2.5)	…
**TIL grade**	…	…	…	0.288
0	52/72 (72.2)	0	22 (55.0)	…
1	19/72 (26.4)	1/1 (100)	7 (17.5)	…
2	1/72 (1.4)	0	1 (2.5)	…
TII	10.0 [6.0–15.0]	10.0 [10.0–10.0]	10.0 [6.0–15.0]	0.849
CD8	1.0 [1.0–1.5]	1.0 [1.0–1.0]	1.0 [1.0–1.0]	0.571
PD-L1	3.0 [3.0–8.0]	1.0 [1.0–1.0]	3.0 [2.5–4.25]	< .001
CPS	4.0 [3.0–11.0]	1.0 [1.0–1.0]	4.0 [1.75–11.0]	< .001
**TIMIT**	…	…	…	1.000
I	27 (37.5)	1 (4.7)	11 (27.5)	…
II	21 (29.2)	0	8 (20.0)	…
III	7 (9.7)	0	3 (7.5)	…
IV	17 (23.6)	0	8 (20.0)	…
Smoking	33 (42.3)	7 (33.3)	17 (42.5)	0.615
**Macrophage**	…	…	…	0.877
< 5	57 (79.2)	1(4.7)	24 (60.0)	…
5–10	13 (18.1)	0	4 (10.0)	…
> 10	2 (2.8)	0	2 ( 5.0)	…
Ki67	0.3 [0.1–0.6]	…	0.3 [0.1–0.6]	…
**Tumor diameter, cm**	…	…	…	…
Imaging	2.4 [1.6–3.6]	2.0 [1.6–3.8]	2.5 [1.2–3.2]	0.562
Surgical	3.0 [1.9–4.0]	2.0 [1.5–3.5]	2.5 [1.5–3.8]	0.160
Pathological	2.5 [1.5–4.0]	2.0 [1.0–3.0]	2.2 [1.5–3.8]	0.138
CEA, ng/mL	41.0 [18.3–63.8]	21.0 [10.0–53.0]	34.0 [10.8–55.3]	0.092
NSE, ug/L	44.5 [17.3–66.8]	37.0 [23.0–52.0]	42.0 [15.3–70.3]	0.604
CYFRA19, ug/L	39.0 [15.3–59.8]	21.0 [12.0–45.0]	32.0 [11.8–53.0]	0.137
SCC , ng/mL	10.0 [7.0–14.8]	8.0 [5.0–12.0]	8.0 [6.0–10.3]	0.044
ProGRP, pg/mL	34.5 [11.5–54.0]	33.0 [18.0–56.0]	32.5 [10.3–58.5]	0.775
WBC count, × 10^9^/L	5.6 [4.6–7.4]	5.7 [4.5–6.5]	5.9 [4.6–7.6]	0.745
Neutrophil count, × 10^9^/L	3.4 [2.5–4.7]	3.2 [2.8–4.0]	3.9 [2.7–5.1]	0.619
Lymphocyte count, × 10^9^/L	1.6 [1.3–1.9]	1.6 [1.4–1.9]	1.5 [1.3–1.8]	0.451
Hemoglobin, g/L	133.0 [124.0–141.0]	140.0 [119.0–152.0]	138.0 [124.0–151.0]	0.220
PLT count, × 10^9^/L	232.0 [181.0–260.0]	227.0 [205.0–268.0]	213.0 [172.0–254.5]	0.863

*CEA* carcinoembryonic antigen; *CPS* carbamoyl-phosphate synthase 1; *CYFRA19* cytokeratin fragment 19; *NSE* neutron-specific enolase;*PLT* platelet count; *ProGRP* progastrin-releasing peptide; *SCC* squamous cell carcinoma; *TIL* tumor infiltrating leukocyte; *WBC* white blood cell.

**Figure 1:**
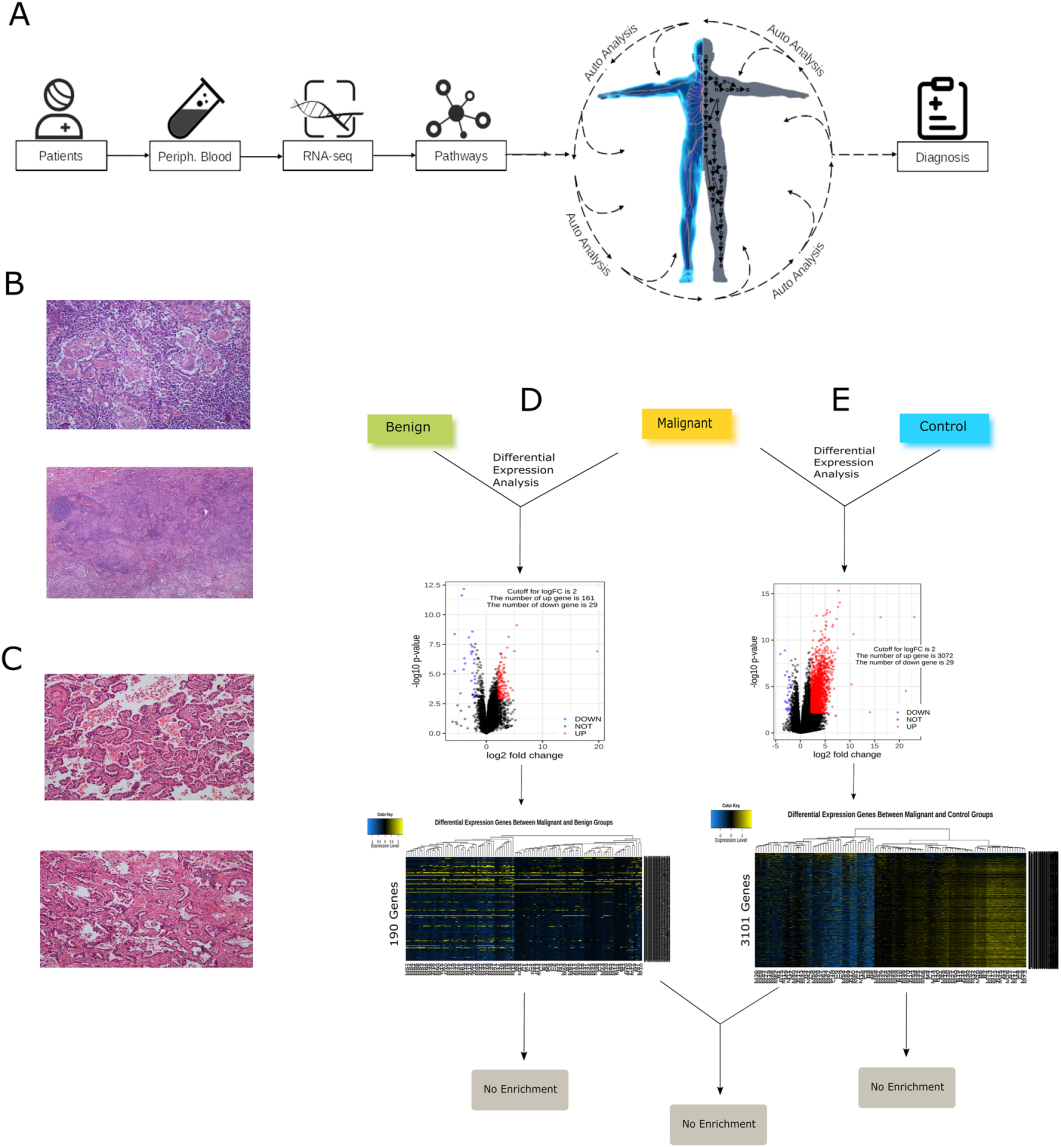
Workflow and analysis of differential expression genes between three groups. (**A**) The schematic representation of the workflow for peripheral blood-based IM-Index calculation. Each blood sample was sequenced to generate RNAseq data, which was integrated into the AI model. Subsequently, the AutoAnalysis was performed to calculated the pathway flux in the AI model, the IM.Index was calculated and diagnosis was defined correspondingly. (**B**) Haematoxylin and eosin (H&E) stained tissue sample of a malignant participant showing invasive carcinoma at the margin (top) and widespread malignancy (down). (**C**) H&E stained tissue sample from a benign participant with dysplastic epithelia at the margin (top) and widespread low aggressive tissue development (down). (**D**) Analysis of differential expressed genes between malignant and benign groups, the result showed that 190 genes were found to be differential expressed, however, the gene ontology (GO) enrichment analysis in this gene set did not reach a positive result. (**E**) The same analysis was conducted between malignant and control groups, the results showed that 3101 genes were differentially expressed between both groups, however, the GO enrichment analysis showed a negative result either.

Written informed consent was obtained from all participants. This study was approved by the Chinese Ethics Committee of Registering Clinical Trials (No.: ChiECRCT20190174) and all methods were performed in accordance to clinical guidance and regulations. Patient demographic and clinical data including age, gender, TNM-stage, diagnosis, tumor grading, nodule size and others were additionally collected. Tumor biopsies from lung cancer patients enrolled in a previous study^37^ were used to generate tumor-specific molecular data. All data analyses were performed in the Tongji Hospital, Wuhan, China. Clinical and sequencing data remain in place and are secured within the hospital data information system. The data analysis team was blinded with respect to the study endpoint (malignancy). Average RNAseq sequencing of leukocytes in these samples was conducted and integrated into an AI model in order to perform a pathway flux analysis at a system level. Based on the results, a host-based immune-related index (IM-Index) was developed and tested for its ability to predict the malignancy of pulmonary nodules. Predictive accuracy of the IM-Index-based method for diagnosis of malignancy were assessed in relation to standard histopathological confirmation of malignancy from tumor biopsies. Figure 1A visualizes the workflow of this study.

### Peripheral leukocyte isolation and extraction

Each blood sample was centrifuged at 1600 × g for 10 min, take 400 μL of the middle layer (leucocyte layer), added it to a 2 mL Eppendorf (EP) tube, added 1.2 mL of red blood cell lysate, from vortex to central mixed, placed at room temperature for 5–10 min, and centrifuged at 4 °C for 1 min at 10000 g; Used a pipette to aspirate the upper layer of solution, discarded, and added 1 mL trizol reagent (Vazyme Biotech, Nanjing, China) and mixed well; Added 200 μL of chloroform, shaked and mixed, and place at 4 °C for 10 min; Centrifuged at 12000 rpm for 10 min at 4 °C; Took the supernatant, added the same volume of isopropanol as the supernatant, and let stand on ice for 25 min; Centrifuged at 12,000 rpm for 10 min at 4 °C, discarded the supernatant; Added 1 mL 70% ethanol (75%) and centrifuged at 12000 rpm for 10 min at 4 °C, and repeated this step once more; Centrifuged at 4 °C 12,000 rpm for 2 min, left it for 2-5 min to air dry; After adding 30 μL of DEPC water, Nanodrop (Thermo Scientific, Waltham, USA) was used to measure the RNA concentration.

### RNAseq data analysis

The RNA concentration was measured by Nanodrop (Thermo Scientific, Waltham, USA) and RNA quality was determined by Qubit RNA IQ assay. Only RNA samples with RNA Integrity Number (RIN) > 7 were studied. An RNAseq analysis of each qualified sample was performed and an RNA library was prepared and sequenced (paired-end 150-bp reads) using VAHTS mRNA-seq v3 Library Prep Kit for Illumina (Vazyme Biotech, Nanjing, China) and daDNA HS Assay Kit (YEASEN Biotech, Shanghai, China). In brief, the mRNA was isolated and fragmented from total RNA, then the synthesis and purification of double-stranded cDNA was implemented. After terminal repair and junction ligation, the ligation products were purified, the fragment size was sorted, and the library was amplified. Lastly, the sequencing results of the mRNA were analyzed. Based on these results, sequence reads were aligned using Salmon (v0.9.1), and the transcript quantification was performed by aggregating the transcript counts. Salmon uses the raw fastq sequence reads and the reference transcriptome assembly (GRCh37). A quasi-mapping method is then applied where the program tool computes the mapping of reads to the transcript positions without performing a base-to-base alignment of reads to the transcript.

### Pathological diagnosis

Surgically resected lung cancer tumor specimens were fixed in 10% neutral-buffered formalin (NBF) and embedded in paraffin. All embedded blocks were cut into 3 μm in thickness, H&E-stained, and imaged using a Nikon 80i optical microscopy. The expression of CD8 and PD-L1 in lung cancer tissue was measured by EnVision immunohistochemistry (IHC) using EnVision™ system. Rabbit anti-human CD8 monoclonal antibody (Clone SP16, cell membrane-located) was purchased from ZsBio (Beijing, China). Mouse anti-Human PD-L1 monoclonal antibody (Clone 22C3, cell membrane-located in cancer cells and cell membrane- or cytoplasm-located in immune cells) was obtained from DAKO (Santa Clara, CA, USA). Commercially available normal lung tissues and PBS buffer were used as negative and blank controls, respectively.

### The construction of artificial intelligent (AI) MODEL

The construction of AI model utilized the biological knowledge derived from literature references such as PubMed and publicly available databases such as Reactome (https://reactome.org/). The model possesses four layers with following relationship: (1) gene → (2) RNA → (3) protein/complex/compound → (4) pathway → (1) gene (Supplement Fig. 1A) with last layer (115 pathway) being connected back to the first layer (3643 gene) due to diverse feedback mechanisms. A summary of the statistical component of the AI model is provided in Supplement Table 1. The signaling and metabolic components of the AI model were derived from our previously published molecular signaling map (MSM)^38^ and metabolic network (MCPM)^13^, respectively. Each component in the AI model is associated with a unique corresponding ID. For instance, gene is linked with Ensemble-ID, protein with UniProt ID, compound with ChEBI-ID and so on. The unique-ID system is organized with the modeling station of SimCell. The transcriptional- and translational-regulations and feedback loops have also been considered in AI model. The transcriptional regulation information derives from the TRANSFAC database (http://genexplain.com/transfac). The miRNA regulation network has been integrated based on the information from miRNA database miRBase (http://www.mirbase.org/) and miRNA target database mirWalk (http://zmf.umm.uni-heidelberg.de/apps/zmf/mirwalk2/). The miRNA pattern were defined two aspects: miRNA gene transcription and miRNA target binding (Supplement Fig. 1B). The kinetic parameters for the AI model were derived from different kinetic databases including BRENDA (https://www.brenda-enzymes.org), SABIO-RK (http://sabio.h-its.org/), and NIST (https://kinetics.nist.gov/kinetics/index.jsp). This layer-structure is translated into a two-array digital structure for pathway flux calculation (Eq. (1) and (2), described in the following section) (Supplement Fig. 1C).

### Genome-scale pathway flux analysis (GPFA)

The GPFA is developed to detect whether a therapeutic intervention can invoke a genome-scale significant change of data flux within a cellular system. The data flow (flux) was generated from AutoAnalyze^14^ based on related readout components in the AI model. For instance, a molecular reaction R with role ∈ {e, g, i, s, tr(a) , tr(r) }, R symbolizes a reaction set; (e: enzyme; g: gene; i: inhibitor; s: substrate; tr(a): transcriptional activator , tr(r): transcriptional repressor):

1 $${\text{I}}_{{\text{j}}} \left( {{\text{reaction}},{\text{role}}} \right) \, = \, \prod\limits_{{{\text{c}}({\text{object}})}} {{\text{objects}}\; \in \;{\text{reactant}}\;\left( {{\text{reaction}},{\text{role}}} \right).{\text{objects}}}$$

The data-flow for this reaction I ∈ R is computed by applying the mass action law with the required input concentrations of related reactants, c(objects). Here,

2 $${\text{c}}\left( {{\text{object}}_{{\text{i}}} } \right) = \prod\limits_{{{\text{c}}({\text{gene}})}} {{\text{reactants}}\; \in \;({\text{reaction}},\;{\text{role}}).{\text{genes}}}$$

The concentration of each reactant is determined by the input of gene expression profile of a corresponding patient. The flux of a pathway P is defined as flux(P), the amount of information flowing through this pathway:

3 $${\text{flux}}\left( {\text{P}} \right) = \left( {\sum\limits_{{{\text{i}} \in {\text{P}}}} {{\text{I}}_{{\text{i}}} \left( {{\text{reaction}},\;{\text{role}}} \right){\text{/N}}\left( {\text{P}} \right)} } \right) - {\text{flux}}\left( {\text{crosstalk(P)}} \right)$$

N(P): the number of reactions of the pathway P; Crosstalk(P): the crosstalks pathways related to the pathway P.

### Statistical analysis

Spearman’s correlation coefficients were calculated to investigate the correlation between the IM index, and other biomarkers and laboratory markers. Logistic regression models were performed to investigate the ability of a marker to distinguish two groups of patients: malignant vs. non-malignant. Only complete observations are considered. For the IM-index and each biomarker, a logistic regression model was fitted separately. Receiver operating characteristic (ROC) analyses were conducted to compare their performance. Area under the curve (AUC) and the 95% confidence intervals were calculated. Odds ratio (OR) was calculated to reflect the prediction rate. With the null hypothesis that the biomarker gives equal or better classification than the index, one-sided hypothesis test was performed and bootstrapped *p*-value of the test was calculated (with 2,000 replications). Kruskal–Wallis test and Wilcoxon test were performed to analyze the index-based classification. For all analyses, *p* values < 0.05 were considered to be statistically significant. *p* values were not adjusted for multiple testing because of the explorative manner of the analyses. Statistical analyses were performed using R software^15^.

## Result

### Clinical characteristics of participants

The median age of participants in original cohort was 58.0 years old (interquartile range [IQR] 52.0–64.0), sex and smoking status were almost equally distributed with slightly more male than female patients (57.6% vs. 42.4%) and slightly more non-smokers than smokers (59.6% vs. 40.4%). In this original cohort, 78 patients were diagnosed with malignant (78/99, 78.8%) and 21 with benign pulmonary nodules (21/99, 21.2%) by standard diagnostic test (Table 1). In addition, 10 healthy participants, who attended routine medical check in Tongji Hospital, were included and defined as control cohort, with a mean age of 32.5, no smoking history, no comorbidity, and 60% female. Of the 99 pulmonary nodules in the original cohort, post-operative histopathological analysis categorized 78 (78/99, 78.8%) as malignant, having aggressive tumor tissue development with dysplastic epithelial / invasive carcinoma at the margin and carcinoma in situ at the center; immunohistochemistry confirmed this malignant signature (Fig. 1B). Twenty-one nodules (21/99; 21.2%) were categorized as benign, having a low aggressive / adequate tissue development with normal epithelial at the margin and dysplastic epithelial at/near by the center (Fig. 1C). Among these, 57.1% (12/21) had at least one elevated tumor marker or malignant dysplasia sign (intraoperative frozen sections), and 76.2% (16/21) had nodules over 1.5 cm in diameter. These clinical features were consistent with the characteristics of the equilibrium phase of the CIC. Within the malignant group, patients were mainly in stage I (52/78, 66.7%) of disease, smaller numbers were in stages II (10/78, 12.8%), III (13/78, 16.7%) and IV (3/78, 3.8%). By histological analysis, the majority of tumors were adencarcinoma (52/78, 66.7%) or squamous carcinoma (19/78, 24.4%), moderately differentiated (33/63, 52.4%), and positive for EGFR mutation (19/30, 63.3%). Furthermore, in the malignant and benign groups, pulmonary nodule number and size were measured from three perspectives (imaging, surgery, and pathology) and showed a median maximum nodule diameter of 2.3 cm, 2.5 cm, and 2.2 cm respectively. Protein levels of five lung cancer-specific biomarkers were measured in tumor tissues and peripheral blood specimens from patients in the benign and malignant groups. Counts of different immune cells in the tumor microenvironment (TME) and the peripheral blood were also measured for both groups (Table 1). Additionally, an independent validation cohort was subsequently recruited, which consists of 30 patients with malignant pulmonary nodes and 10 with benign pulmonary nodes. Clinical characteristics of this validation cohort were summarized in the Table 1 and Supplement Table 1. After acquisition of each blood sample and performance of the differential cell count, leukocytes were isolated and preprocessed for RNAseq analysis (Methods).

### Analysis of differential expression genes in three participant-groups

Based on our understanding of the CIC, we hypothesized that the function of the immune system should vary between normal, benign and malignant groups. Thus we initially investigated the differentially expressed genes from peripheral blood leukocytes from these groups. For this purpose, the DEseq2^16^ was applied to analyze RNAseq data. The results showed that 190 genes (161 upregulated, 29 downregulated) were differentially expressed by comparing between malignant and benign groups (LogFC > 2 & Bonferroni correction < 0.05; Fig. 1D). Between malignant and control groups 3101 genes (3072 upregulated, 29 downregulated) were differentially expressed (the same criteria; Fig. 1E). Subsequently, the gene ontology (GO) enrichment analysis showed that the differential expressed genes from each set were not enriched in any of the three main ontological categories (biological process, cellular component, and molecular function). The common set between both upregulation-gene sets (161 ∩ 3072) contained 125 genes. However, the GO enrichment analysis with this set showed no commonality. Similarly, both downregulation-gene sets (29 ∩ 29) shared no common genes (Fig. 1D,E).

### Application of genetic information from peripheral blood leukocytes for malignancy diagnosis

**Figure 2:**
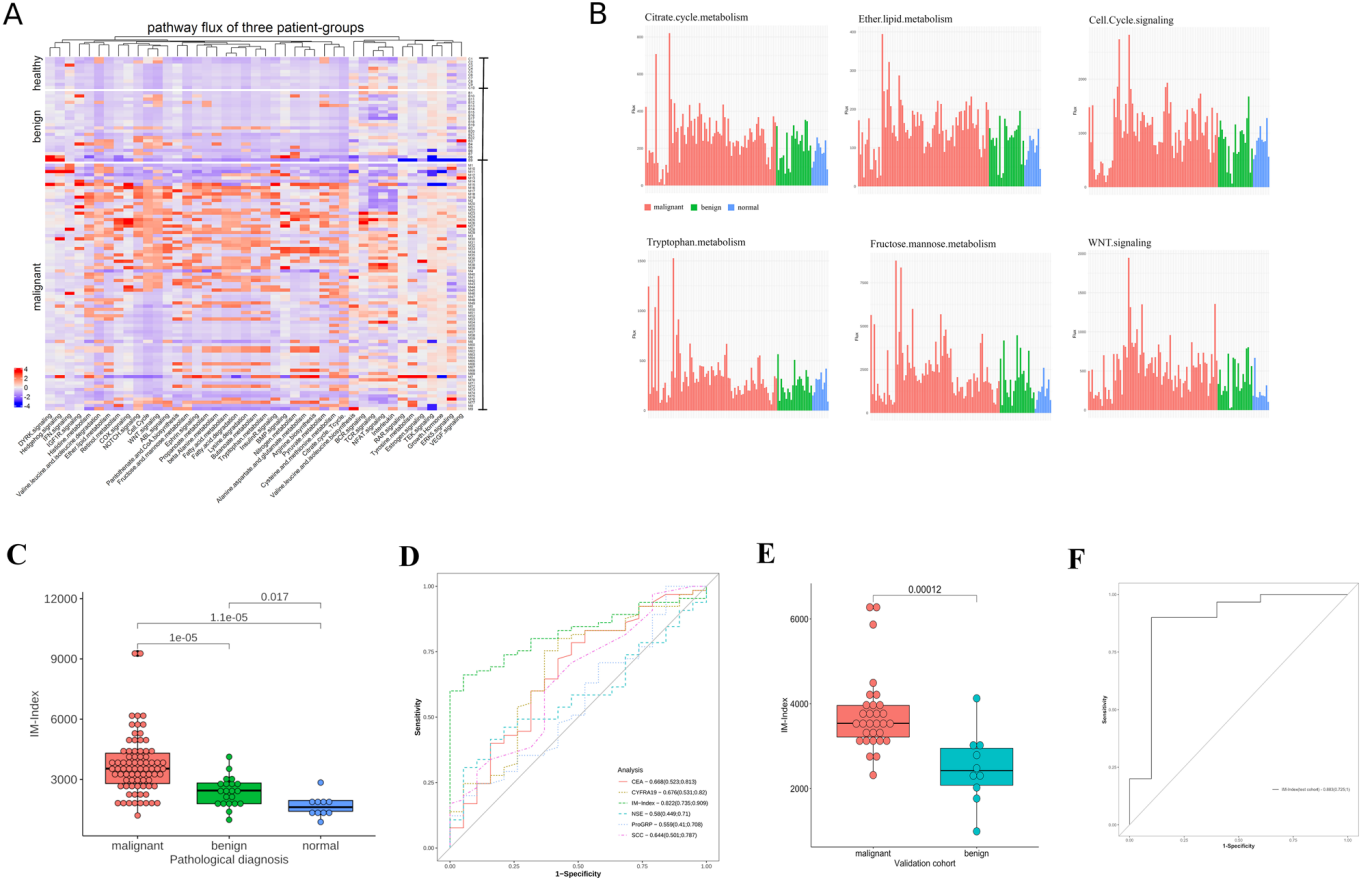
(**A**) Heatmap of the differentiated flux of 43 molecular pathways in three participant-groups; (**B**) top 6 pathways selected from the 43 molecular pathways from (**A**). (**C**) Boxplot of the immune-related index (IM-Index) for malignant (red), benign pulmonary nodules (green), as well as healthy controls (blue). The *p*-value obtained through the Kruskal–Wallis test showed statistical significance with *p* < 0.001. (**D**) ROC analysis results comparison of IM-Index (AUC: 0.822) and 5 lung cancer-specific biomarkers. (**E**) Boxplot of IM-Index comparison between malignant and benign subgroups in validation cohort; (**F**) ROC analysis of IM-Index in the validation cohort.

To determine if differences between participant groups could be observed on a molecular pathway level, a genome-scale pathway flux analysis (GPFA) was performed using an artificial intelligence (AI) model (Methods). Pathway flux was calculated by AutoAnalysis^37^ based on RNAseq of peripheral blood samples from each participant. Results showed that flux in 43 pathways significantly differentiated between the three groups (*p* < 0.05; Fig. 2A). In particular, the malignant group had a strong flux intensity in the majority of these analyzed pathways such as tryptophan metabolism and citrate-cycle (Fig. 2B & Supplement Fig. 2), whereas almost no pathway with any enhanced flux activity was evident for the healthy controls (Fig. 2A; Supplement Fig. 2). Flux in the benign group was in between the normal and malignant groups (Fig. 2A; Supplement Fig. 2).

Based on these results, an index system was defined to summarize flux intensities of these pathways, which derived from the gene expression of 3643 genes.

The index system was termed immune-related index (IM-Index) using following definition:

$${\text{IM - Index}} = \alpha *\sum\limits_{{{\text{p}}\; \in \;{\text{signaling}}\;{\text{transduction}}}} {flux(P) + \beta *\sum\limits_{{{\text{p}}\; \in \;{\text{energy}}\;{\text{metabolism}}}} {flux(P)} } \quad \;\left( {{\text{Supplement}}\;{\text{Information}}\;2} \right)$$

The default values of *α, β* are set to 1 in this study. The Kruskal–Wallis test showed that IM-Index could differentiate between the three groups (OR: 1.17 [95% CI: 1.1–1.25, *p* < 0.001]; Fig. 2C), the median of IM-Index from the malignant, benign and control groups were: 3429.3*, 2483.4, 1869.8* respectively. The Receiver Operating Characteristic (ROC) analysis showed that IM-Index was able to differentiate between benign (n = 21) and malignancy (n = 78) pulmonary nodules with an AUC: *0.822 (95% CI: 0.75–0.91, p* < *0.001)* (Fig. 2D). Moreover, IM-Index out-performed five different lung cancer-specific peripheral blood biomarkers (CEA, CYFRA19, NSE, ProGRP, and SCC) for malignant cancer diagnosis (Fig. 2D; Table 2). In the validation cohort, the Kruskal–Wallis test showed that IM-Index could differentiate between malignant and benign groups (OR: 1.31 [95% CI: 1.18–1.43, *p* < 0.001] ; Fig. 2E) and the ROC analysis showed the corresponding result of AUC: 0.883 (95% CI: 0.73–1.00, *p* < 0.001; Fig. 2F).

**Figure 3:**
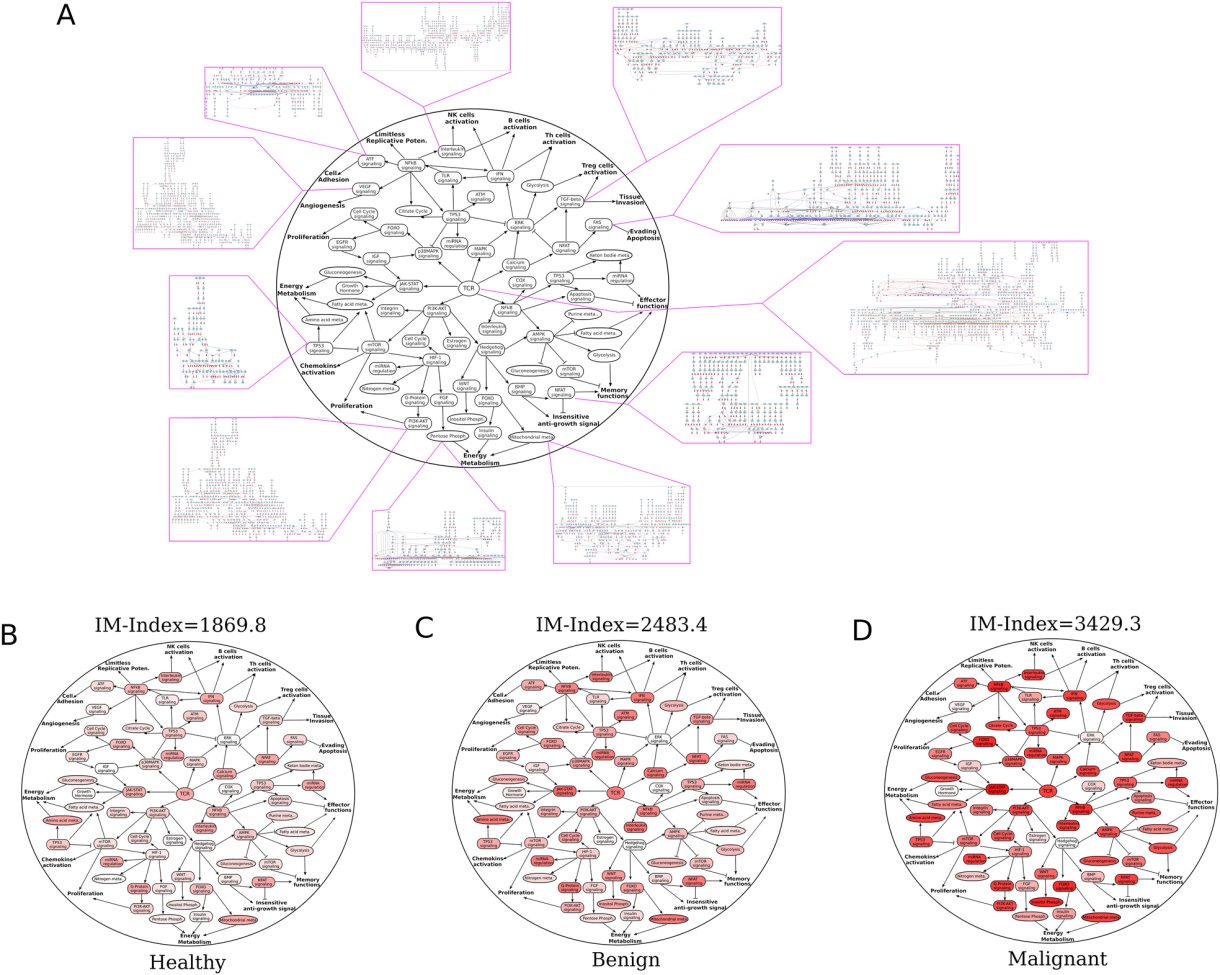
Schematic Immune-related Pathway Interaction Map and IM-Index. (**A**) A simplified overview of the abstract signaling interaction and crosstalk between different signaling and metabolic pathways in the artificial intelligent (AI) model. The map is termed PIML (pathway interaction map of leukocyte). Each element symbolizes a corresponding pathway containing up-, middle-, and downstream components including gene, RNA, protein, compound, and complex. (**B**–**D**) three PIMLs visualize these three groups with median IM-Index respectively.

In addition, a simplified pathway interaction map of leukocyte (PIML; Fig. 3A) within the AI model was created to visualize pathway flux in these three groups. These PIMLs represent the unique pathway signature three groups (healthy, benign and malignant) respectively (Fig. 3B–D), which could represent unique signatures for each phase of the CIC in lung cancer.

**Table 2:** AUC value comparison between IM-Index and 5 other lung cancer-specific biomarkers.

Comparison	AUC of the index	AUC2 of the biomarker	Bootstrapped *p*-value
IM-Index vs. CEA	0.822	0.668	0.029
IM-Index vs. NSE	0.822	0.58	0.002
IM-Index vs. CYFRA19	0.822	0.676	0.034
IM-Index vs. SCC	0.822	0.644	0.01
IM-Index vs. PROGRP	0.822	0.559	0.002

### Using the IM-index and leukocyte RNAseq to distinguish malignant lung cancer subtypes

**Figure 4:**
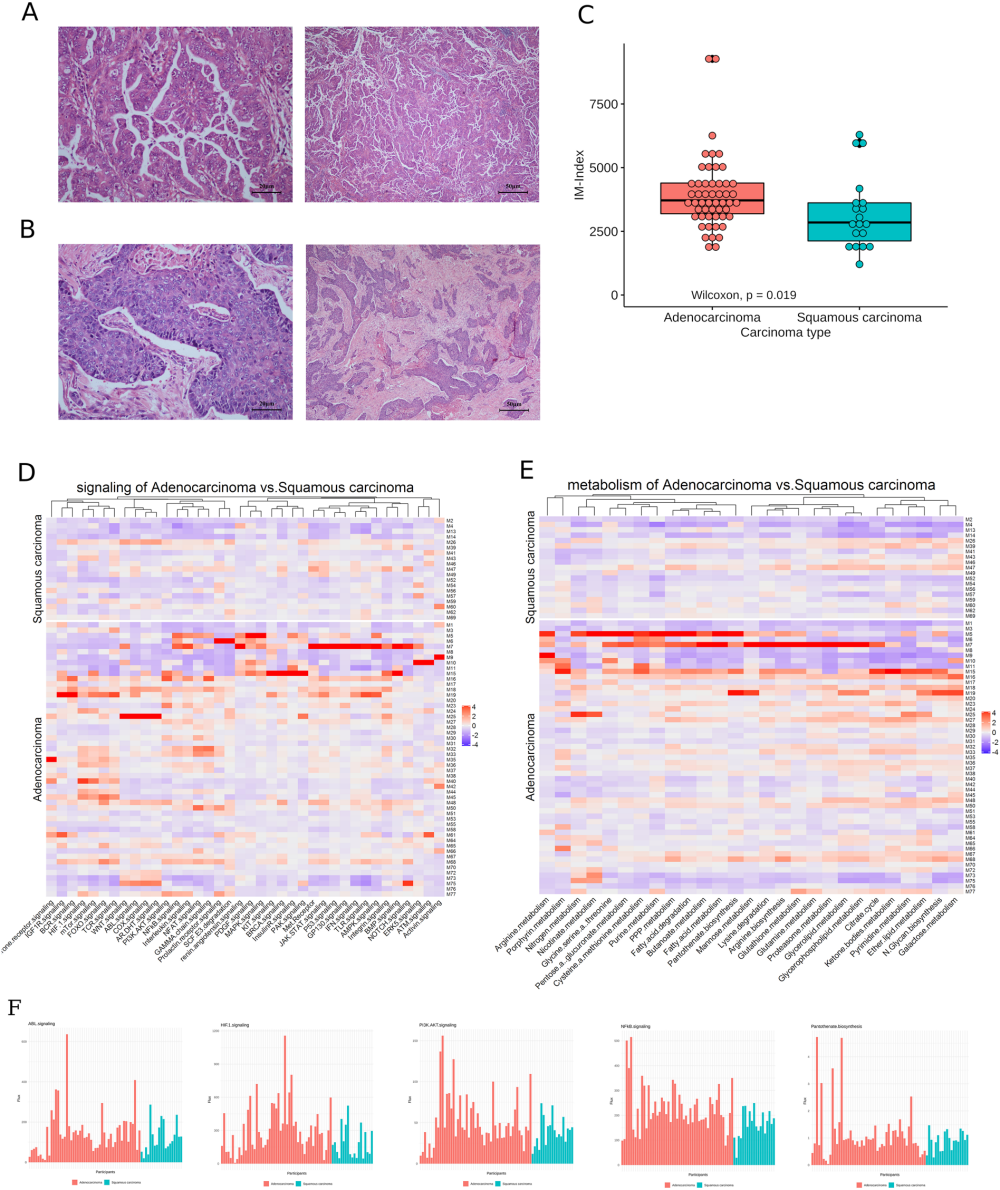
Histopathology of adenocarcinoma and squamous carcinoma tissues and differentiation between both sub-groups. (**A**) Haematoxylin and eosin (H&E) stained tissue sample of a participant (M11) showing adencarcinoma tissue at the center (left) and widespread malignancy (right) (**B**) H&E stained tissue sample of a participant (M43) showing squamous carcinoma at the center (left) and at the margin (right). (**C**) Boxplot of the immune-related index (IM-Index) for adenocarcinoma (red) and squamous carcinoma sub-groups (cyan). The *p*-value obtained through the Wilcoxon test showed statistical significance with *p* = 0.019. (**D**,**E**) Heatmap of the differentiated activities (fluxes) of signaling- and metabolic pathways for both sub-groups. (**F**) five differentiated signaling pathways between adenocarcinoma and squamous carcinoma groups.

A refined analysis focused tumor/host interactions on the malignant group. From the tumor side, postoperative pathological analysis showed that participants of lung adenocarcinoma, 66.2% of the malignant group, often had origin from bronchial mucosa and grew later in the small bronchi of the lung (Fig. 4A). The disease progression was often aggressive. The participants with lung squamous carcinoma, 24.7% of the malignant group, originated mostly from larger bronchi and were often associated with bronchoonstriction and obstructive pneumonia (Fig. 4B). The majority of this sub-group had a history of smoking and the mean age of this group is 67 years old. In general, the disease progression was milder.

The IM-Index could differentiate both types of lung cancer with statistical significance (OR: 1.2 [95% CI: 1.14–1.35, *p* = 0.019]; Fig. 4C). GPFA showed that signaling pathways mTor (bonferroni correction, *p* = 0.02), HIF (*p* = 0.02), FOXO (*p* = 0.01), PI3K-AKT (*p* = 0.04), integrin (*p* = 0.04), JAK-STAT (*p* = 0.01) and others displayed a higher flux in the adenocarcinoma sub-group compared to the squamous cell carcinoma group (Fig. 4D,F). In addition, the metabolic pathways such as citrate-cycle (*p* = 0.001), lipid (*p* = 0.003), amino acid (*p* = 0.002) and glycolysis (*p* = 0.001) showed a higher flux in the adenocarcinoma sub-group (Fig. 4E; Supplement Fig. 3). These findings demonstrate that development of adenocarcinoma is a signaling-intense and metabolically demanding process and likely reflects the more aggressive nature of this type of lung cancer.

### Lung cancer pathogenesis: immune-related pathway activity of host versus tumor

**Figure 5:**
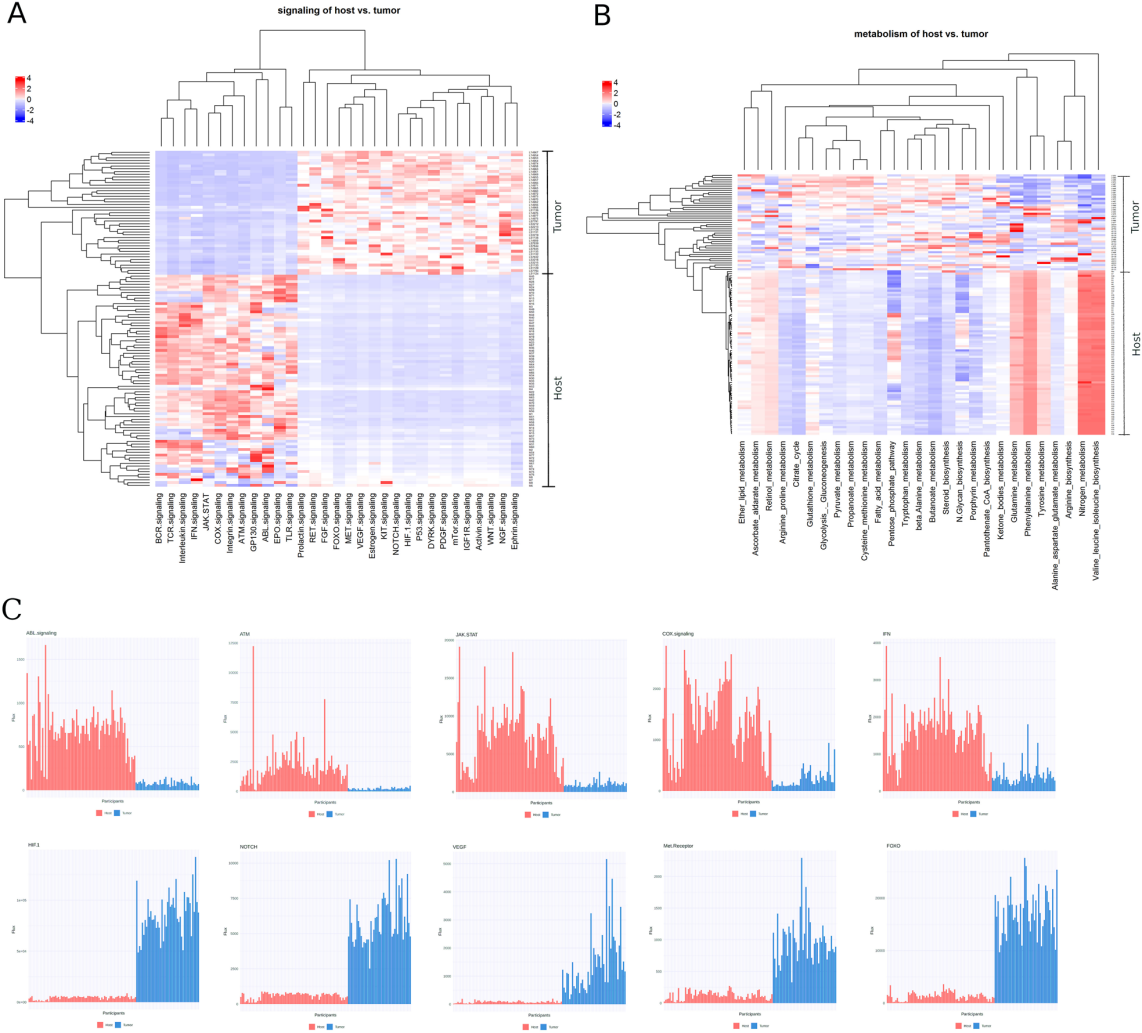
Heatmap of signaling and metabolic pathways comparing host- and tumor-side from the malignant tissue and peripheral blood samples. (**A**) Comparison of flux activities within signaling pathways in the AI model between host- and tumor-side from lung cancer patient samples. (**B**) Flux activities of metabolic pathways in the AI model between host- and tumor-side. (**C**) Ten highly differentiated pathways for the comparison between host- and tumor-side.

The gene expression profiles from biopsies were collected from a previous study^37^, and then integrated into the AI model. AutoAnalysis was performed with the model to calculate pathway fluxes for each biopsy and subsequently GPFA was performed. The results were divided into separate signaling (Fig. 5A) and metabolic (Fig. 5B) perspectives. High and low signaling pathway activities (high vs low pathway flux) were clearly differentiated from one another and such pathways could distinguish between host (peripheral blood leukocytes) and tumor responses. For instance, the signaling pathways VEGF (bonferroni correction, *p* = 4.76e−17), PDGF (*p* = 8.81e−16), and FGF (*p* = 2.94e−18) all showed a higher activity (high flux) on tumor-side (Fig. 5A; Supplement Fig. 4), which potentially reflects a sustained angiogenesis^39,40^. The mTor (*p* = 5.74e−17) and WNT (*p* = 2.44e−16) pathways were also more highly activated on tumor-side (Fig. 5A; Supplement Fig. 4), which may be associated to the uncontrolled proliferation^41,42^. The signaling pathways HIF (*p* = 1.32e−17), FOXO (*p* = 7.88e−14) and NOTCH (*p* = 1.52e−17), associated with tissue invasion were also more highly activated on the tumor-side^43–45^. In contrast, signaling pathways associated with a highly functioning innate immune system (TLR, COX)^46,47^, the functionality^48^, differentiation^19^, and maturation^49^ of T-Lymphocytes (JAK-STAT, EPO, Integrin), and the functionality of the T- and B-cell^50,51^ (TCR, BCR, Interleukin, ATM) were more highly activated on the host-side (peripheral blood) (*p* < 0.05; Fig. 5A; Supplement Fig. 4).

From the metabolic perspective, it is shown that there is a clear and structural metabolism pattern for the host-side (Fig. 5B; Supplement Fig. 4): relatively high activities of valine, leucine and isoleucine biosynthesis (*p* = 7.08e−18), nitrogen (*p* = 3.12e−17), glutamine and phenylalanine metabolism (*p* = 3.43e−18); relatively low activities of butanoate metabolism (*p* = 1.06e−18), beta-alanin metabolism (*p* = 1.15e−18) and citrate cycle (*p* = 1.16e−18). In contrast to the host-side (peripheral blood), a clear pattern in its metabolic pathways did not become evident on the tumor-side (Fig. 5B; Supplement Fig. 4).

## Discussion

The current study combined peripheral blood leukocyte-based RNAseq data with an AI model as a rapid, non-invasive tool for early diagnosis of malignancy in pulmonary lung nodules. Current knowledge of biochemical signaling and metabolic pathways was collected and applied to inform the AI model. This AI model is unique because its construction is only driven by the biological knowledge, and not influenced by any existing genetic data. The model, facilitated with flux-flow based algorithm from AutoAnalyze, was used to calculate fluxes in pathways involved in host immunity. Based on these results an immune-related index (IM-Index) was defined and tested for its diagnostic potential. The IM-Index clearly differentiated between patients with malignant versus benign pulmonary nodules with a high accuracy (AUC: *0.822* [*95% CI: 0.75–0.91*] *p* < *0.001;* Fig. 2). These results demonstrate for the first time that combining genetic information from peripheral blood leukocytes with AI modeling can accurately define the malignancy status of pulmonary nodules. The result in the validation cohort analyzed with IM-Index further confirmed its predictive ability. These results suggest that this approach could be used successfully for early clinical diagnosis and potentially the response to treatment. Further, by clearly differentiating between patients with benign nodules and healthy controls, the IM-Index may be able to distinguish the three phases of cancer pathogenesis as defined by the Cancer Immuno-editing Concept (CIC).

In addition, this approach provided novel mechanistic insight into the host/tumor relationship. For instance, signaling pathways such as WNT^52^, NOTCH^53^, ERK^54^ and others were significantly and uniquely activated in the malignant group, which likely reflects a robust host immune response to aggressive tumor behavior (Fig. 2A). Similarly, malignancy was associated with increased metabolic activation in leukocytes, including nitrogen^55^, fatty acid^56^, cysteine, and methionine^57^.

Smaller differences in pathway fluxes (activities) between the benign and control groups further imply that early, more subtle changes in host immunity may occur during the equilibrium phase. This finding may necessitate addition of more refined stages to the current CIC definition.

Several recent studies have attempted to extract information from peripheral blood leukocytes for diagnosis in lung cancer^58^, however, these focused solely on the expression levels of a few selected proteins and did not investigate the overall functional status of host immunity. In contrast to such studies, our work detailed a broad range of metabolic and signaling pathways unique to the host- and tumor-side of cancer pathogenesis on a systems level. Specifically, pathways that enforce proper immune functioning were activated on the host-side of the equation. On the tumor-side, cells strongly exhibited the hallmarks for malignancy. This supports our notion that signaling- and metabolic-pathway related information derived from host immune cells could be used to aid in sustaining a rapid and accurate assessment of host immune function. By contrast, chaotic and unstructured patterns of signaling and metabolism were found on the tumor-side, likely reflecting uncontrolled cellular proliferation and tumor growth.

These dramatic and dynamic differences could be exploited to develop novel therapeutic interventions that shift the balance toward host immunity and tumor cell elimination. Such therapeutic intervention targeting host versus cancer metabolism could be made on a personalized level. For example, the pentose-phosphate pathway (PPP), steroid biosynthesis, fatty-acid metabolism and citrate cycle all showed relatively high activities in the majority of tumor samples, a strong indication of heightened metabolic demands (Fig. 5). As these demands accelerate, these metabolic pathways may offer vulnerable targets and time points when treatment may be especially effective. Changing metabolic and signaling patterns during and after therapy could also indicate treatment efficacy, host immune status and overall prognosis.

Cancer research has entered the era of “Big Data”. The key challenge is how to combine large-scale, high-dimensional and complex data for clinical applications. The recent development of artificial intelligence (AI) may provide a novel approach to meet this key challenge. Multiple studies have attempted to develop AI models to reduce dimensionality of big data and extract high-level features for prediction purposes^31–33^, which ultimately leads to the focus on the selection of training data. This may intricately increase the risk of over-parameterization. The present study definitely shows that an unbiased AI model can be constructed with solely a knowledge-based platform and that this model can be successfully applied using understand complex biological systems such as the human immune system. Further, pathway flux analysis is an invaluable approach to manage bio data’s the “curse of dimensionality”^59^ of big data.

There are several limitations to our study. First, although our IM-Index represents the latest biochemical signaling and metabolic information regarding strength and function of the host immunity, this field is complex and extensive, with new knowledge constantly evolving. Thus, the IM-Index definition will require consistent updating. Second, the current parameters *α, β* of IM-Index were set to a default value of 1 for this study since relevant information to define more precisely these parameters are lacking. Both parameters may also vary based on the type of cancer. It is also possible that pathway defined within this index system need to be parameterized. Therefore, follow-up studies may provide information to refine our model and increase its diagnostic accuracy and sensitivity. Third, although the AutoAnalysis does not strictly depend on kinetic parameters, such parameters in the AI model were derived from different kinetic databases. Future work will focus on the improvement of kinetic parameter settings in a more systematical and consistent way to meet the updated version of AI model. Fourth, there are several assumptions within the AI model including that the concentrations of H_2_O, and O_2_ remain constant during the simulation in AutoAnalysis. Lastly, the number of patients studied is limited and group size is not equally distributed, thus limiting the statistical power. In addition, other types of lung cancer have not been included.

In light of our findings and their significant potential for early diagnosis of malignant lung cancer, additional prospective studies in a larger, multicenter cohorts of patients having various types of lung and non-lung cancers are warranted.

## Conclusion

Our findings demonstrate that a non-invasive peripheral blood leukocyte-based AI model with IM-Index could be used clinically to differentiate sub-types of malignant lung cancer. Further, this approach provides important information regarding lung cancer pathogenesis and host/tumor interactions that could be exploited for development of novel treatment modalities.

## Supplementary Information

Supplementary Information 1.

Supplementary Information 2.

Supplementary Information 3.
