# Supplementary material for: Differential early diagnosis of benign versus malignant lung cancer using systematic pathway flux analysis of peripheral blood leukocytes
Source: Sci Rep. 2022 Mar 24;12:5070. doi: 10.1038/s41598-022-08890-x (PMC8948197; doi:10.1038/s41598-022-08890-x)
Supplement: Supplementary file 1 — Supplementary Information 1. [file 41598_2022_8890_MOESM1_ESM.pdf]

Supplement Information / Figures / Tables

Differential Early Diagnosis of Benign vs Malignant Lung Cancer using Systematic Pathway Flux Analysis of Peripheral Blood Leukocytes

Jian Li[1,+], Xiaoyu Li[2,+], Ming Li[3,+], Hong Qiu[2], Christian Saad[4], Bo Zhao[5], Fan Li[5], Xiaowei Wu[5], Dong Kuang[6,7], Fengjuan Tang[6,7], Yaobing Chen[6,7], Hongge Shu[8], Jing Zhang[8], Qiuxia Wang[8], He Huang[9], Shankang Qi[9], Changkun Ye[10], Amy Bryant[11], Xianglin Yuan[2], Christian Kurts[1], Guangyuan Hu[2,*], Weiting Cheng[12,*], Qi Mei[2,*]

[1] Institute of Molecular Medicine and Experimental Immunology, University Clinic of Rheinische Friedrich-Wilhelms-University, Bonn, Germany
[2] Department of Oncology, Tongji Hospital, Tongji Medical College, Huazhong University of Science and Technology, Wuhan, Hubei, People's Republic of China
[3] Department of Oncology, Wuhan Pulmonary Hospital, Wuhan, Hubei, People's Republic of China
[4] Department of Computer Science, University of Augsburg, Augsburg, Germany
[5] Department of thoracic surgery, Tongji Hospital, Tongji Medical College, Huazhong University of Science and Technology, Wuhan, Hubei, People's Republic of China
[6] Institute of Pathology, Tongji Hospital, Tongji Medical College, Huazhong University of Science and Technology, Wuhan, Hubei, People's Republic of China
[7] Department of Pathology, School of Basic Medicine, Tongji Medical College, Huazhong University of Science and Technology, Wuhan, Hubei, People's Republic of China
[8] Radiology department, Tongji Hospital, Tongji Medical College, Huazhong University of Science and Technology, Wuhan, Hubei, People's Republic of China
[9] Shanghai Institute of Materia Medica, Chinese Academy of Sciences, Shanghai, People's Republic of China
[10] Medical Research Center of Yu Huang Hospital, Yu Huang, Zhejiang, People's Republic of China
[11] Department of Biochemical and Pharmaceutical Sciences, College of Pharmacy, Idaho State University
[12] Department of Oncology, Wuhan No. 1 Hospital, Wuhan, Hubei, People's Republic of China
[*] Corresponding Authors: h.g.y.121@163.com, joycvt@126.com, borismq@163.com
[+] Contributing equally authors

Supplement Figure 1: **A**: the internal database diagram of the artificial intelligent (AI) model; **B**: the miRNA modeling pattern in the AI model. **C**: two array digital structure of the AI model;

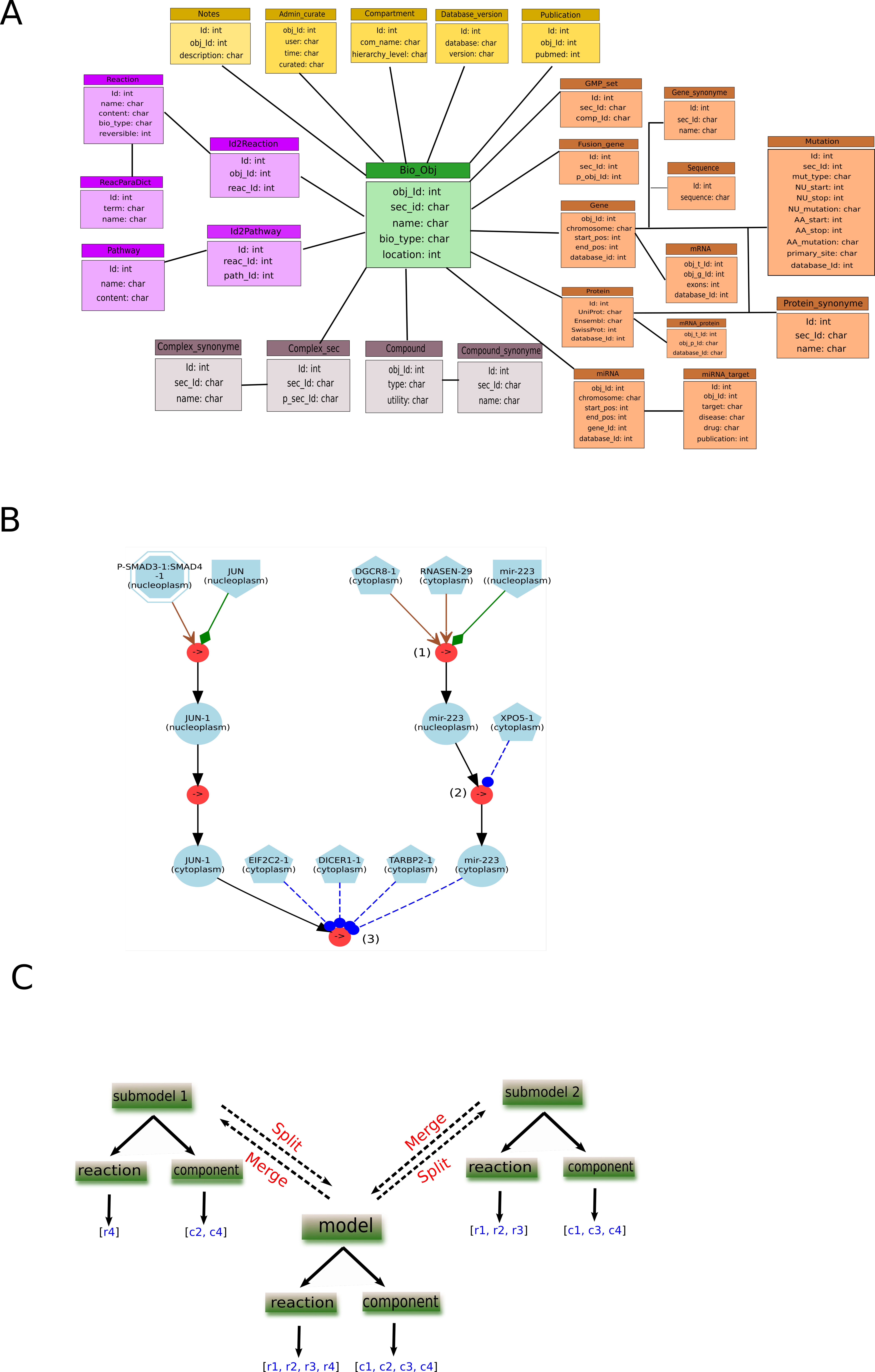
A
Notes
Id: int
obj_Id: int
description: char
Admin_curate
obj_Id: int
user: char
time: char
curated: char
Compartment
Id: int
com_name: char
hierarchy_level: char
Database_version
Id: int
database: char
version: char
Publication
Id: int
obj_Id: int
pubmed: int
Reaction
Id: int
name: char
content: char
bio_type: char
reversible: int
Id2Reaction
Id: int
obj_Id: int
reac_Id: int
ReacParaDict
Id: int
term: char
name: char
Id2Pathway
Id: int
reac_Id: int
path_Id: int
Pathway
Id: int
name: char
content: char
Bio_Obj
obj_Id: int
sec_id: char
name: char
bio_type: char
location: int
GMP_set
Id: int
sec_Id: char
comp_Id: char
Gene_synonyme
Id: int
sec_Id: char
name: char
Fusion_gene
Id: int
sec_Id: int
p_obj_Id: int
Sequence
Id: int
sequence: char
Mutation
Id: int
sec_Id: int
mut_type: char
NU_start: int
NU_stop: int
NU_mutation: char
AA_start: int
AA_stop: int
AA_mutation: char
primary_site: char
database_Id: int
Gene
obj_Id: int
chromosome: char
start_pos: int
end_pos: int
database_id: int
mRNA
obj_t_Id: int
obj_g_Id: int
exons: int
database_Id: int
Protein
Id: int
UniProt: char
Ensembl: char
SwissProt: int
database_Id: int
mRNA_protein
obj_t_Id: int
obj_p_Id: char
database_Id: char
Protein_synonyme
Id: int
sec_Id: char
name: char
Complex_synonyme
Id: int
sec_Id: char
name: char
Complex_sec
Id: int
sec_Id: char
p_sec_Id: char
Compound
obj_Id: int
type: char
utility: char
Compound_synonyme
Id: int
sec_Id: char
name: char
miRNA
obj_Id: int
chromosome: char
start_pos: int
end_pos: int
gene_Id: int
database_Id: int
miRNA_target
Id: int
obj_Id: int
target: char
disease: char
drug: char
publication: int
B
P-SMAD3-1:SMAD4-1 (nucleoplasm)
JUN (nucleoplasm)
DGCR8-1 (cytoplasm)
RNASEN-29 (cytoplasm)
mir-223 ((nucleoplasm)
(1)
JUN-1 (nucleoplasm)
mir-223 (nucleoplasm)
XPO5-1 (cytoplasm)
(2)
JUN-1 (cytoplasm)
EIF2C2-1 (cytoplasm)
DICER1-1 (cytoplasm)
TARBP2-1 (cytoplasm)
mir-223 (cytoplasm)
(3)
C
submodel 1
reaction
component
[r4]
[c2, c4]
Split
Merge
submodel 2
Merge
Split
reaction
component
[r1, r2, r3]
[c1, c3, c4]
model
reaction
component
[r1, r2, r3, r4]
[c1, c2, c3, c4]

Supplement Figure 2: IM-Index related pathway flux plot in the three participant groups.

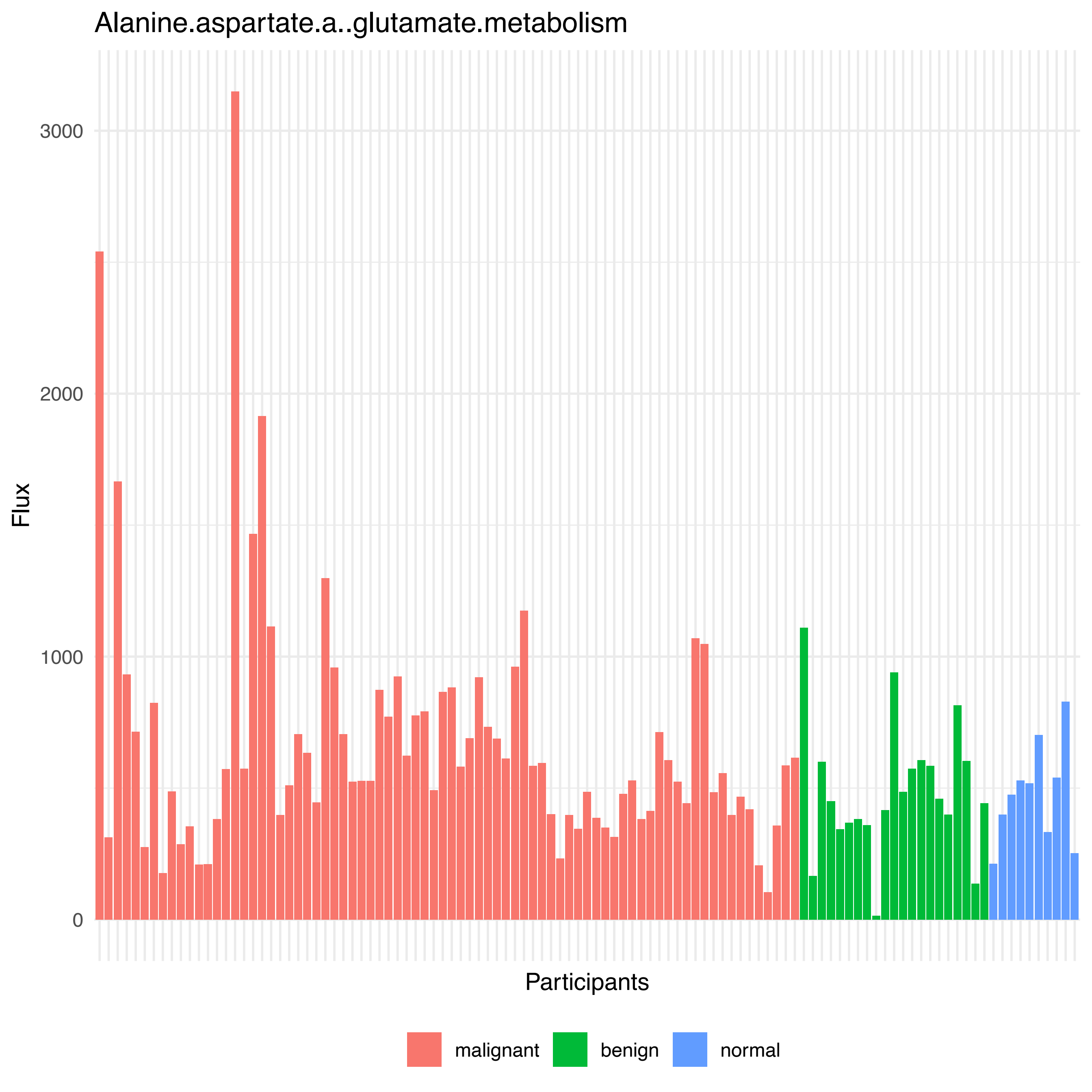

Alanine.aspartate.a..glutamate.metabolism
3000
2000
1000
0
Flux
Participants
malignant
benign
normal

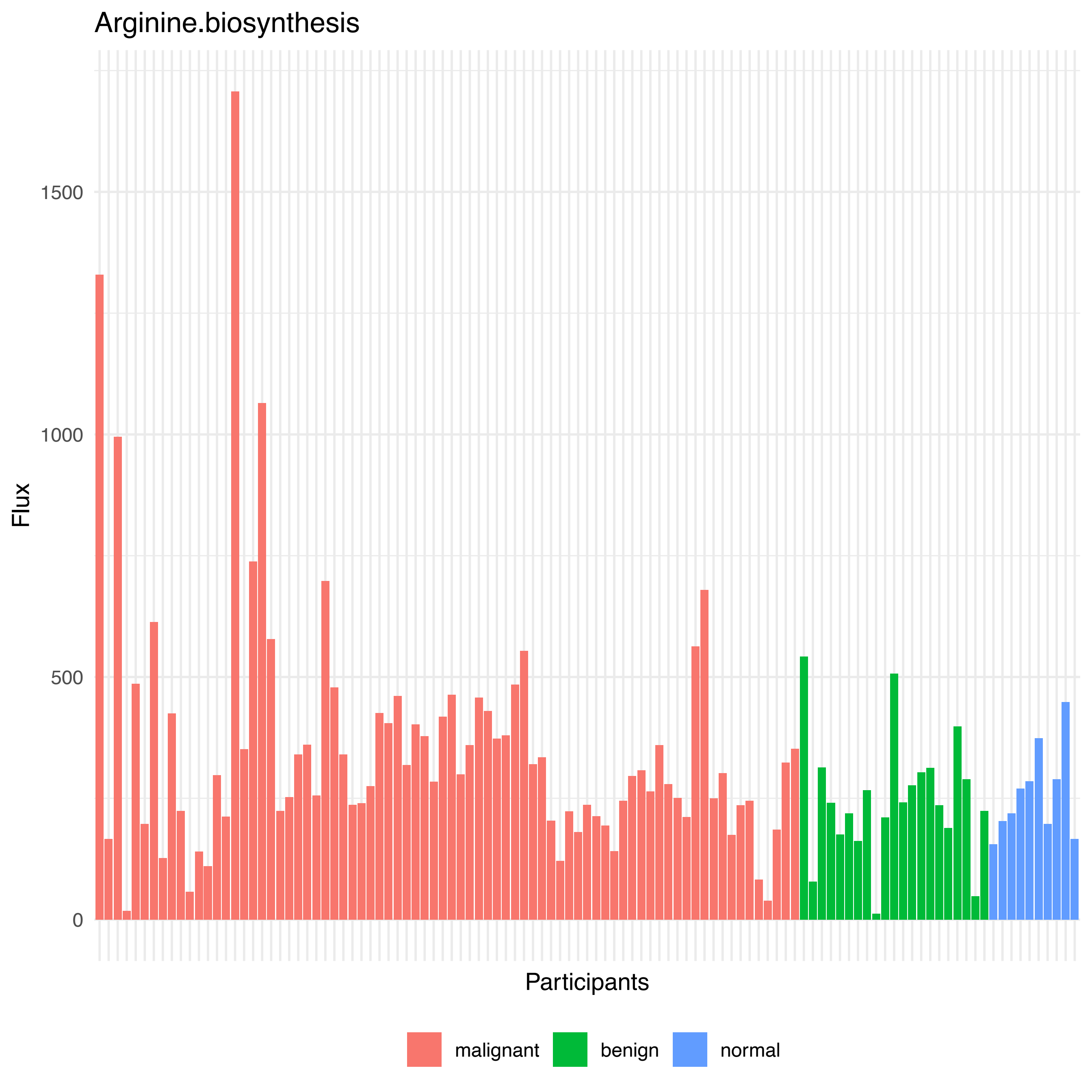

Arginine.biosynthesis
1500
1000
500
0
Flux
Participants
malignant
benign
normal

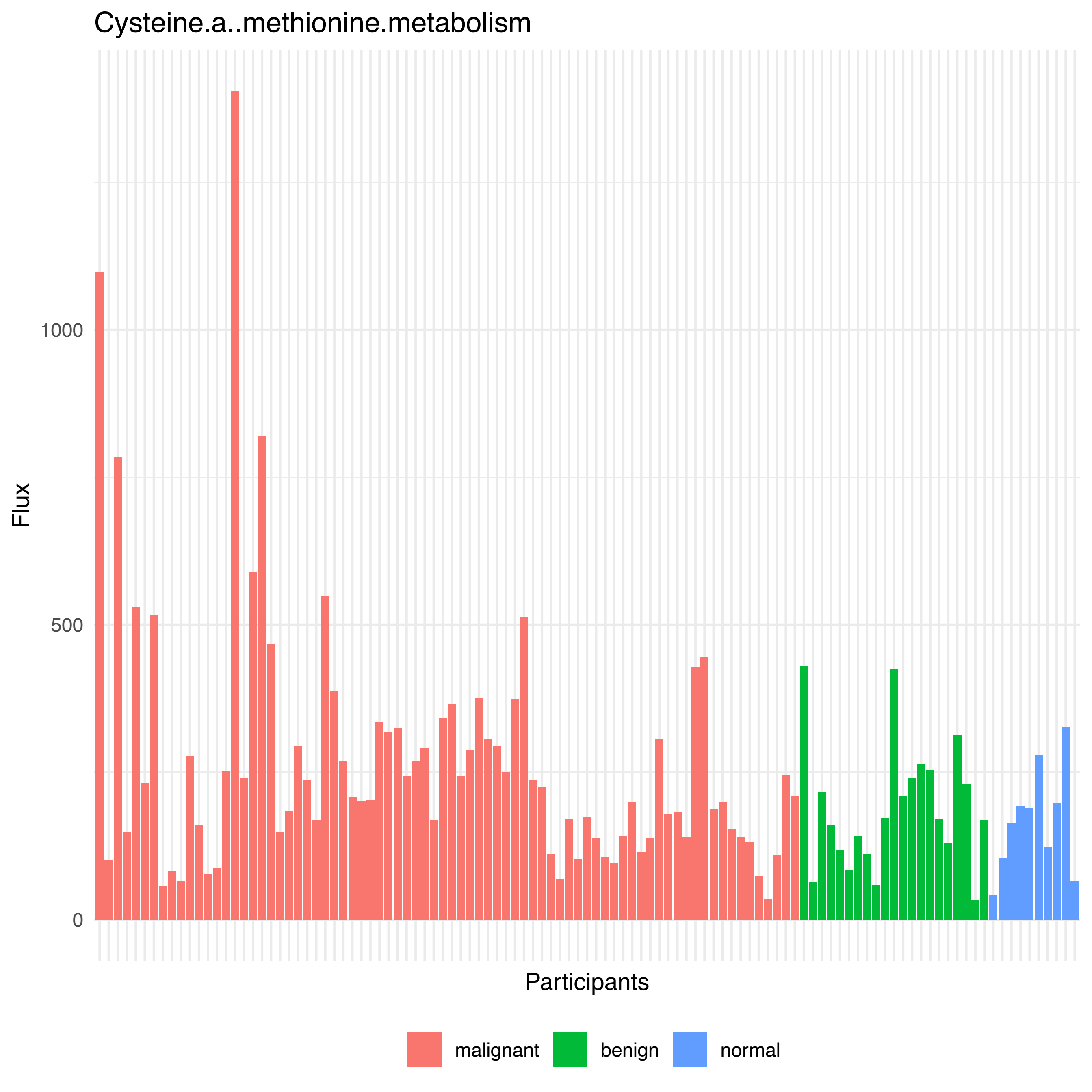

Cysteine.a..methionine.metabolism
Flux
1000
500
0
Participants
malignant
benign
normal

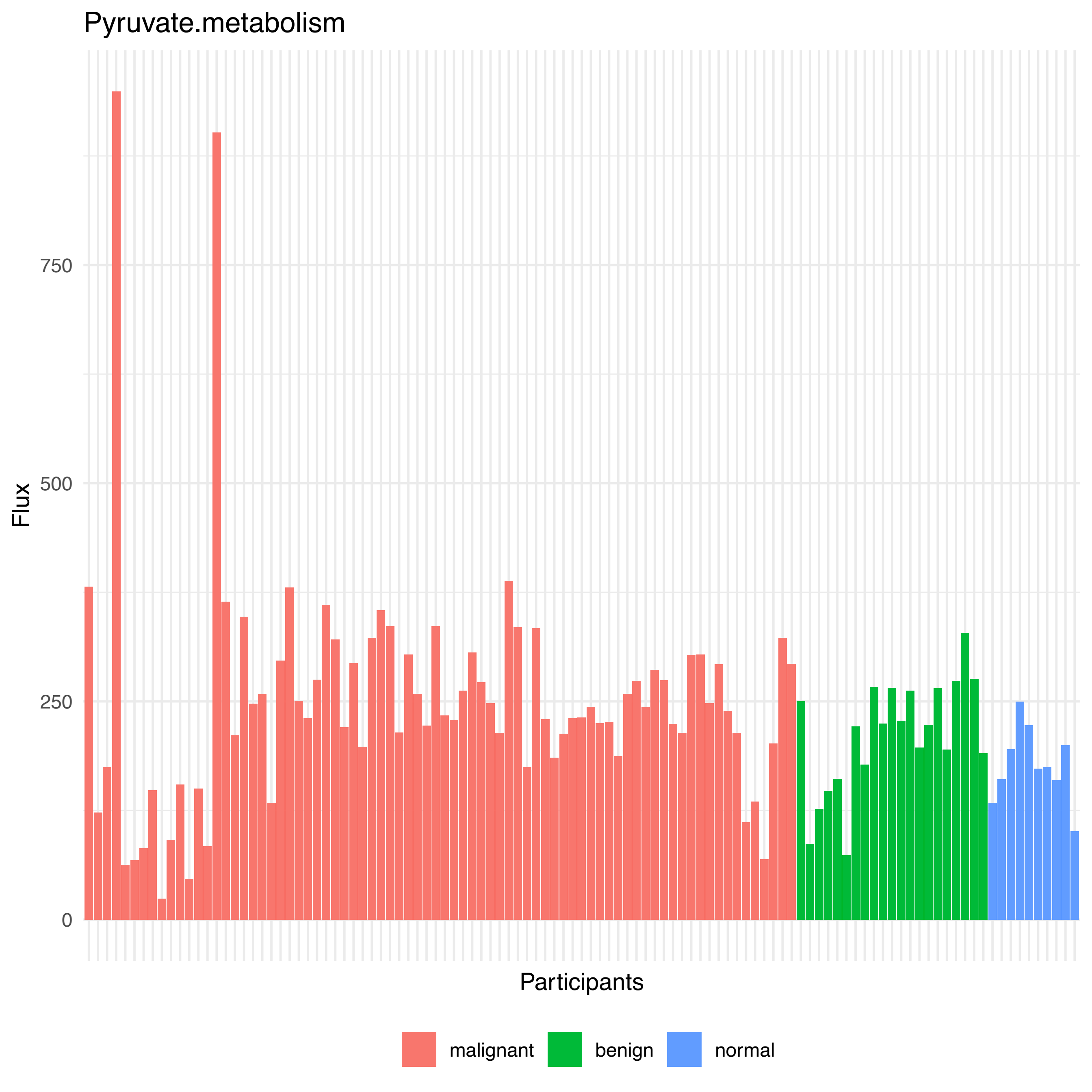

Pyruvate.metabolism
750
500
250
0
Flux
Participants
malignant
benign
normal

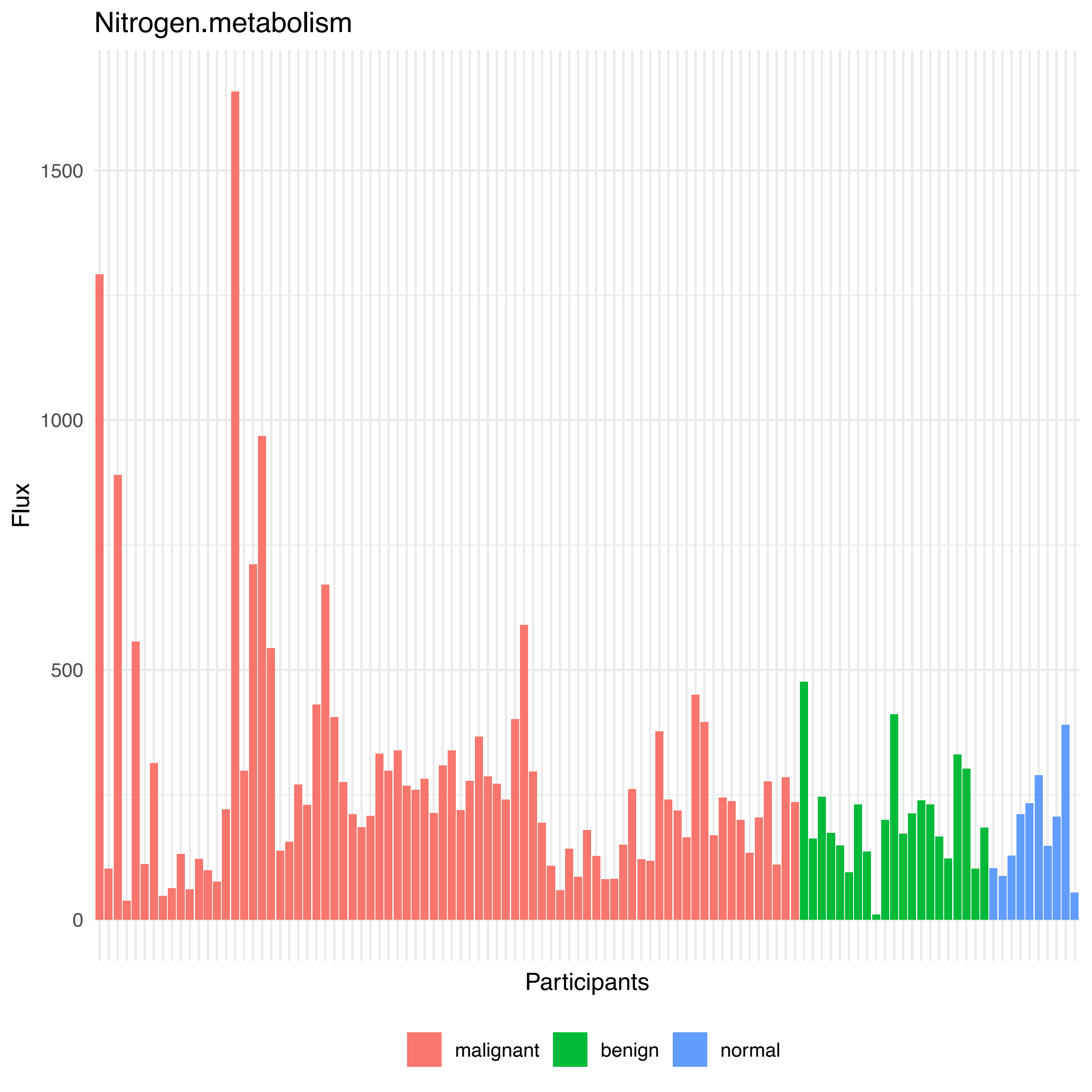

Nitrogen.metabolism
1500
1000
500
0
Flux
Participants
malignant
benign
normal

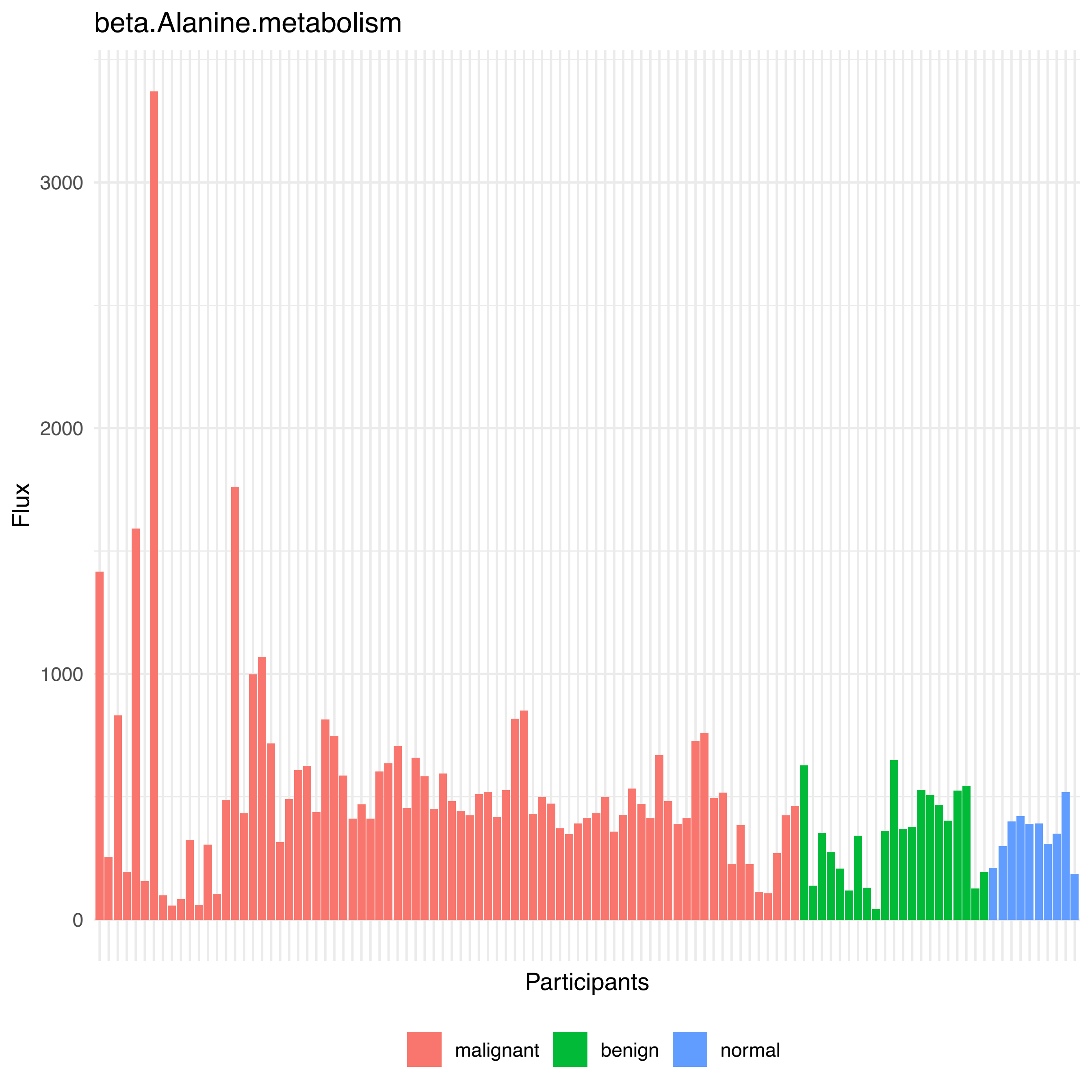
beta.Alanine.metabolism
Flux
3000
2000
1000
0
Participants
malignant
benign
normal

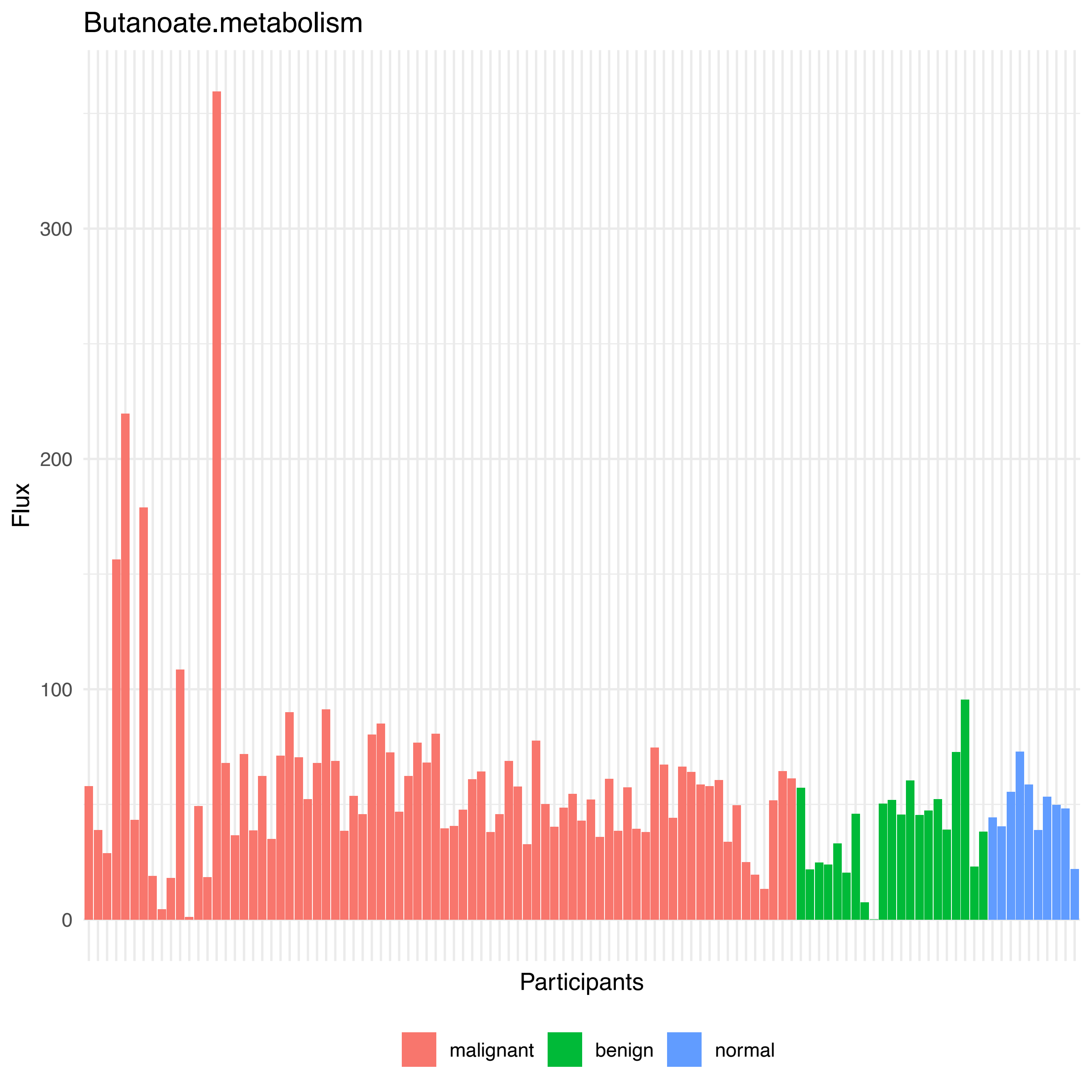

Butanoate.metabolism
Flux
300
200
100
0
Participants
malignant
benign
normal

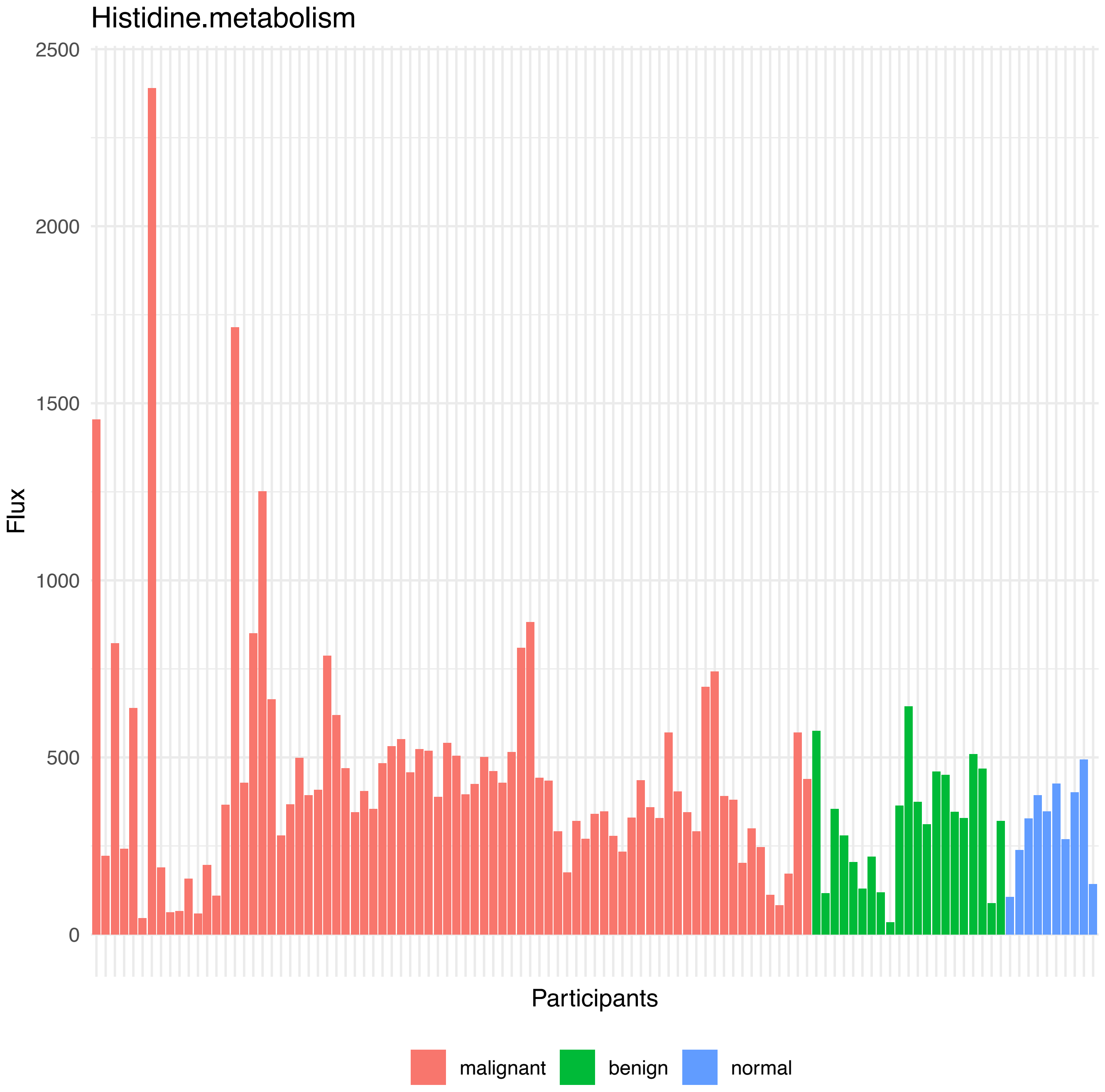

Histidine.metabolism
2500
2000
1500
1000
500
0
Flux
Participants
malignant
benign
normal

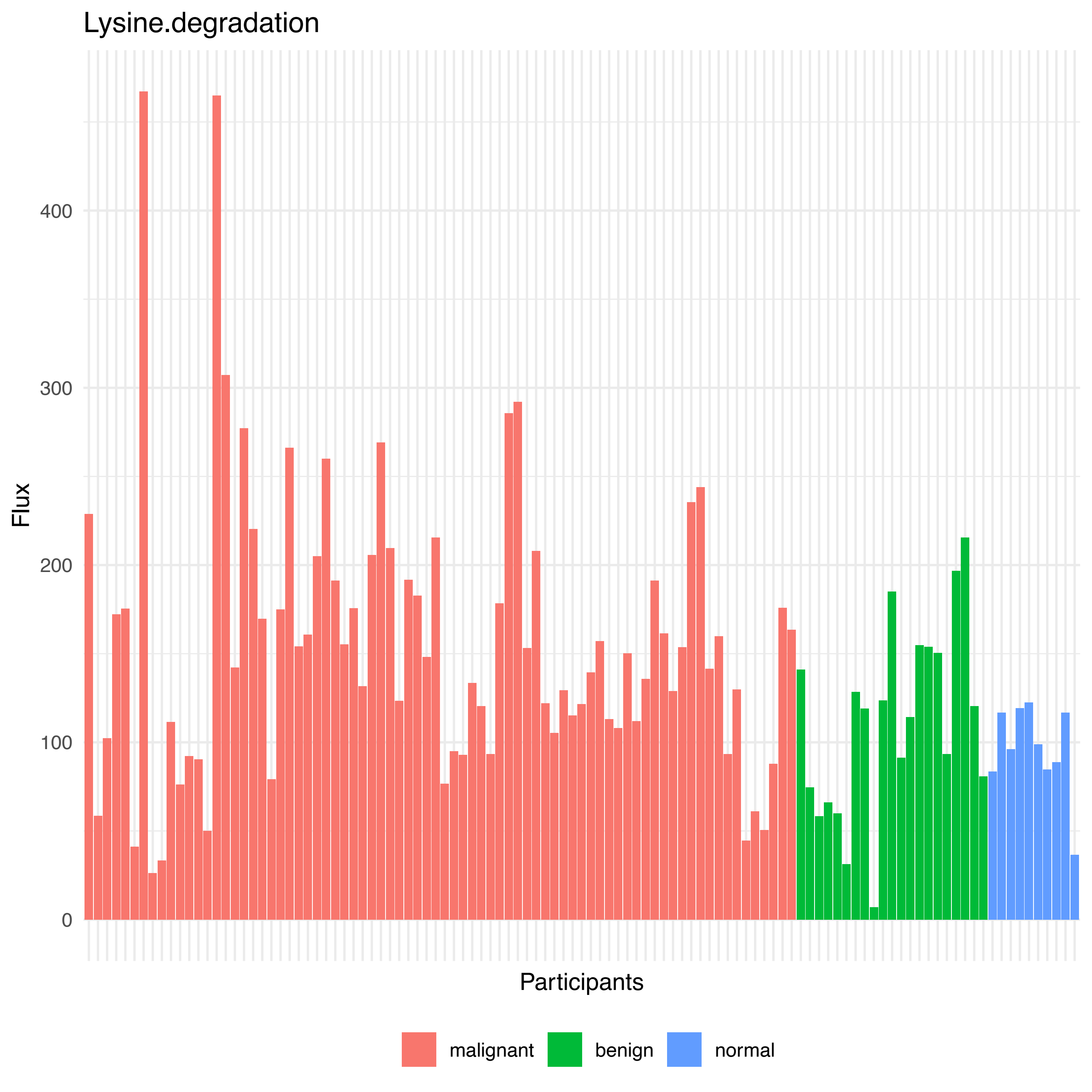

Lysine.degradation
Flux
400
300
200
100
0
Participants
malignant
benign
normal

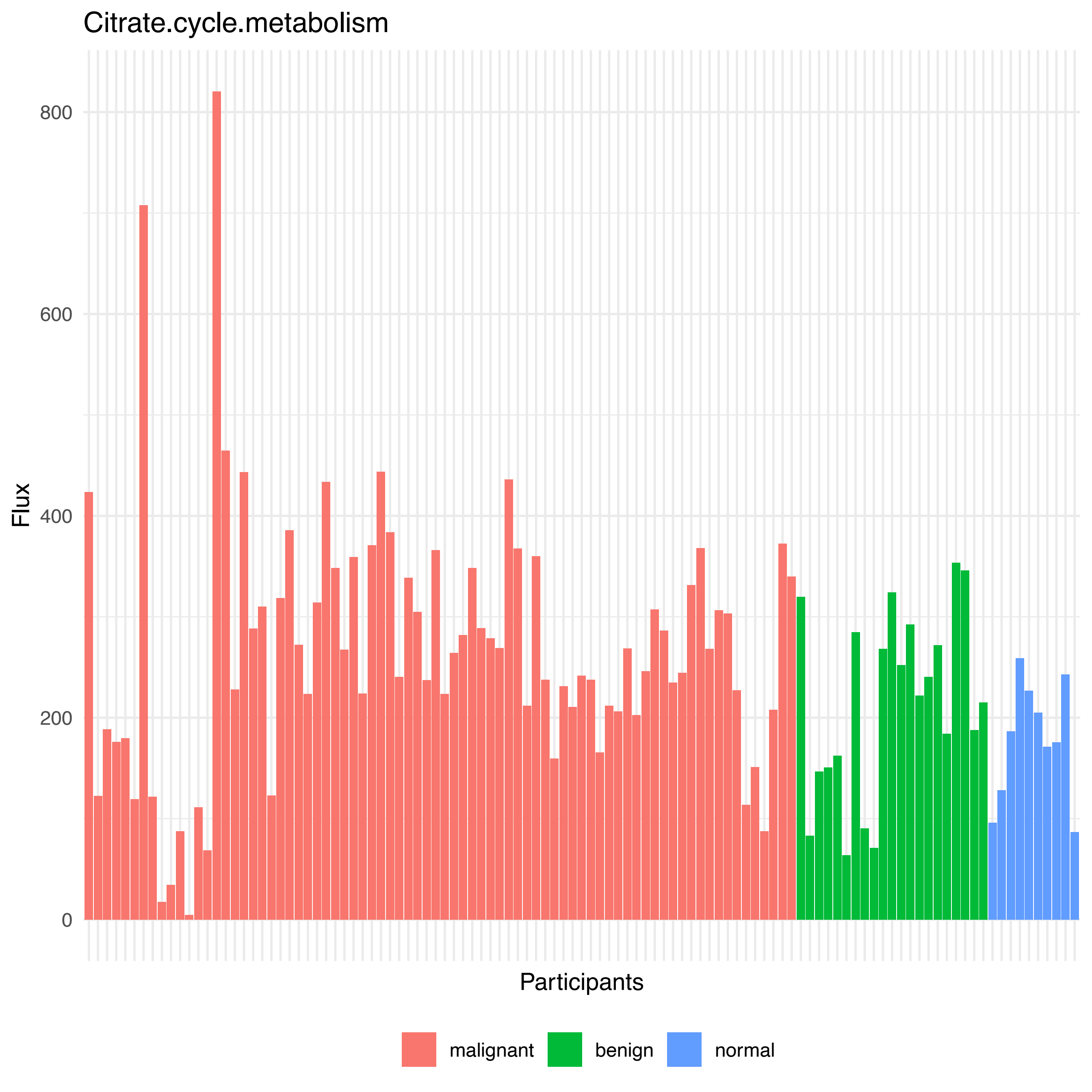

Citrate.cycle.metabolism
Flux
800
600
400
200
0
Participants
malignant
benign
normal

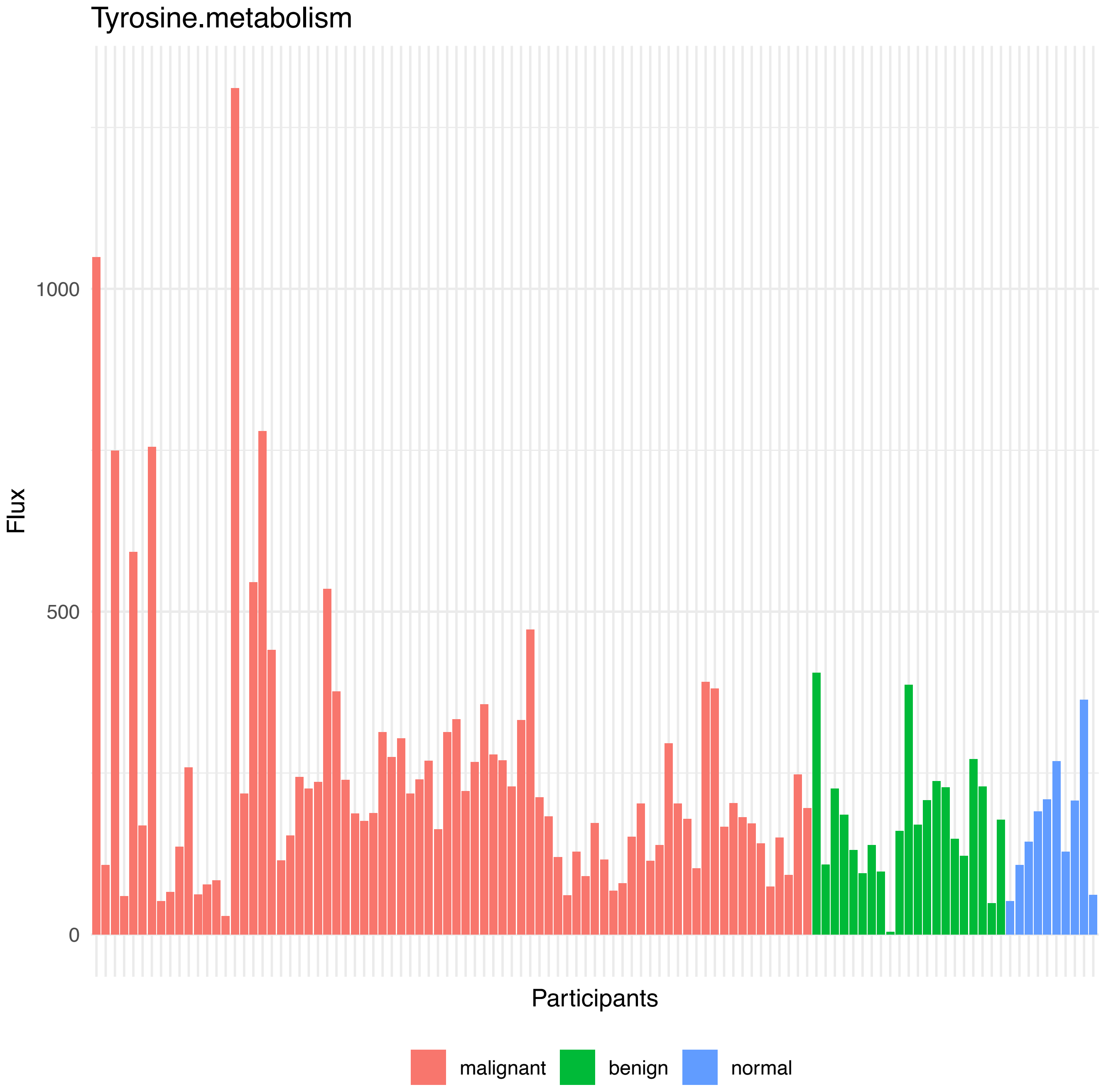

Tyrosine.metabolism
Flux
1000
500
0
Participants
malignant
benign
normal

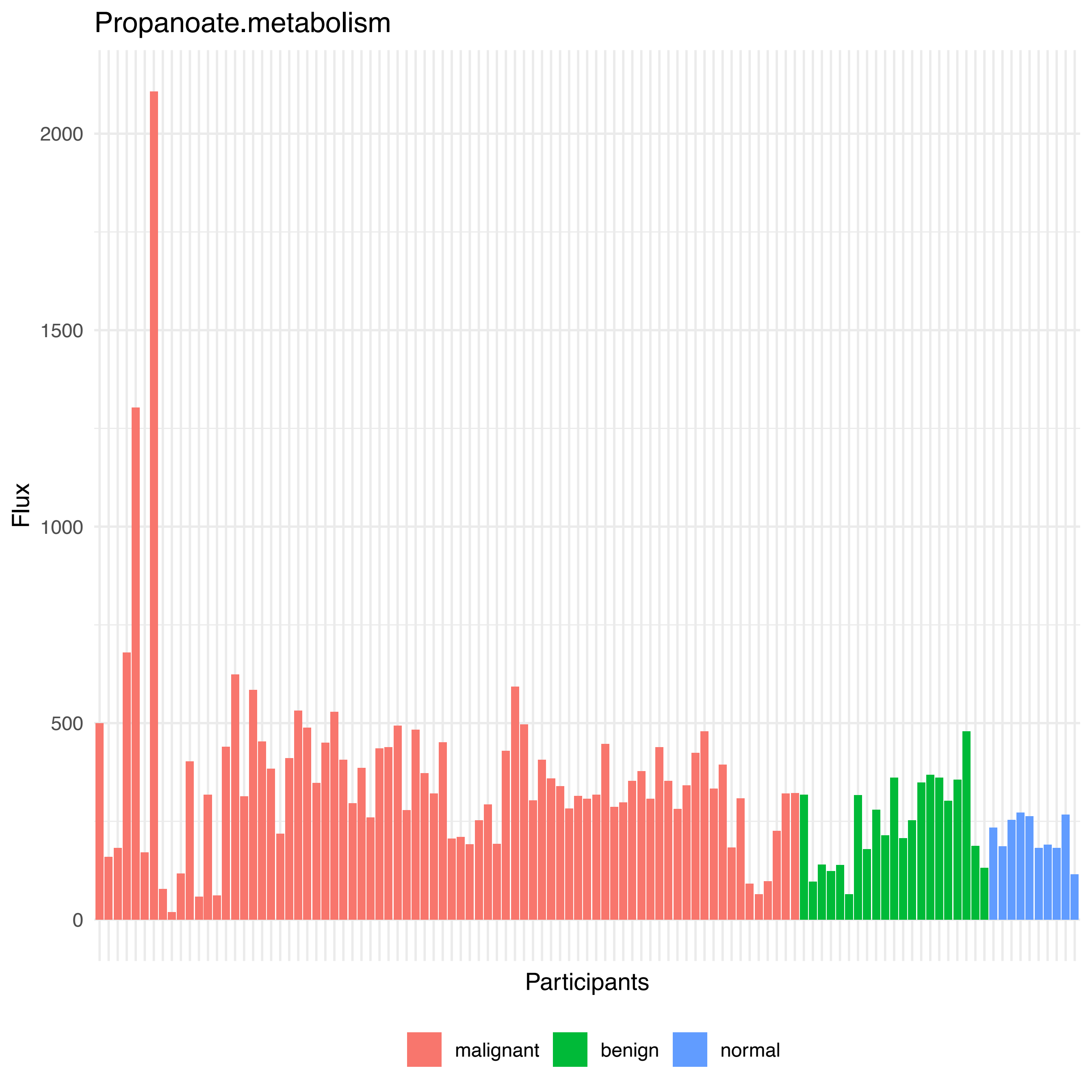
Propanoate.metabolism
Flux
2000
1500
1000
500
0
Participants
malignant
benign
normal

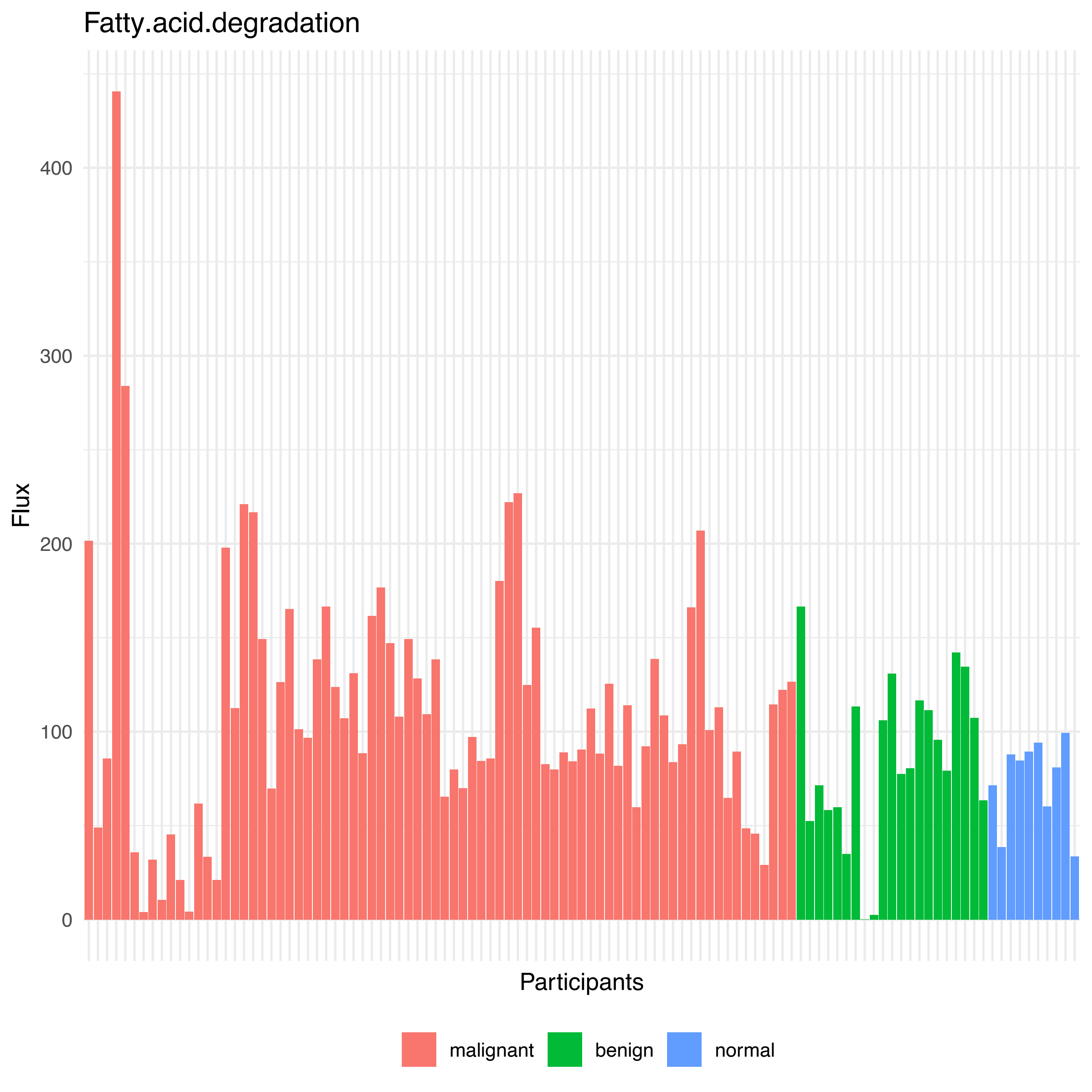

Fatty.acid.degradation
Flux
400
300
200
100
0
Participants
malignant
benign
normal

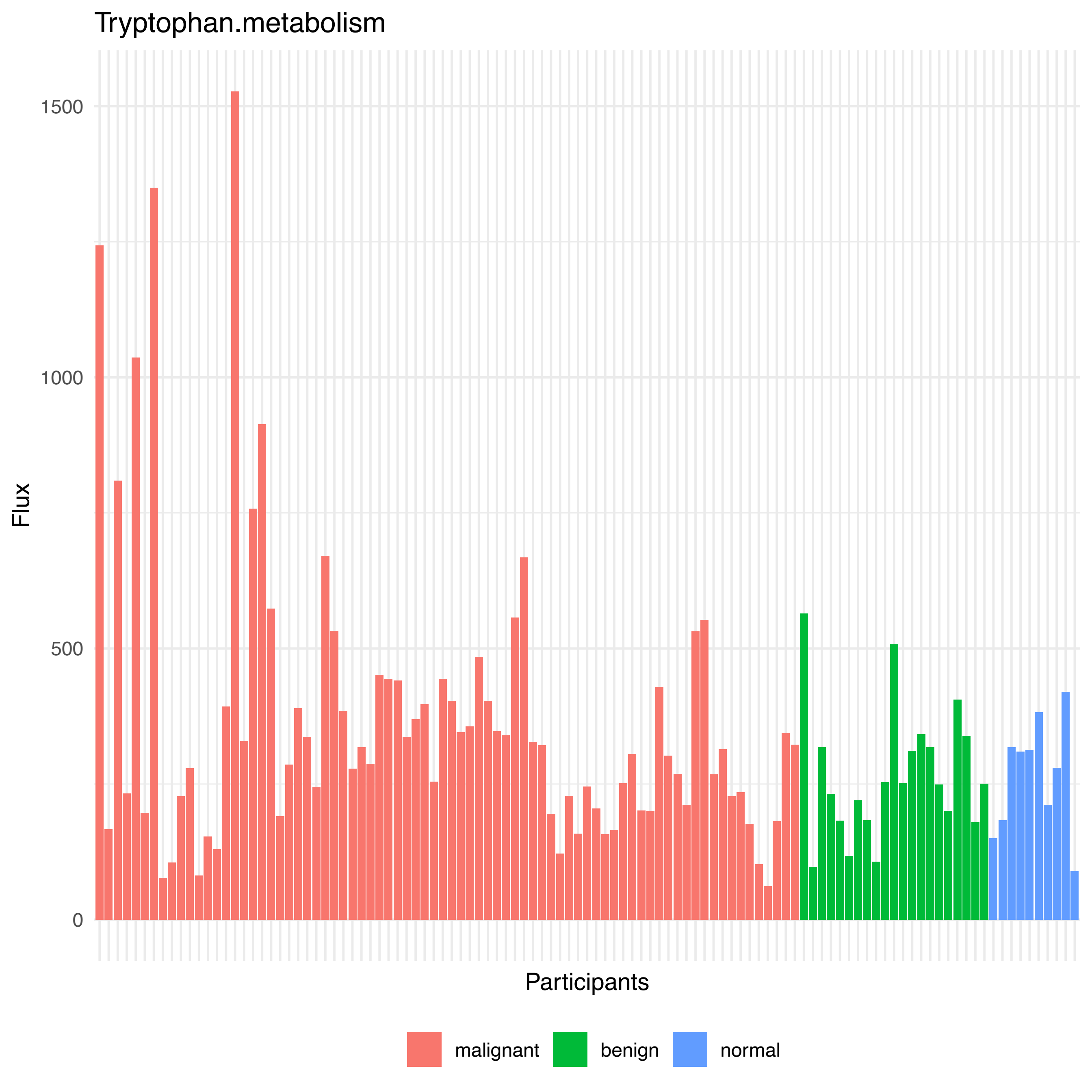

Tryptophan.metabolism
Flux
1500
1000
500
0
Participants
malignant
benign
normal

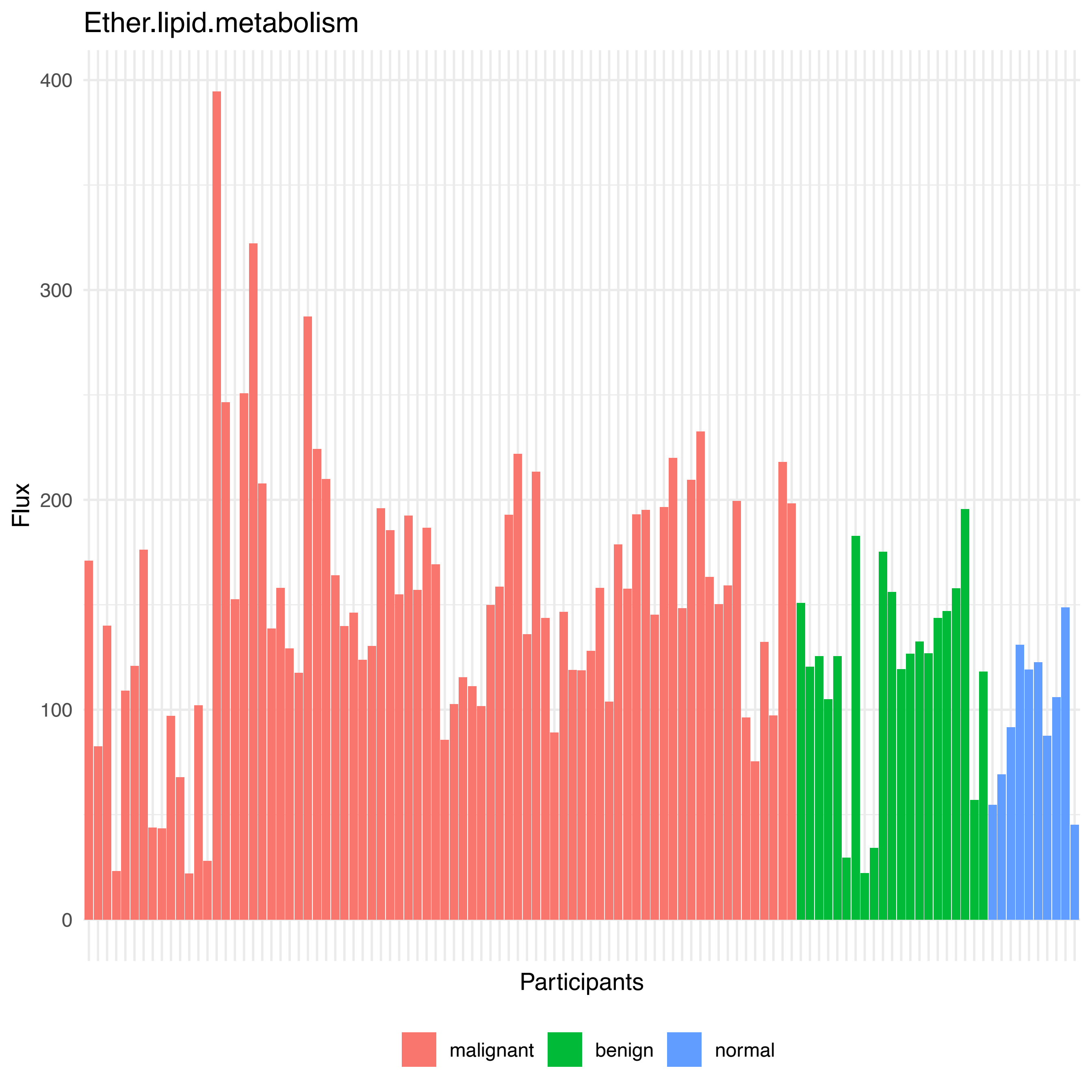

Ether.lipid.metabolism
Flux
400
300
200
100
0
Participants
malignant
benign
normal

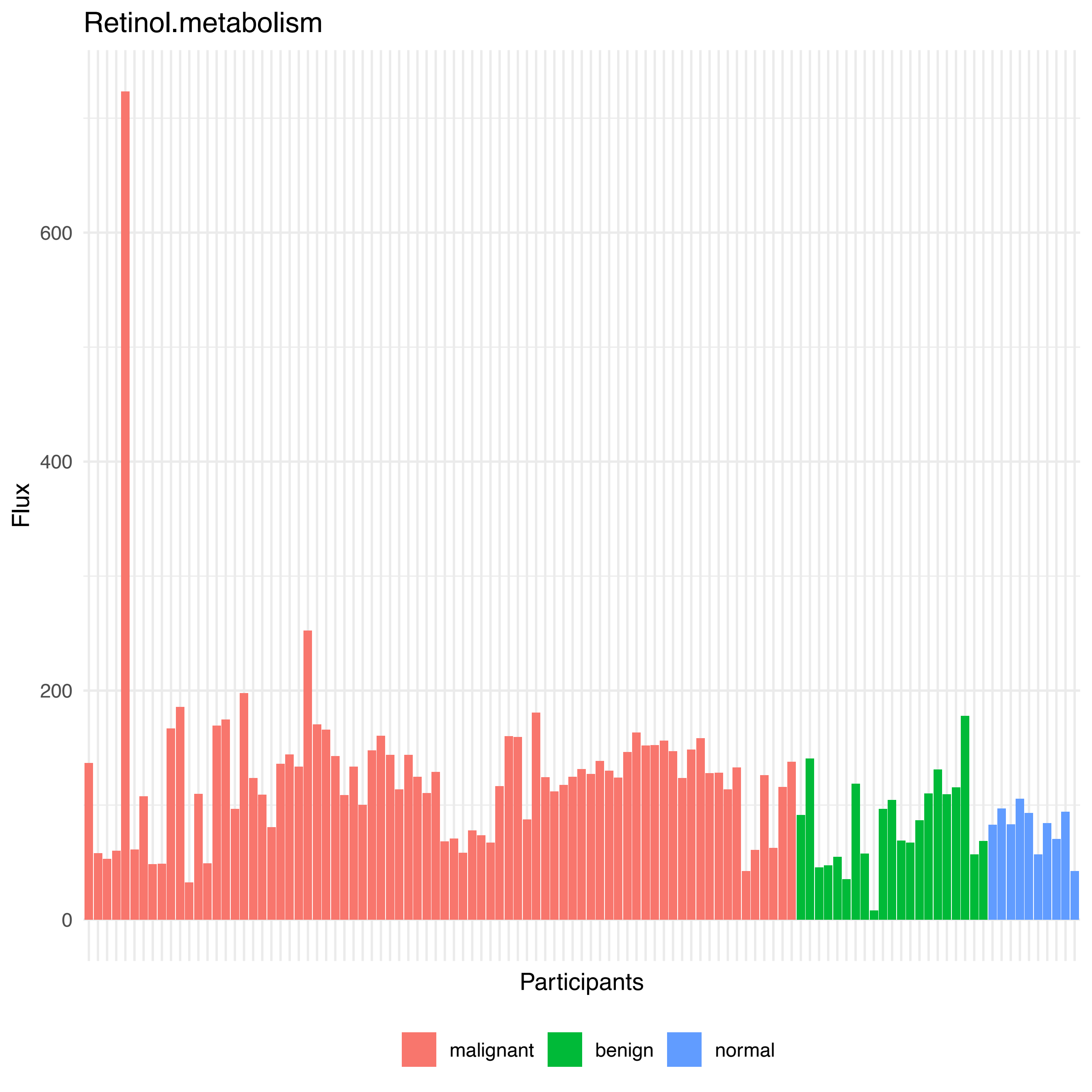

Retinol.metabolism
Flux
600
400
200
0
Participants
malignant
benign
normal

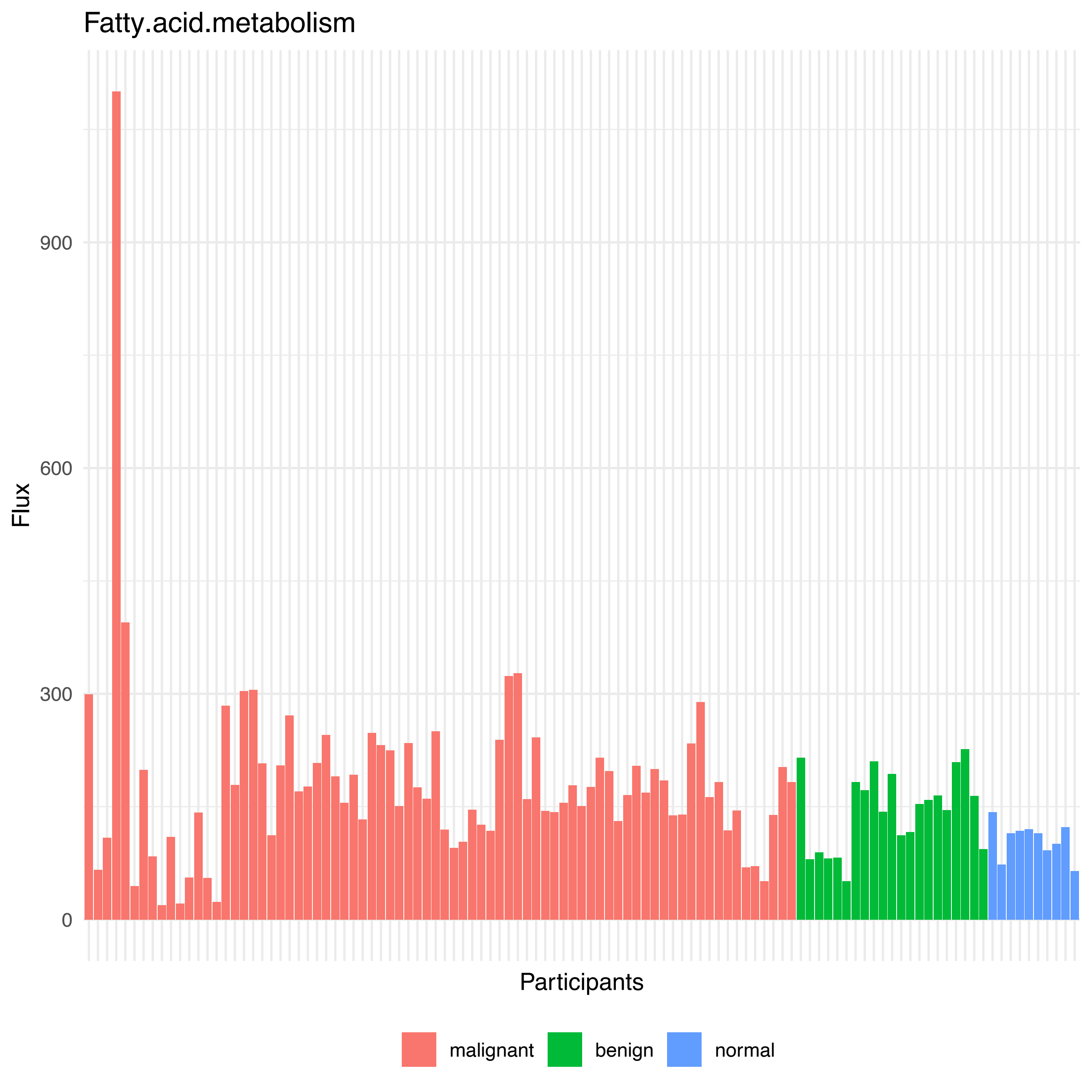

Fatty.acid.metabolism
Flux
900
600
300
0
Participants
malignant
benign
normal

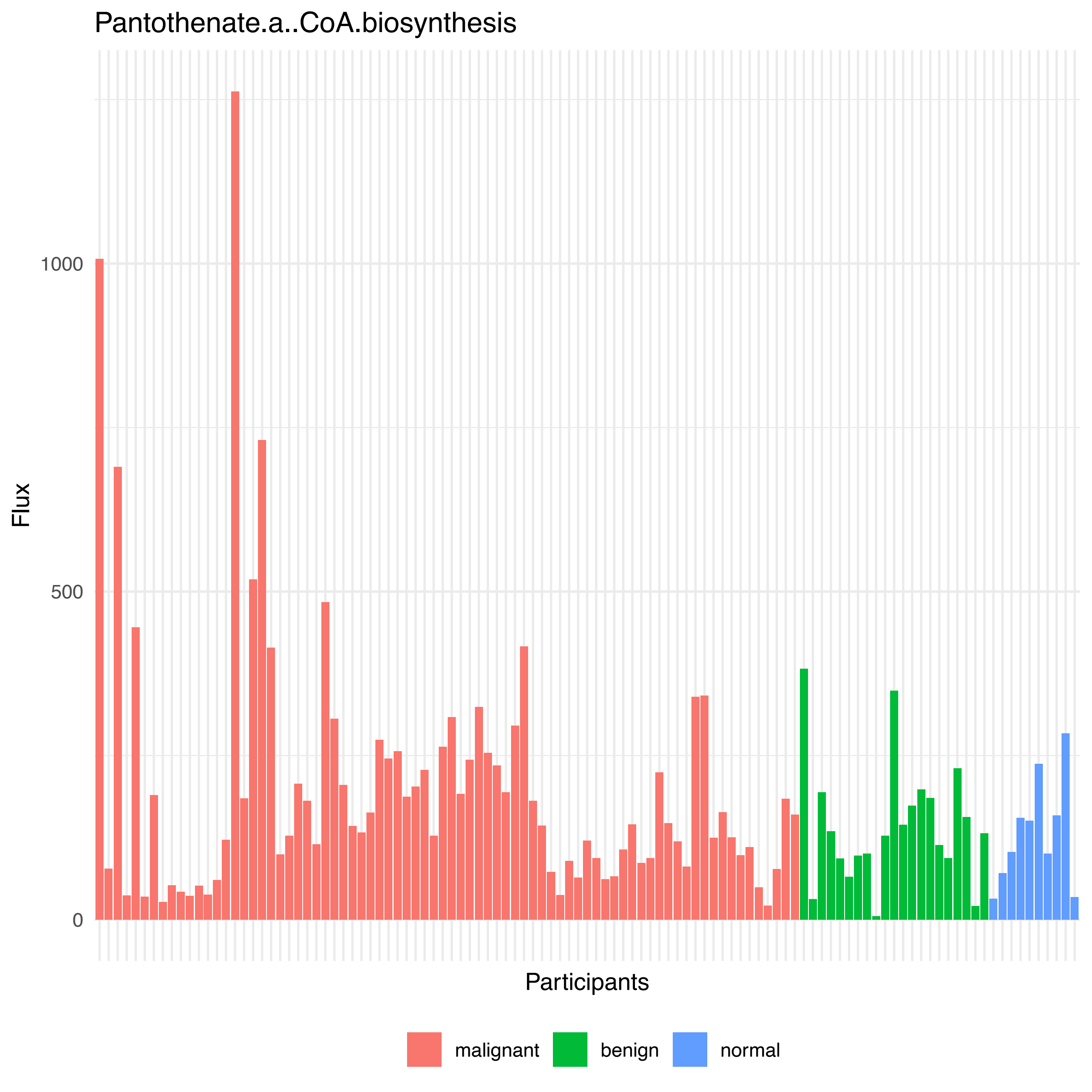
Pantothenate.a..CoA.biosynthesis
Flux
1000
500
0
Participants
malignant
benign
normal

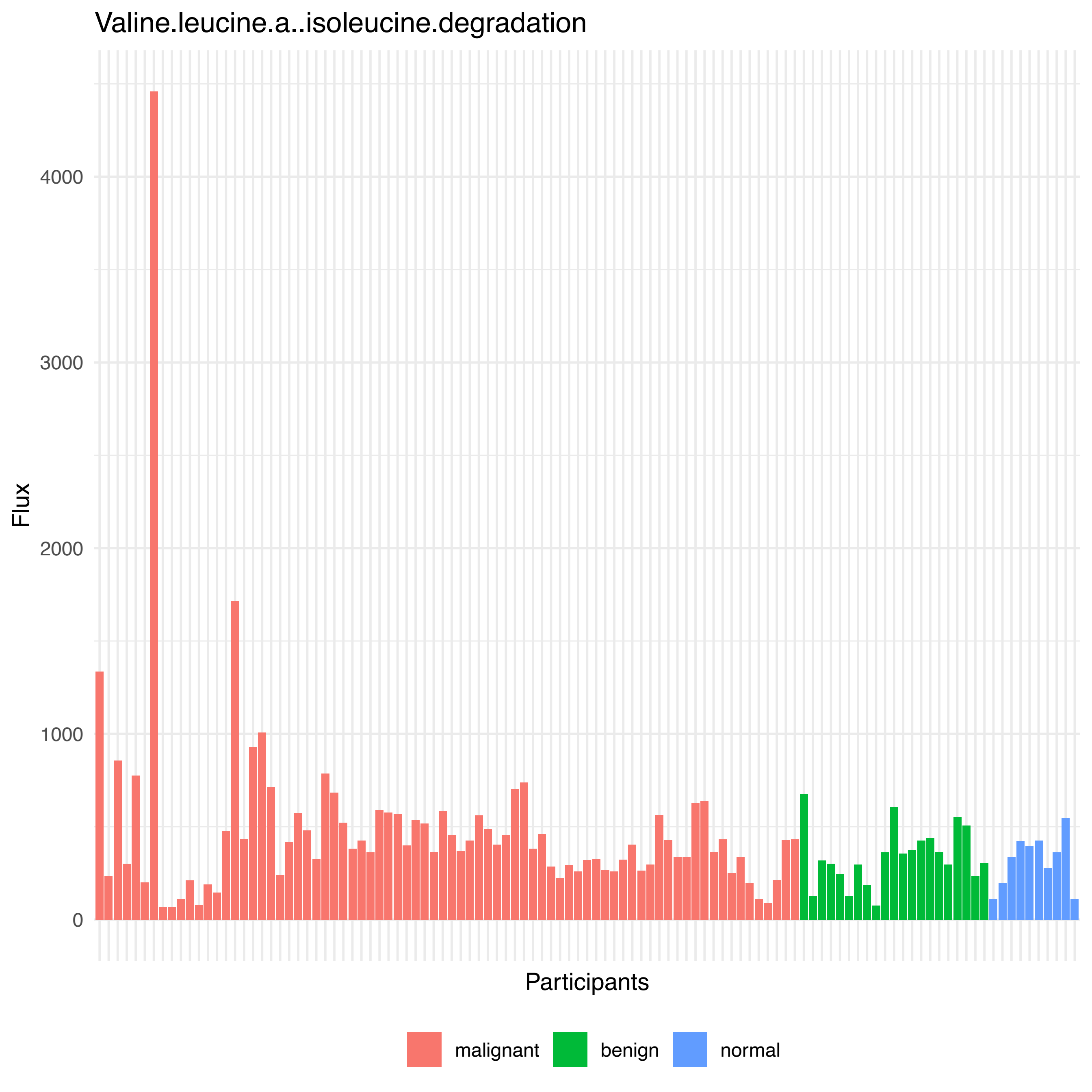
Valine.leucine.a..isoleucine.degradation
Flux
4000
3000
2000
1000
0
Participants
malignant
benign
normal

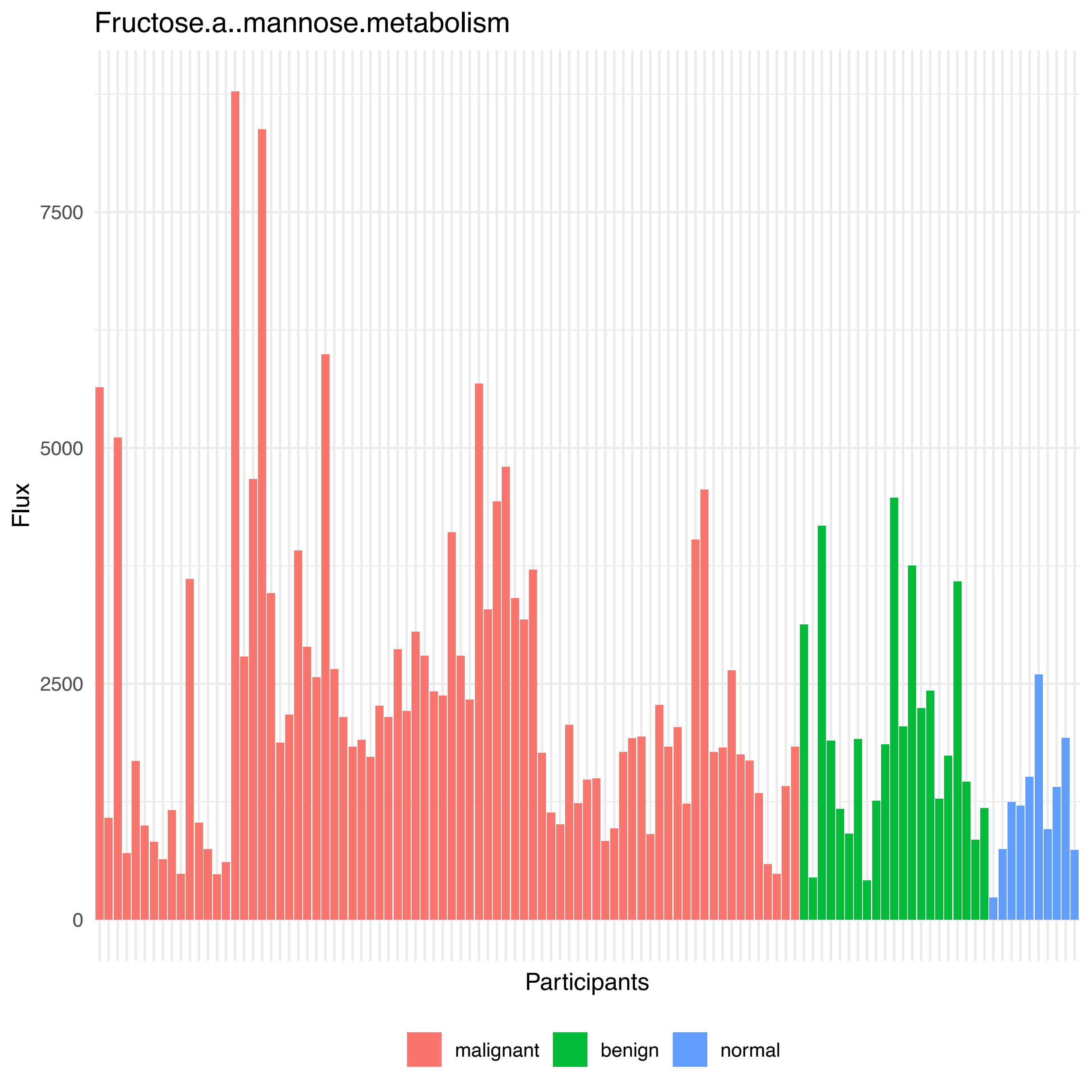

Fructose.a..mannose.metabolism
Flux
7500
5000
2500
0
Participants
malignant
benign
normal

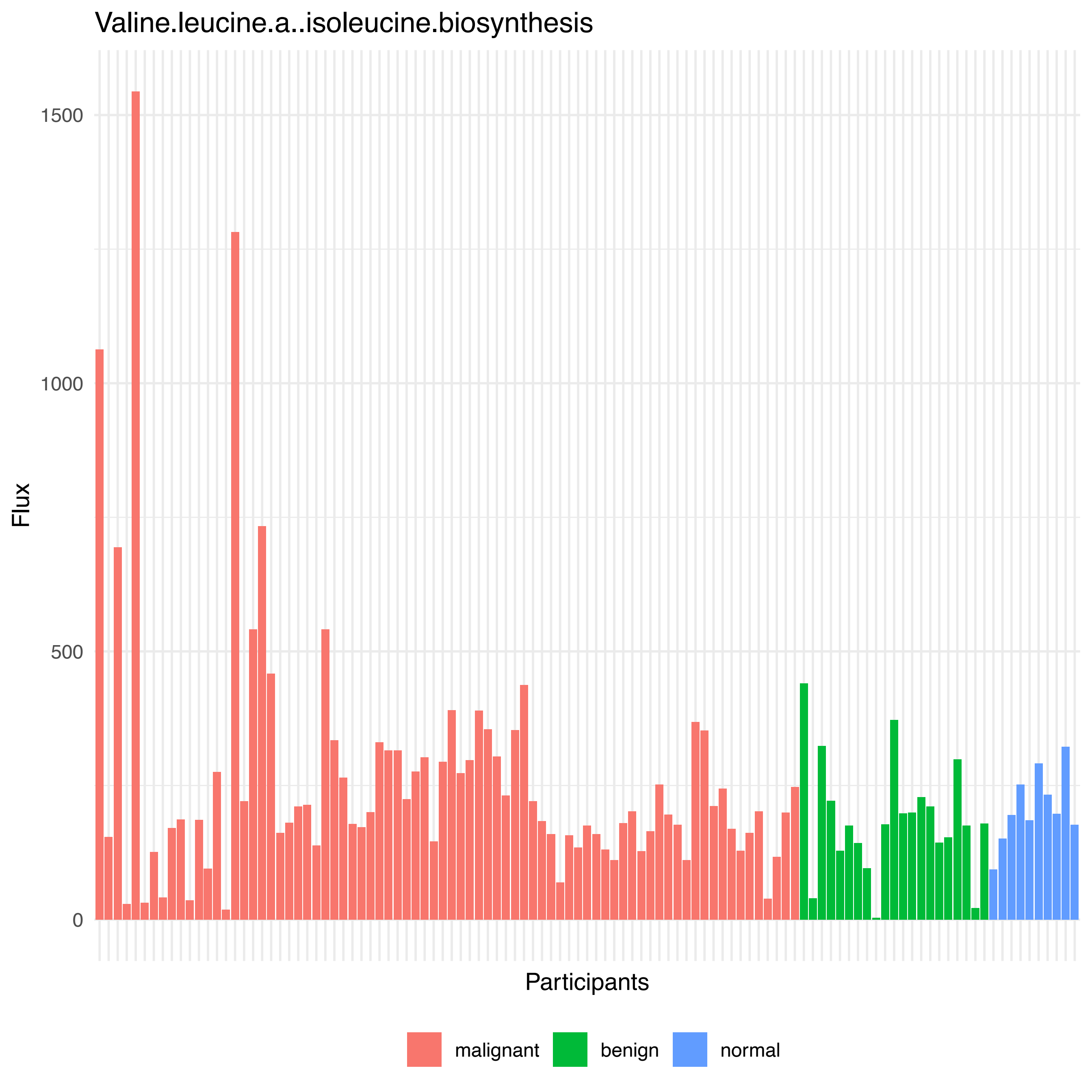

Valine.leucine.a..isoleucine.biosynthesis
1500
1000
500
0
Flux
Participants
malignant
benign
normal

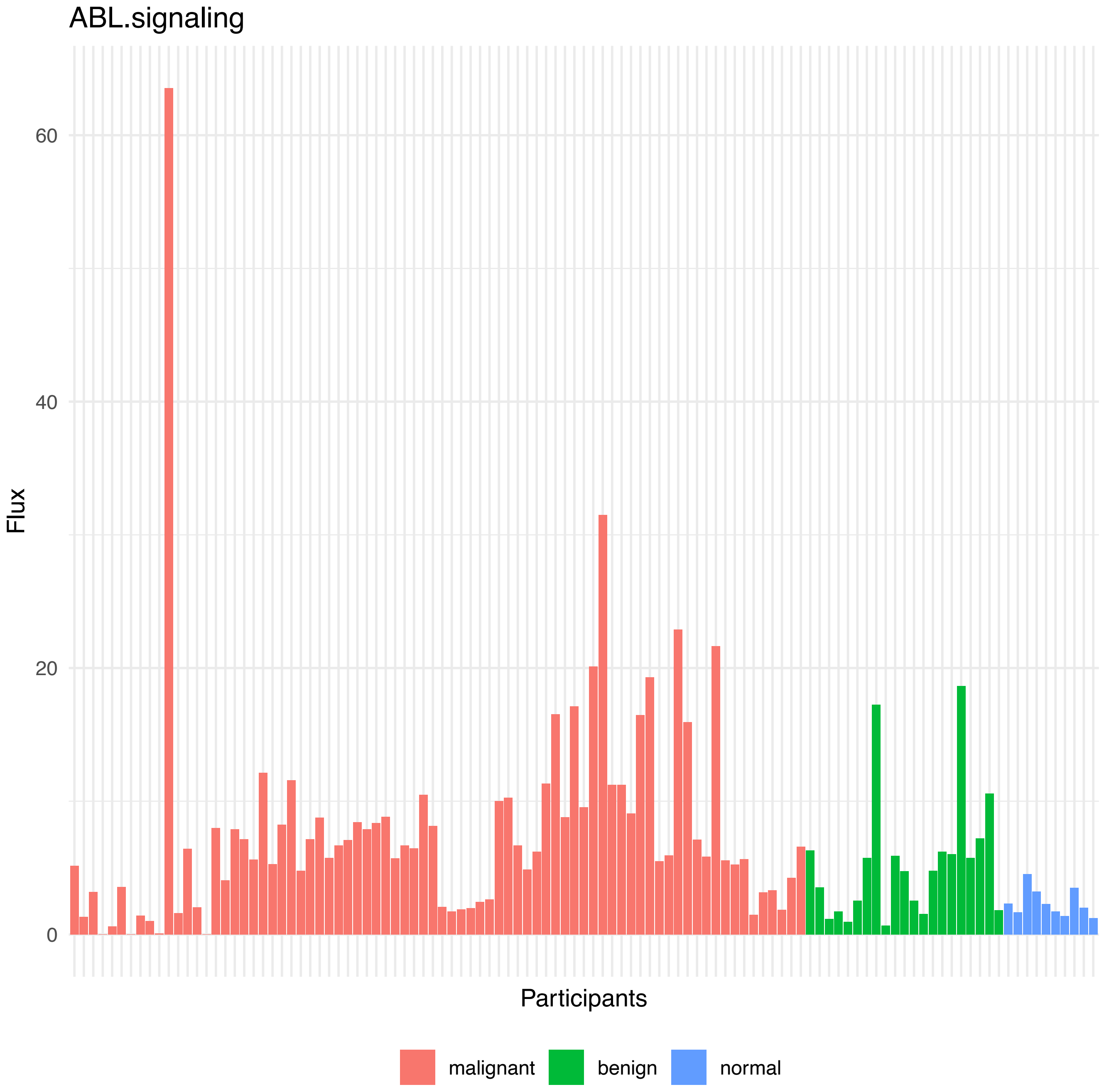
ABL.signaling
60
40
20
0
Flux
Participants
malignant
benign
normal

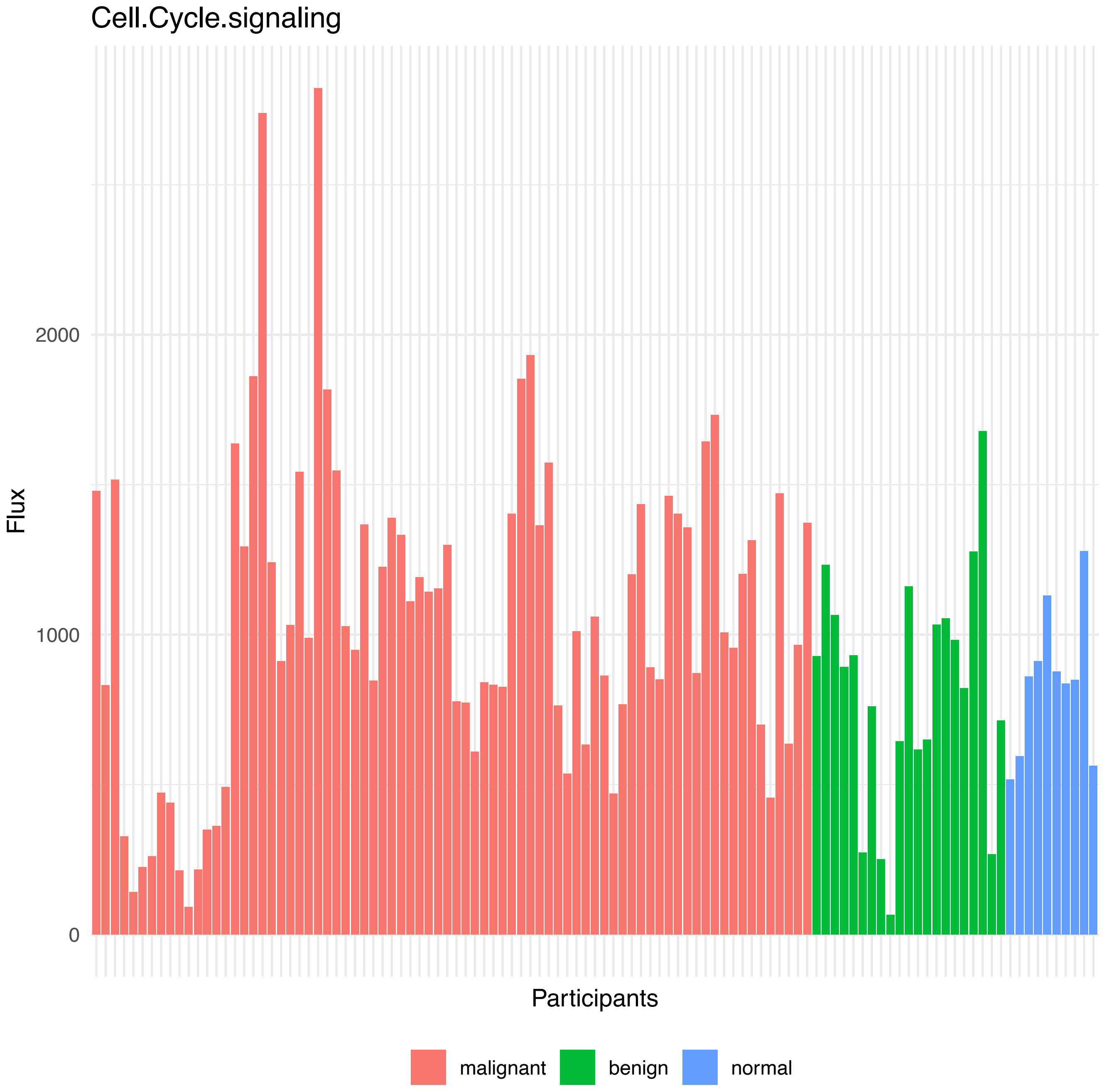
Cell.Cycle.signaling
Flux
2000
1000
0
Participants
malignant
benign
normal

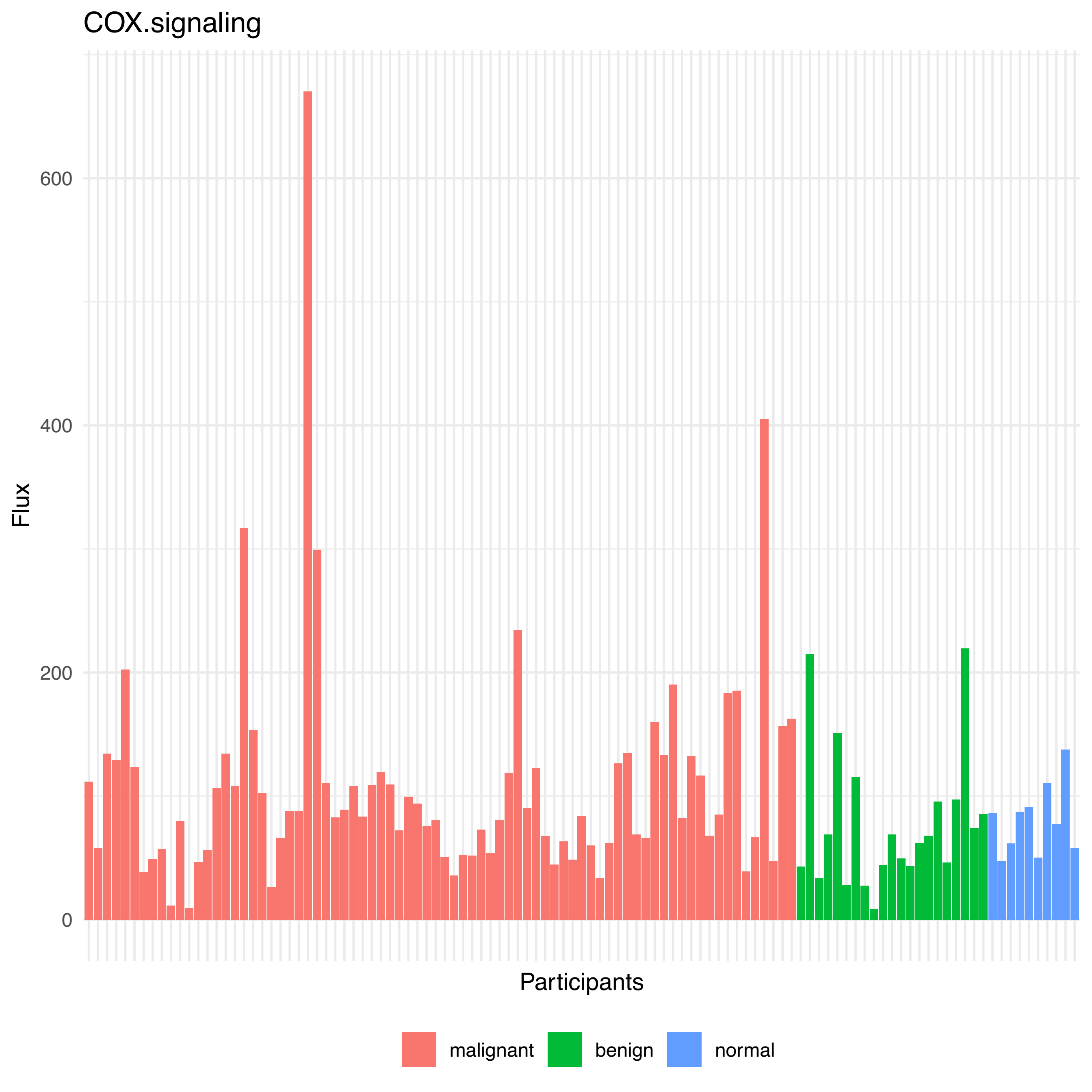
COX.signaling
600
400
200
0
Flux
Participants
malignant
benign
normal

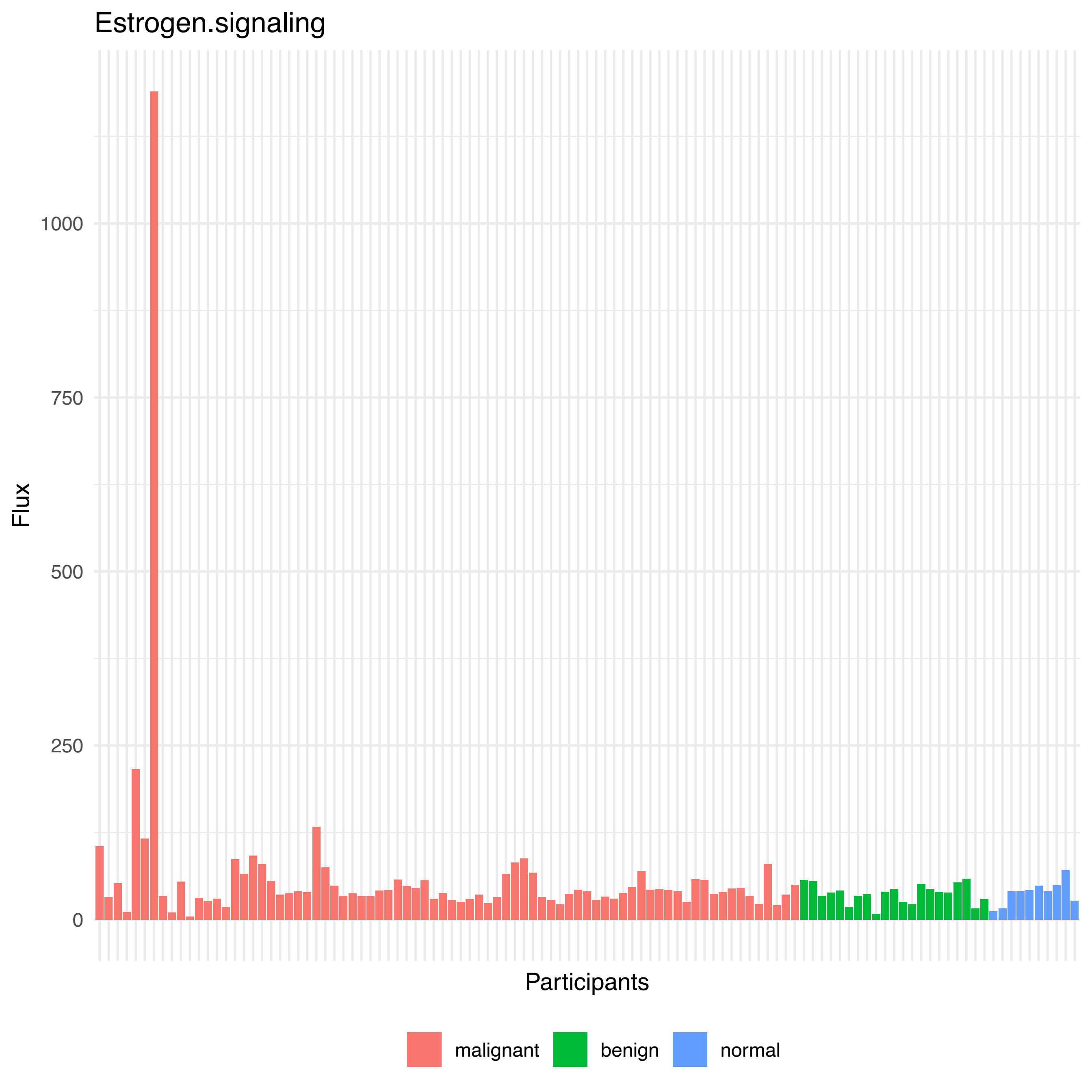

Estrogen.signaling
Flux
1000
750
500
250
0
Participants
malignant
benign
normal

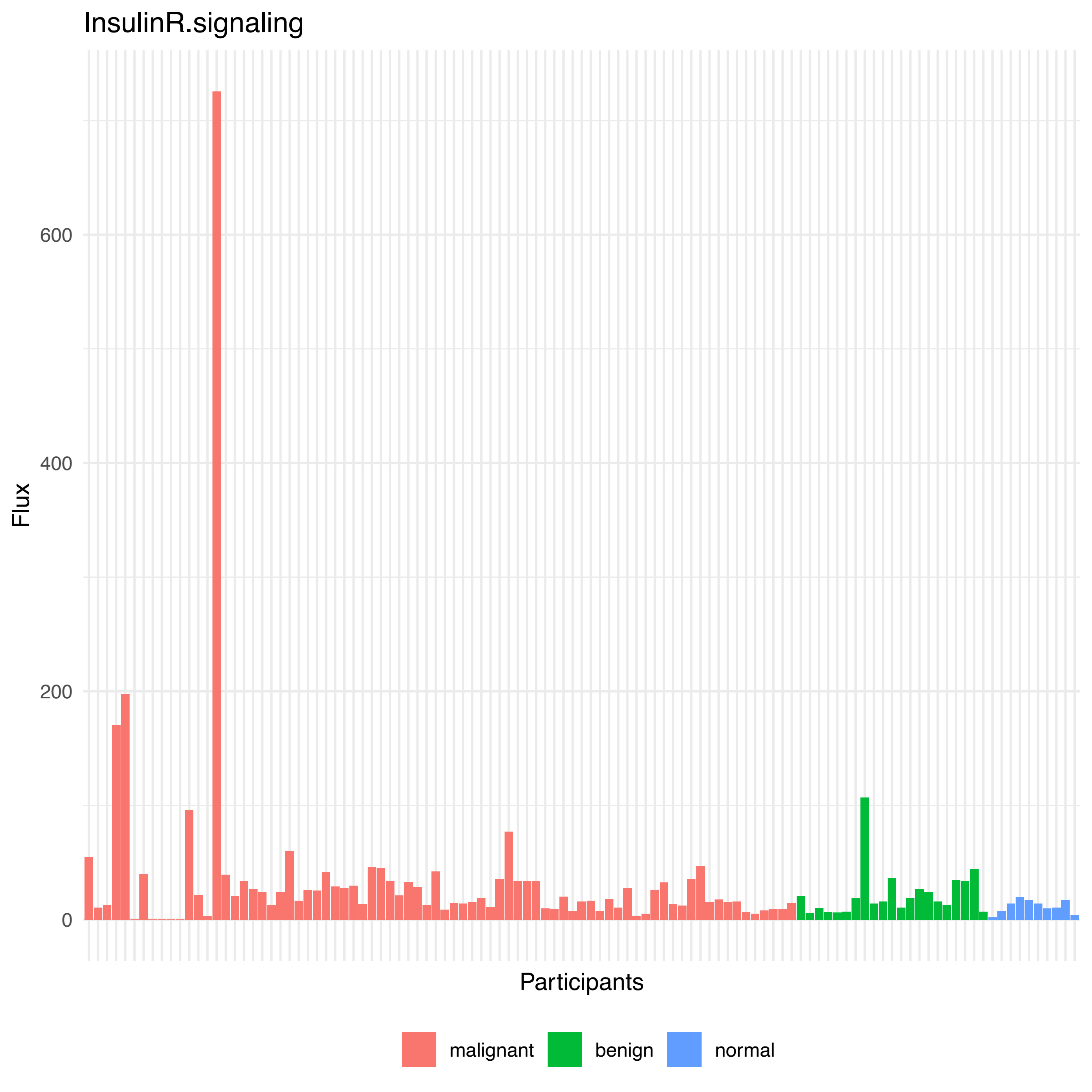

InsulinR.signaling
Flux
600
400
200
0
Participants
malignant
benign
normal

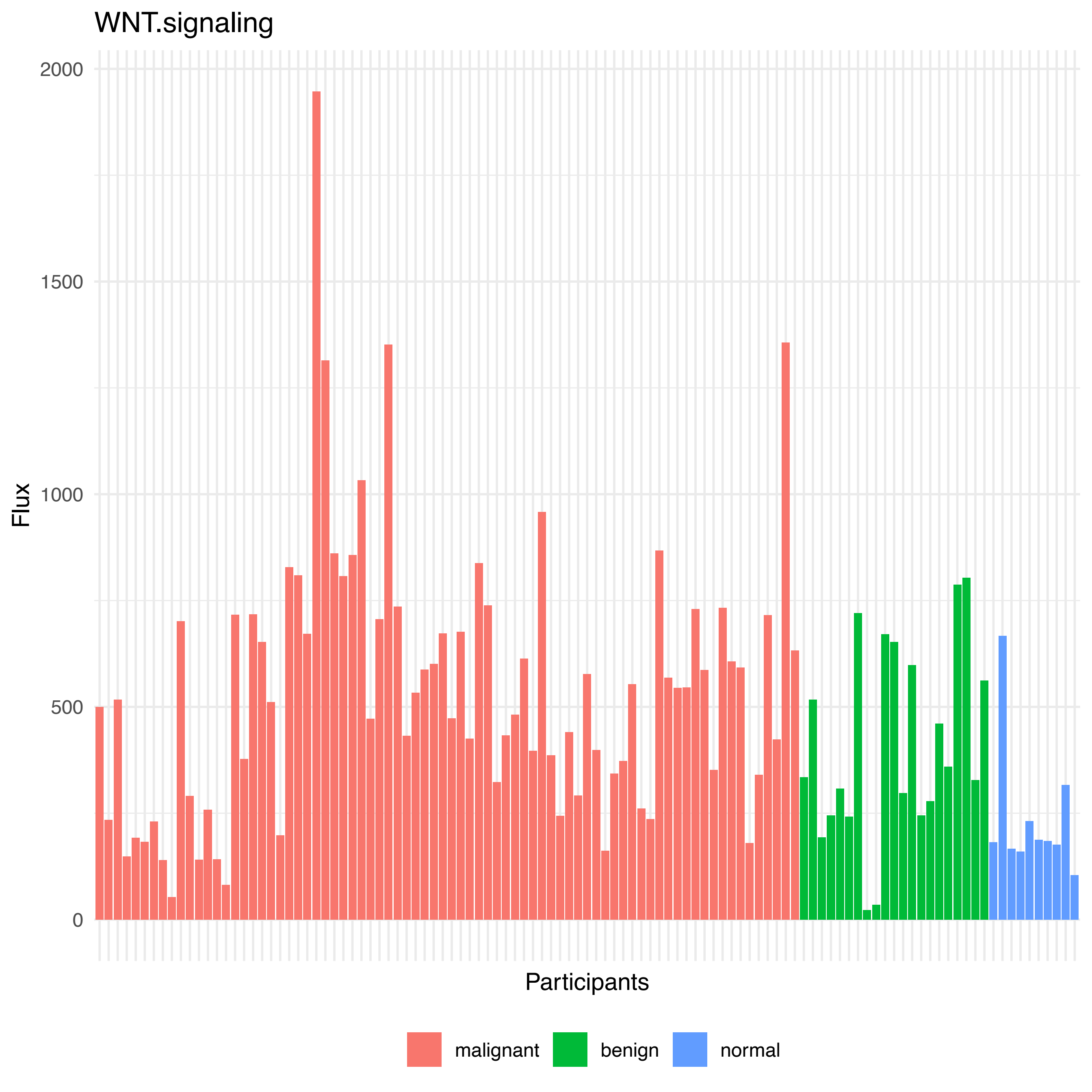

WNT.signaling
2000
1500
1000
500
0
Flux
Participants
malignant
benign
normal

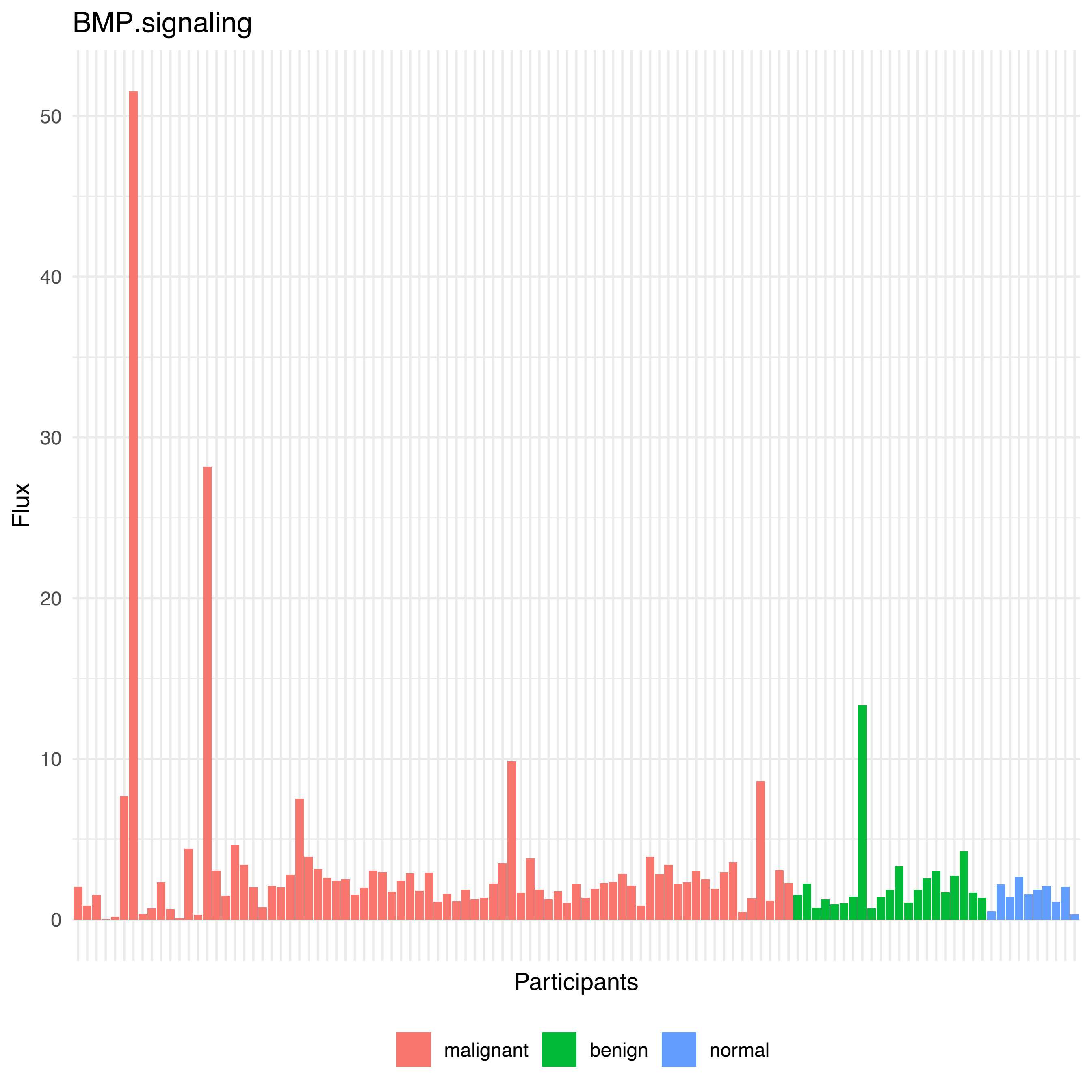

BMP.signaling
Flux
50
40
30
20
10
0
Participants
malignant
benign
normal

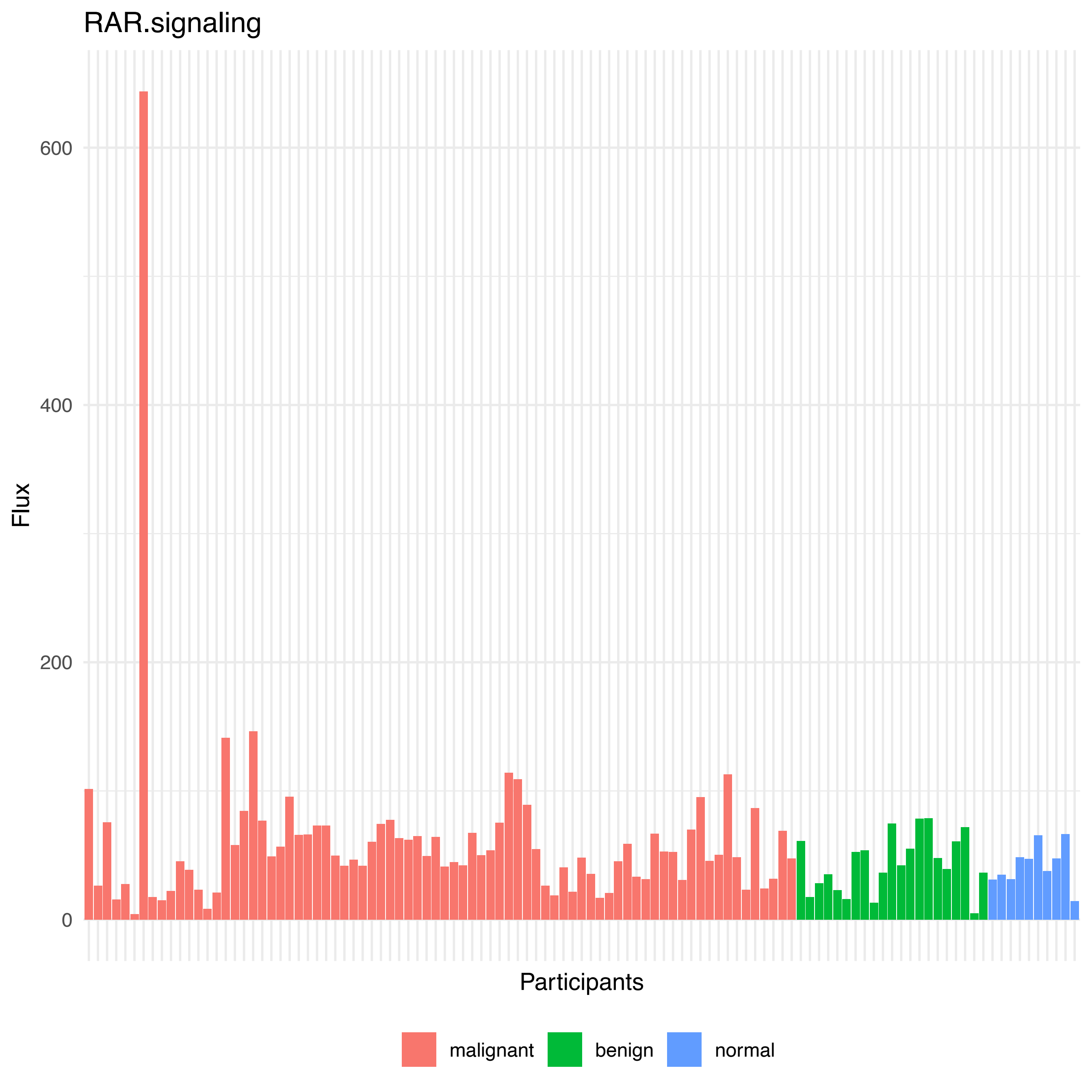
RAR.signaling
600
400
200
0
Flux
Participants
malignant
benign
normal

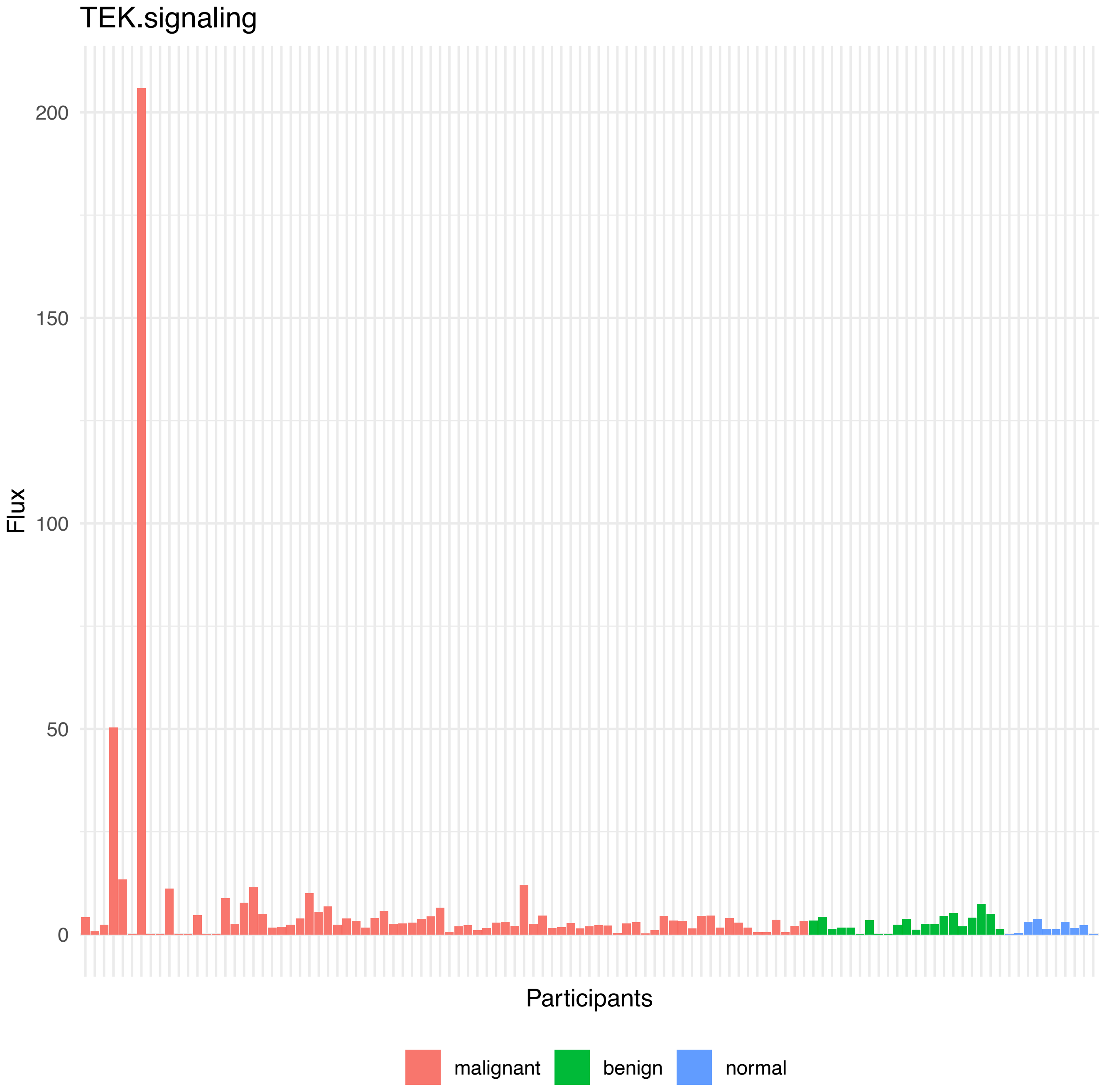
TEK.signaling
Flux
200
150
100
50
0
Participants
malignant
benign
normal

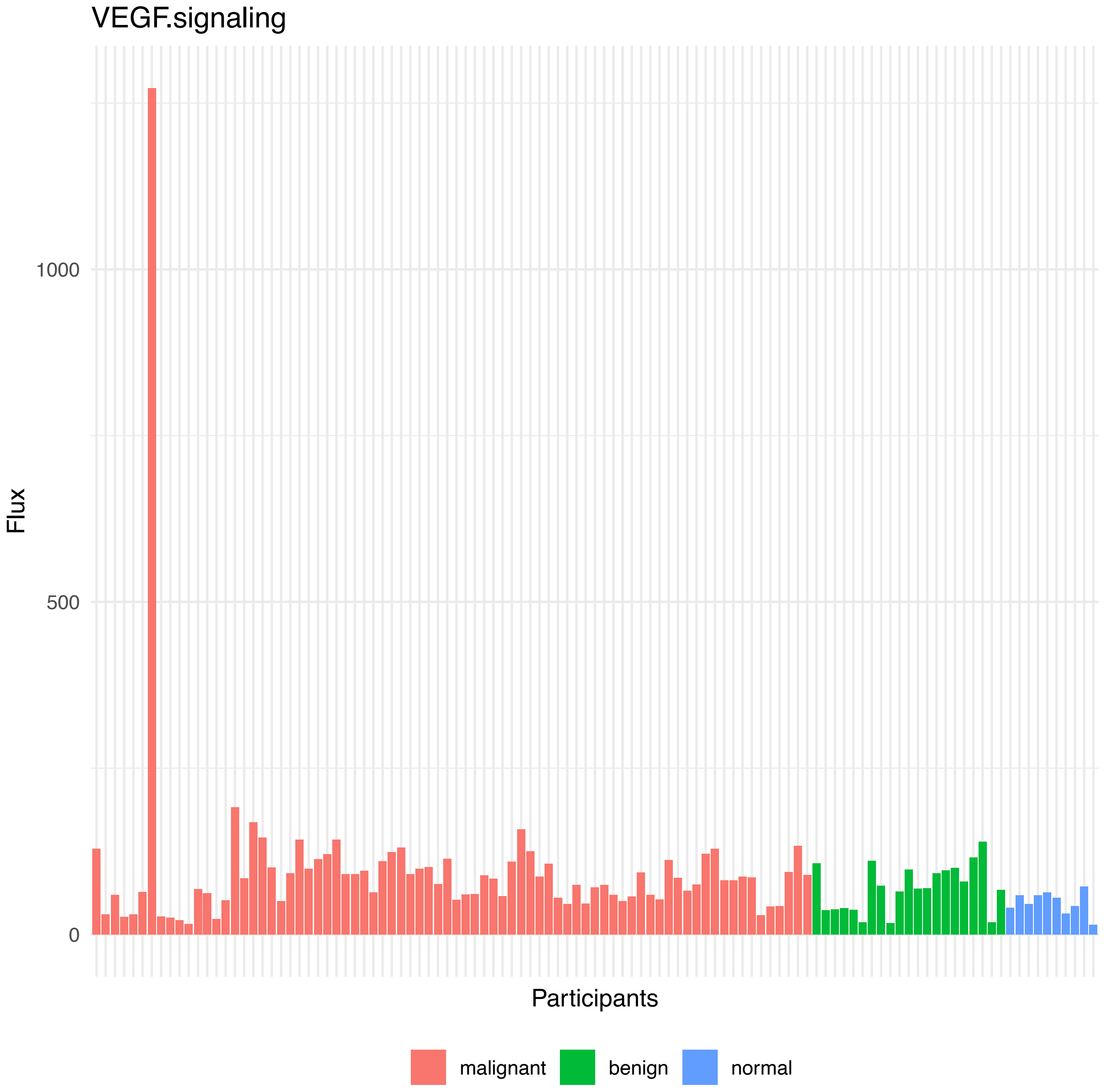

VEGF.signaling
Flux
1000
500
0
Participants
malignant
benign
normal

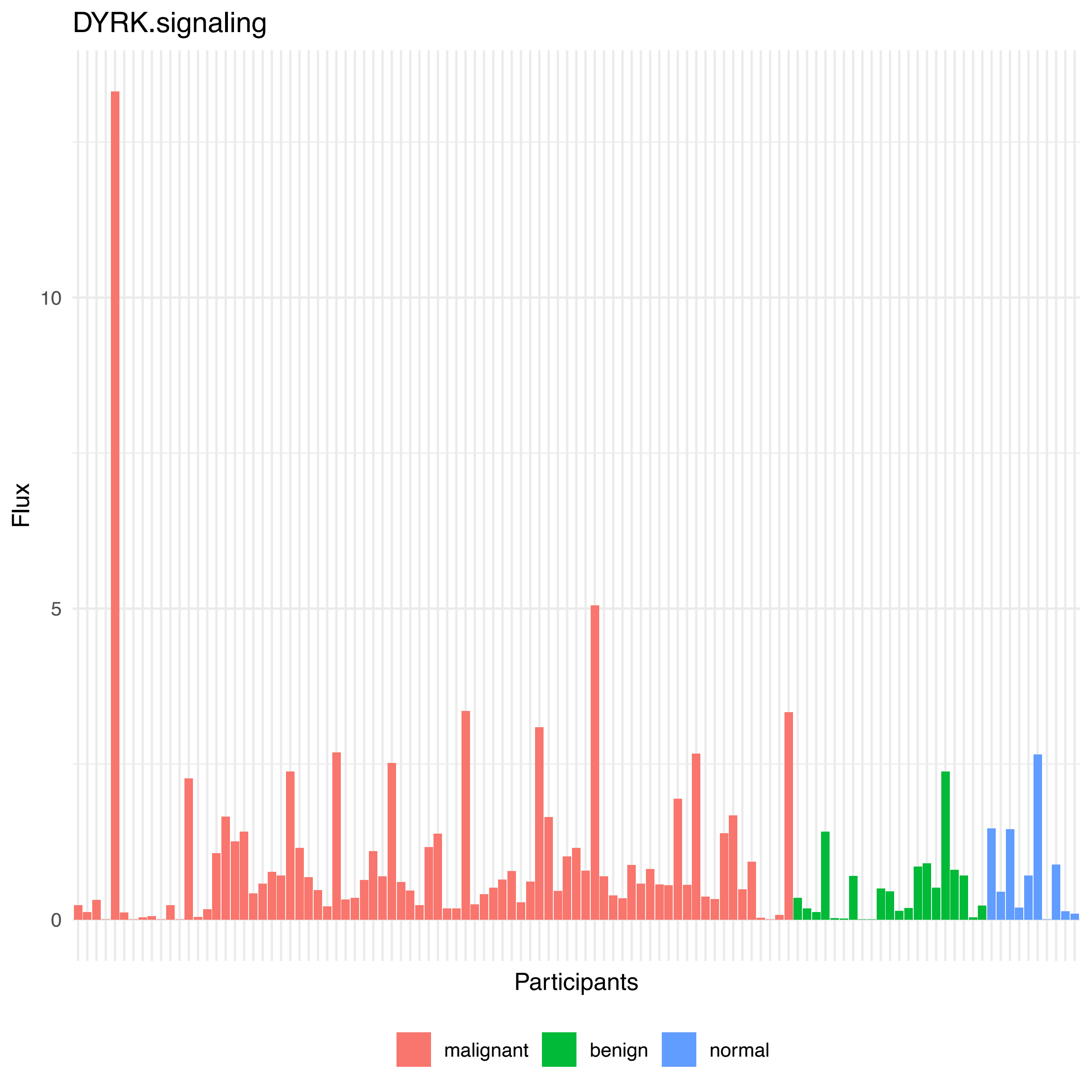
DYRK.signaling
Flux
10
5
0
Participants
malignant
benign
normal

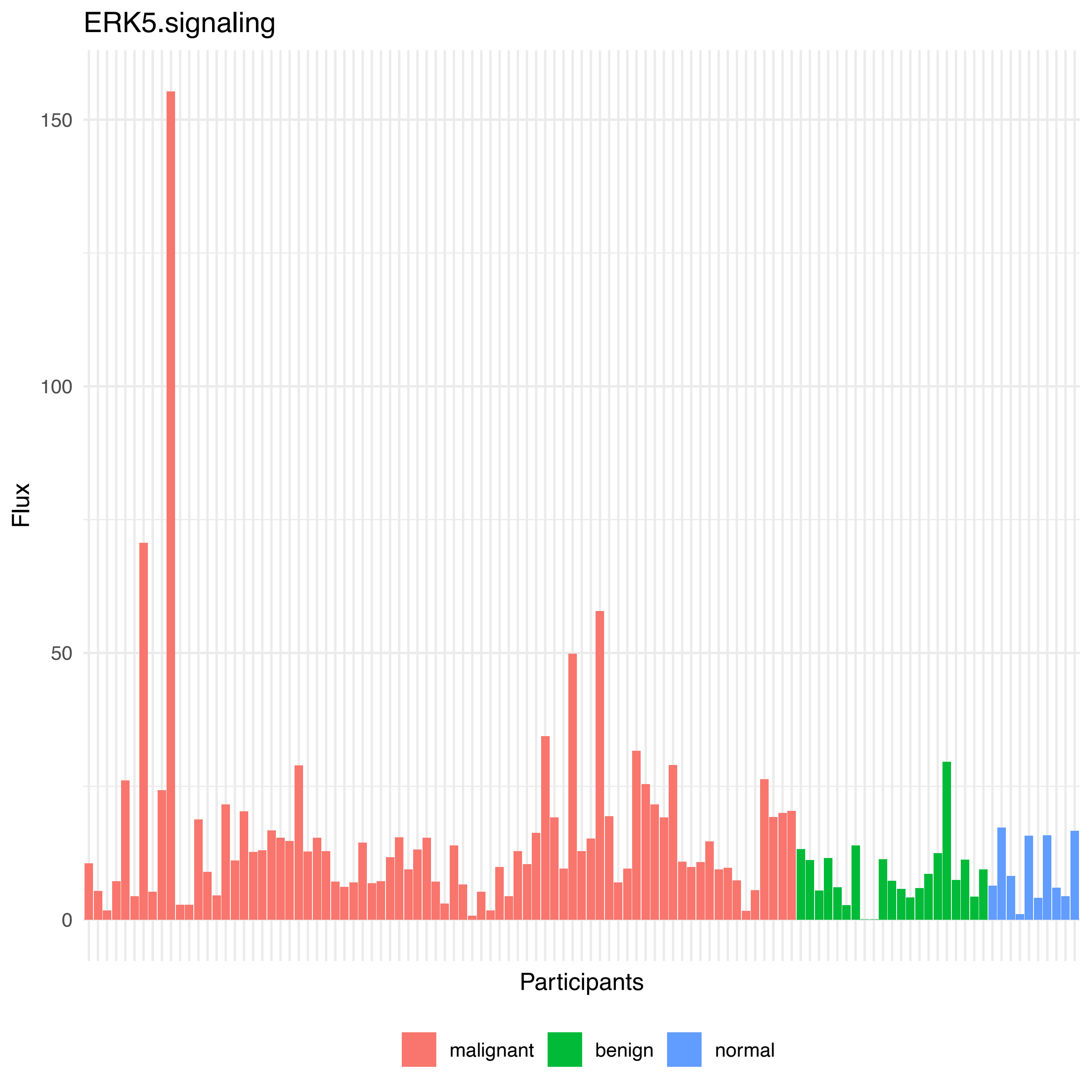

ERK5.signaling
Flux
150
100
50
0
Participants
malignant
benign
normal

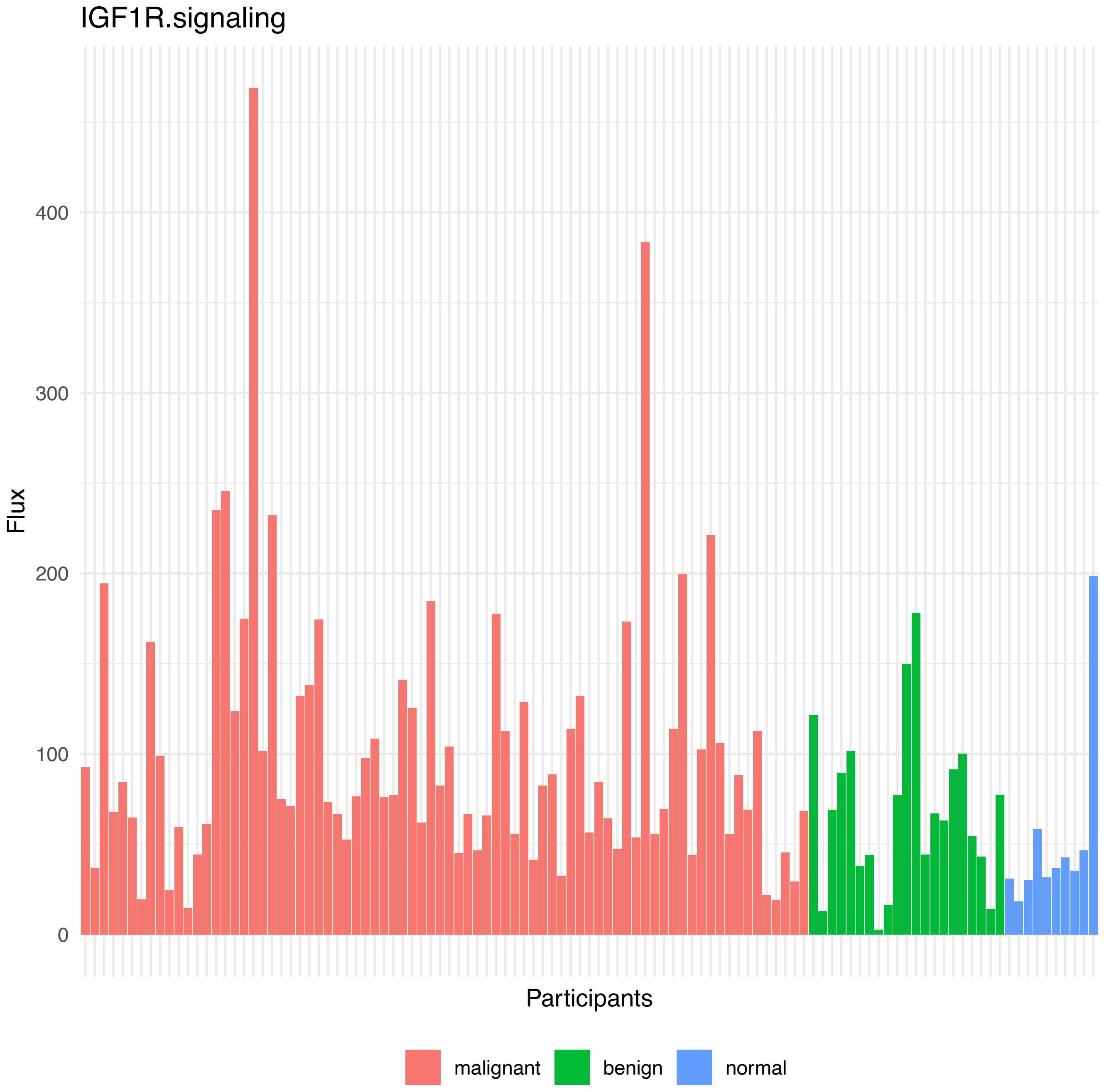
IGF1R.signaling
Flux
400
300
200
100
0
Participants
malignant
benign
normal

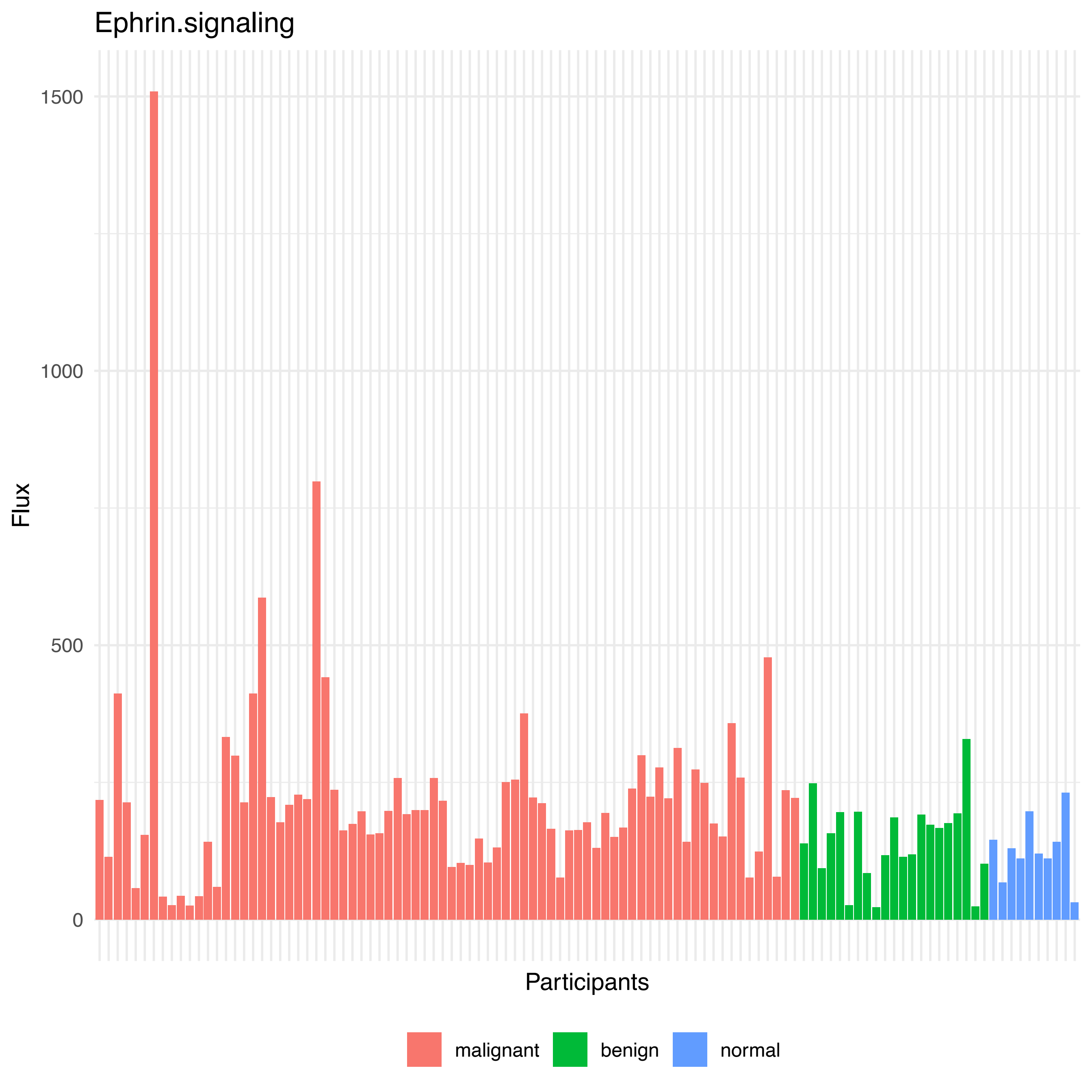
Ephrin.signaling
Flux
1500
1000
500
0
Participants
malignant
benign
normal

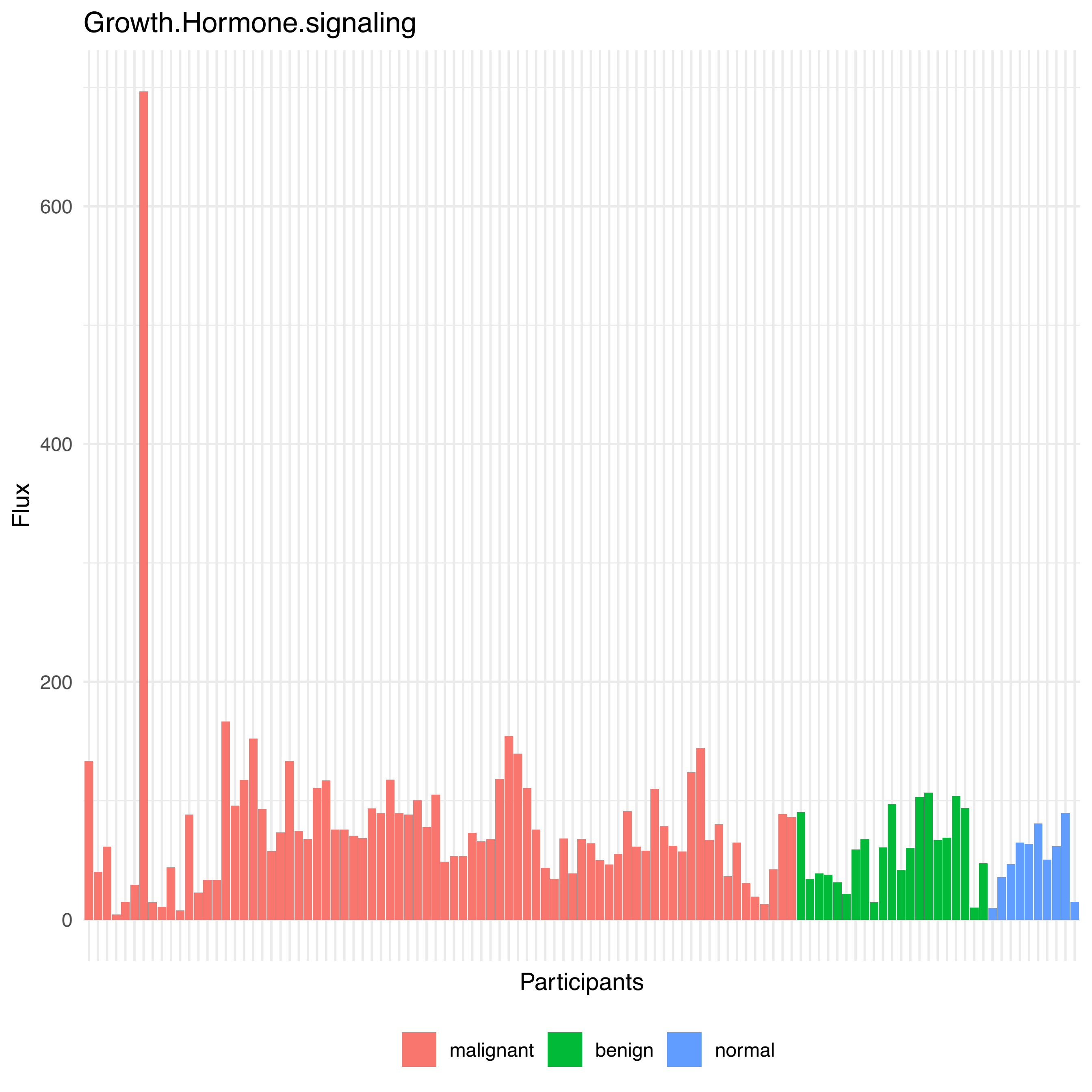
Growth.Hormone.signaling
600
400
200
0
Flux
Participants
malignant
benign
normal

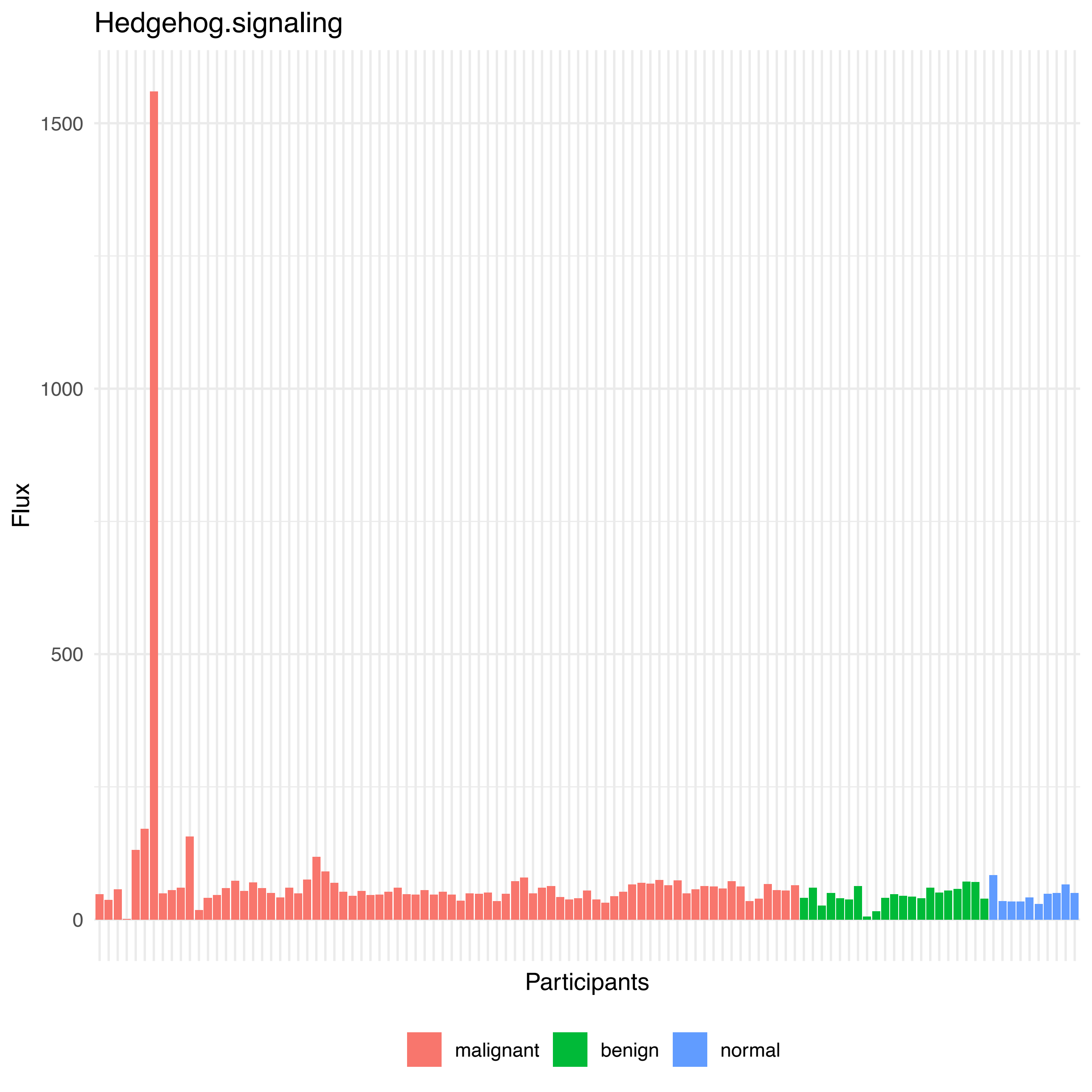

Hedgehog.signaling
Flux
1500
1000
500
0
Participants
malignant
benign
normal

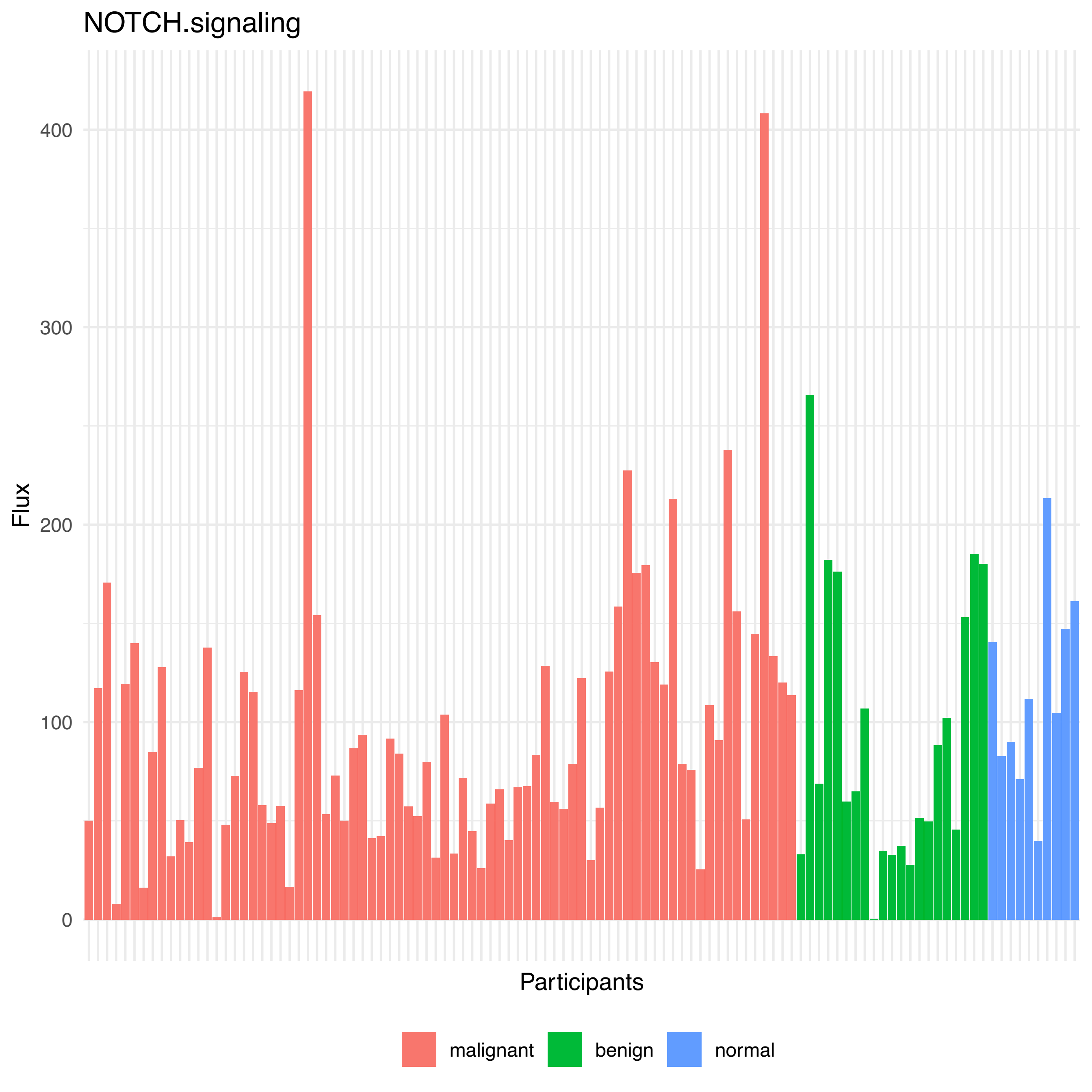

NOTCH.signaling
Flux
400
300
200
100
0
Participants
malignant
benign
normal

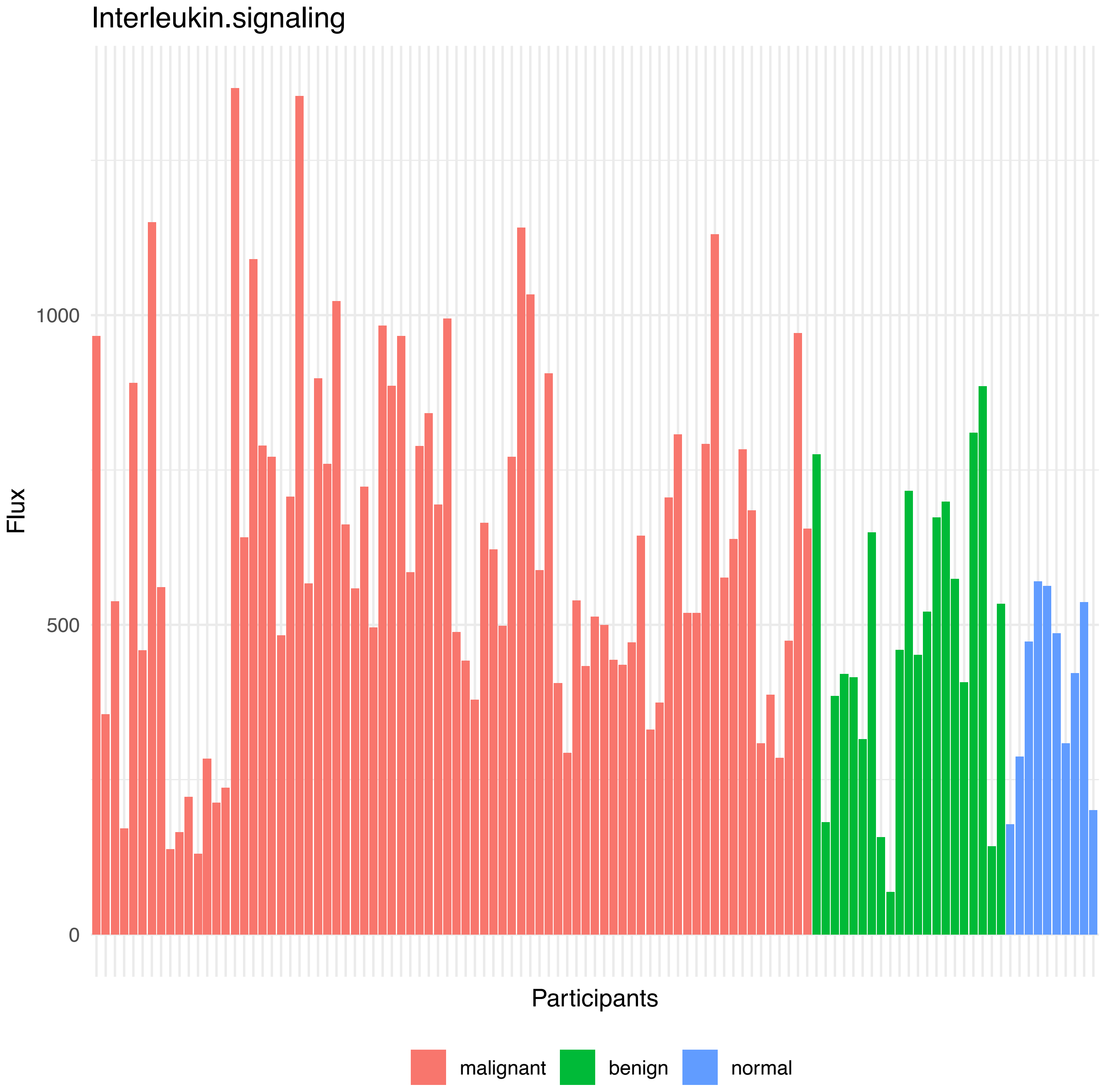
Interleukin.signaling
Flux
1000
500
0
Participants
malignant
benign
normal

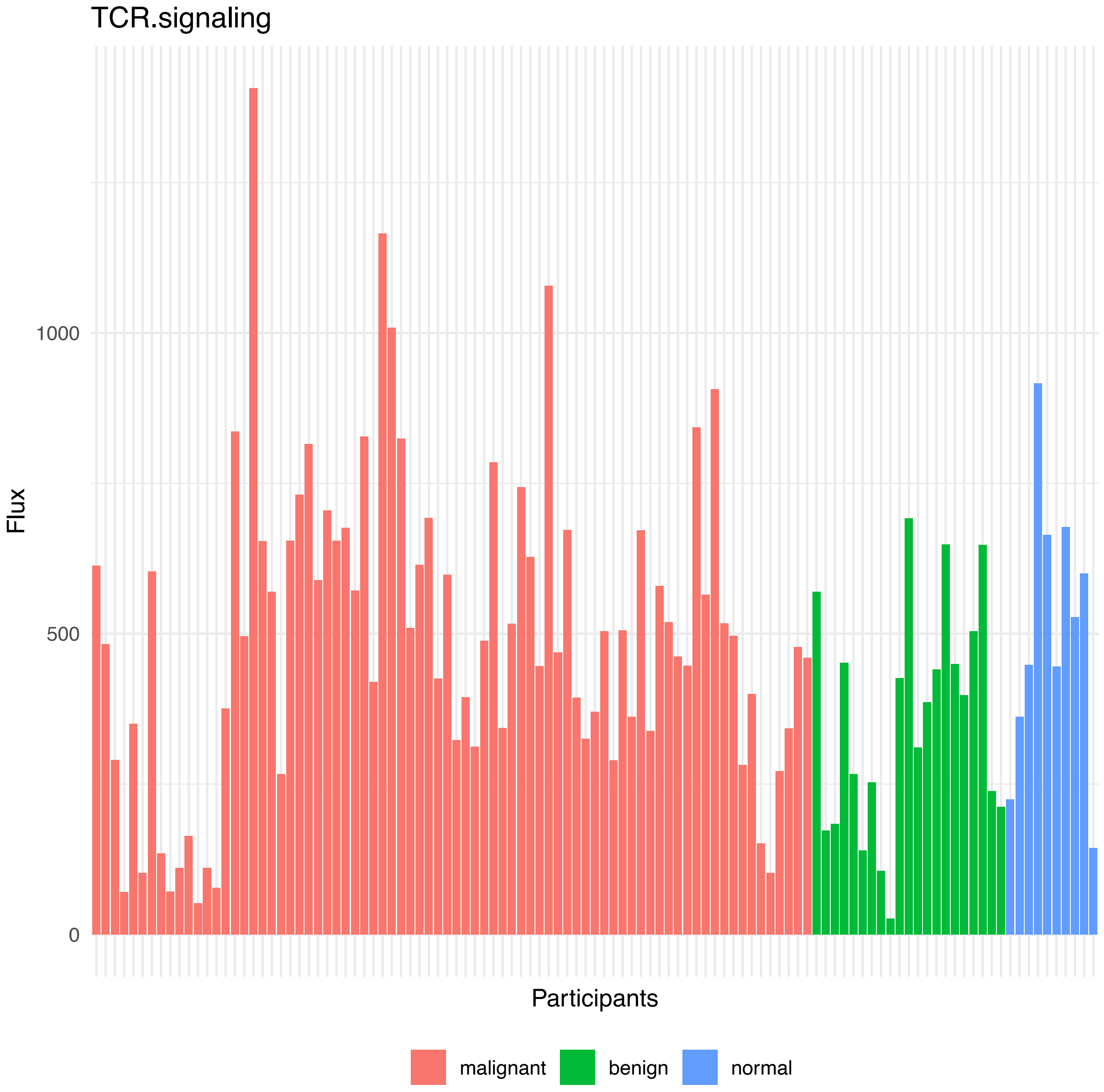

TCR.signaling
Flux
1000
500
0
Participants
malignant
benign
normal

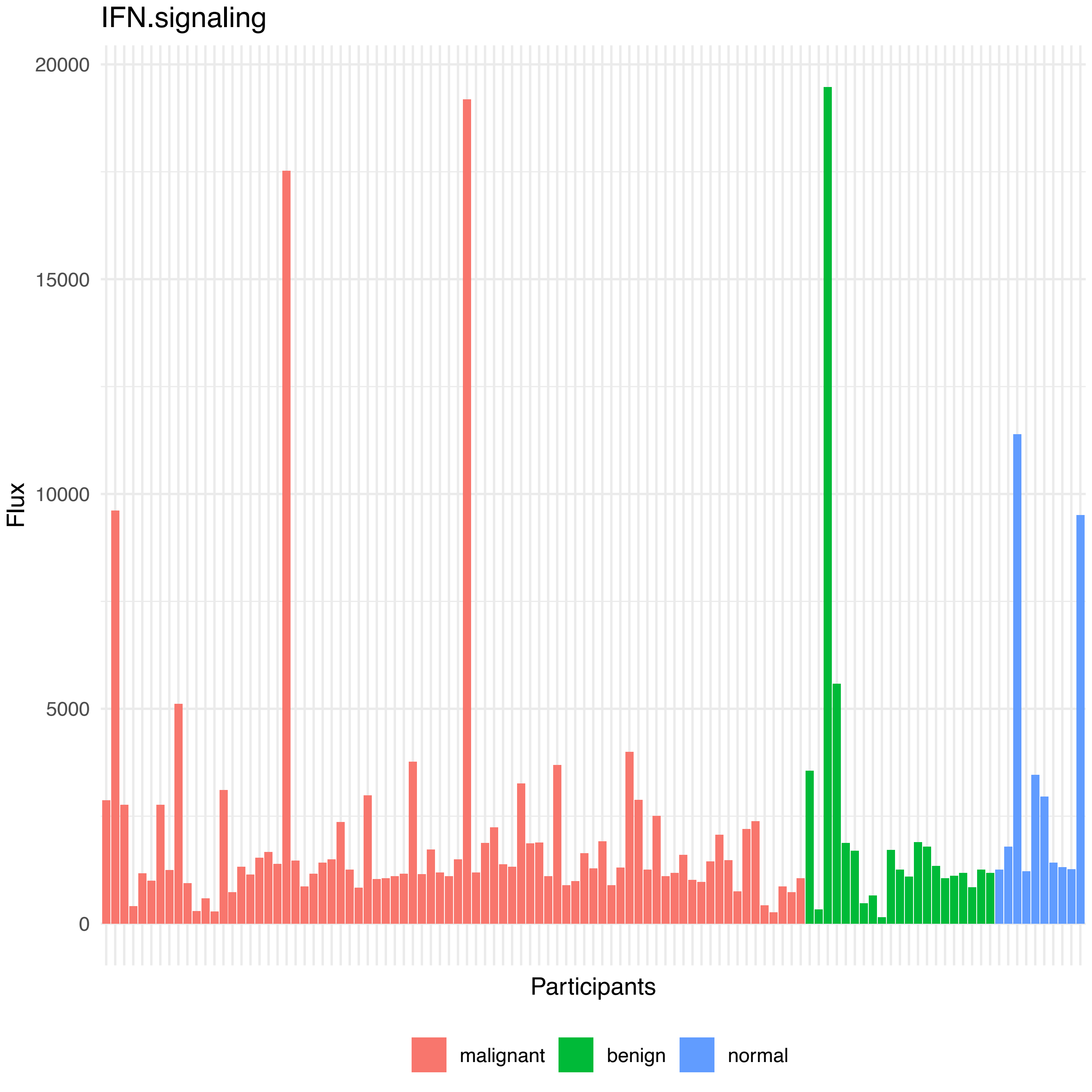

IFN.signaling
Flux
20000
15000
10000
5000
0
Participants
malignant
benign
normal

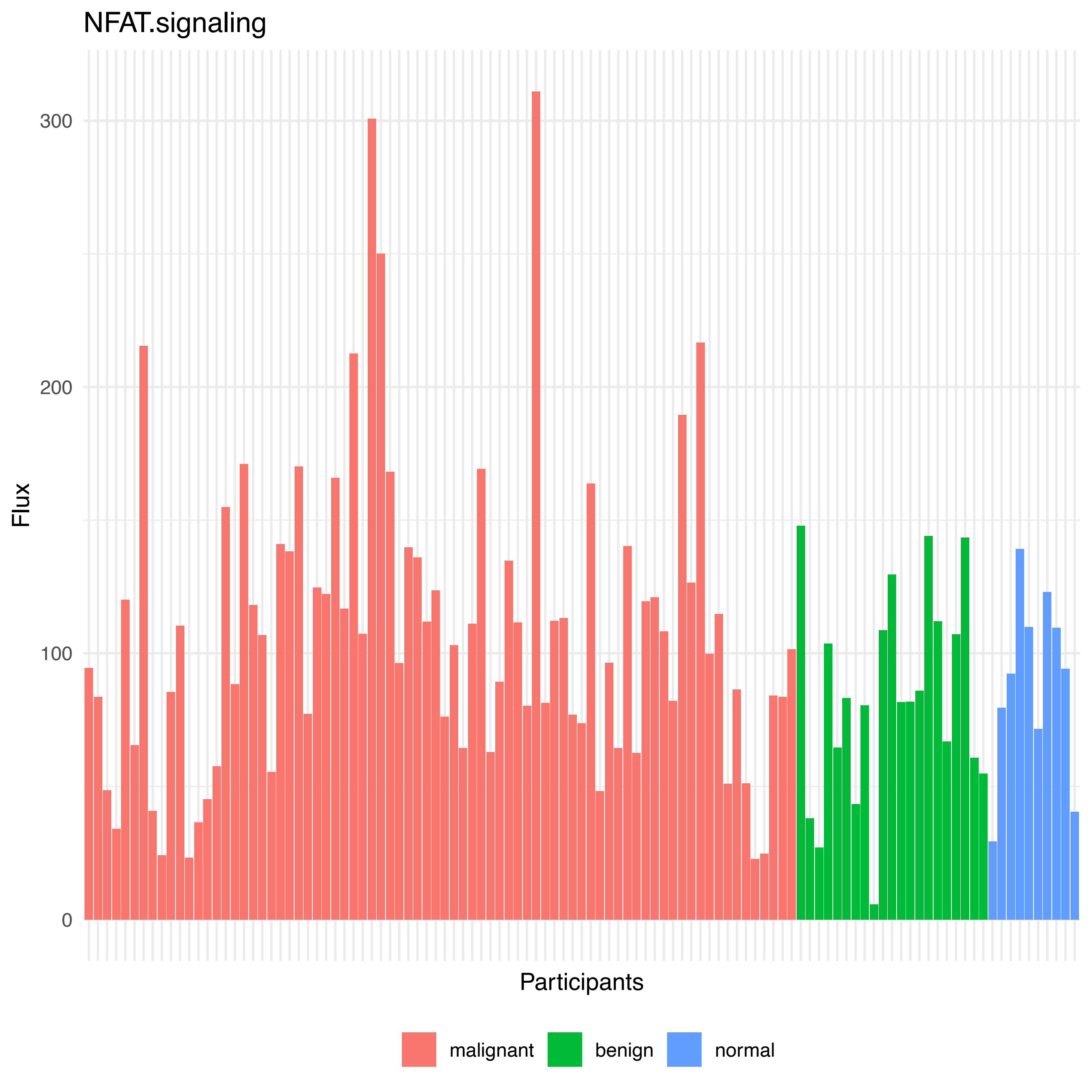

NFAT.signaling
Flux
300
200
100
0
Participants
malignant
benign
normal

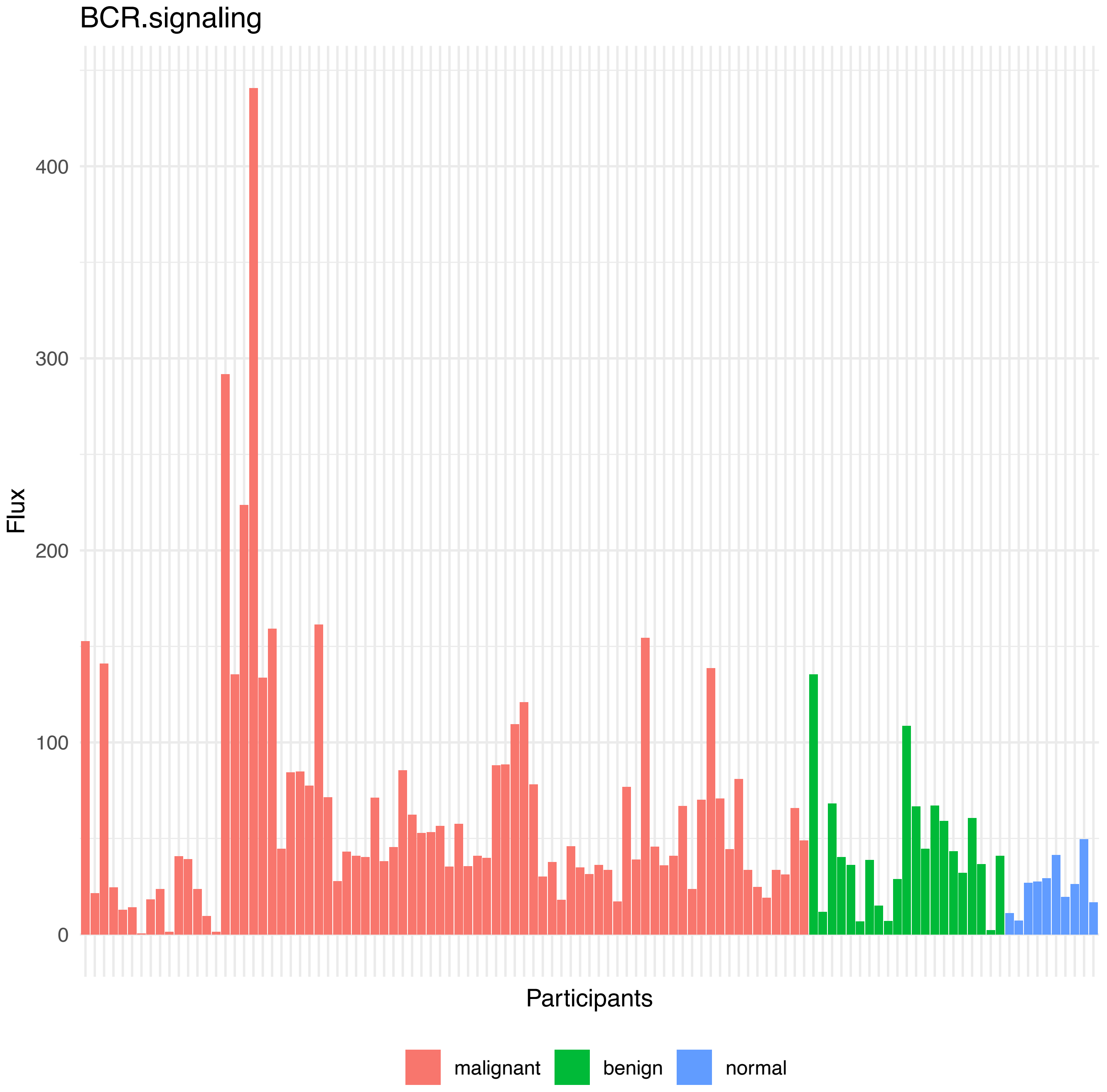
BCR.signaling
Flux
400
300
200
100
0
Participants
malignant
benign
normal

Supplement Figure 3: Pathway flux comparison between adenocarcinoma and squamous carcinoma sub-groups.

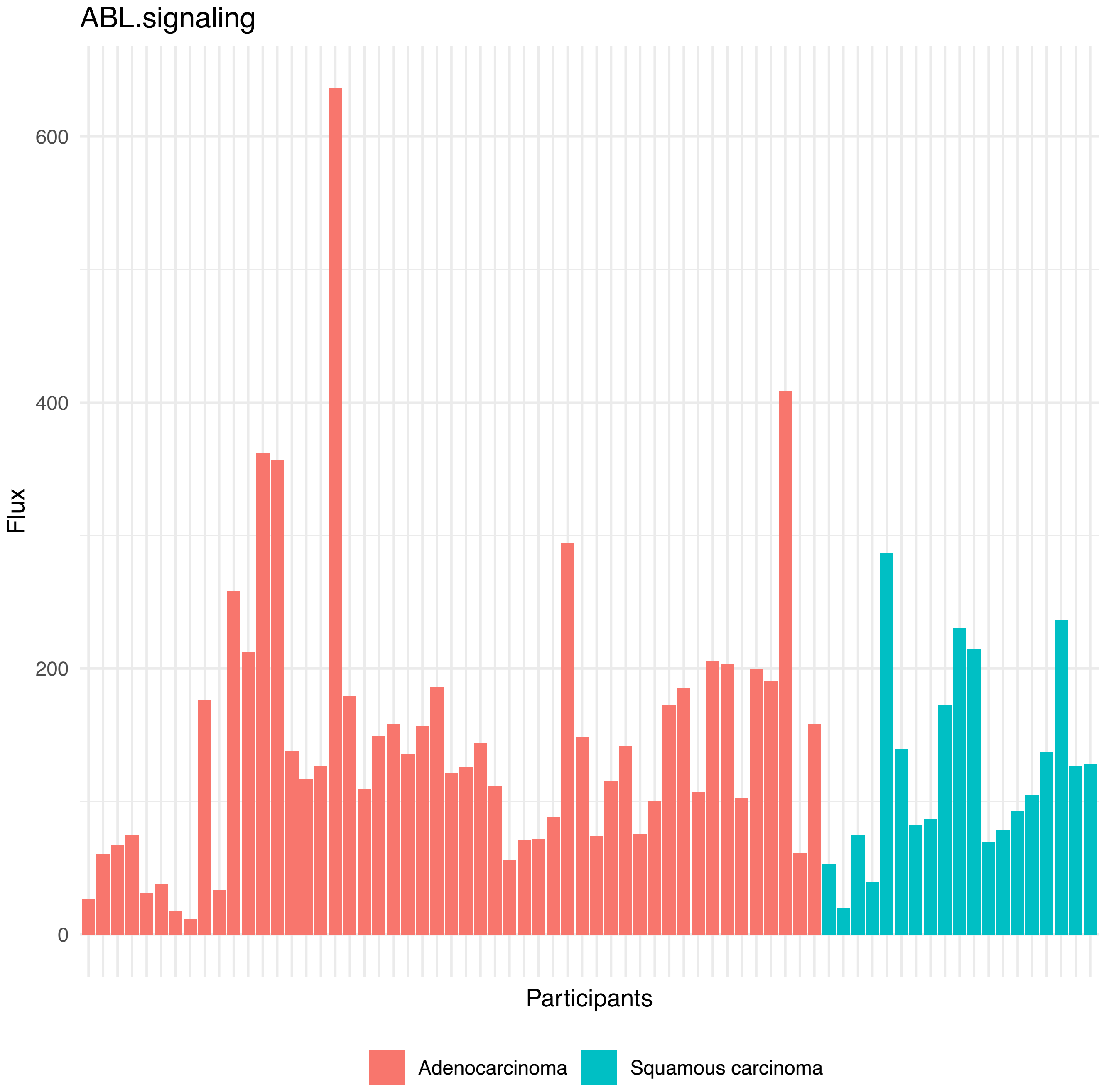
ABL.signaling
600
400
200
0
Flux
Participants
Adenocarcinoma
Squamous carcinoma

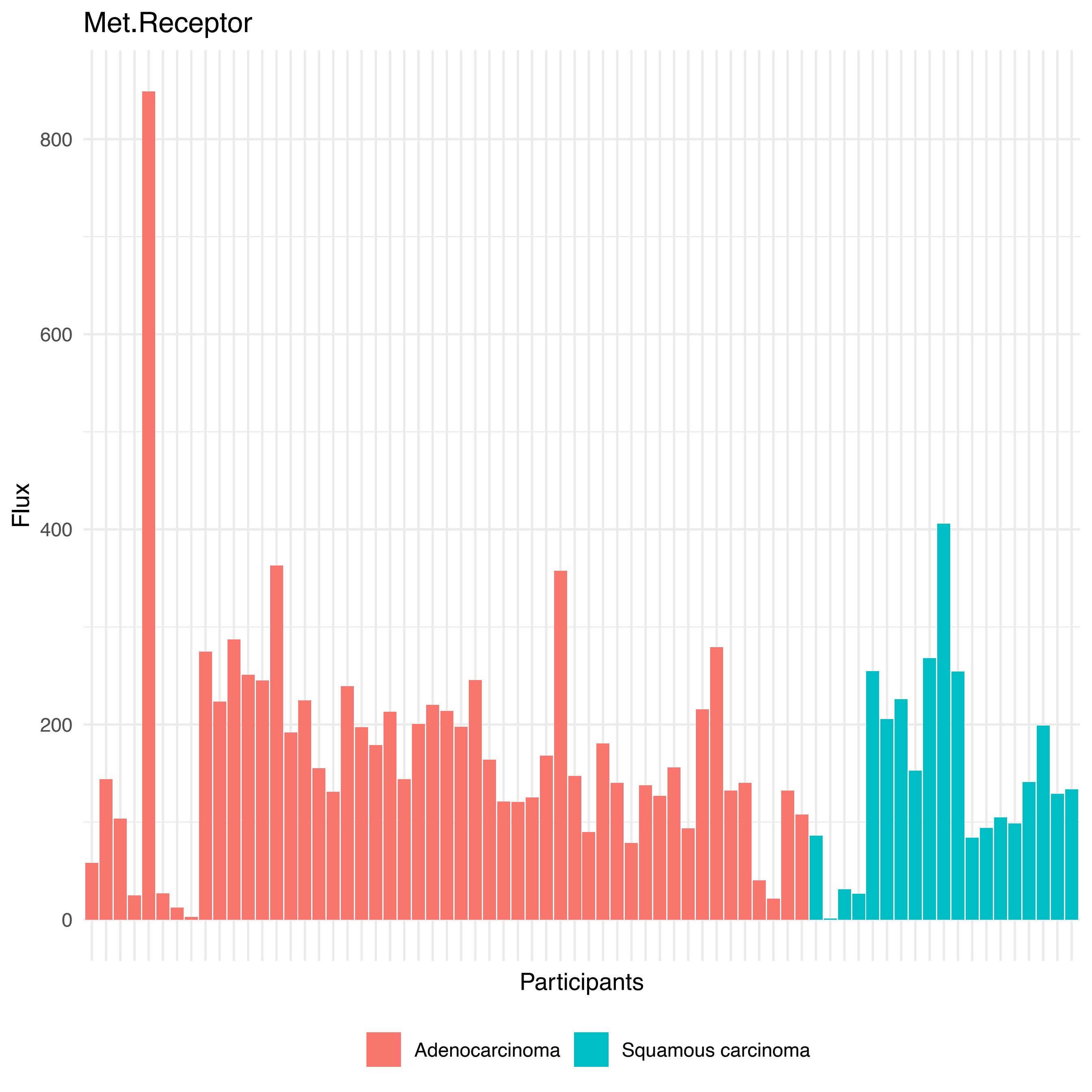

Met.Receptor
Flux
800
600
400
200
0
Participants
Adenocarcinoma
Squamous carcinoma

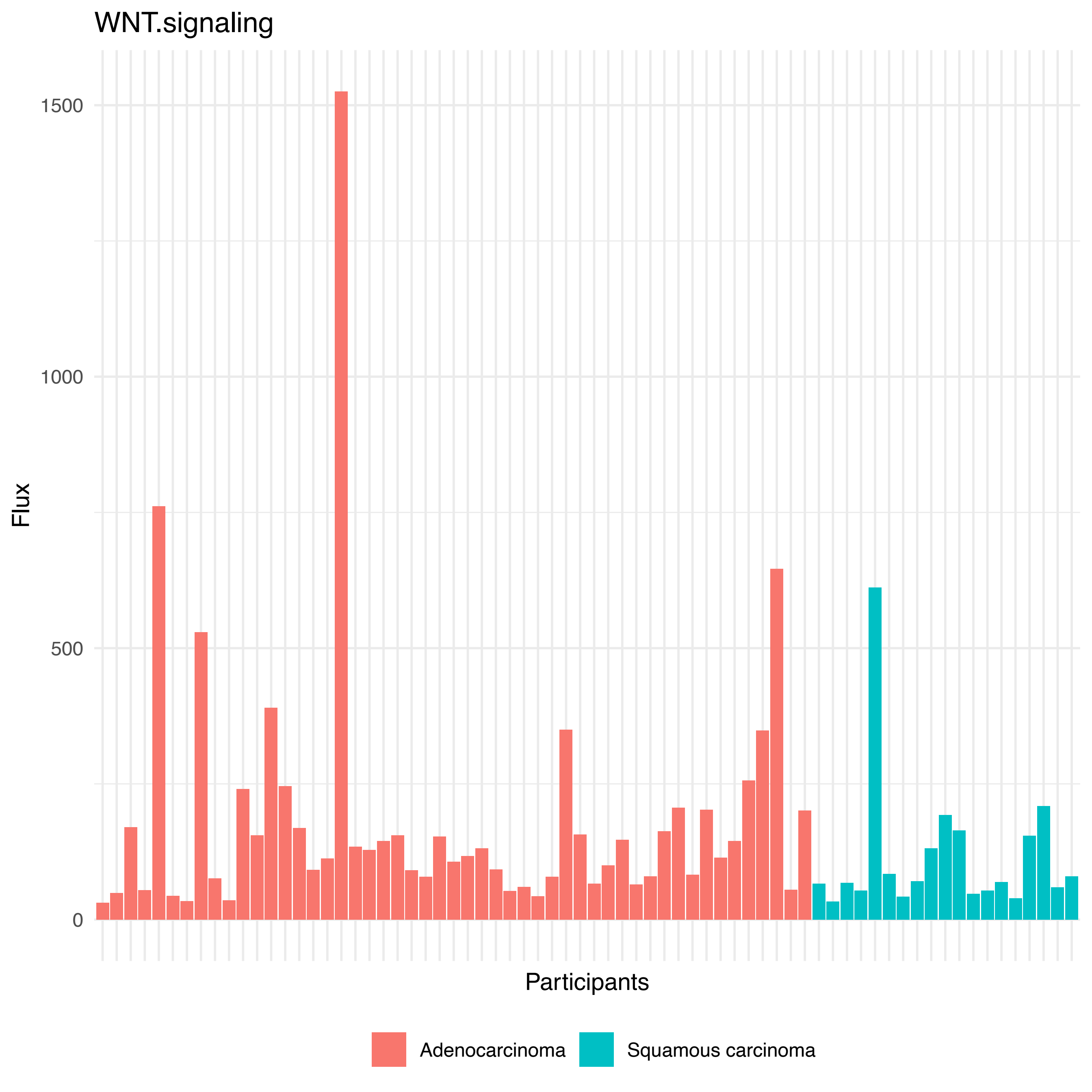
WNT.signaling
1500
1000
500
0
Flux
Participants
Adenocarcinoma
Squamous carcinoma

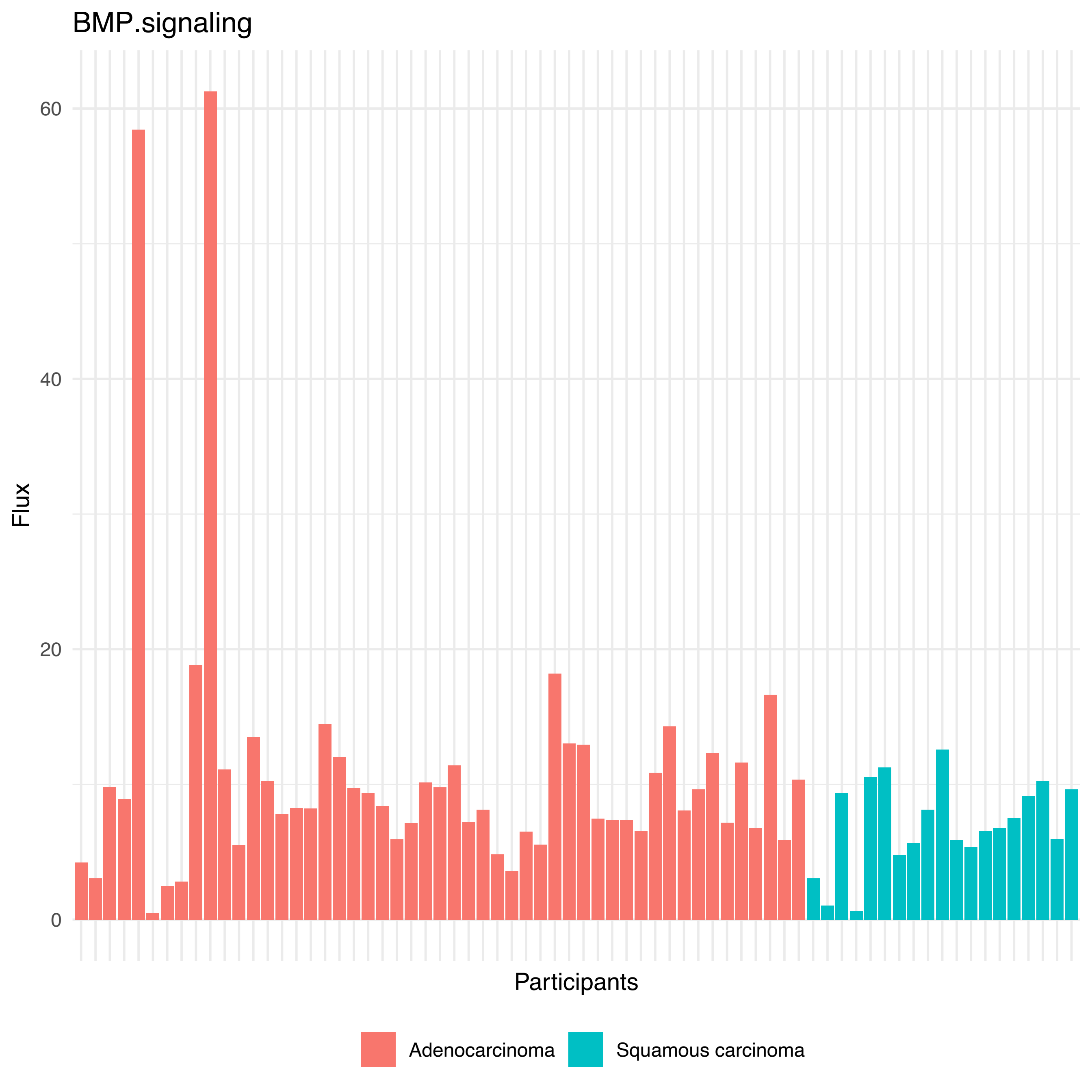

BMP.signaling
Flux
60
40
20
0
Participants
Adenocarcinoma
Squamous carcinoma

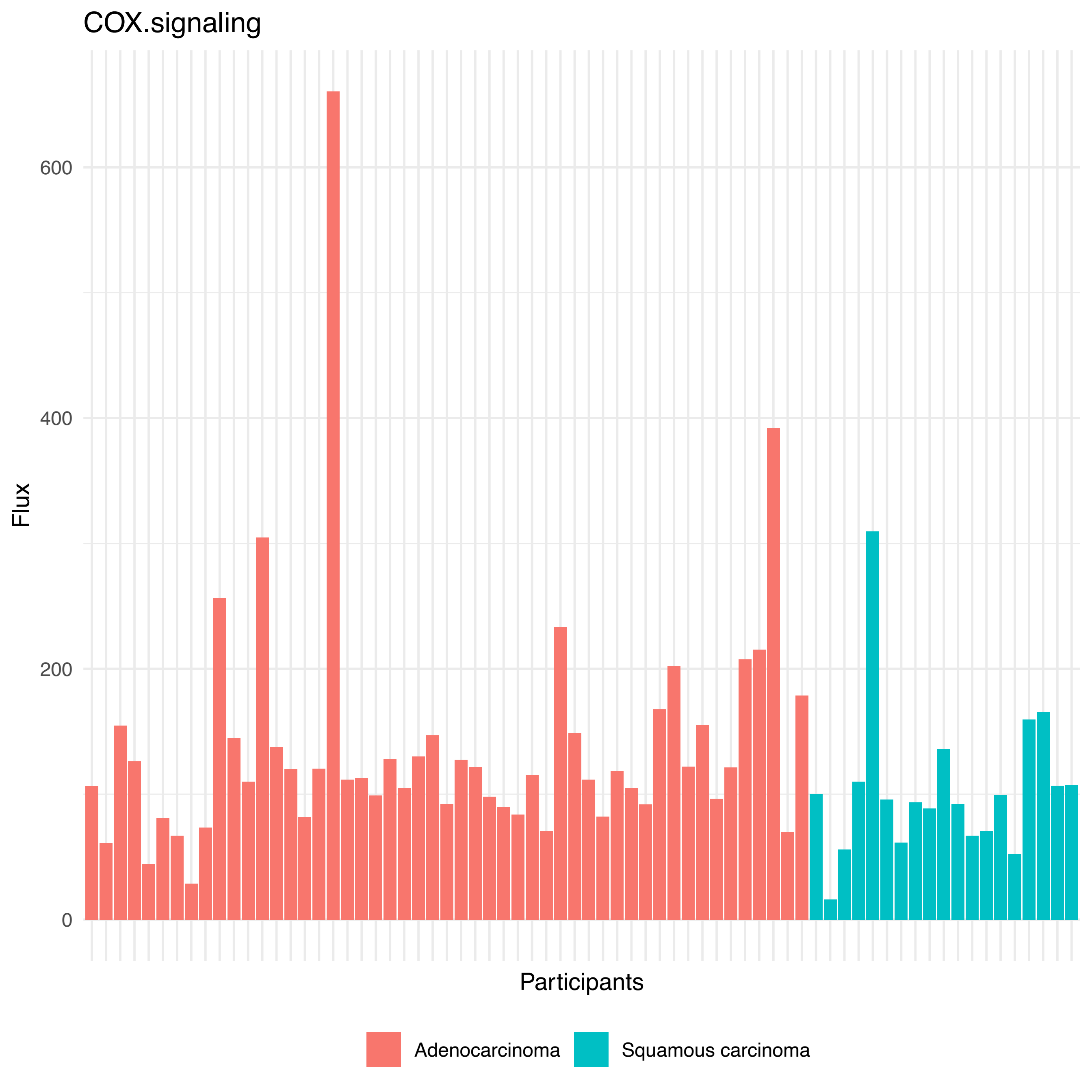
COX.signaling
Flux
600
400
200
0
Participants
Adenocarcinoma
Squamous carcinoma

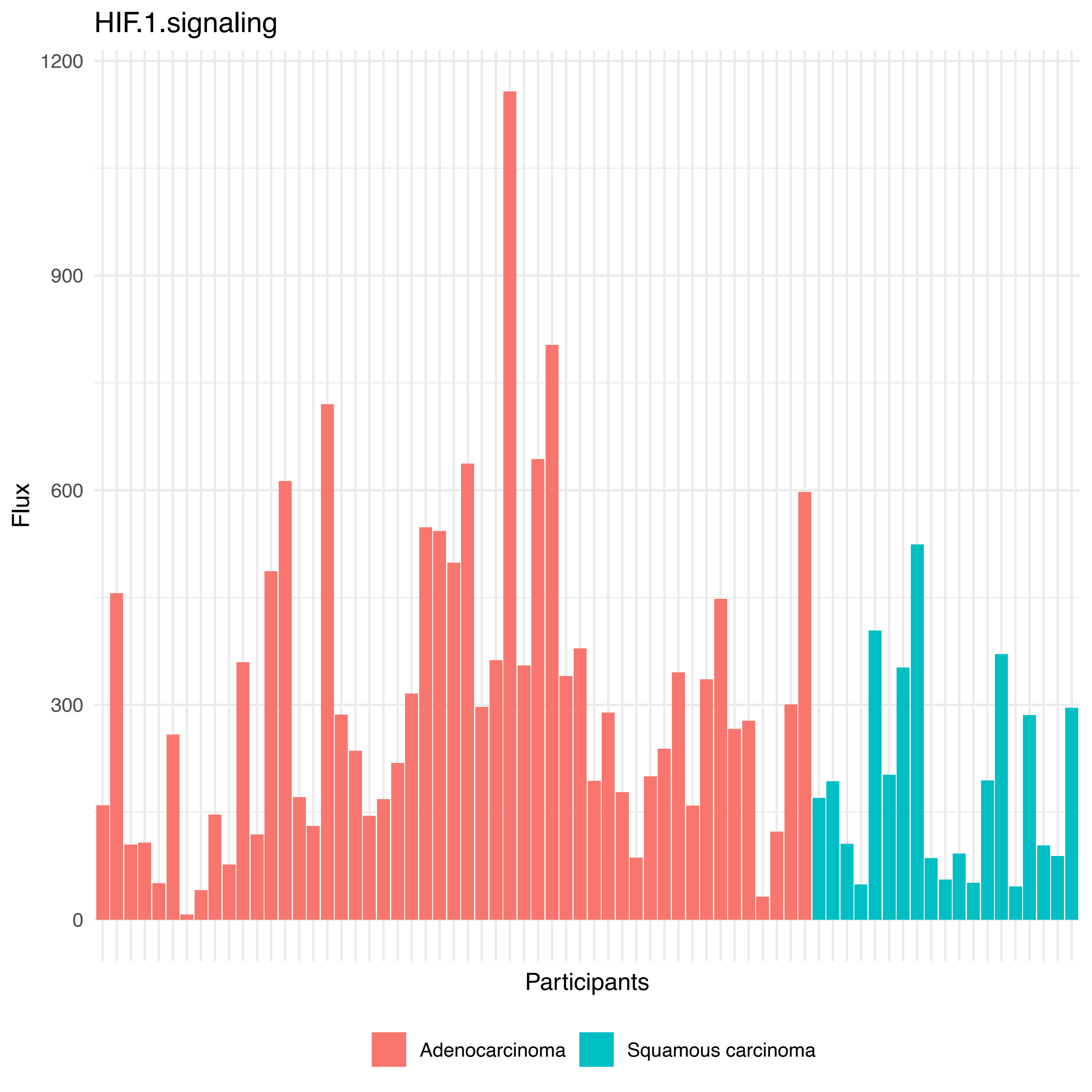
HIF.1.signaling
Flux
1200
900
600
300
0
Participants
Adenocarcinoma
Squamous carcinoma

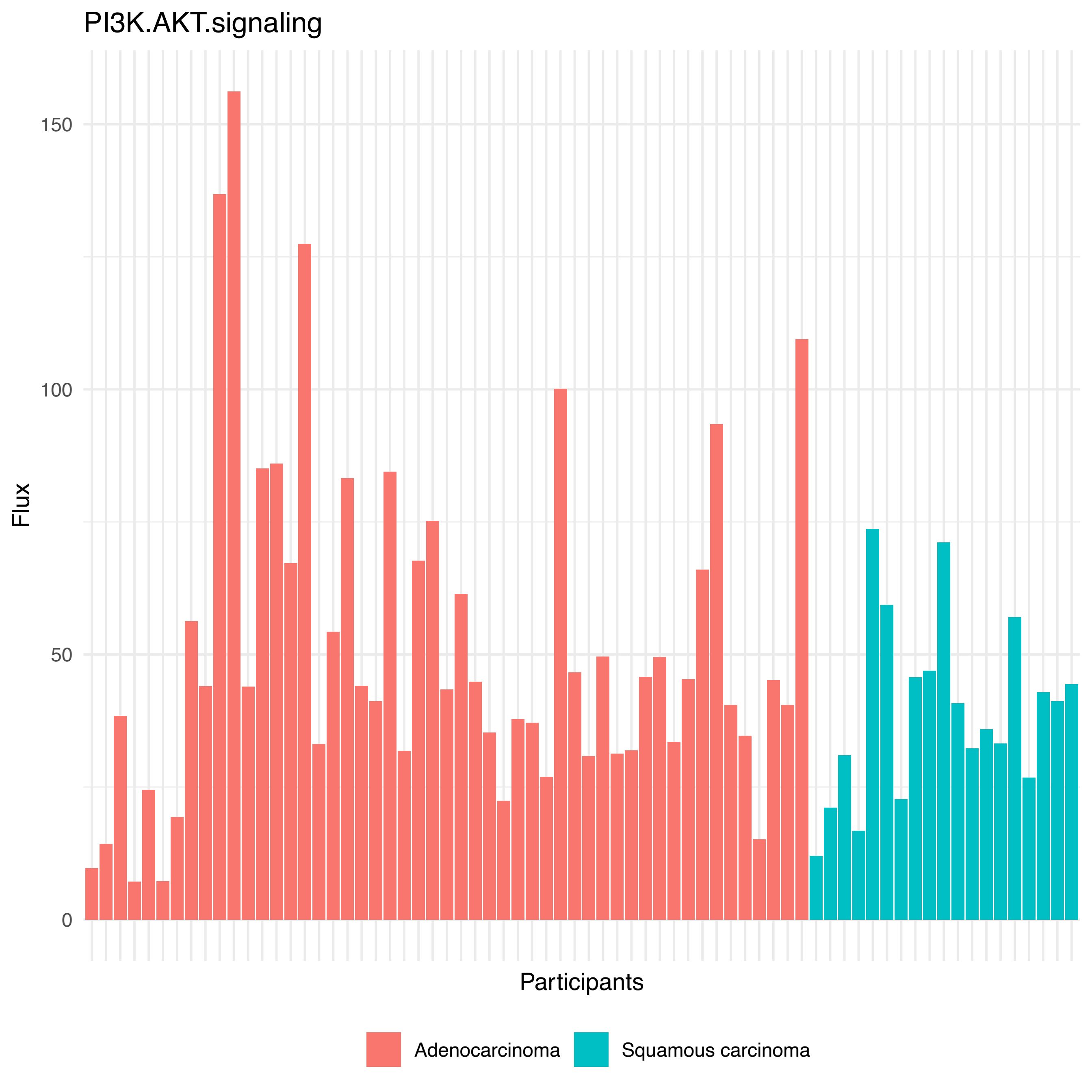

PI3K.AKT.signaling
Flux
150
100
50
0
Participants
Adenocarcinoma
Squamous carcinoma

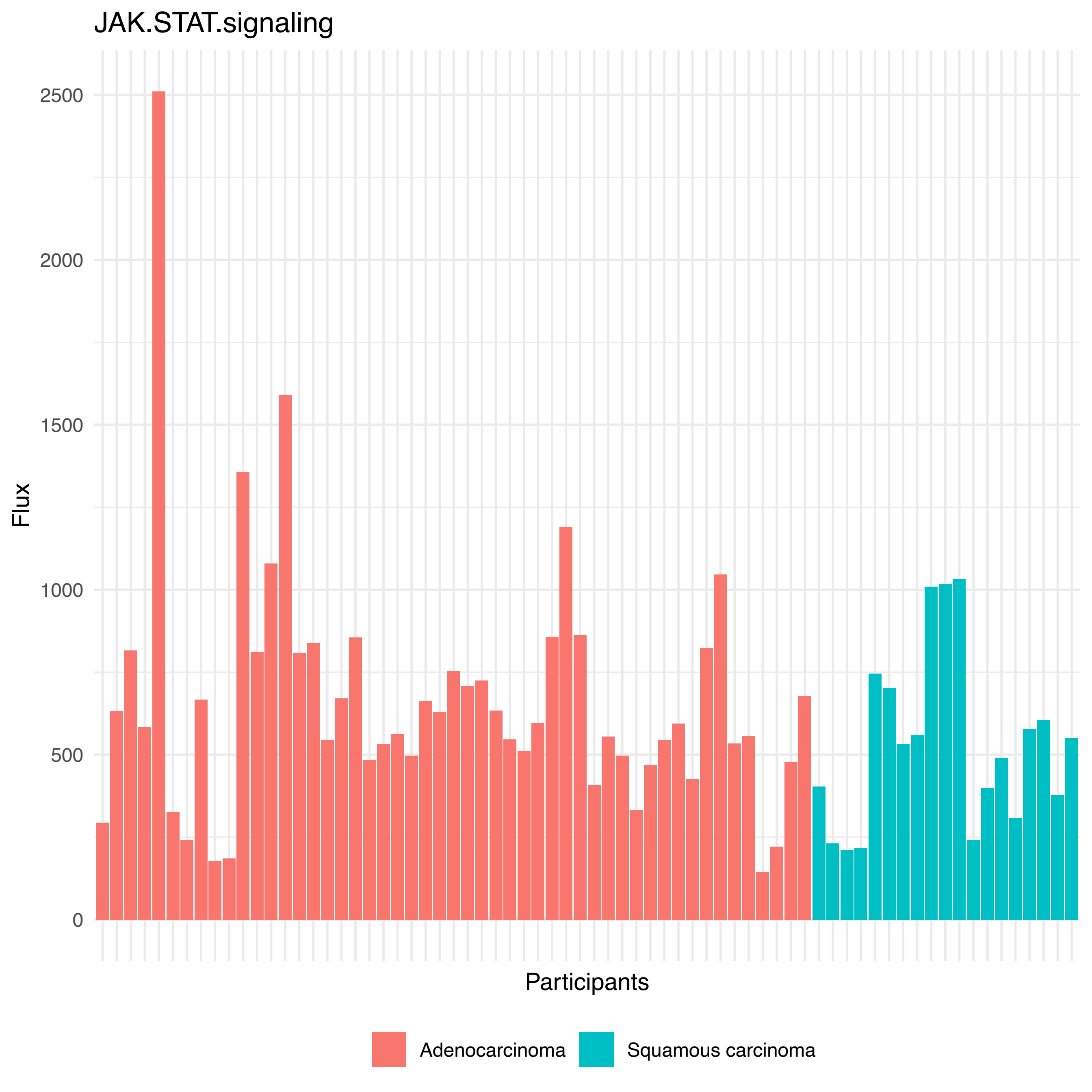
JAK.STAT.signaling
Flux
2500
2000
1500
1000
500
0
Participants
Adenocarcinoma
Squamous carcinoma

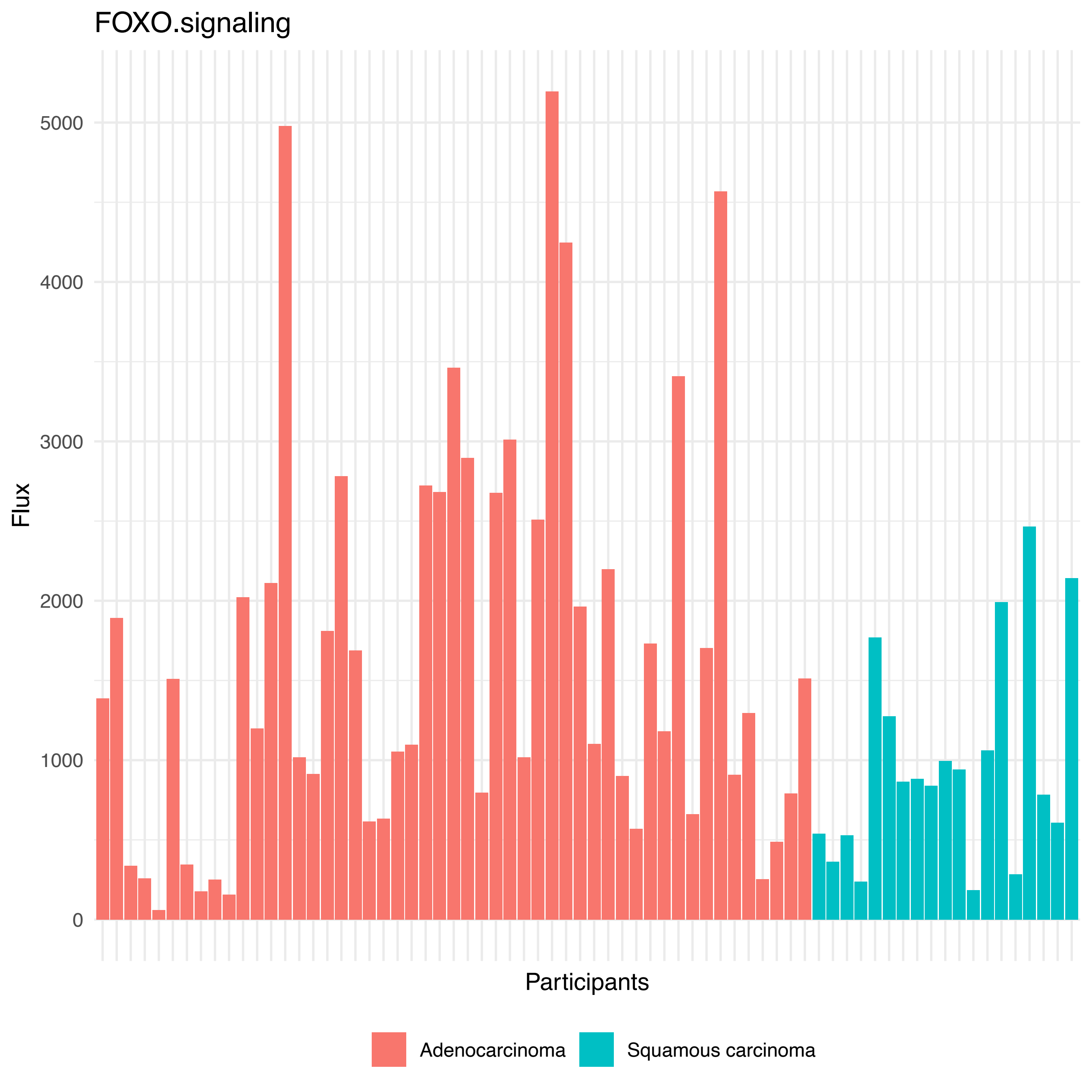

FOXO.signaling
Flux
5000
4000
3000
2000
1000
0
Participants
Adenocarcinoma
Squamous carcinoma

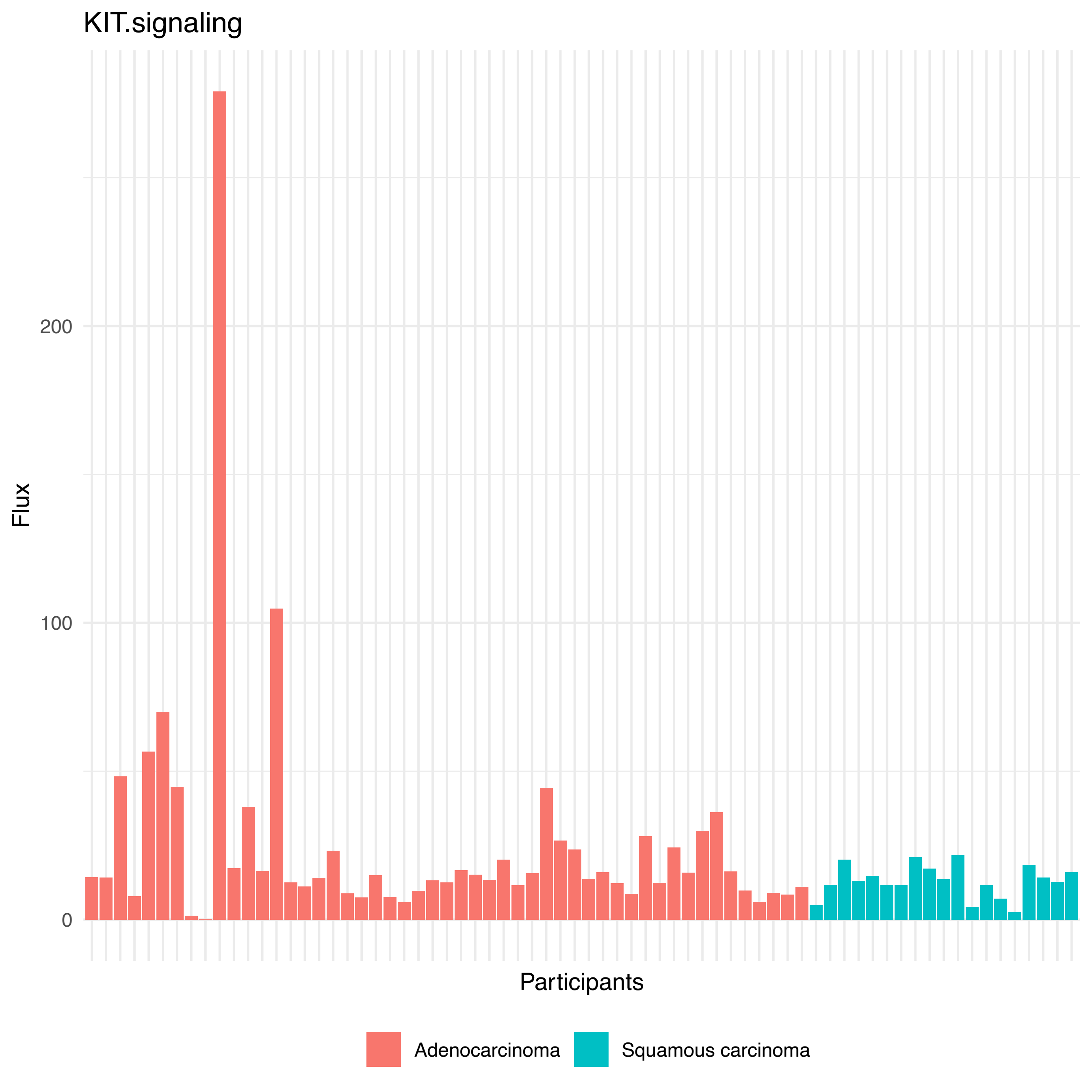
KIT.signaling
Flux
200
100
0
Participants
Adenocarcinoma
Squamous carcinoma

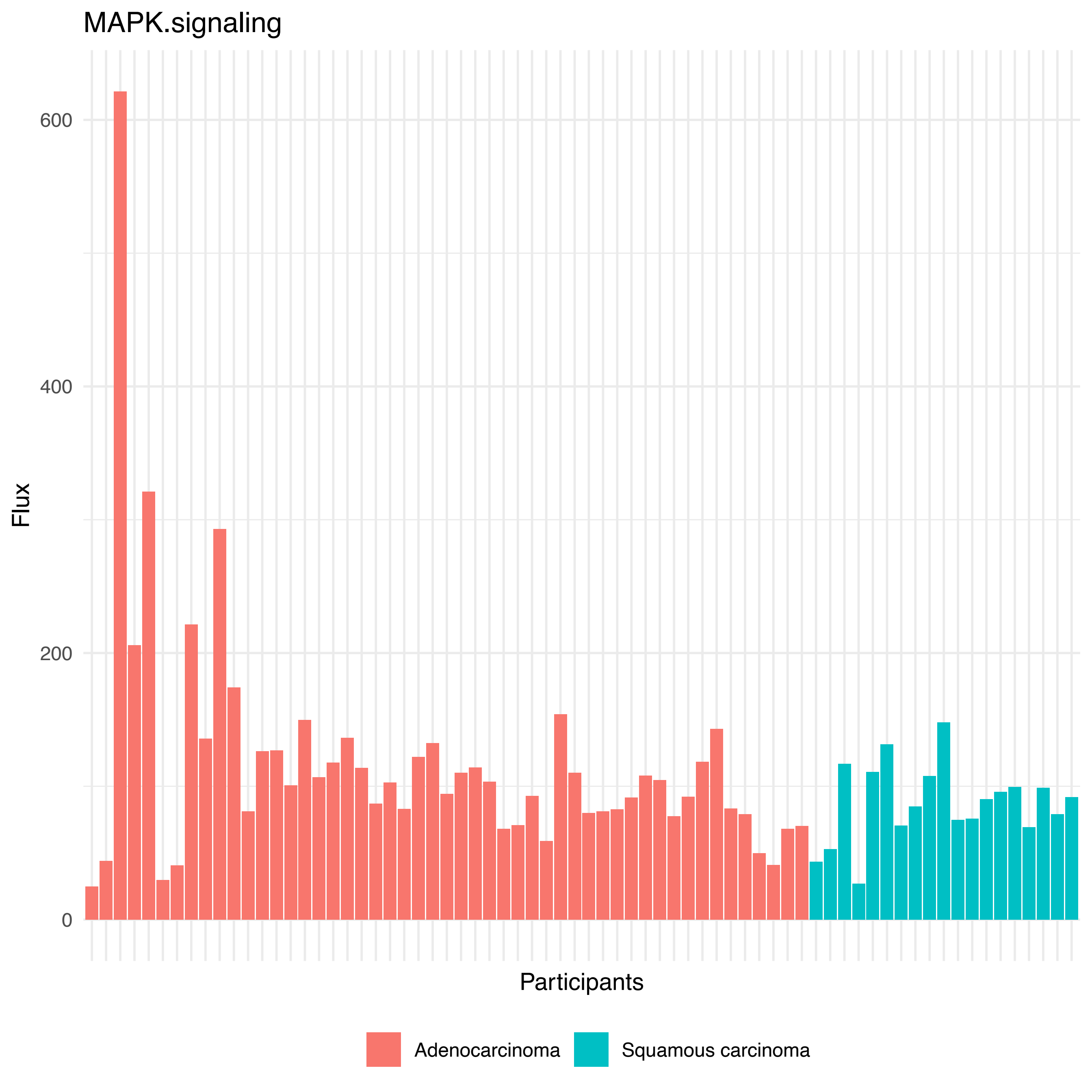
MAPK.signaling
Flux
600
400
200
0
Participants
Adenocarcinoma
Squamous carcinoma

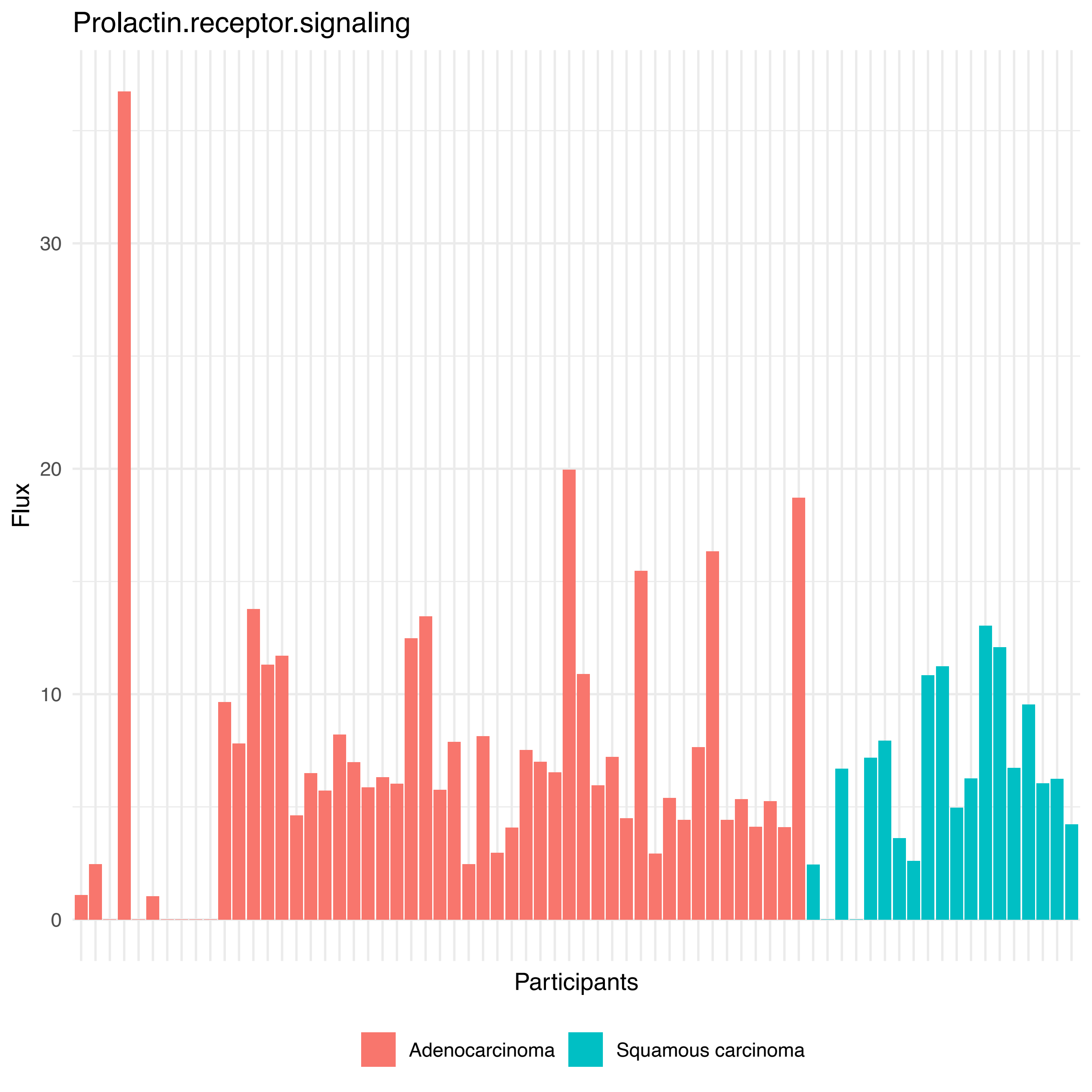

Prolactin.receptor.signaling
Flux
30
20
10
0
Participants
Adenocarcinoma
Squamous carcinoma

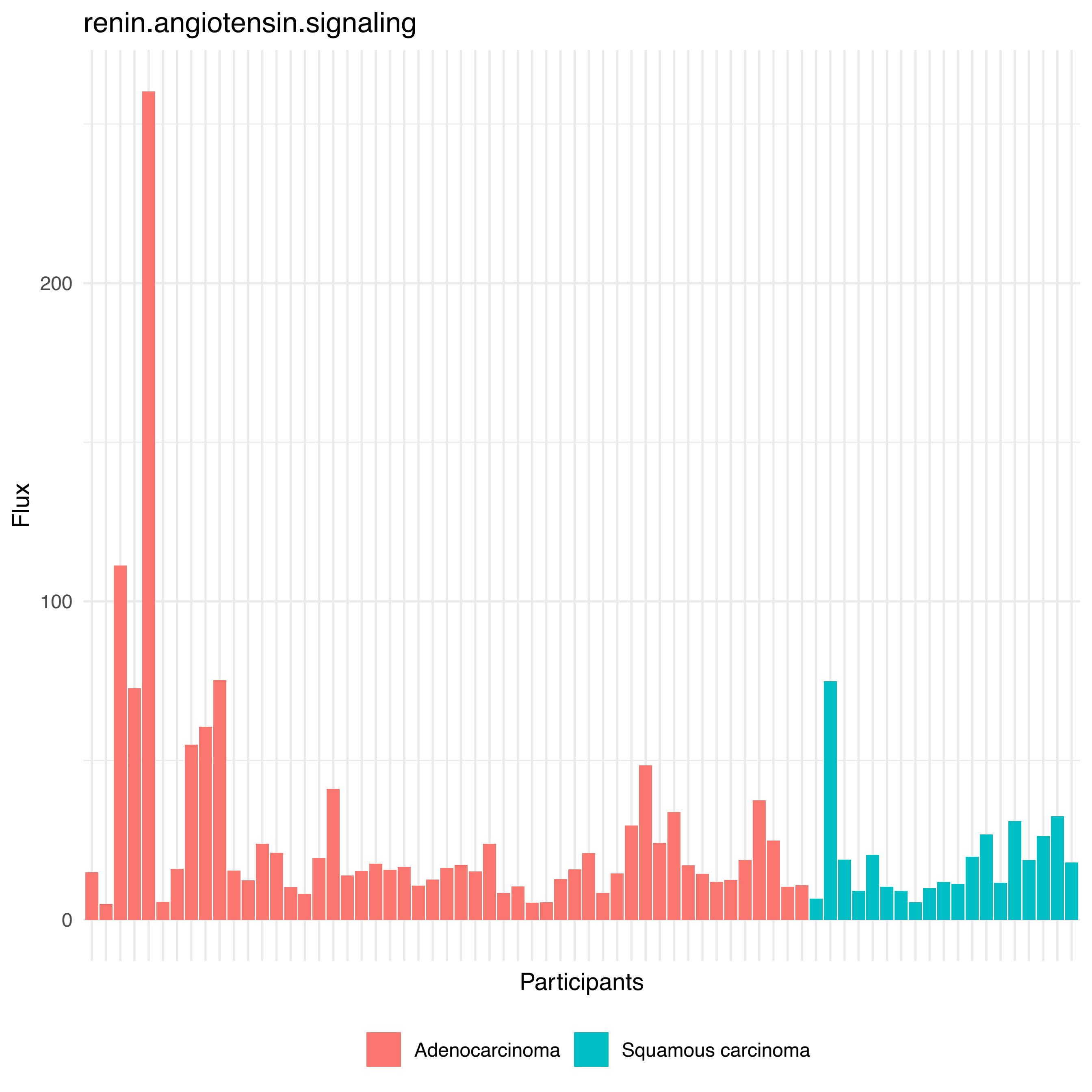

renin.angiotensin.signaling
Flux
200
100
0
Participants
Adenocarcinoma
Squamous carcinoma

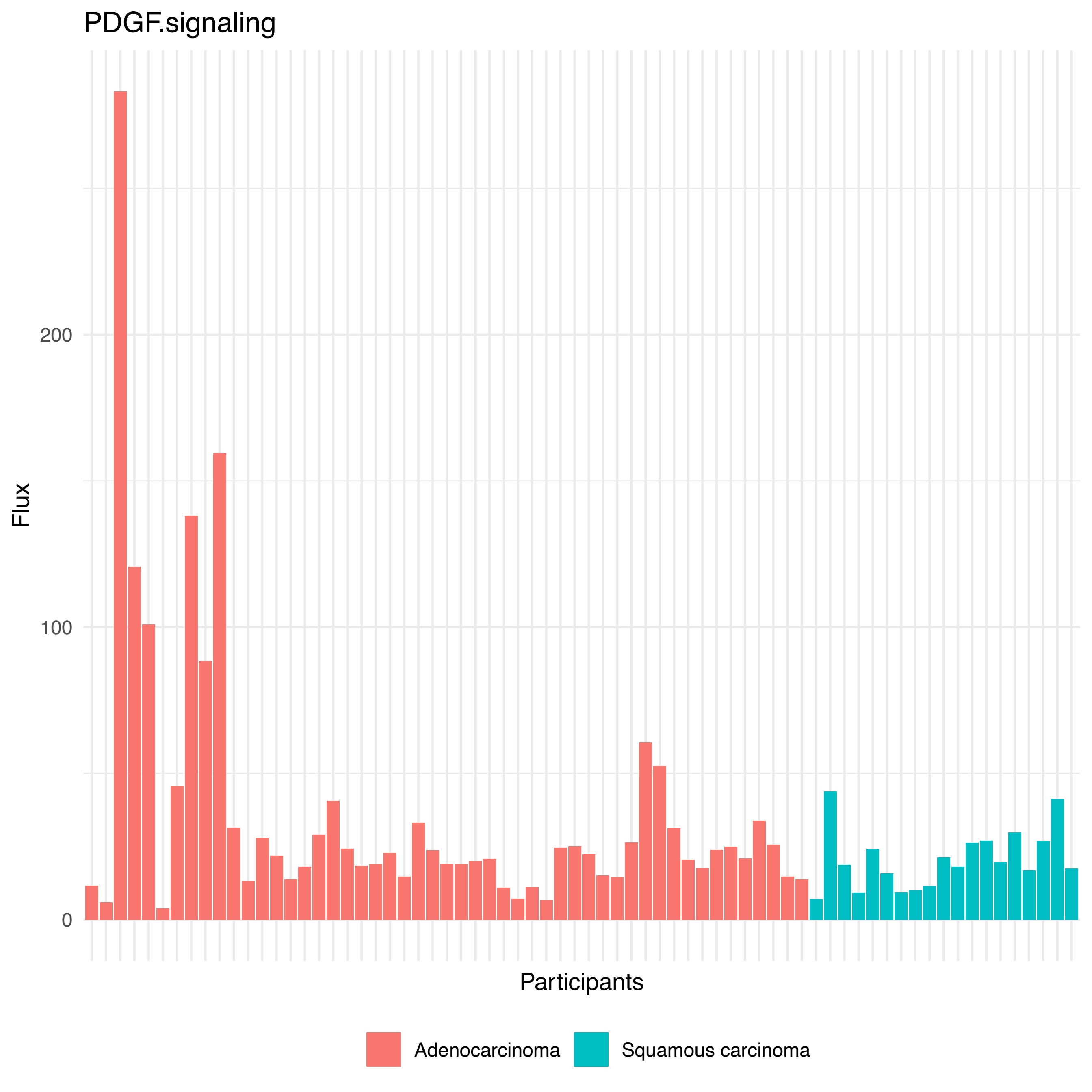
PDGF.signaling
Flux
200
100
0
Participants
Adenocarcinoma
Squamous carcinoma

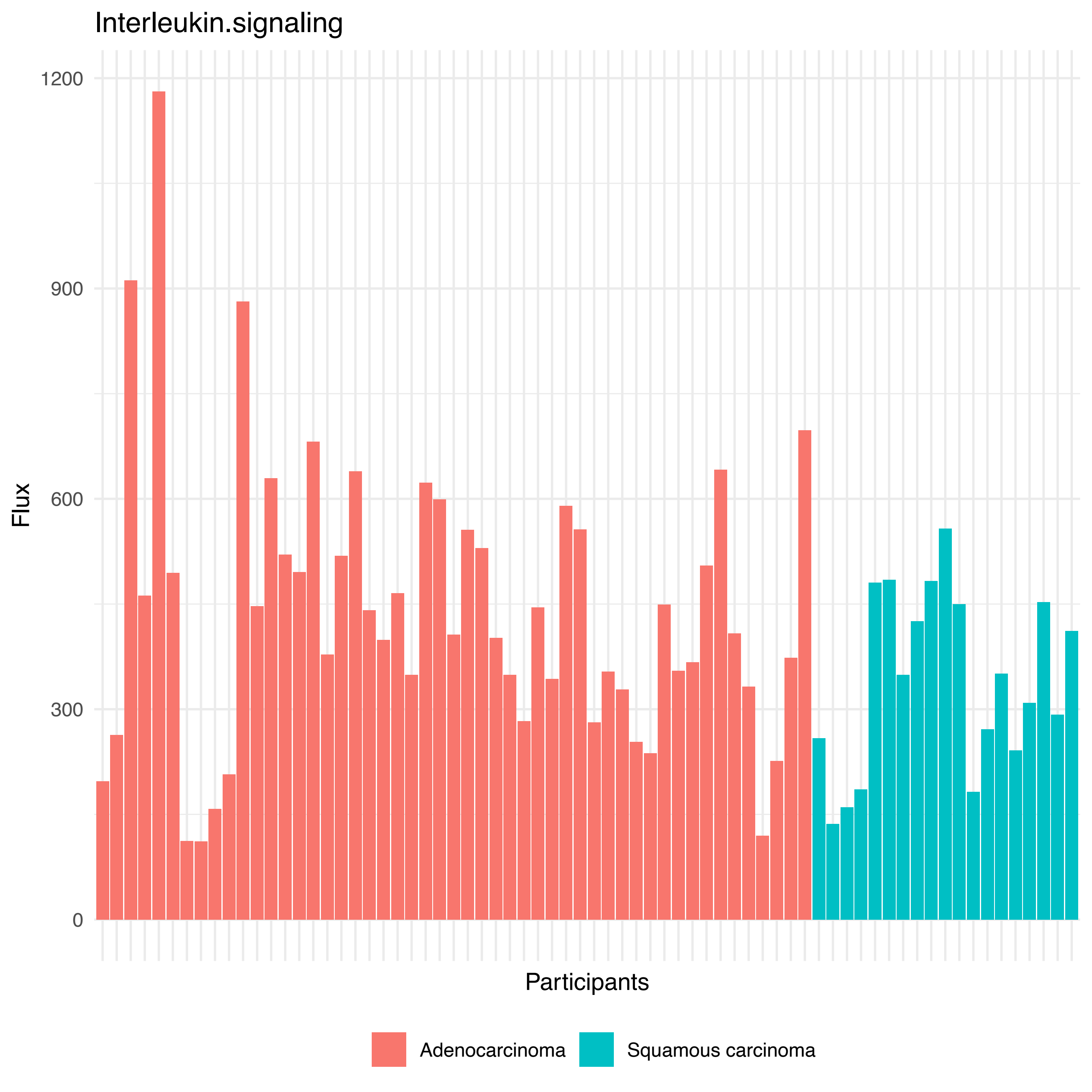

Interleukin.signaling
Flux
1200
900
600
300
0
Participants
Adenocarcinoma
Squamous carcinoma

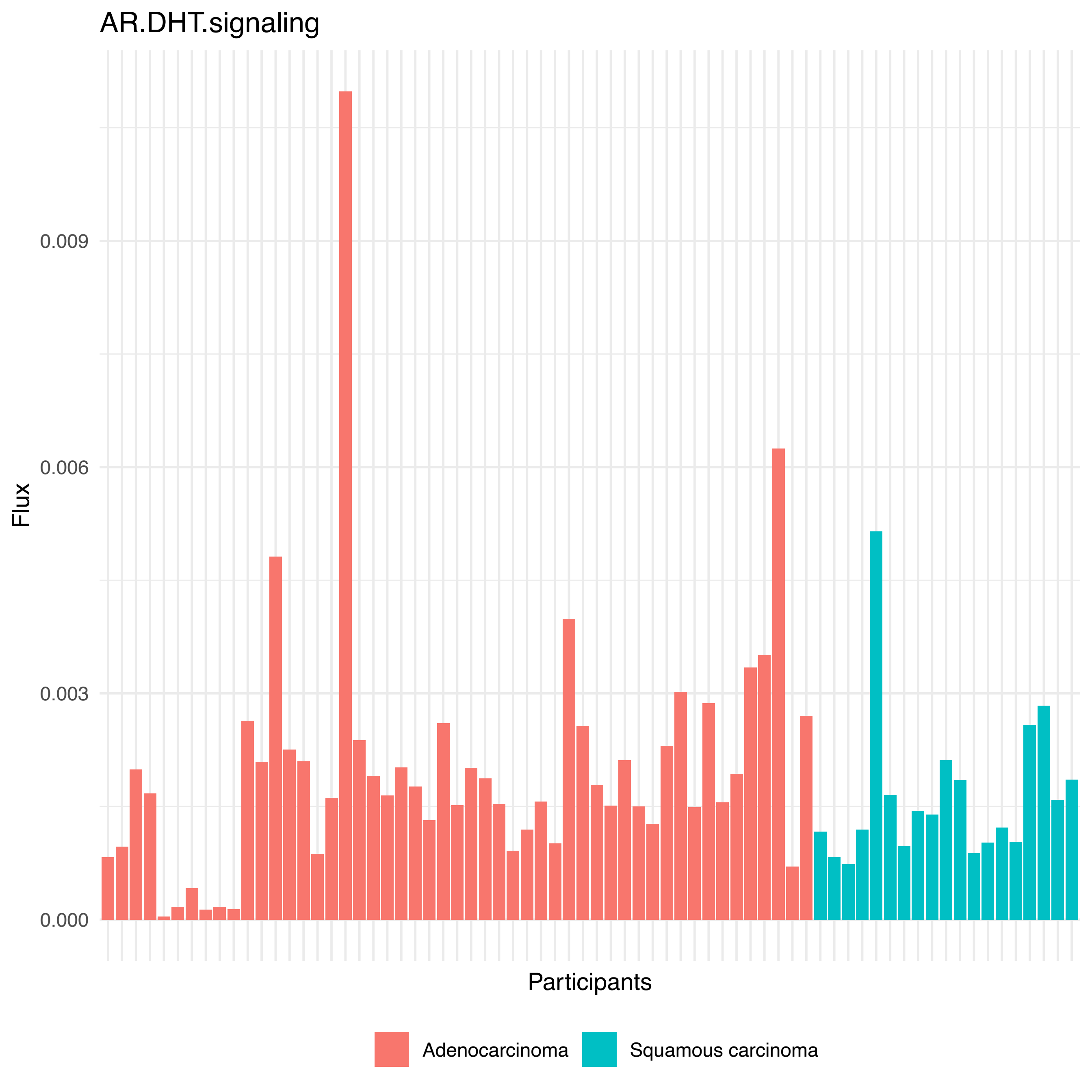
AR.DHT.signaling
Flux
0.009
0.006
0.003
0.000
Participants
Adenocarcinoma
Squamous carcinoma

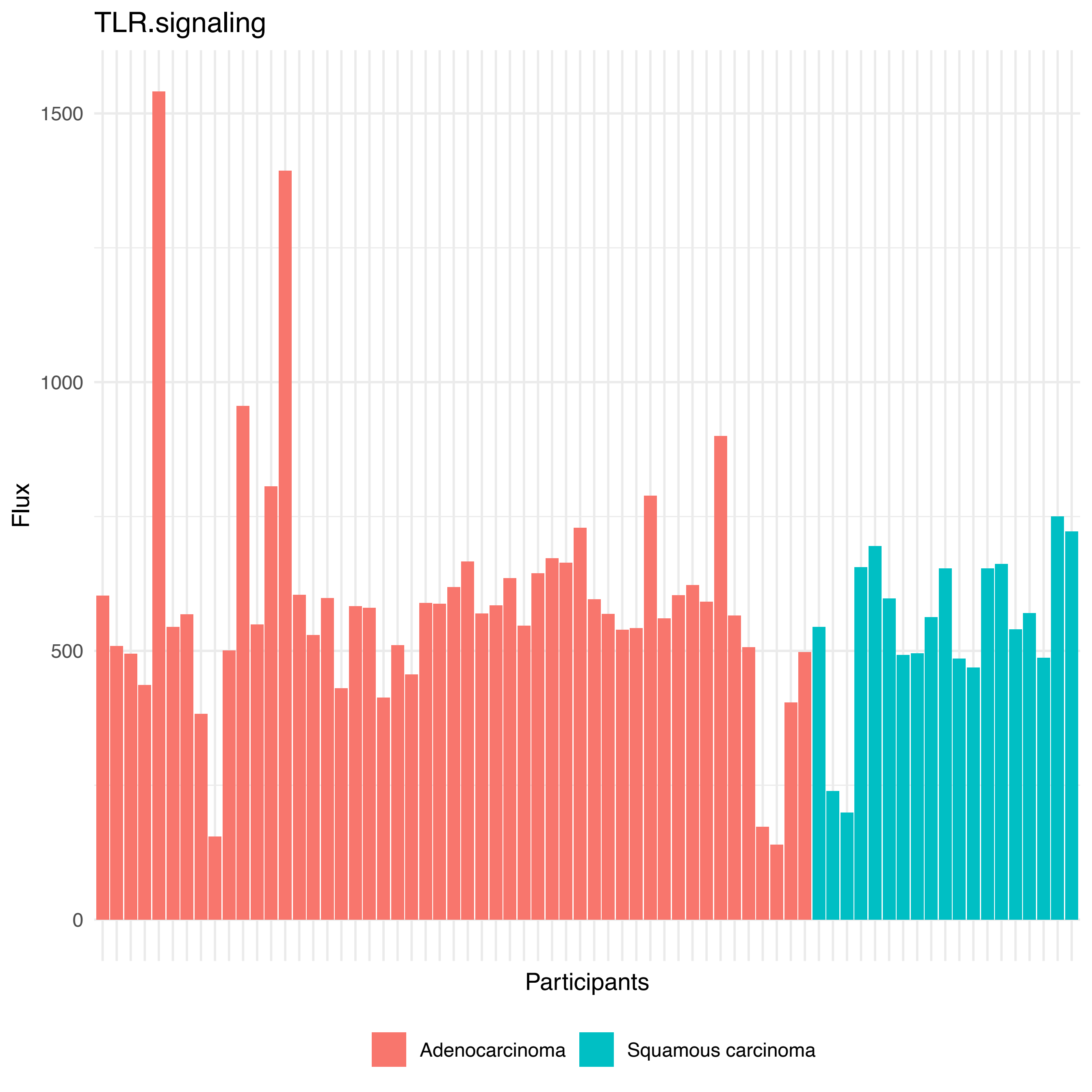

TLR.signaling
1500
1000
500
0
Flux
Participants
Adenocarcinoma
Squamous carcinoma

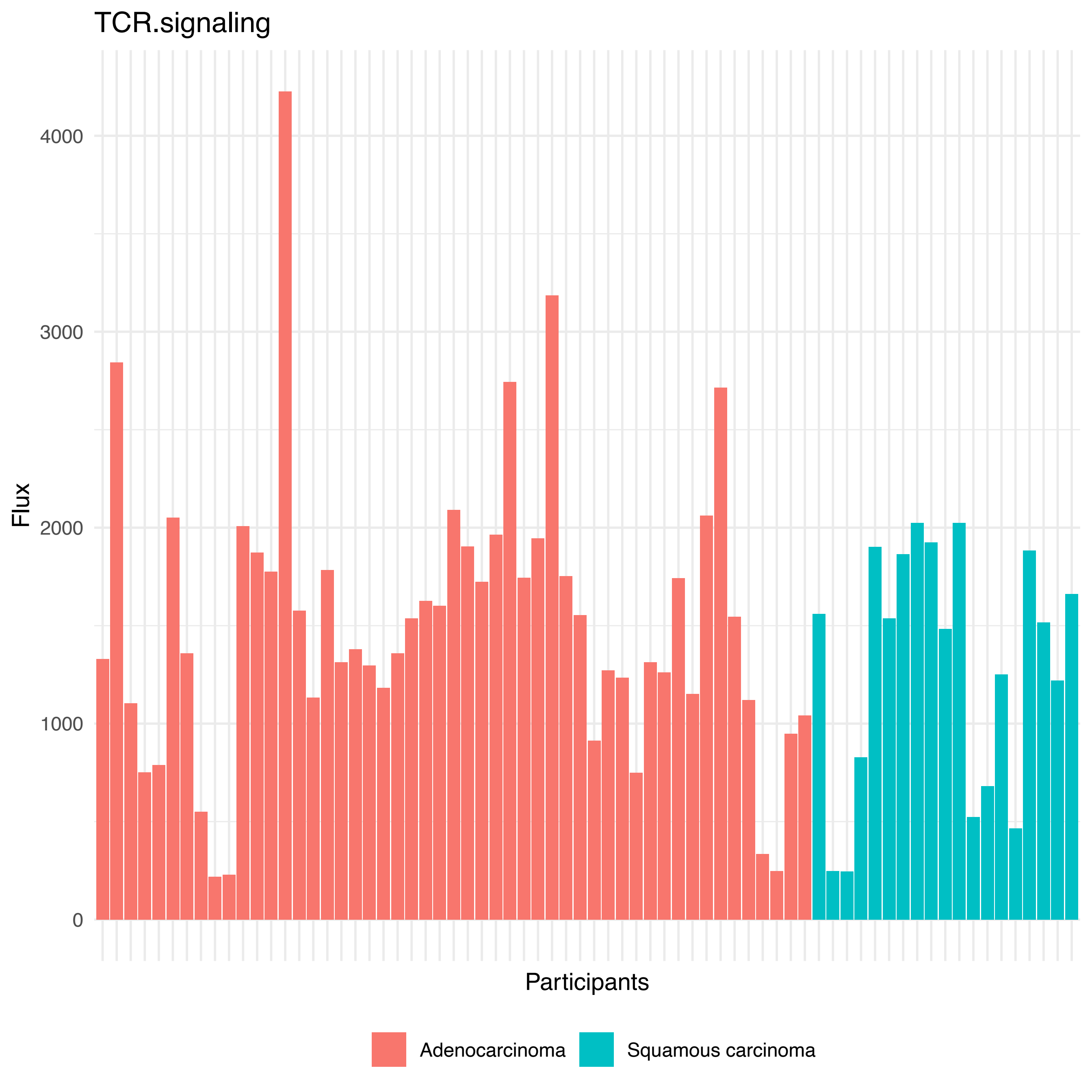

TCR.signaling
Flux
4000
3000
2000
1000
0
Participants
Adenocarcinoma
Squamous carcinoma

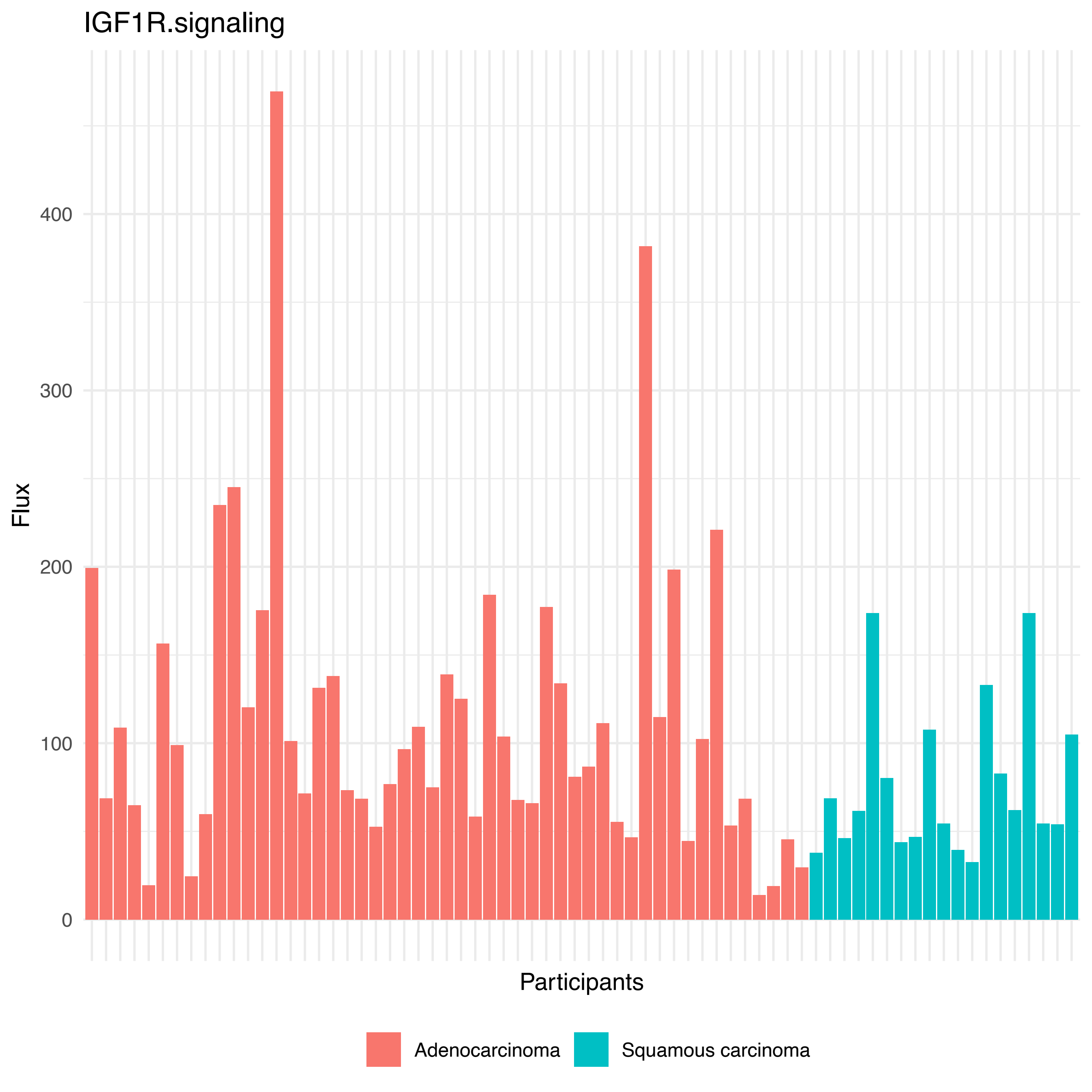

IGF1R.signaling
Flux
400
300
200
100
0
Participants
Adenocarcinoma
Squamous carcinoma

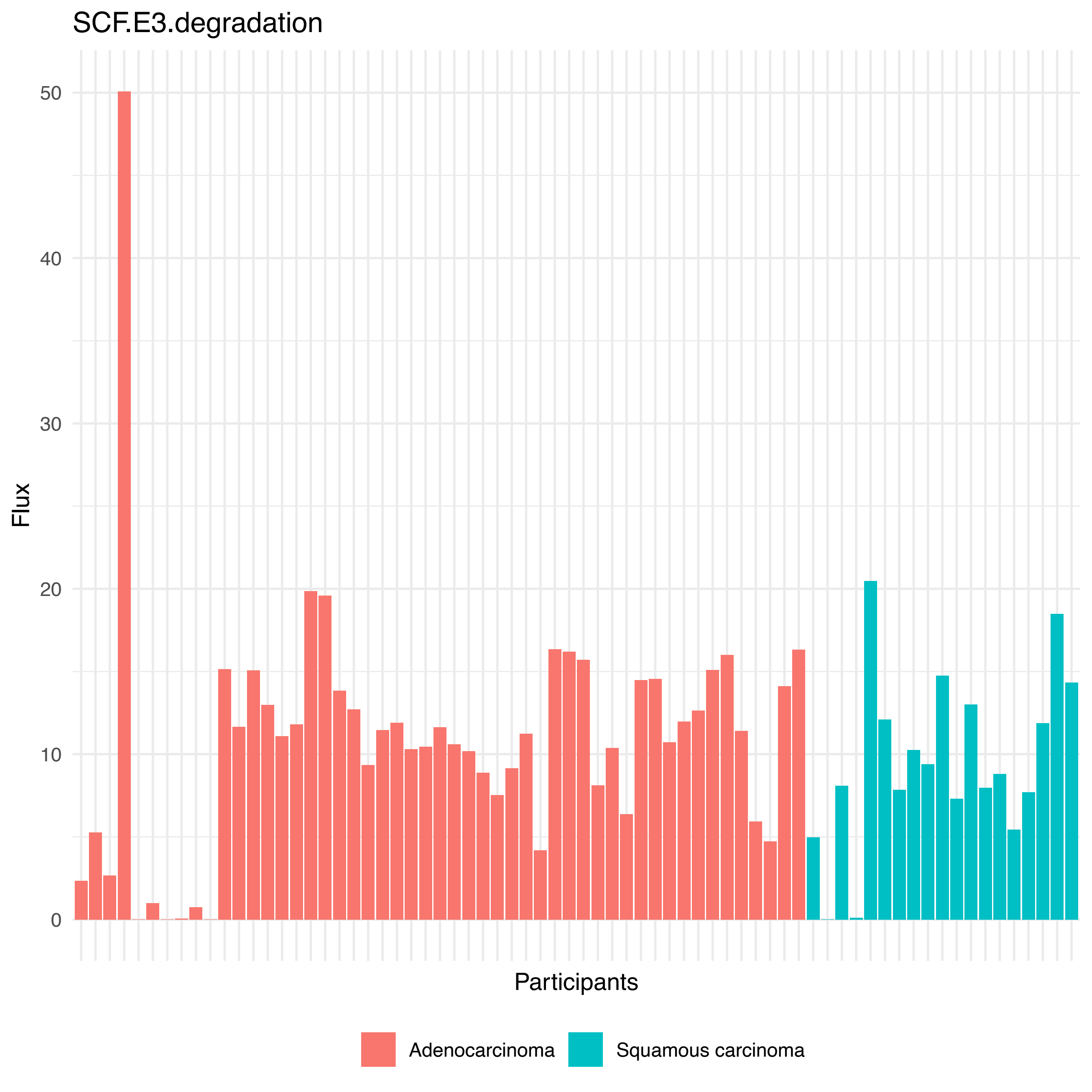

SCF.E3.degradation
Flux
50
40
30
20
10
0
Participants
Adenocarcinoma
Squamous carcinoma

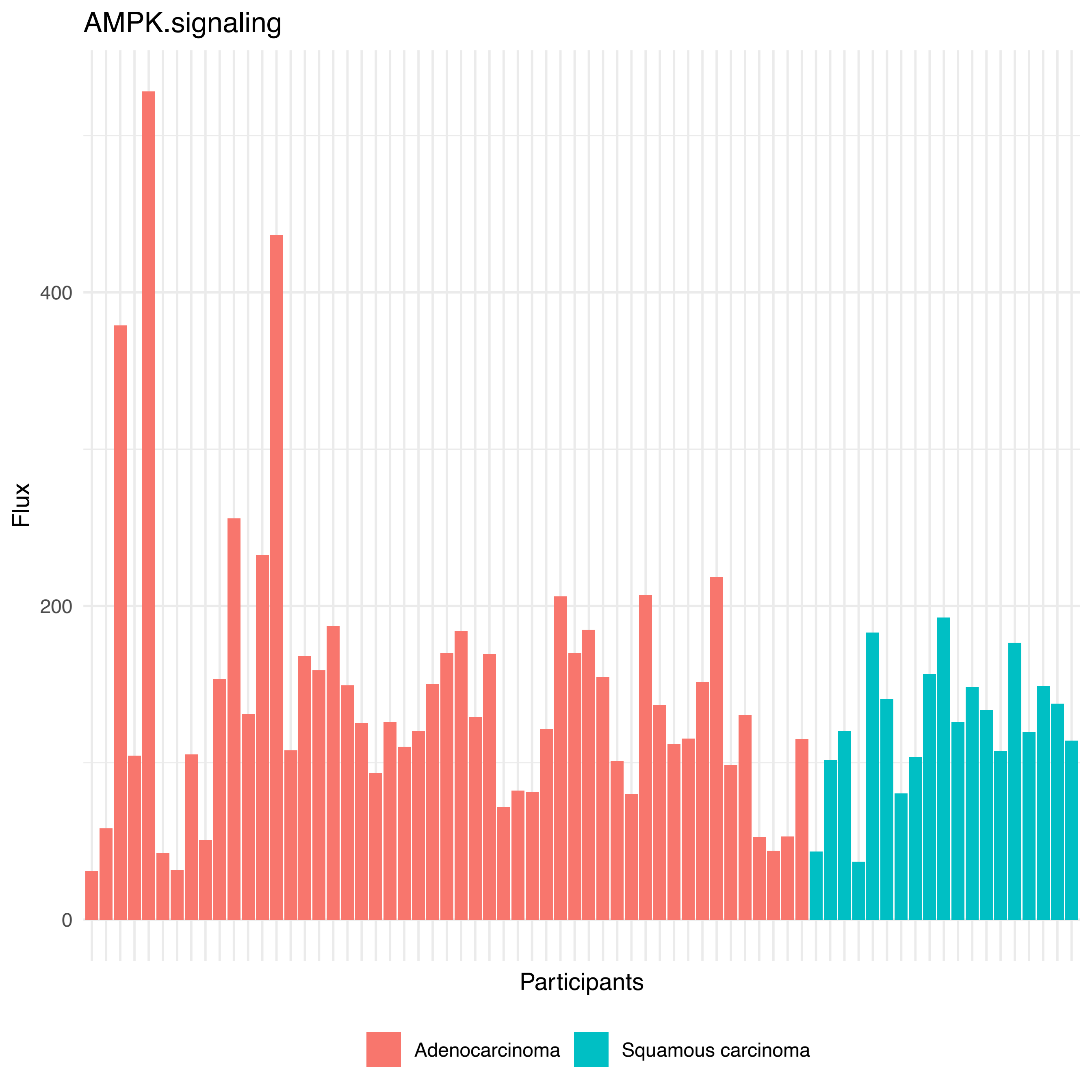

AMPK.signaling
Flux
400
200
0
Participants
Adenocarcinoma
Squamous carcinoma

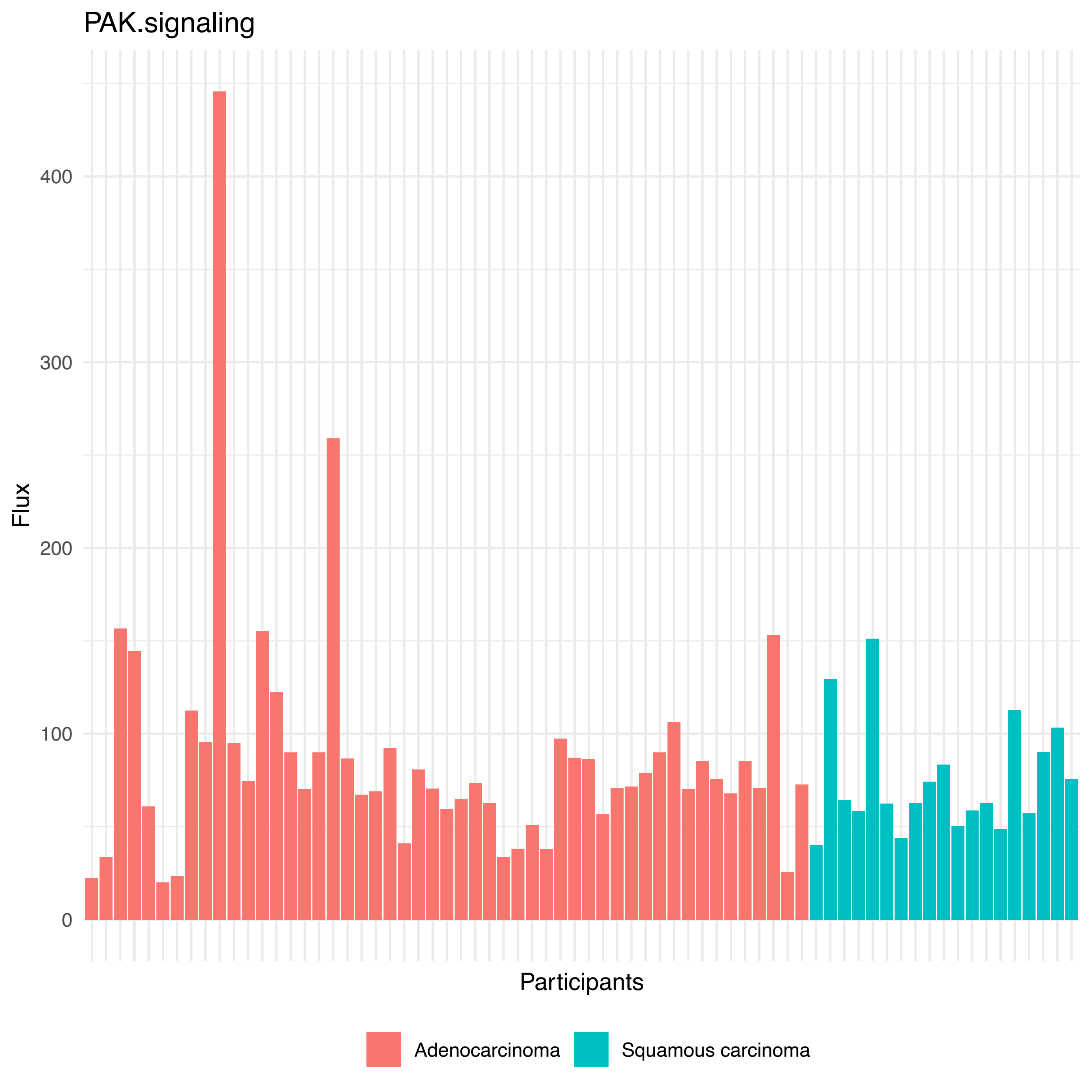

PAK.signaling
Flux
400
300
200
100
0
Participants
Adenocarcinoma
Squamous carcinoma

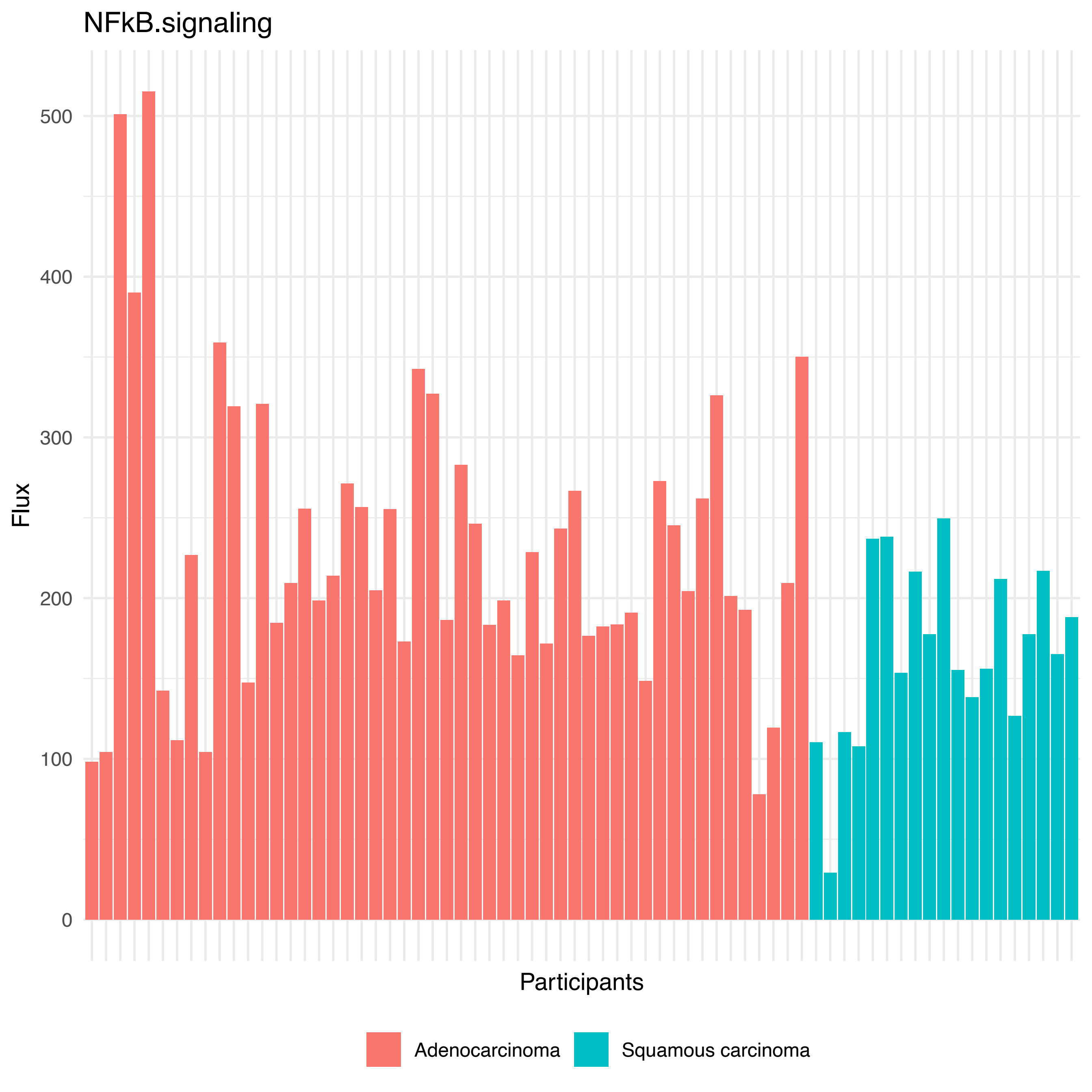
NFkB.signaling
Flux
500
400
300
200
100
0
Participants
Adenocarcinoma
Squamous carcinoma

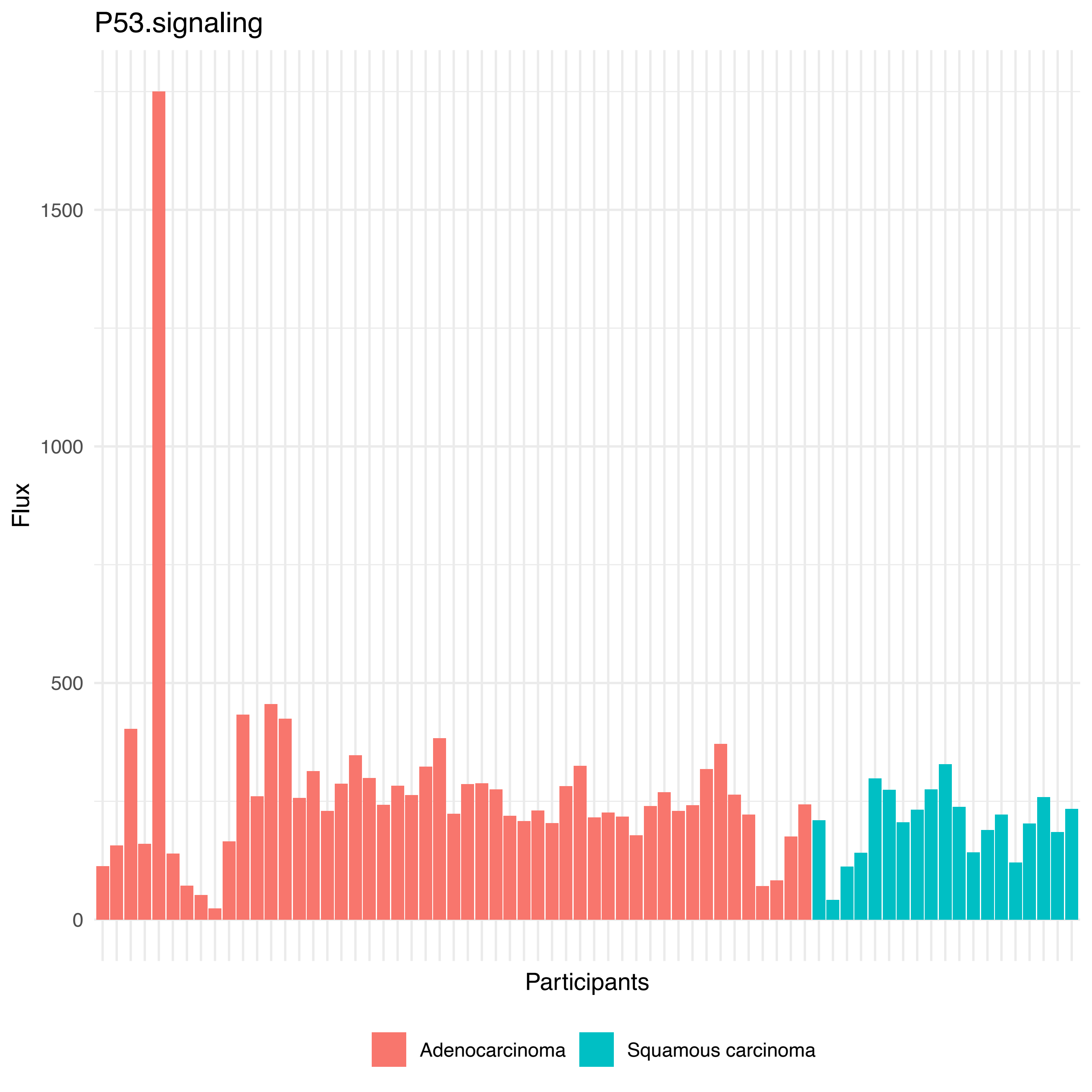

P53.signaling
Flux
1500
1000
500
0
Participants
Adenocarcinoma
Squamous carcinoma

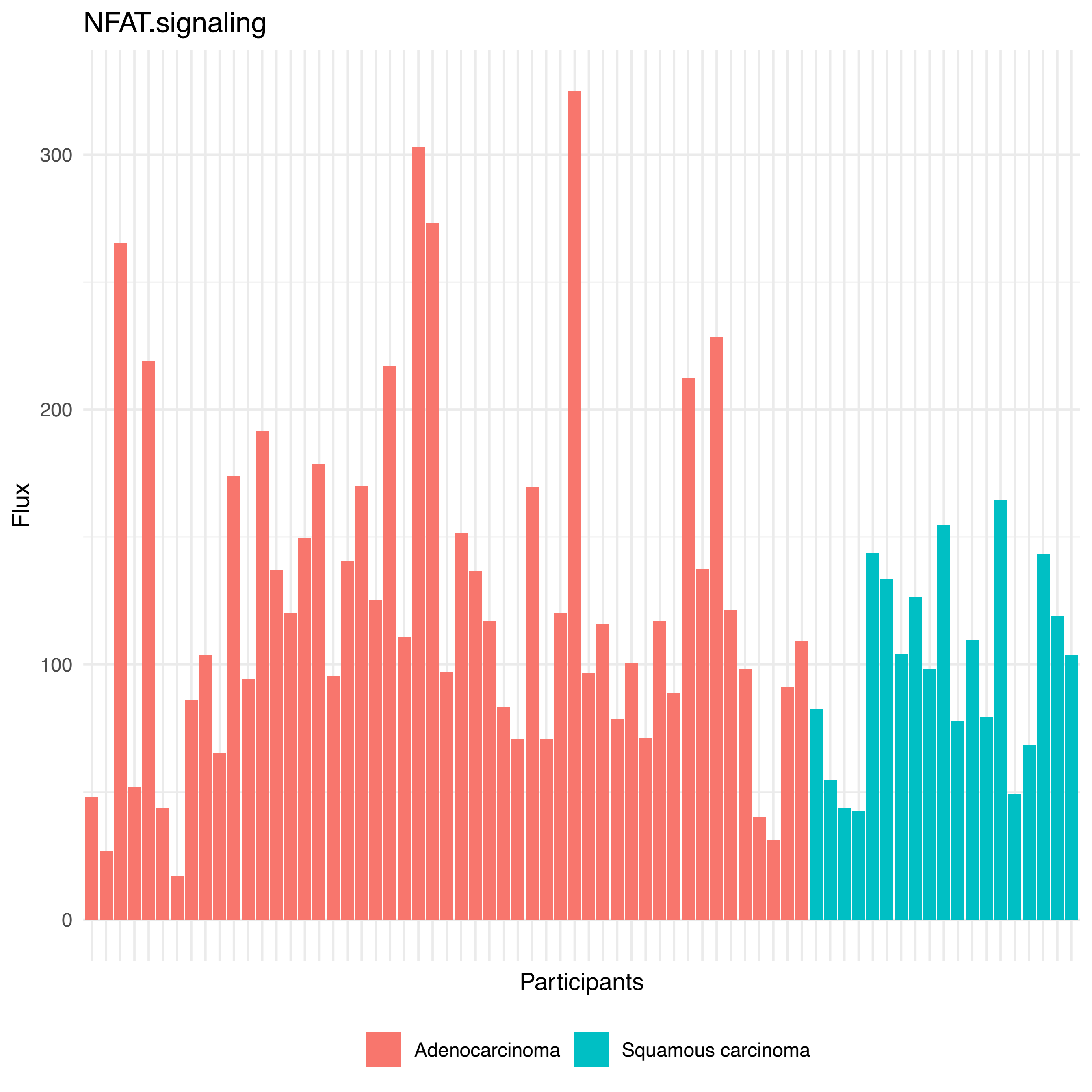

NFAT.signaling
300
200
100
0
Flux
Participants
Adenocarcinoma
Squamous carcinoma

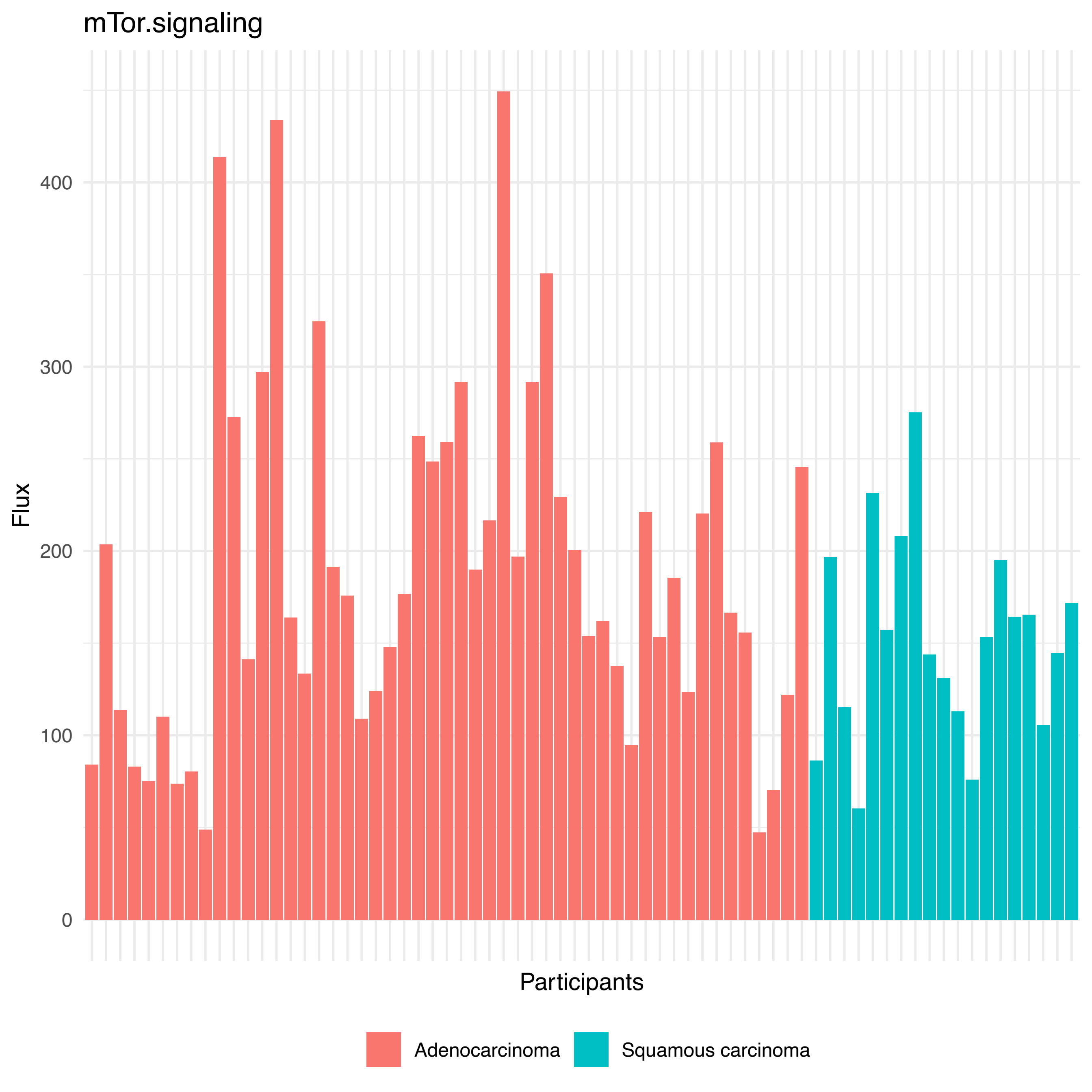

mTor.signaling
Flux
400
300
200
100
0
Participants
Adenocarcinoma
Squamous carcinoma

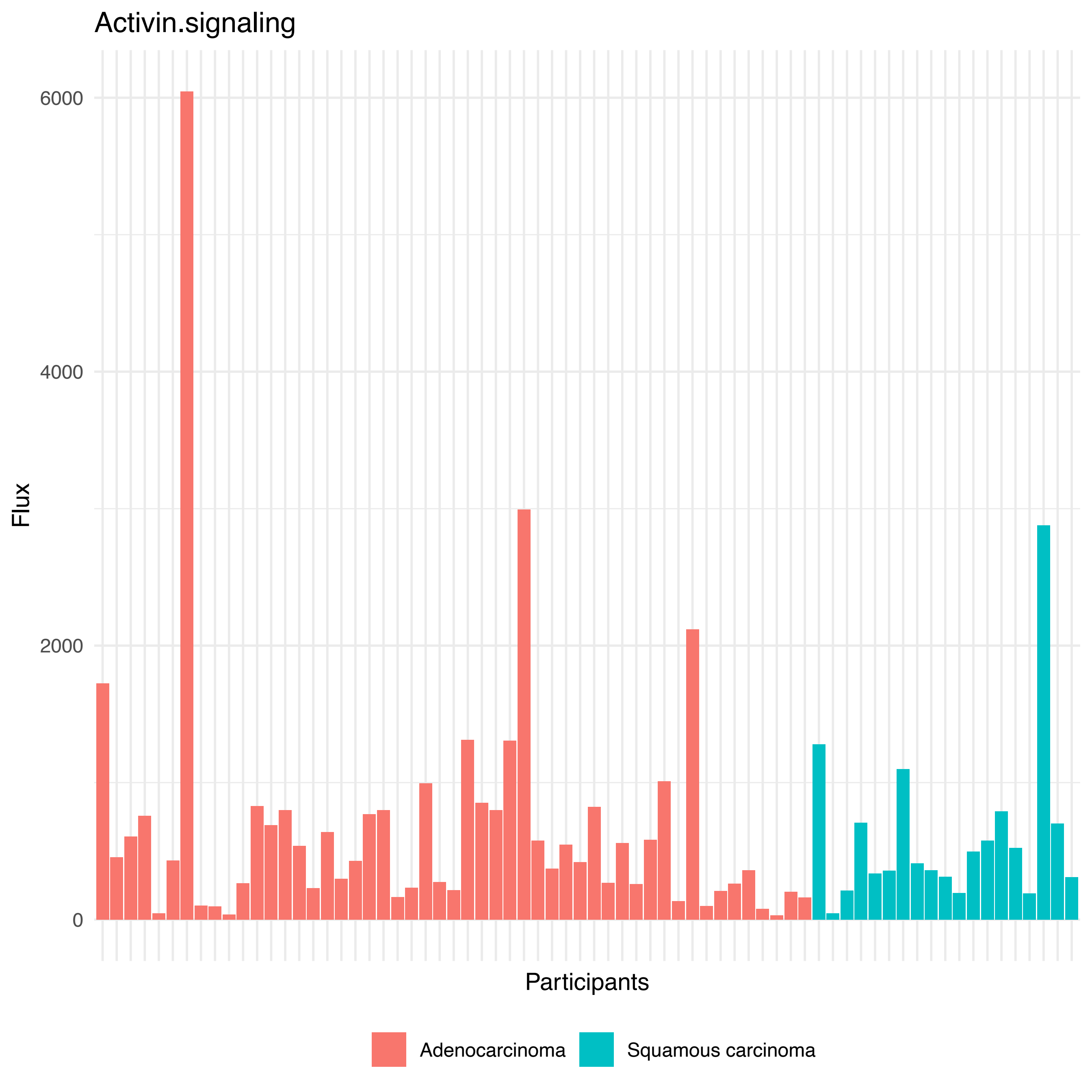
Activin.signaling
6000
4000
2000
0
Flux
Participants
Adenocarcinoma
Squamous carcinoma

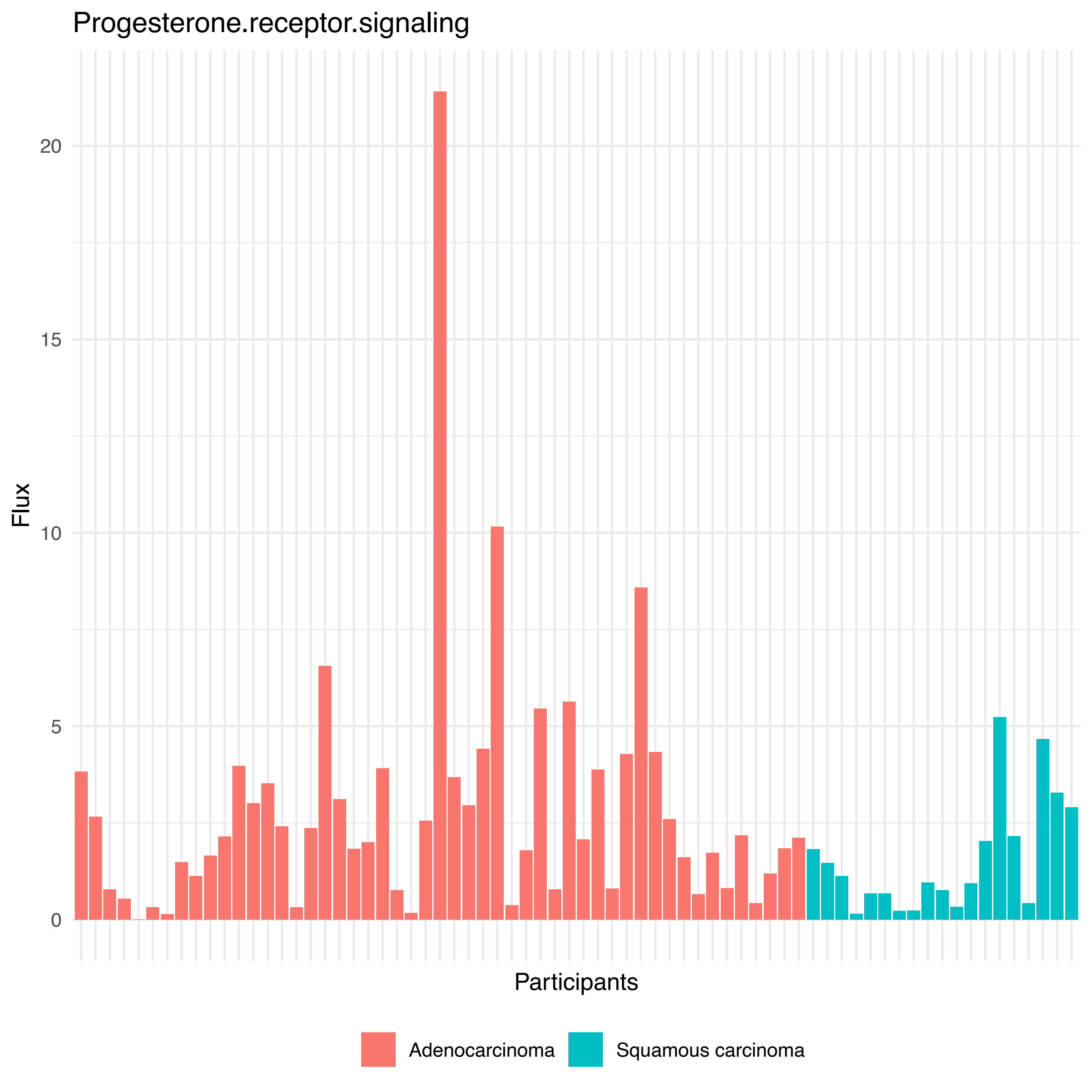

Progesterone.receptor.signaling
Flux
20
15
10
5
0
Participants
Adenocarcinoma
Squamous carcinoma

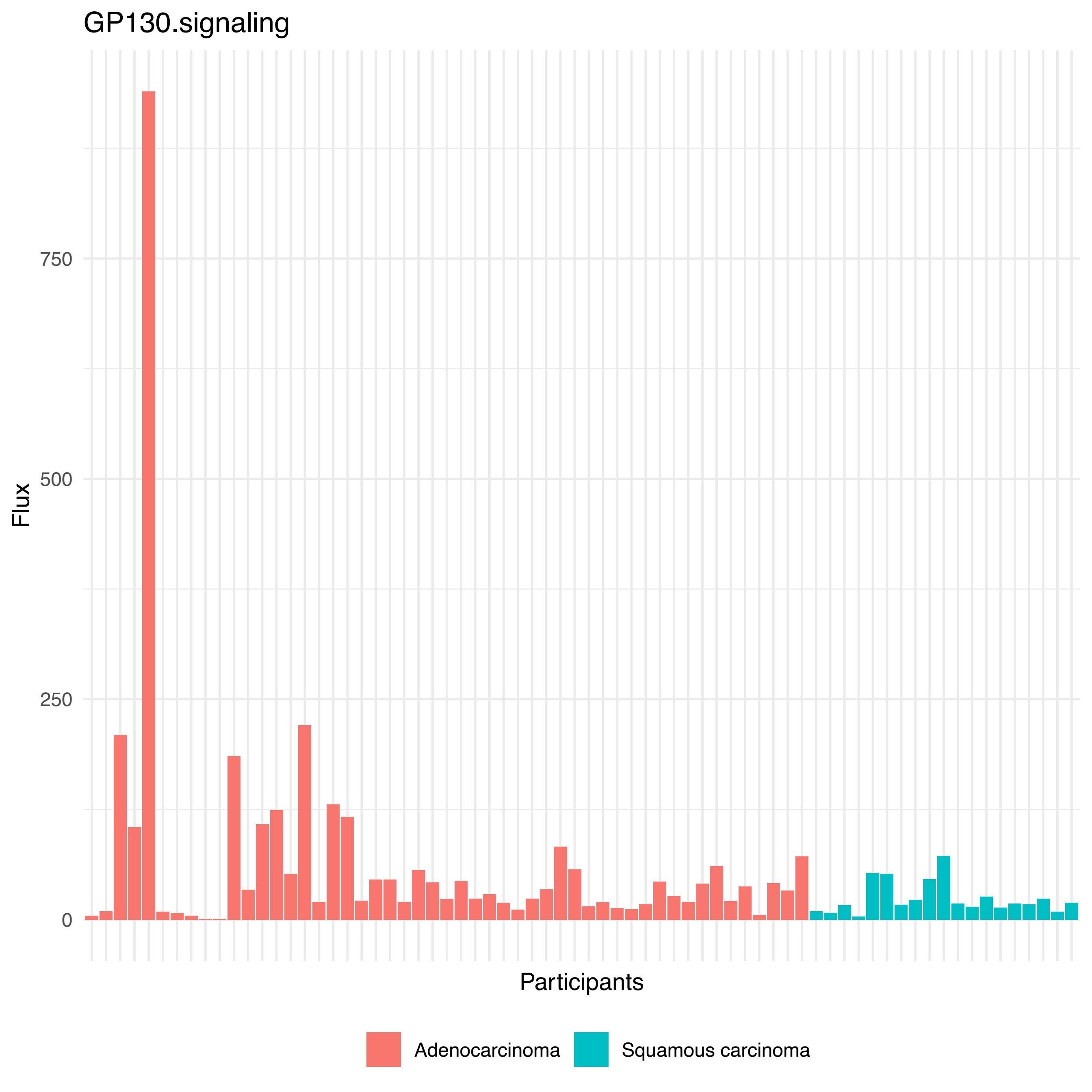
GP130.signaling
Flux
750
500
250
0
Participants
Adenocarcinoma
Squamous carcinoma

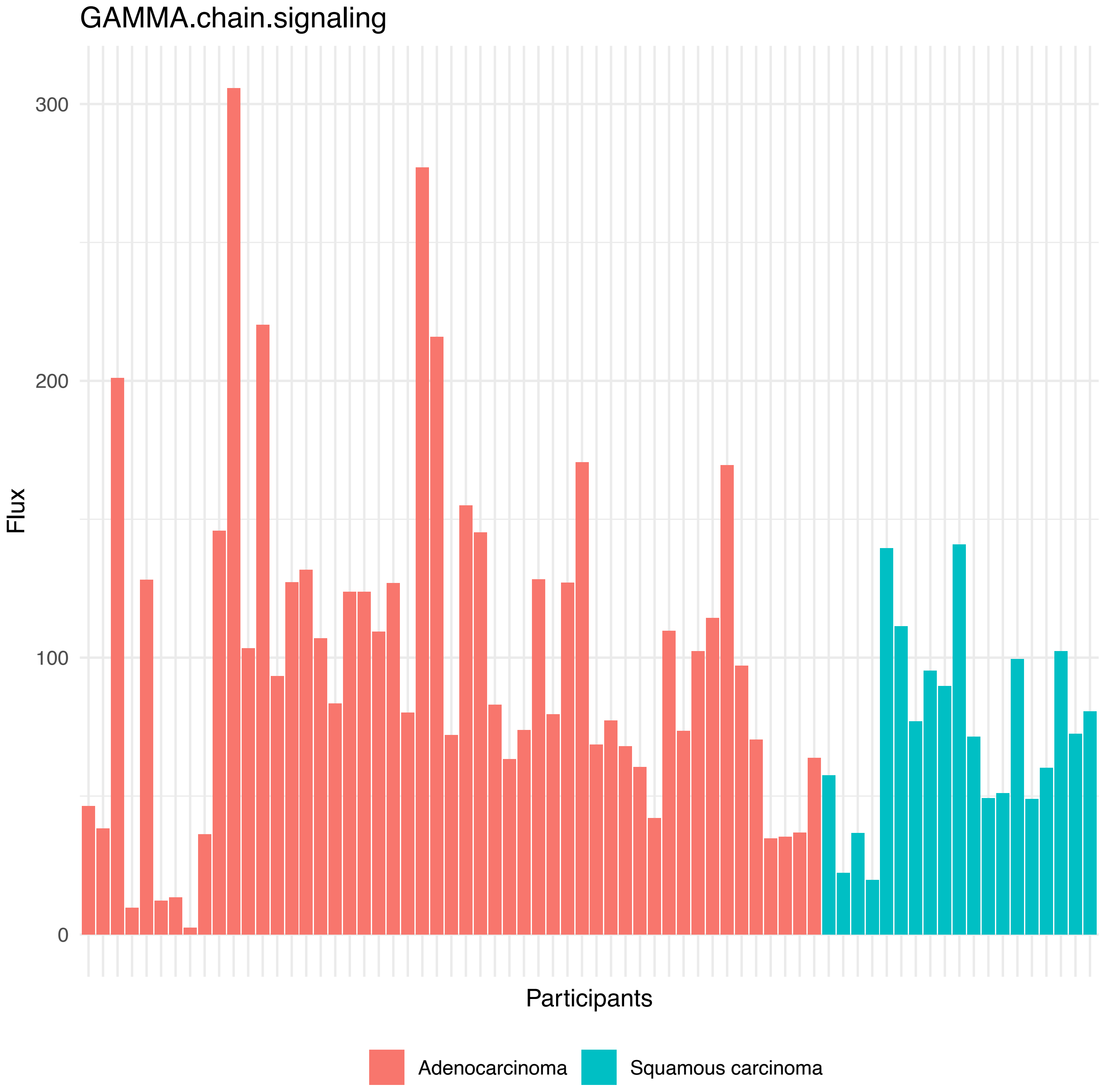
GAMMA.chain.signaling
Flux
300
200
100
0
Participants
Adenocarcinoma
Squamous carcinoma

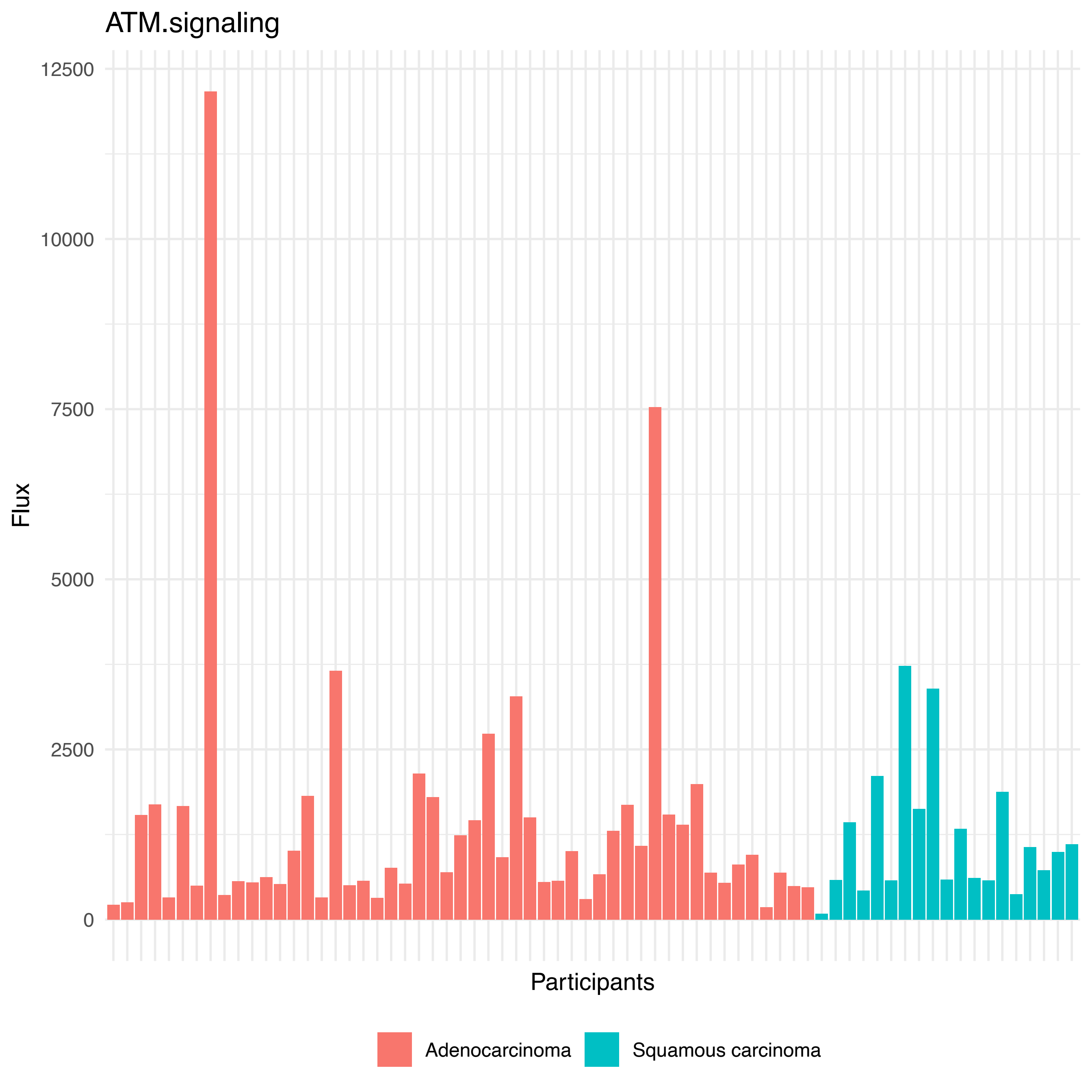

ATM.signaling
12500
10000
7500
5000
2500
0
Flux
Participants
Adenocarcinoma
Squamous carcinoma

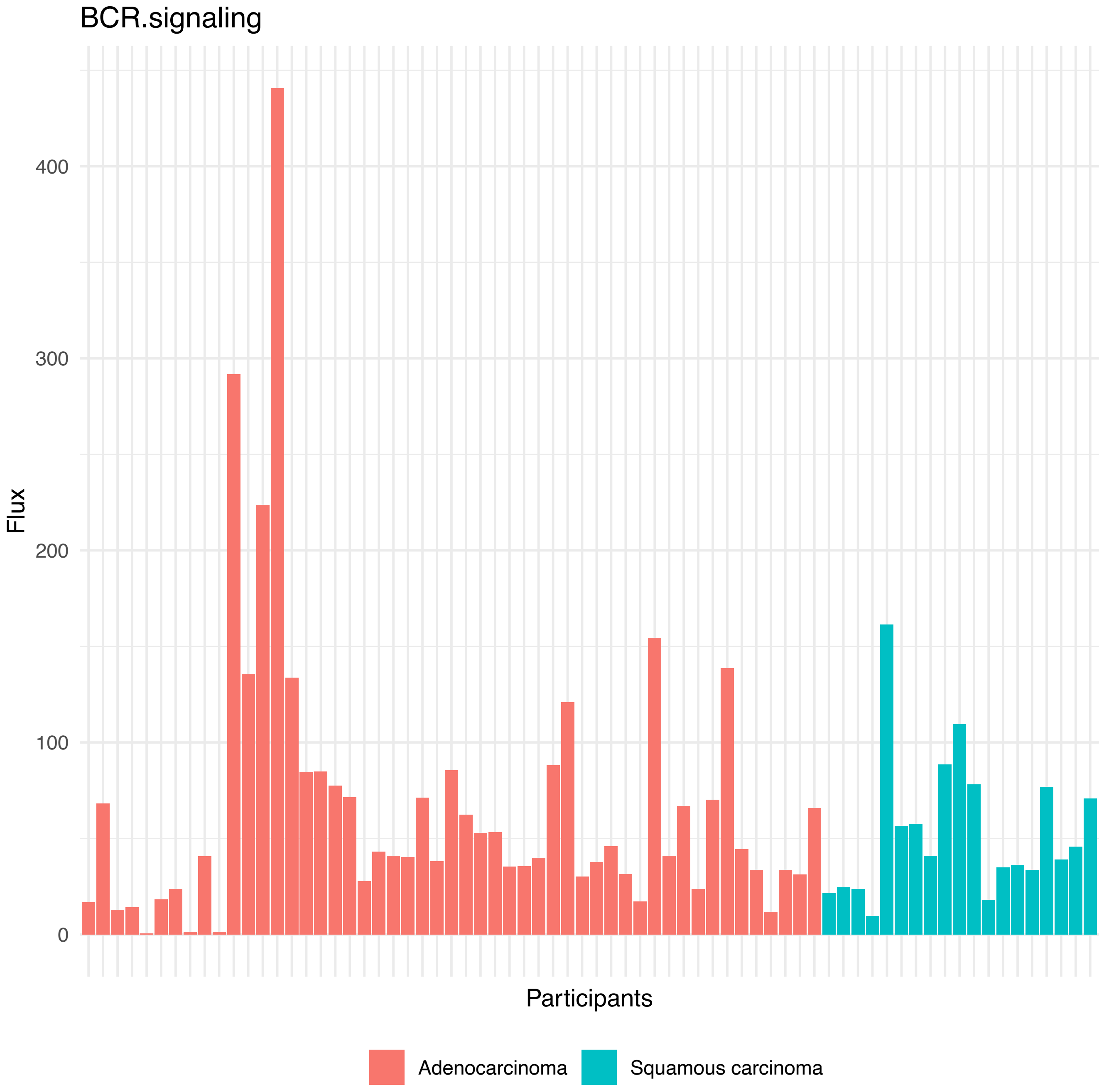
BCR.signaling
Flux
400
300
200
100
0
Participants
Adenocarcinoma
Squamous carcinoma

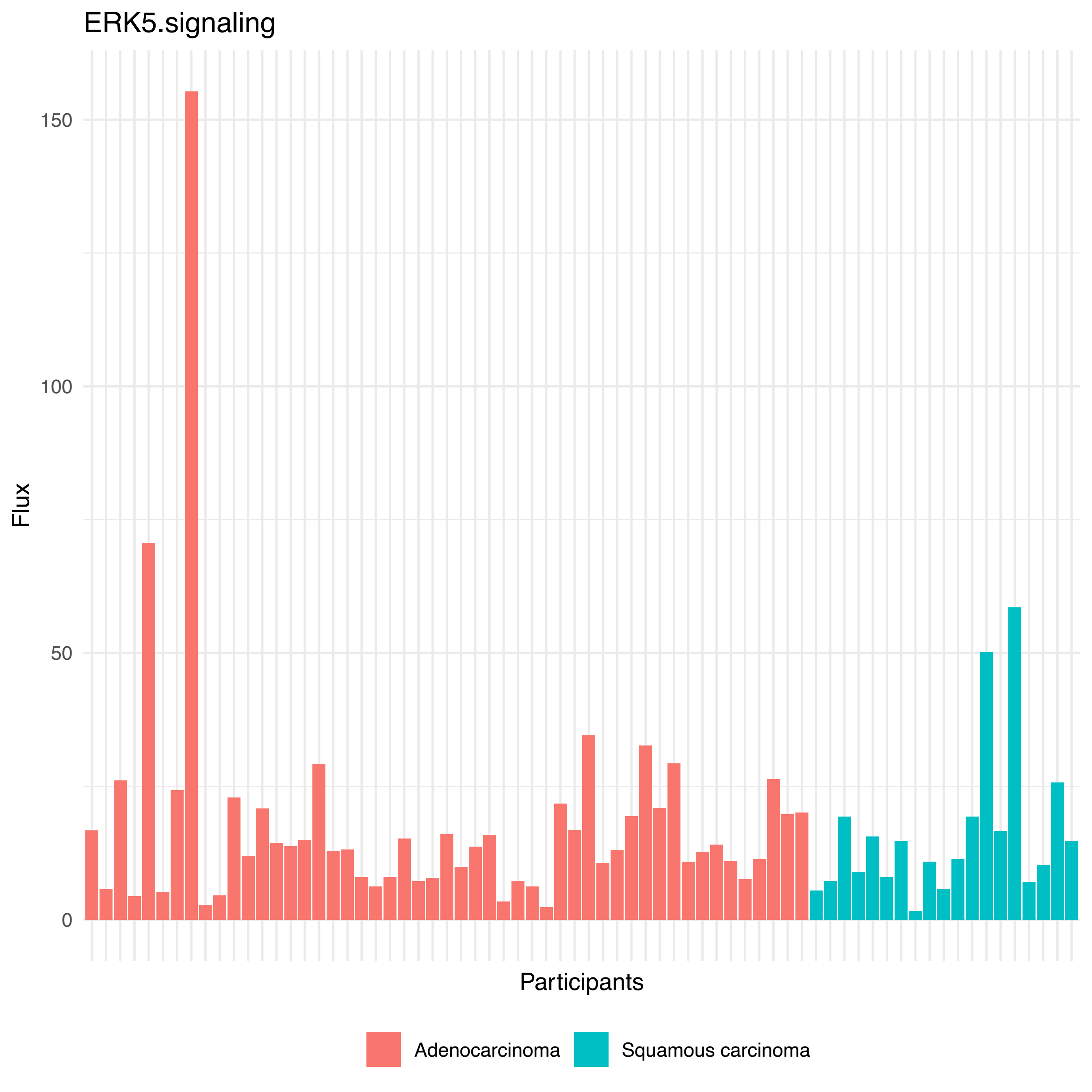
ERK5.signaling
150
100
50
0
Flux
Participants
Adenocarcinoma
Squamous carcinoma

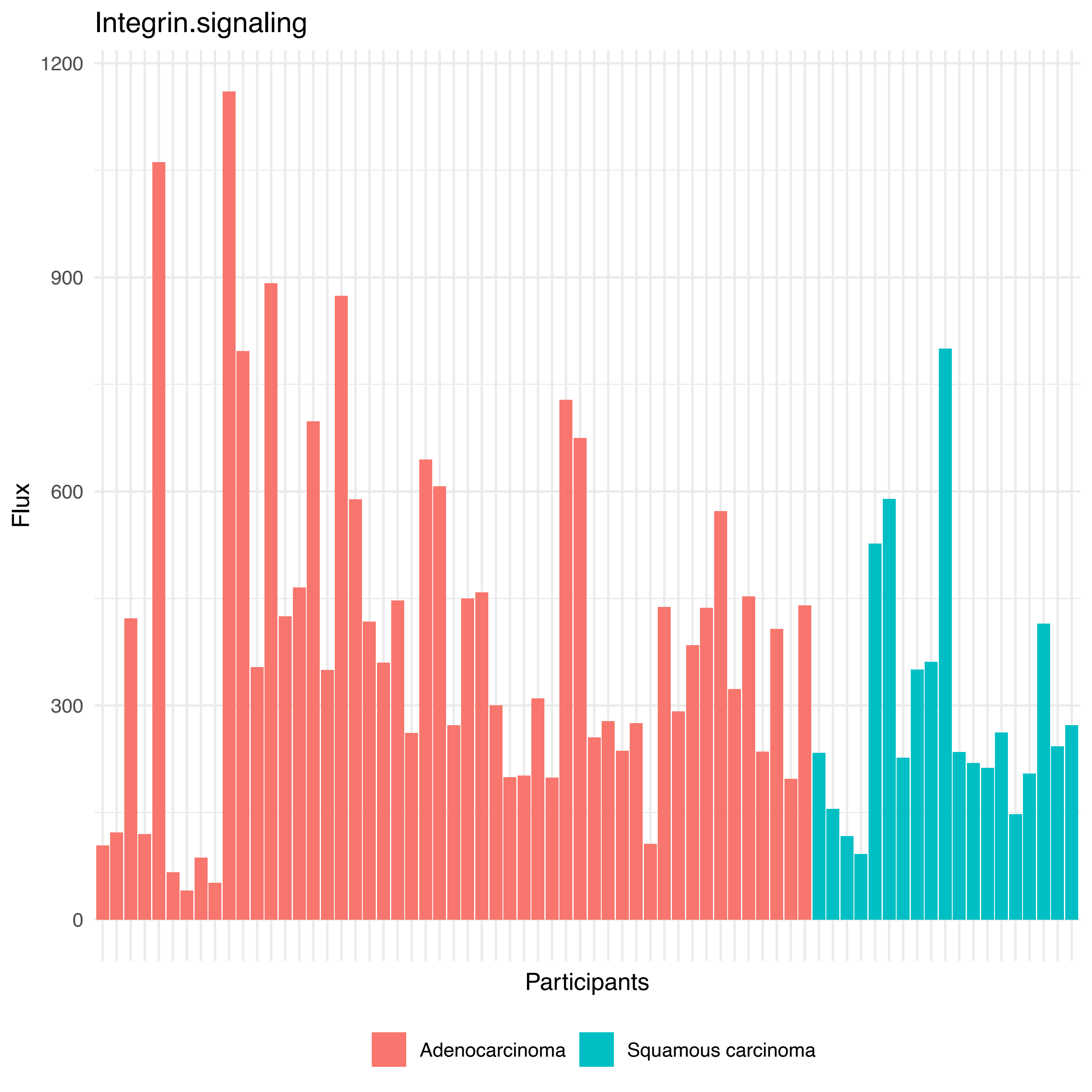
Integrin.signaling
1200
900
600
300
0
Flux
Participants
Adenocarcinoma
Squamous carcinoma

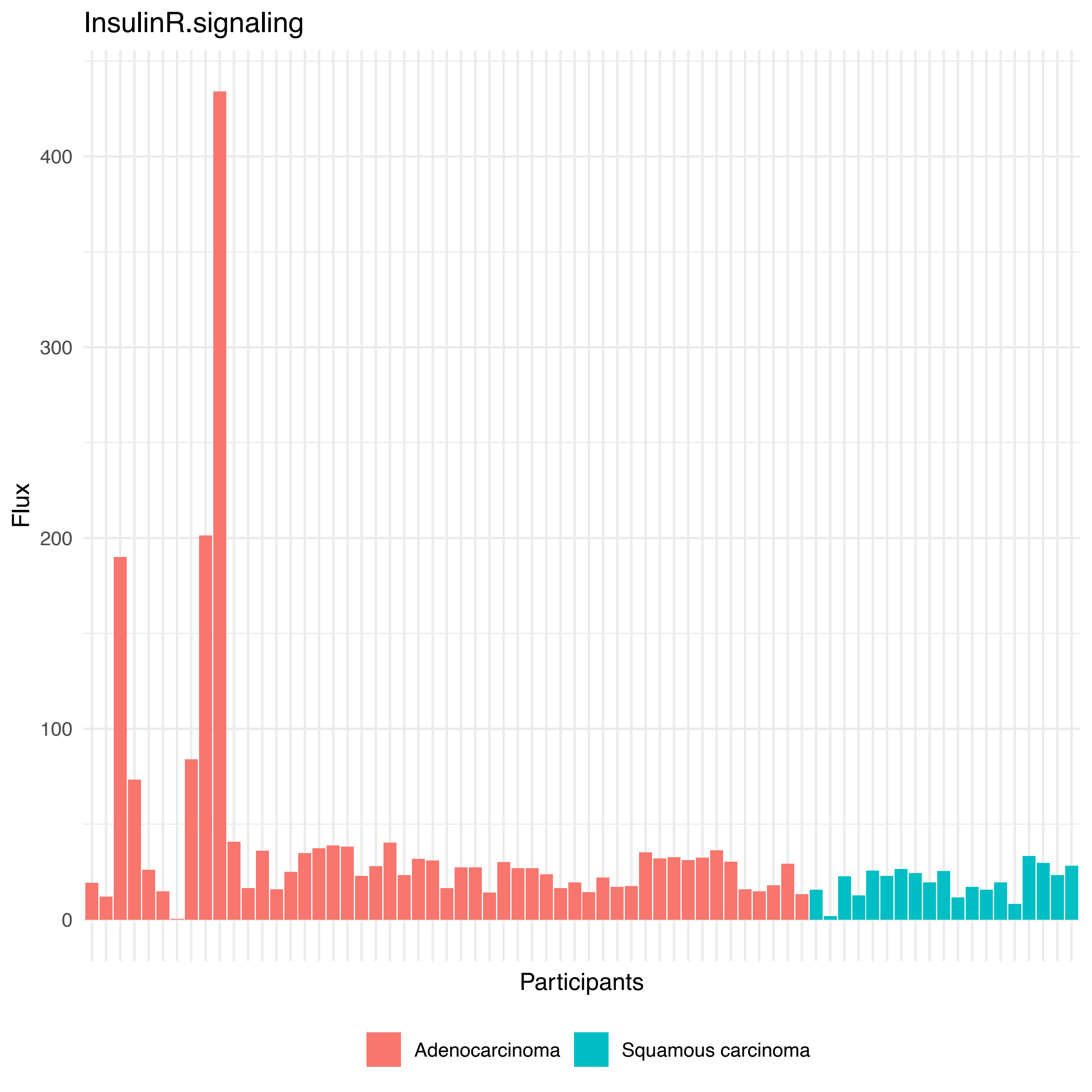

InsulinR.signaling
Flux
400
300
200
100
0
Participants
Adenocarcinoma
Squamous carcinoma

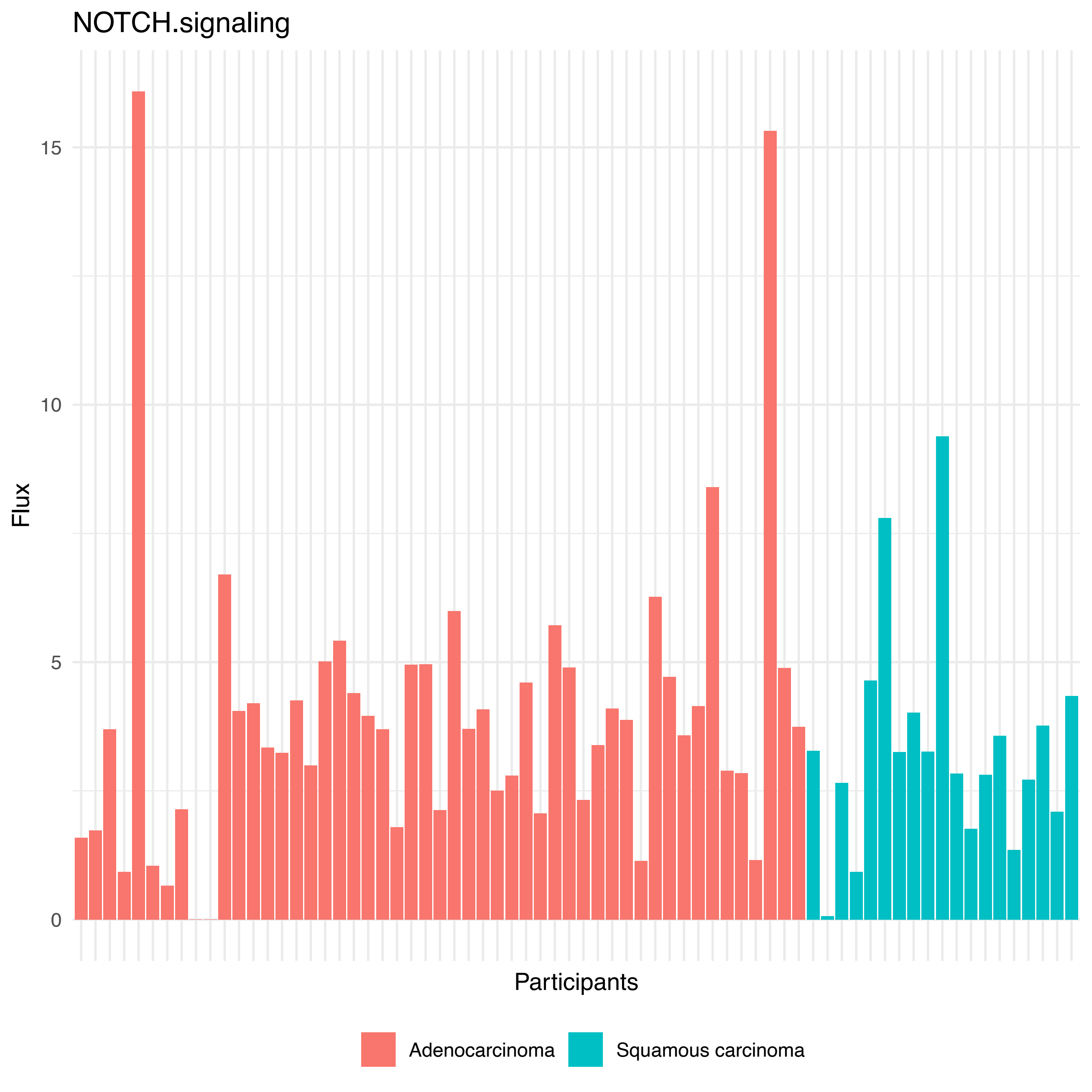
NOTCH.signaling
Flux
15
10
5
0
Participants
Adenocarcinoma
Squamous carcinoma

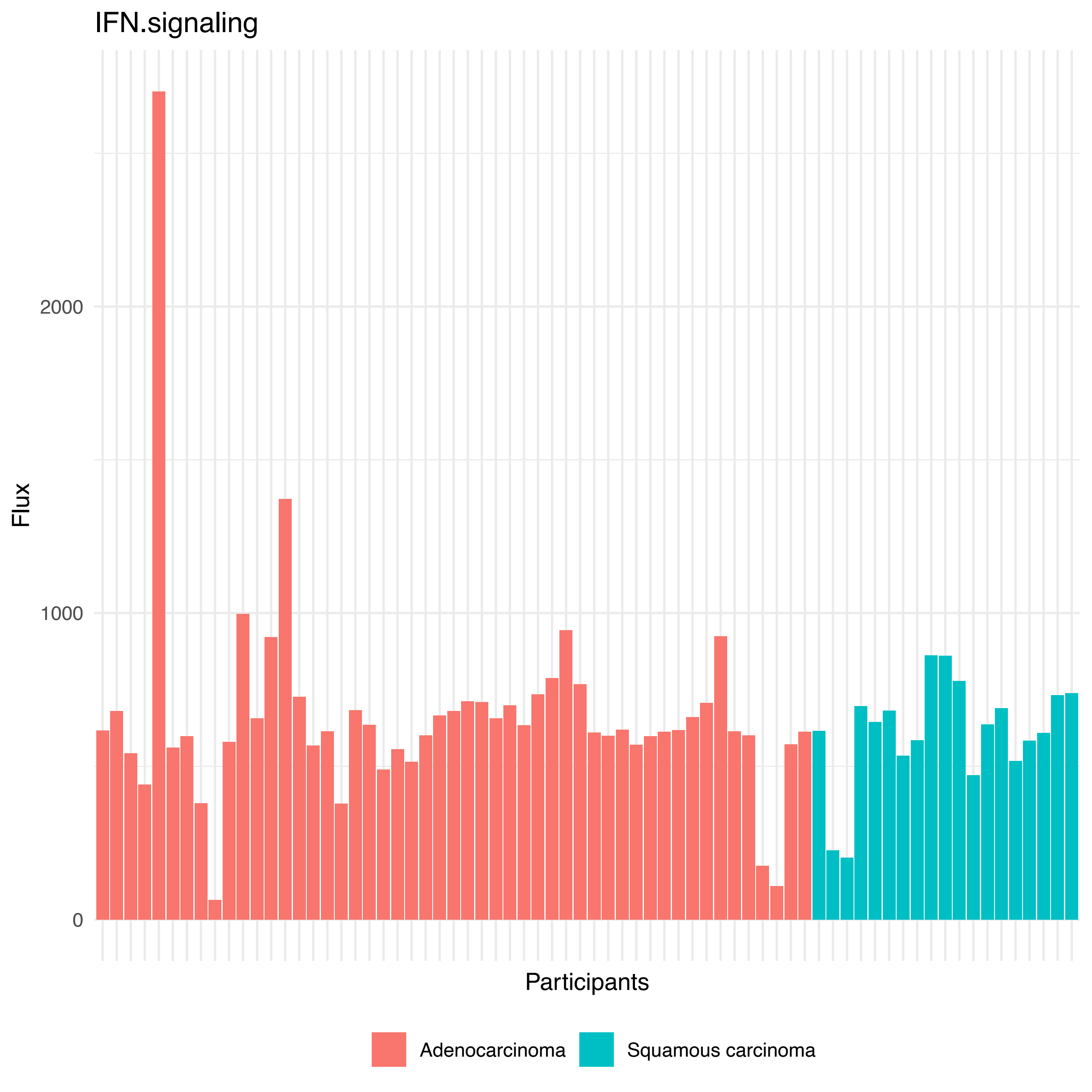
IFN.signaling
Flux
2000
1000
0
Participants
Adenocarcinoma
Squamous carcinoma

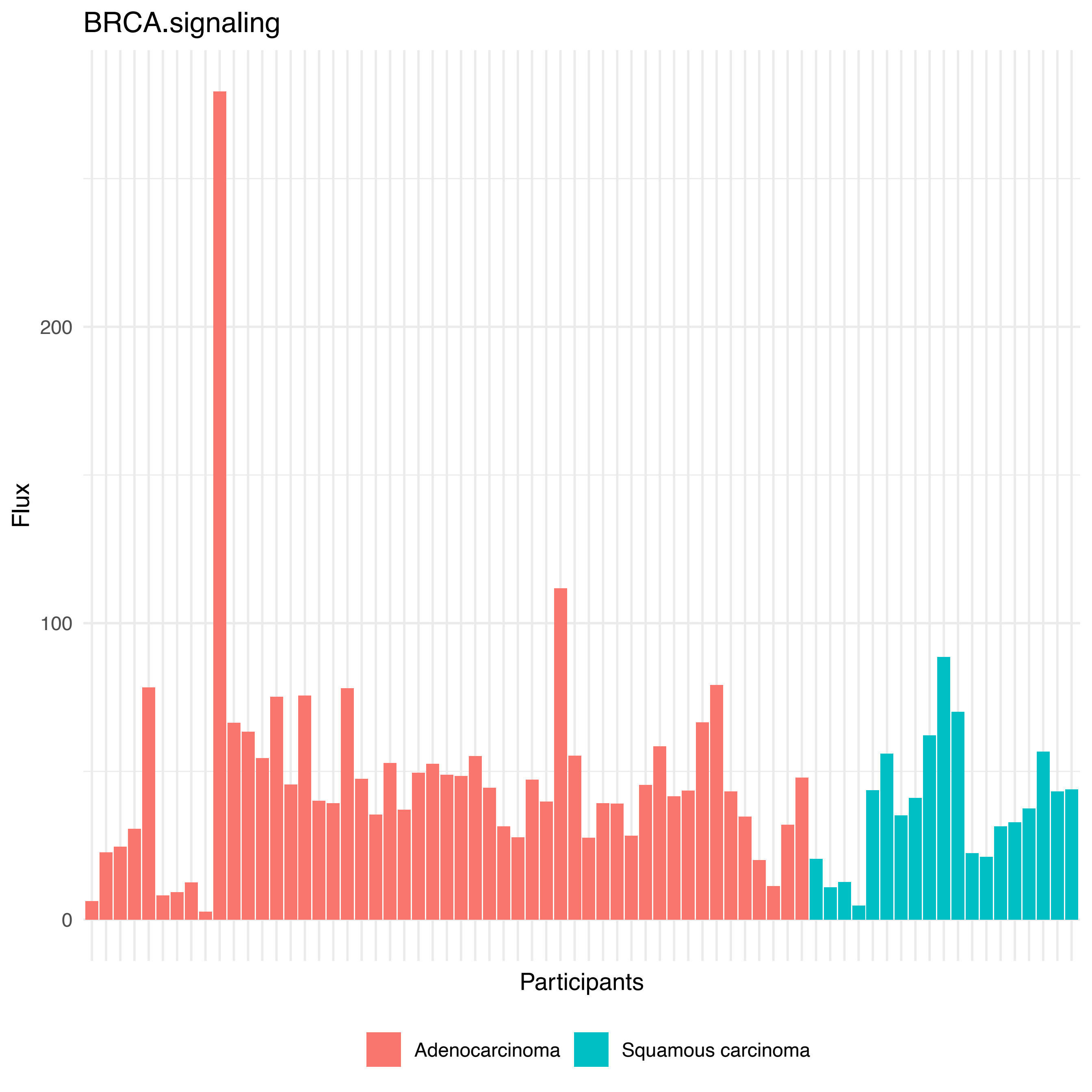

BRCA.signaling
Flux
200
100
0
Participants
Adenocarcinoma
Squamous carcinoma

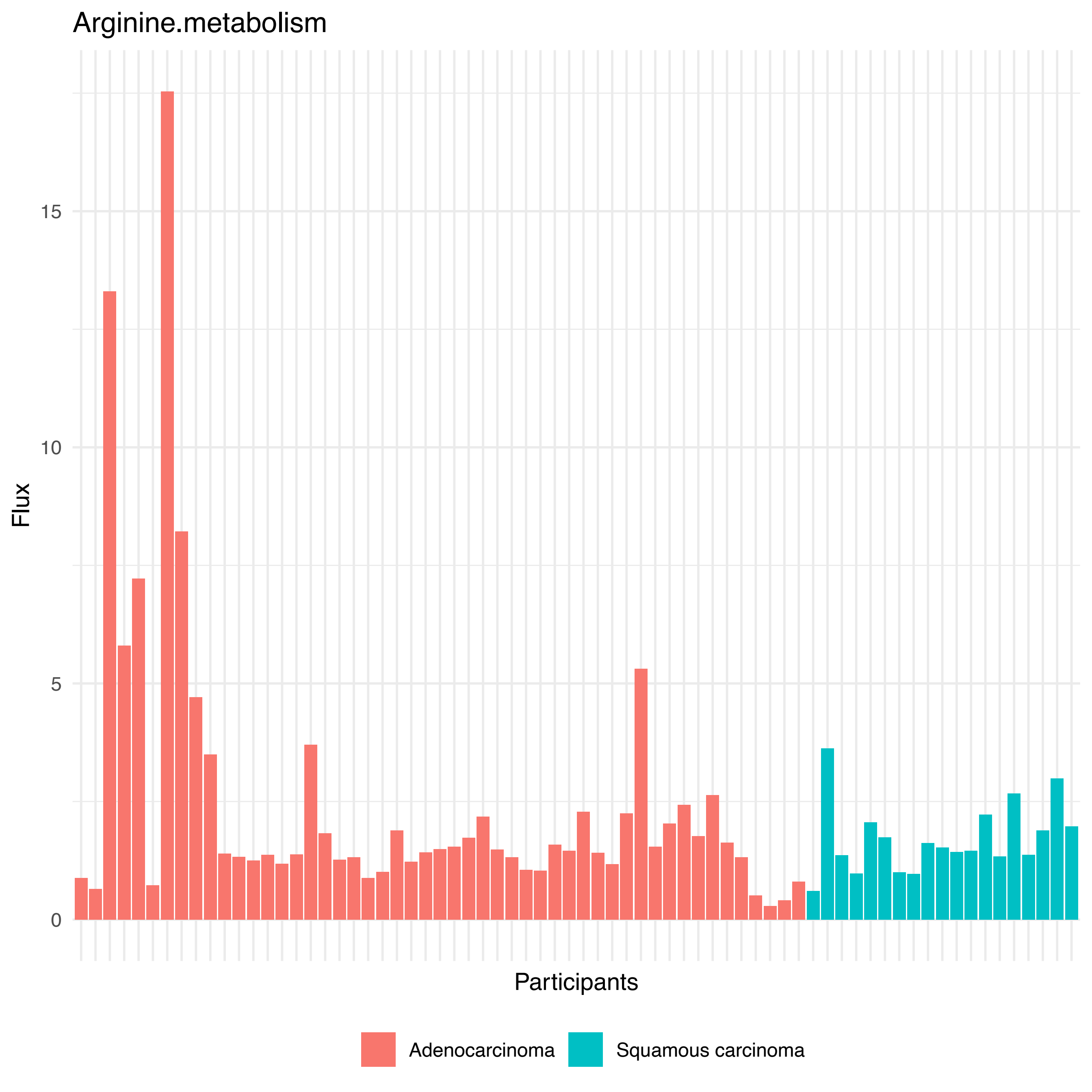

Arginine.metabolism
Flux
15
10
5
0
Participants
Adenocarcinoma
Squamous carcinoma

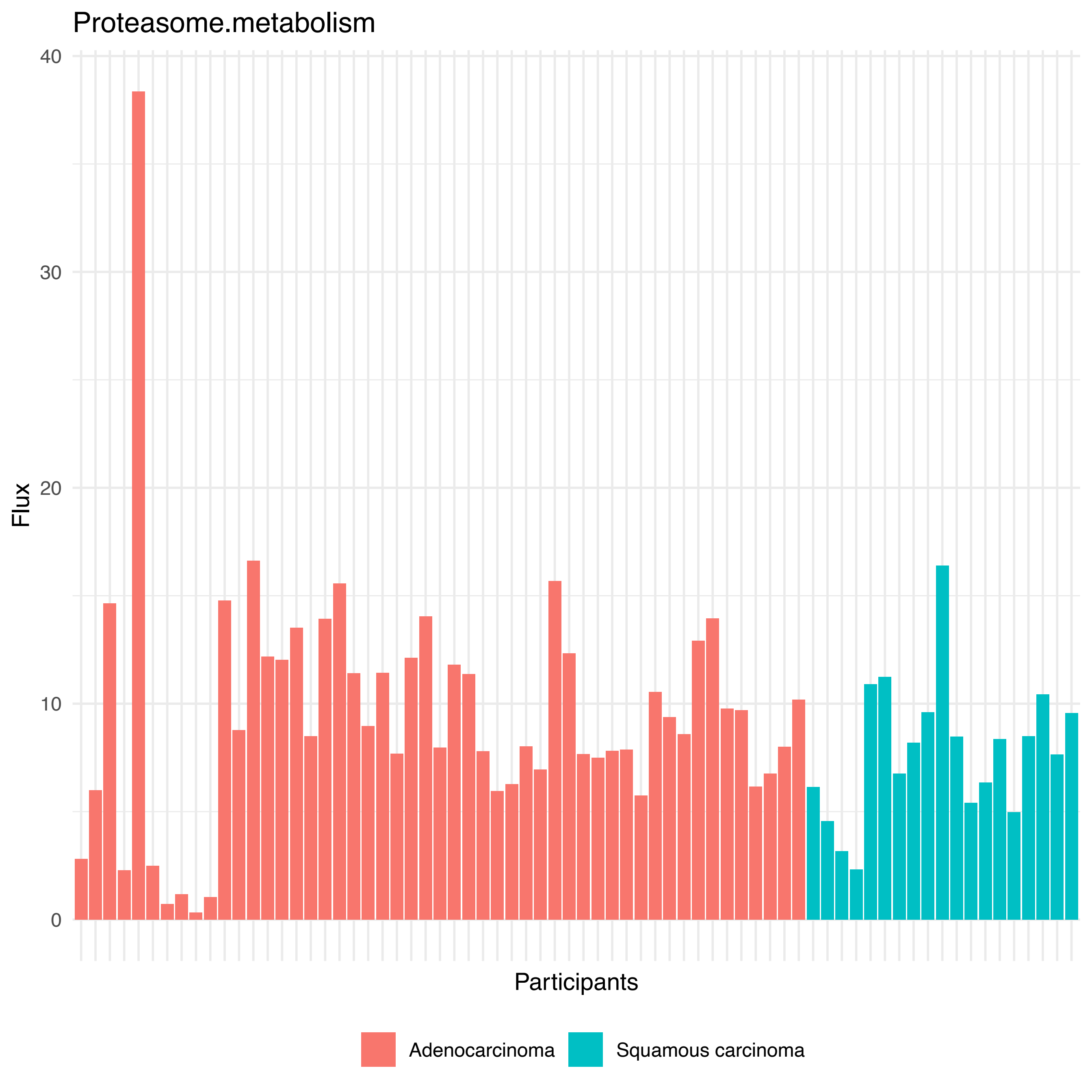
Proteasome.metabolism
40
30
20
10
0
Flux
Participants
Adenocarcinoma
Squamous carcinoma

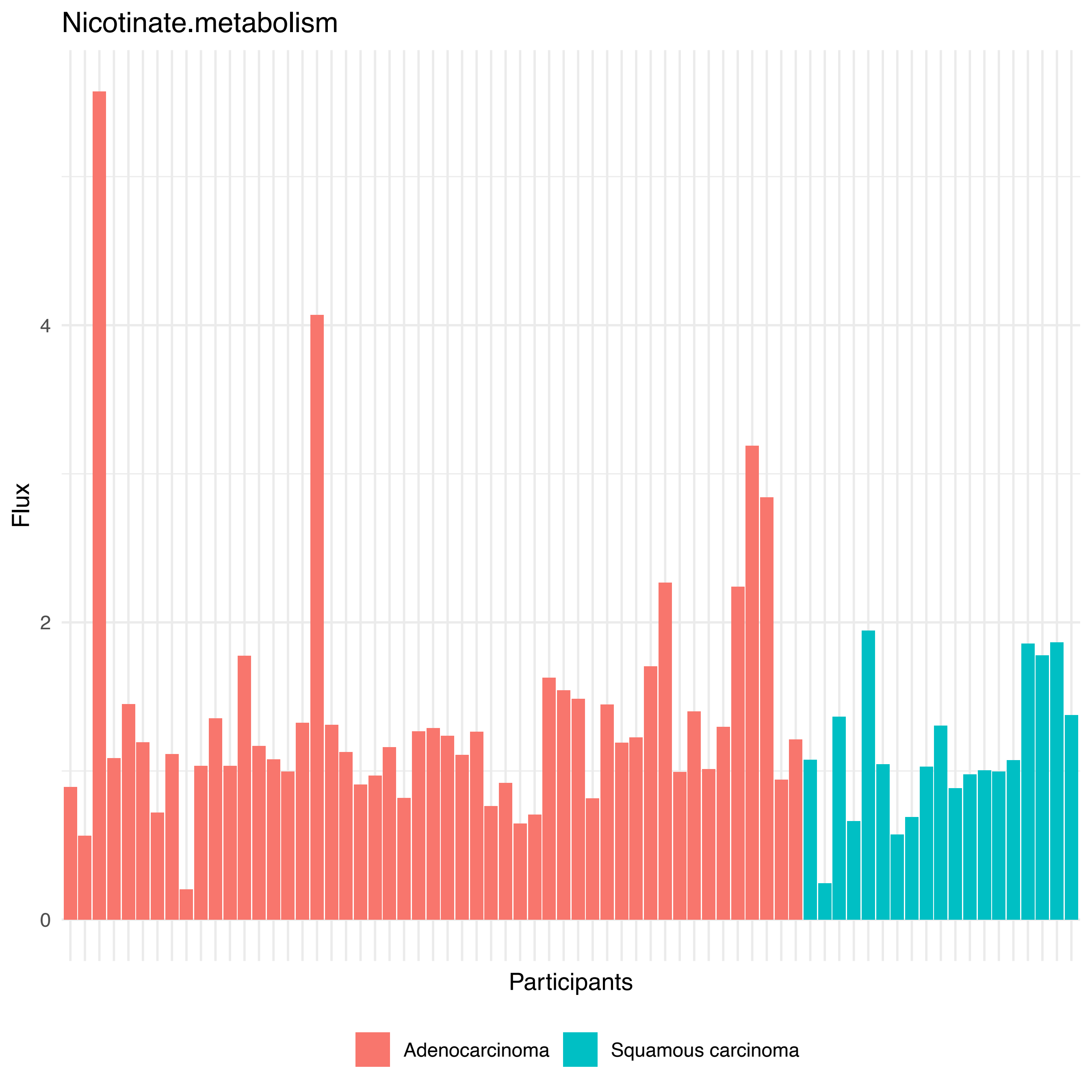

Nicotinate.metabolism
Flux
0
2
4
Participants
Adenocarcinoma
Squamous carcinoma

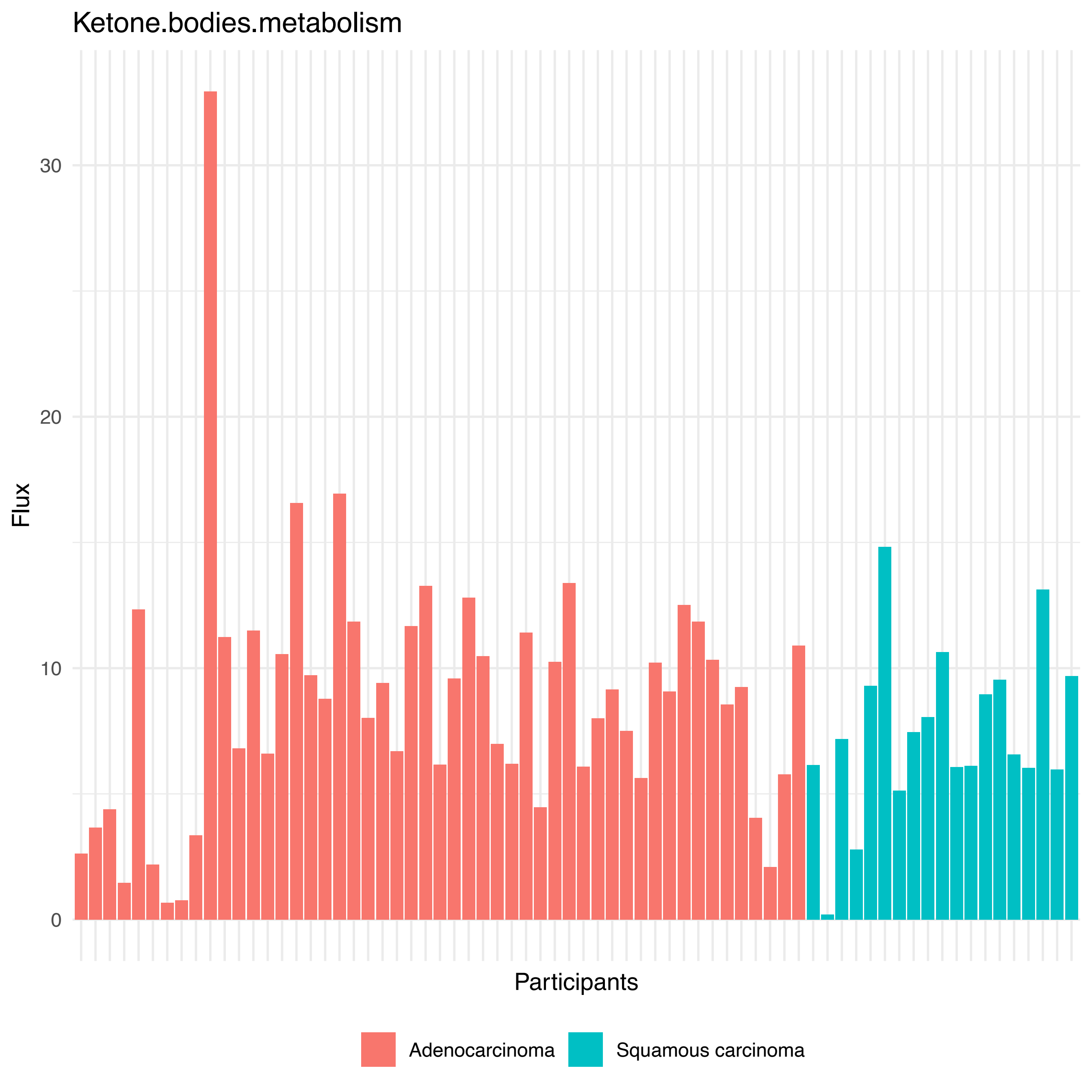

Ketone.bodies.metabolism
Flux
30
20
10
0
Participants
Adenocarcinoma
Squamous carcinoma

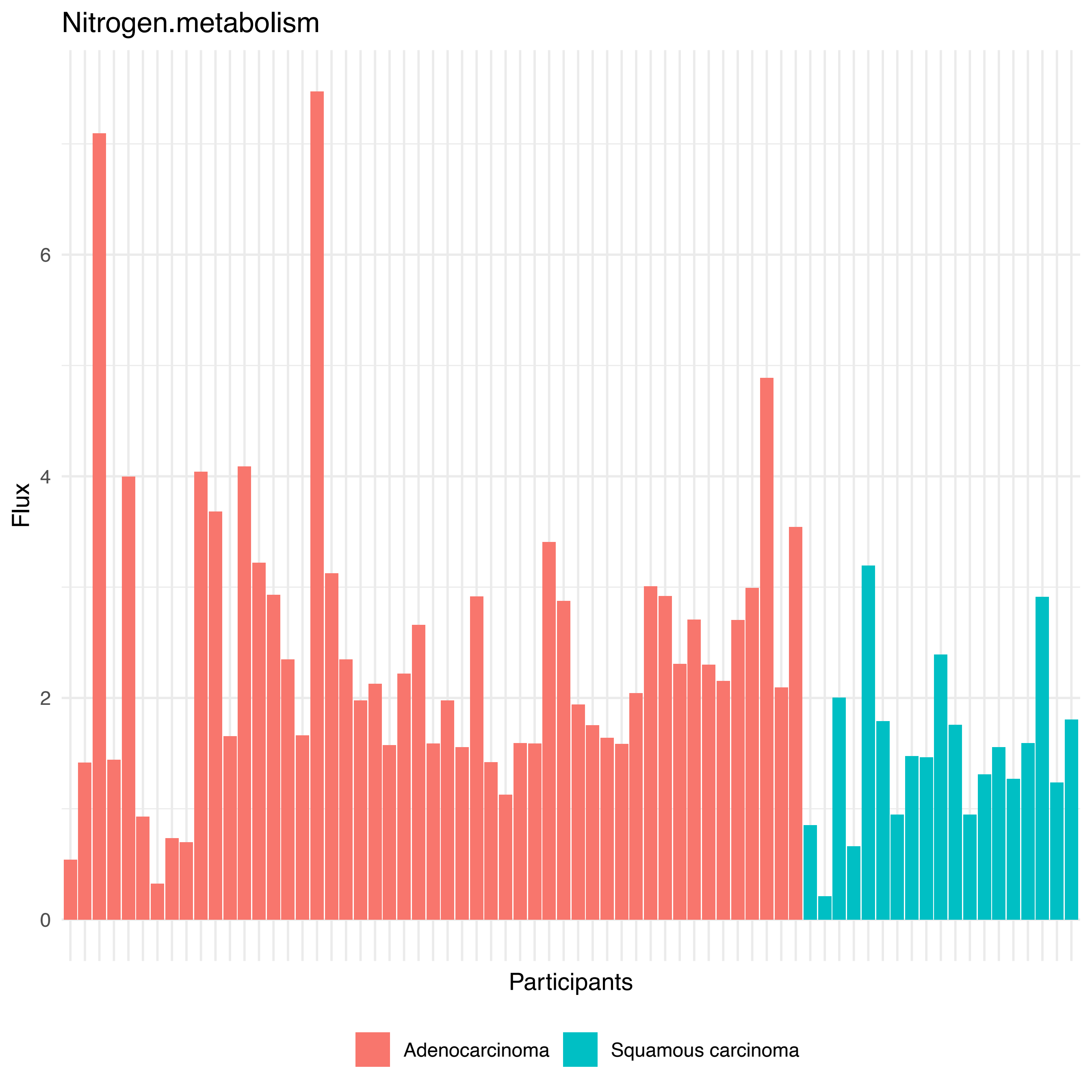

Nitrogen.metabolism
Flux
6
4
2
0
Participants
Adenocarcinoma
Squamous carcinoma

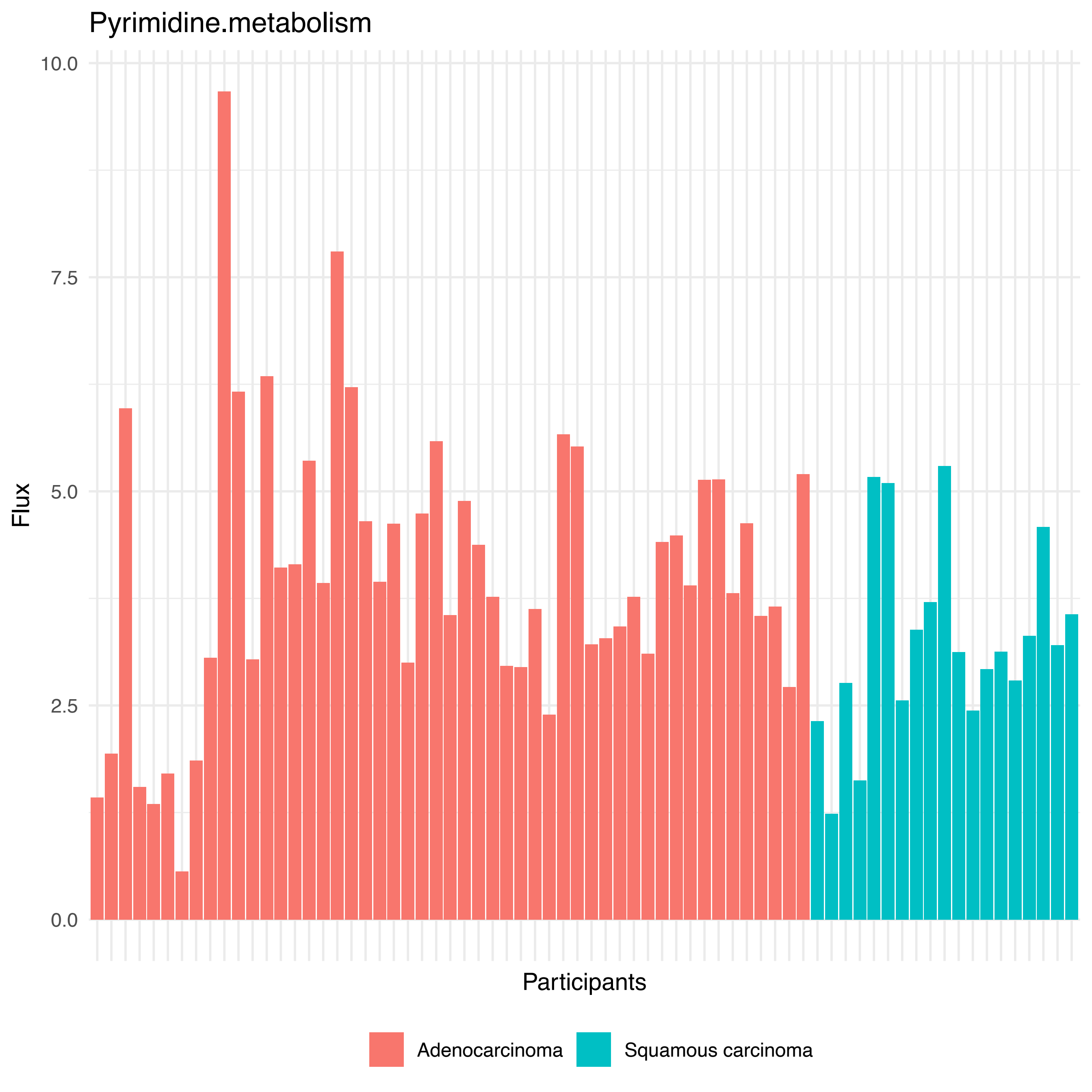

Pyrimidine.metabolism
Flux
10.0
7.5
5.0
2.5
0.0
Participants
Adenocarcinoma
Squamous carcinoma

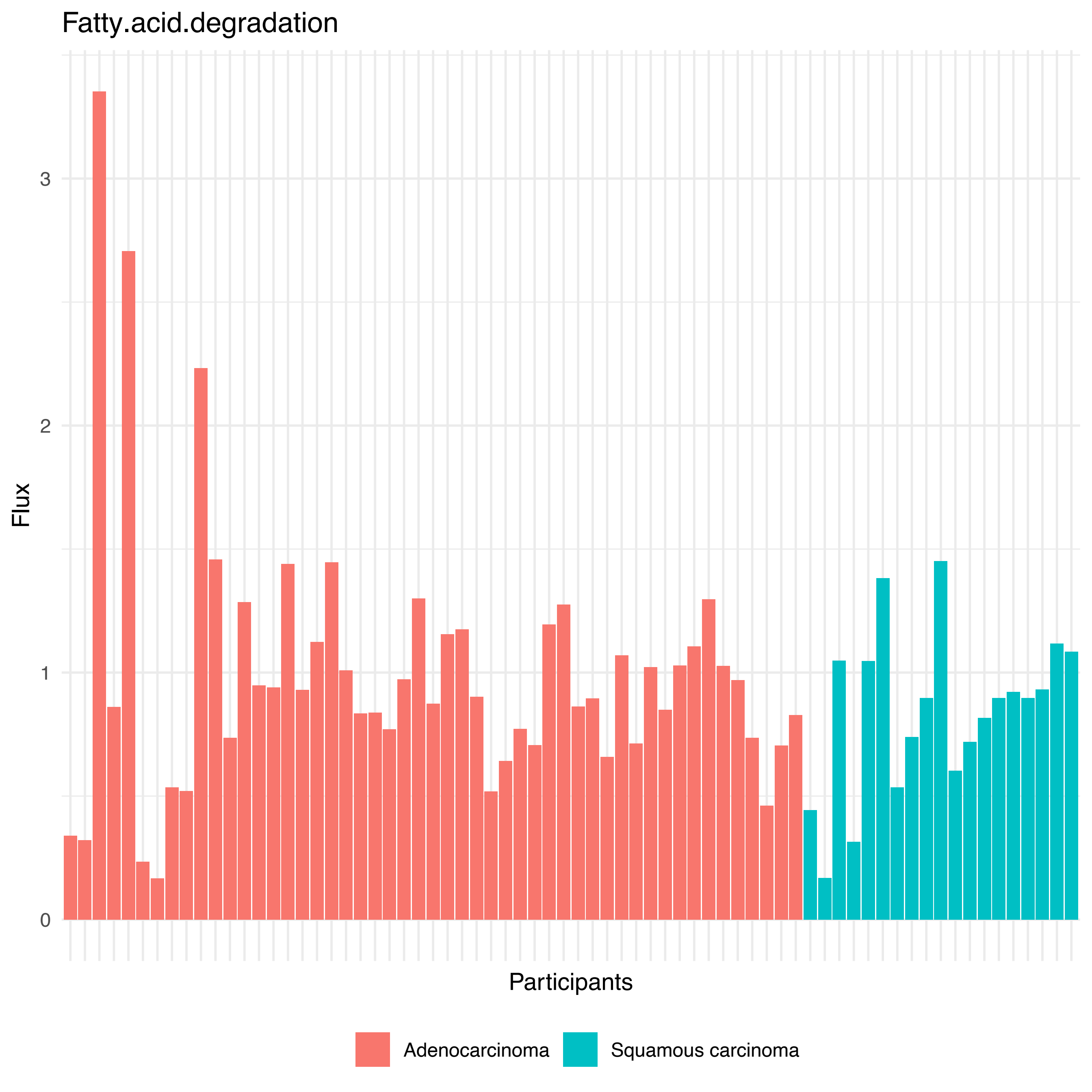

Fatty.acid.degradation
Flux
3
2
1
0
Participants
Adenocarcinoma
Squamous carcinoma

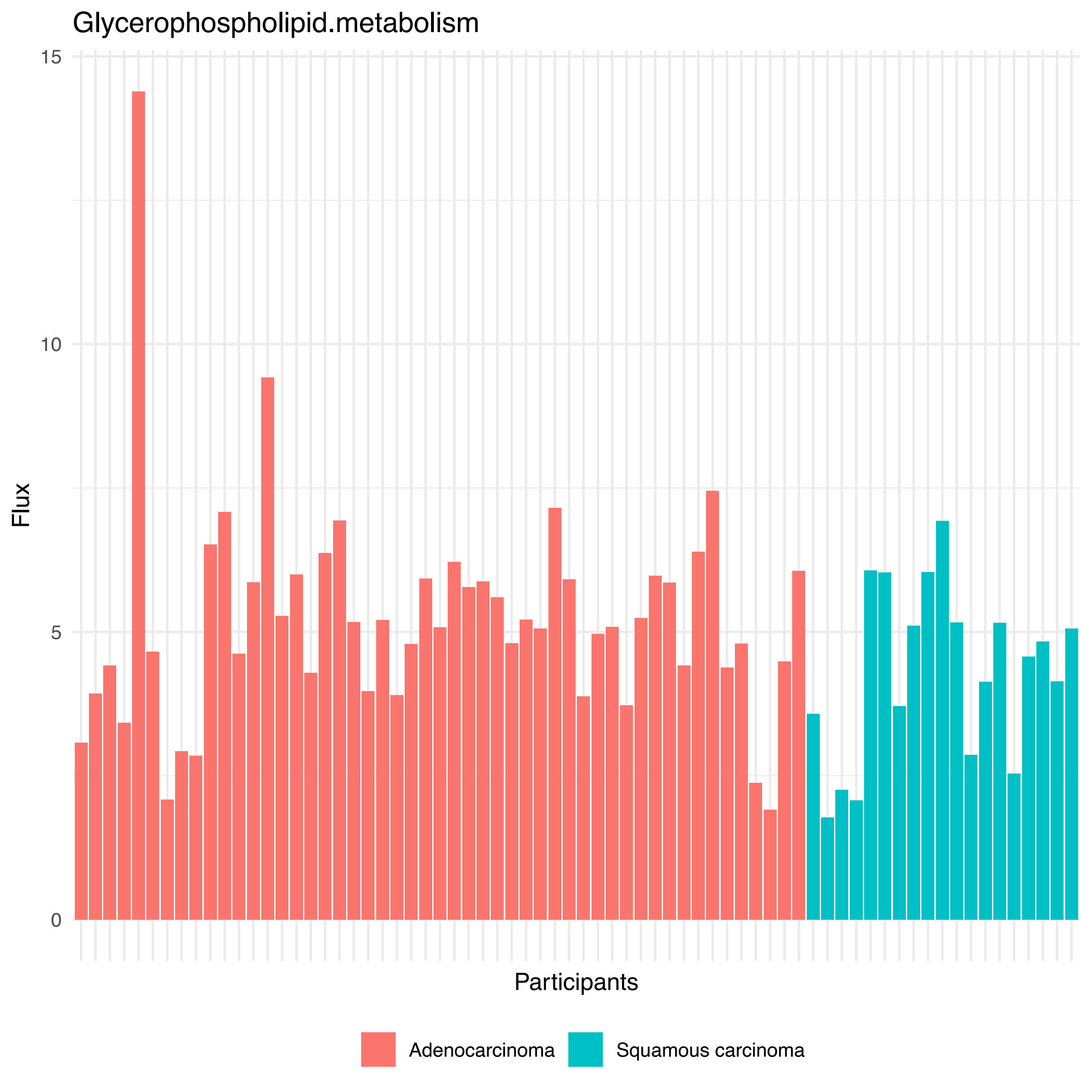

Glycerophospholipid.metabolism
15
10
5
0
Flux
Participants
Adenocarcinoma
Squamous carcinoma

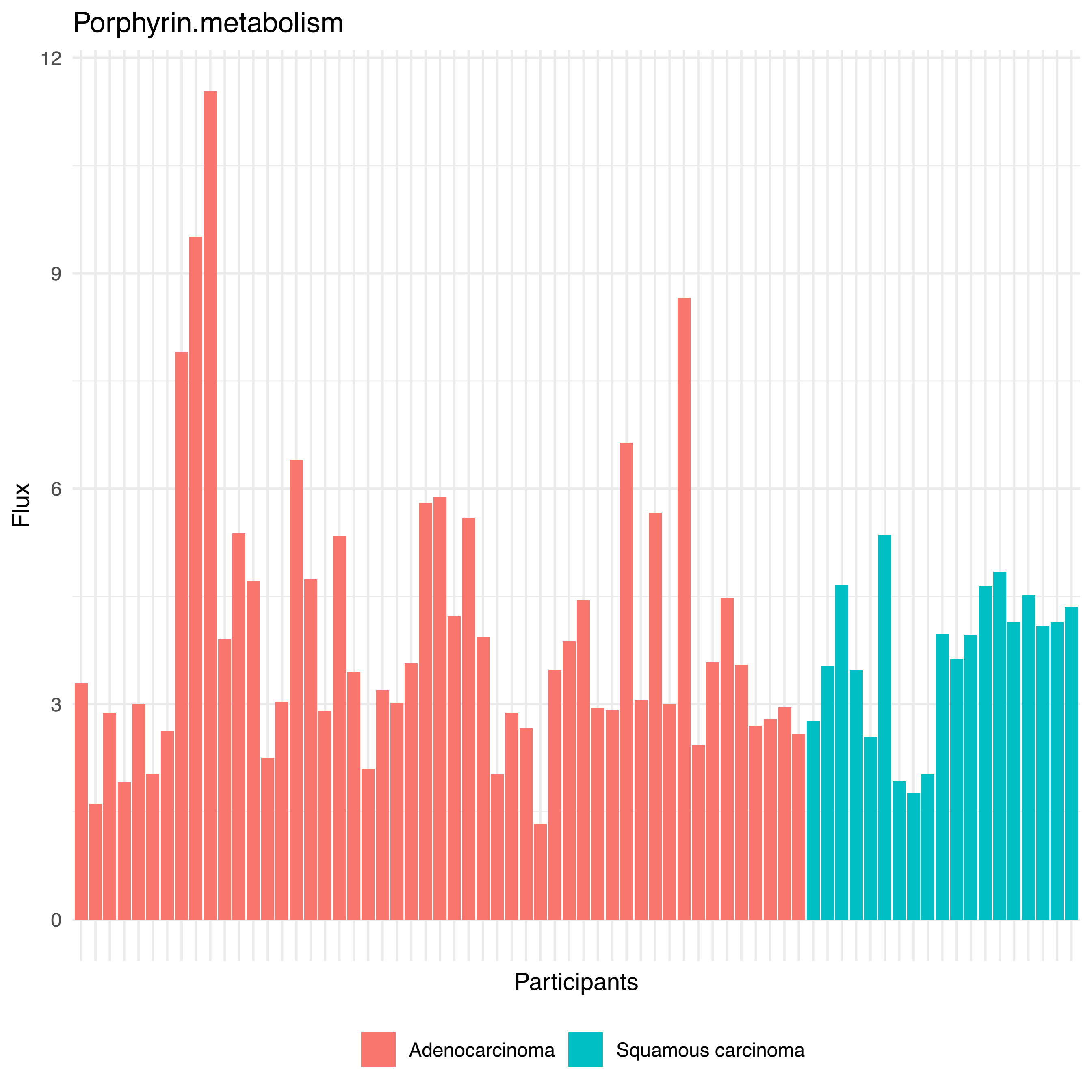
Porphyrin.metabolism
12
9
6
3
0
Flux
Participants
Adenocarcinoma
Squamous carcinoma

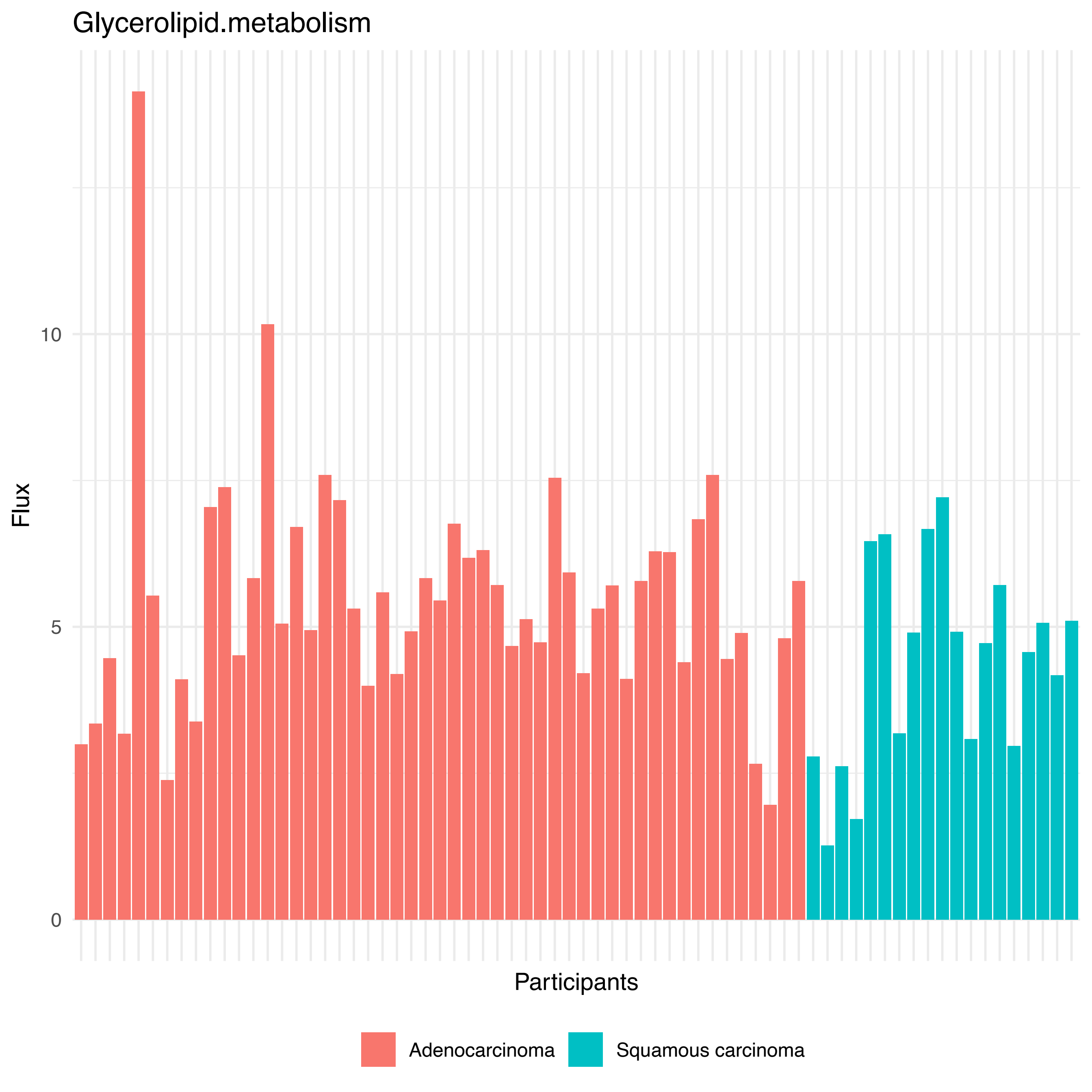
Glycerolipid.metabolism
Flux
10
5
0
Participants
Adenocarcinoma
Squamous carcinoma

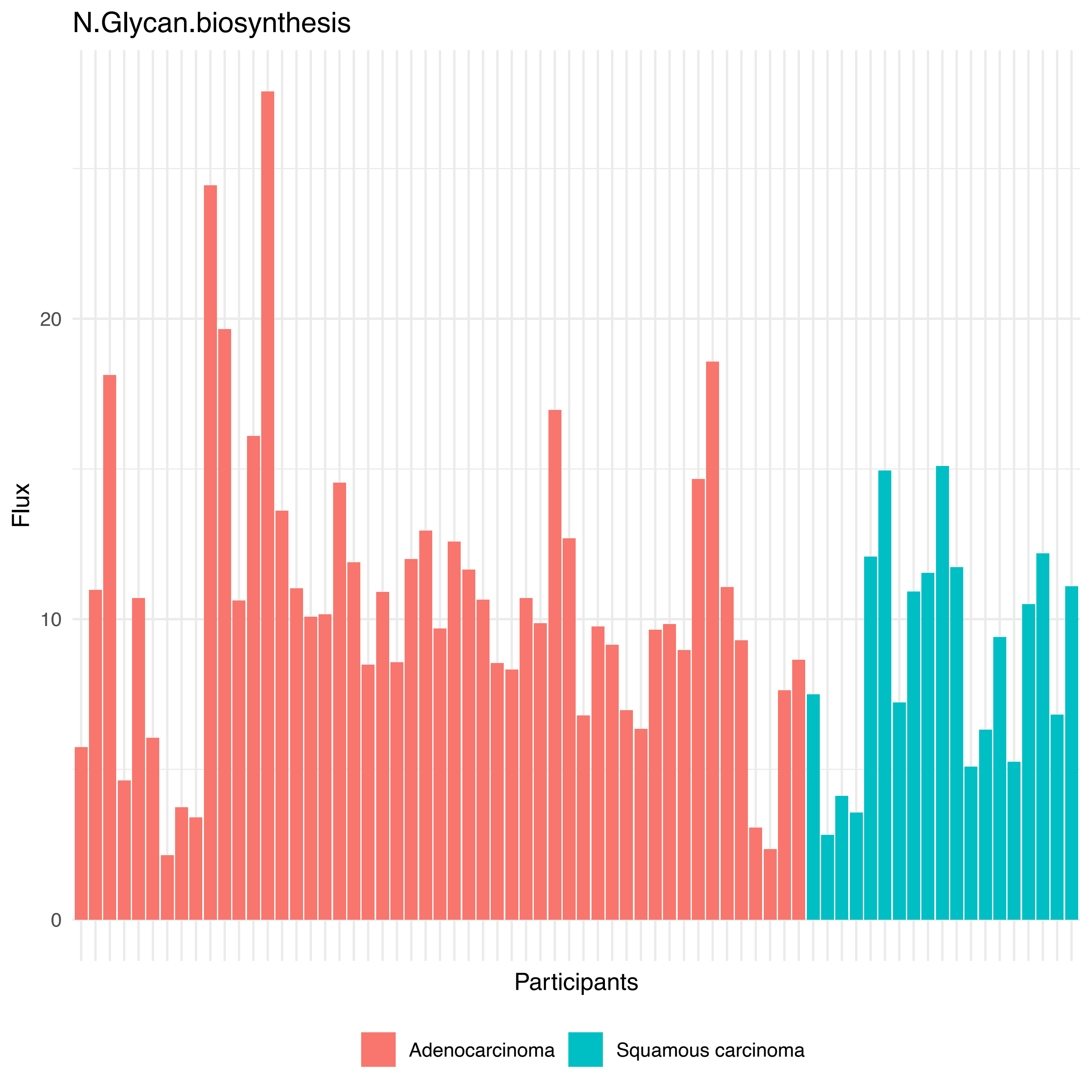
N.Glycan.biosynthesis
Flux
20
10
0
Participants
Adenocarcinoma
Squamous carcinoma

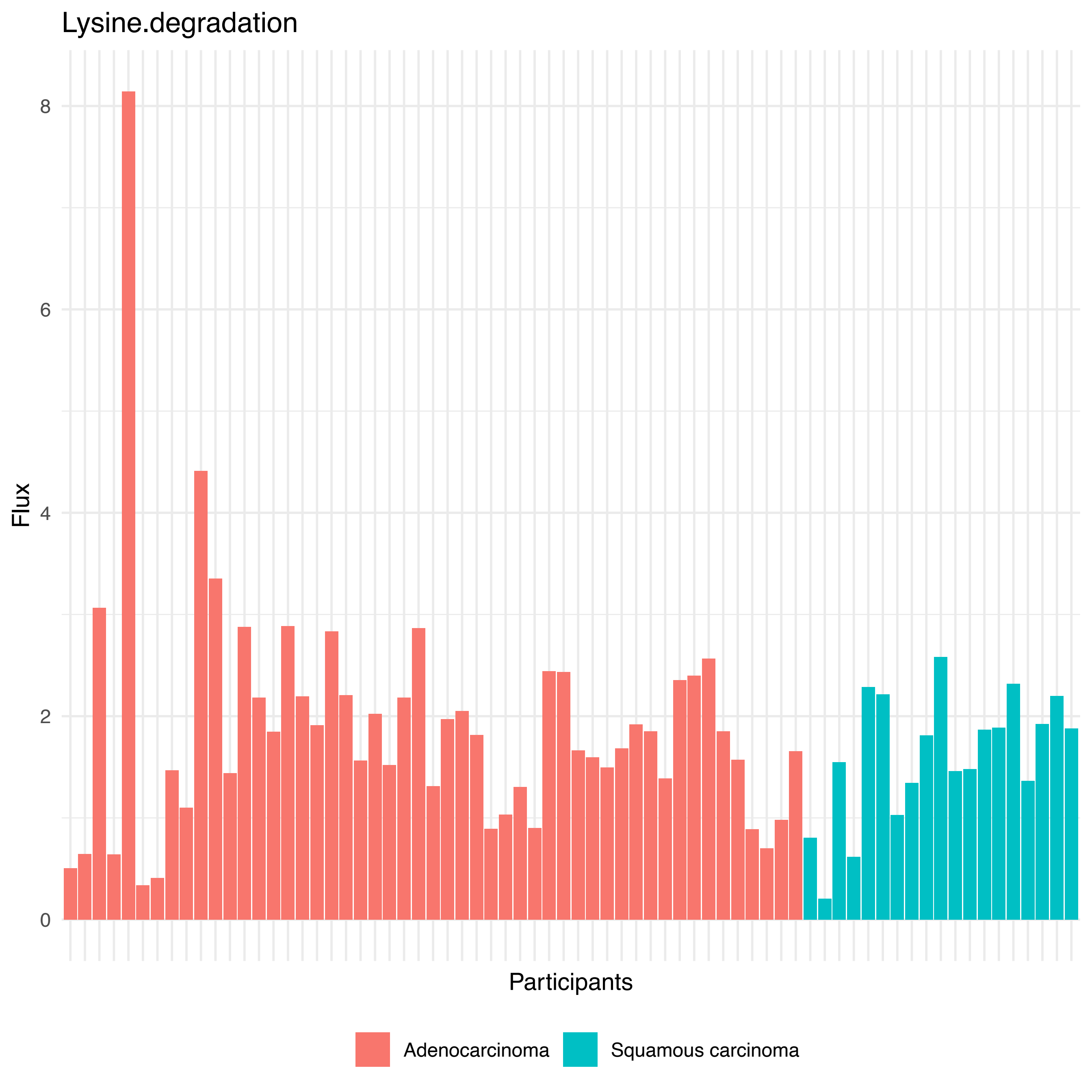
Lysine.degradation
Flux
8
6
4
2
0
Participants
Adenocarcinoma
Squamous carcinoma

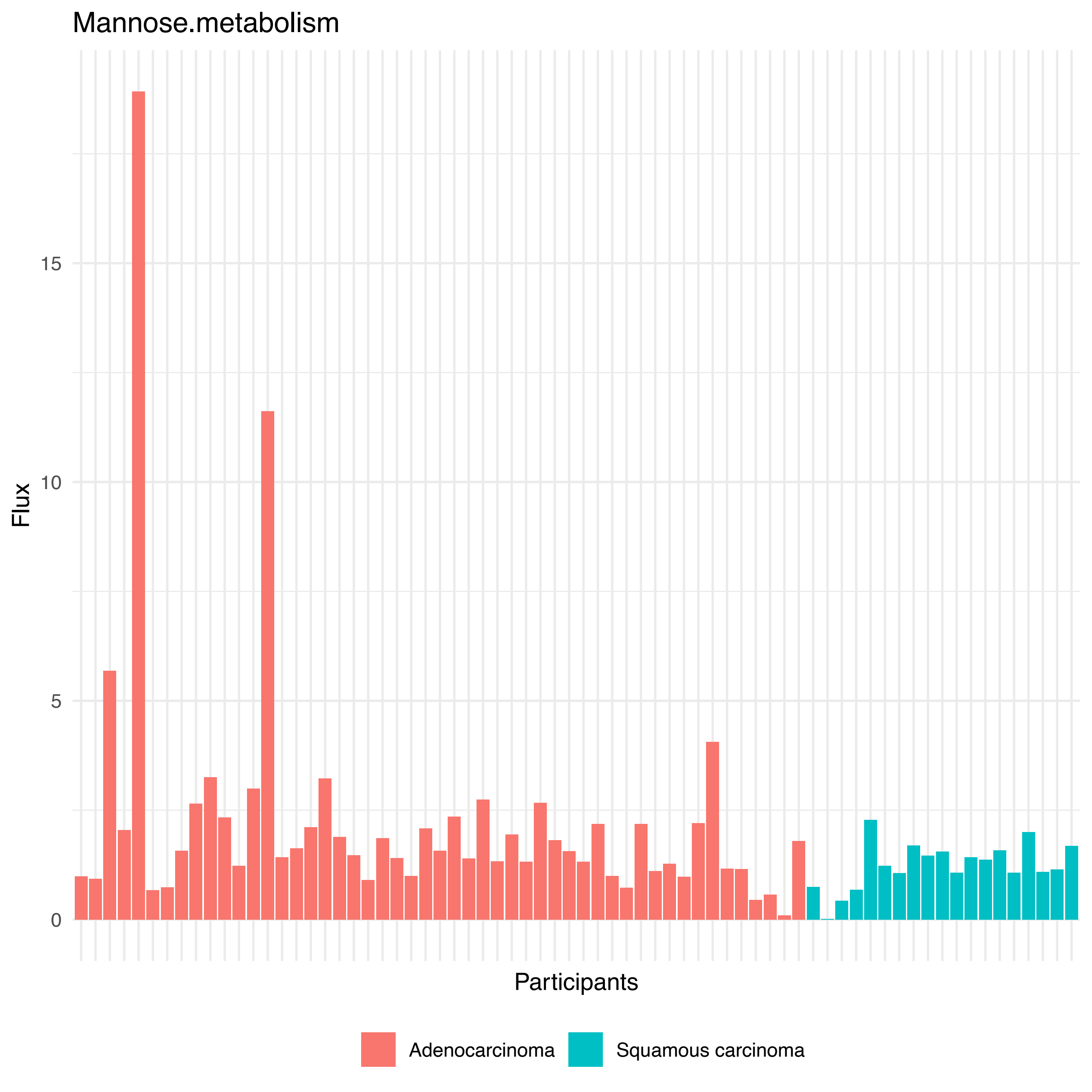

Mannose.metabolism
15
10
5
0
Flux
Participants
Adenocarcinoma
Squamous carcinoma

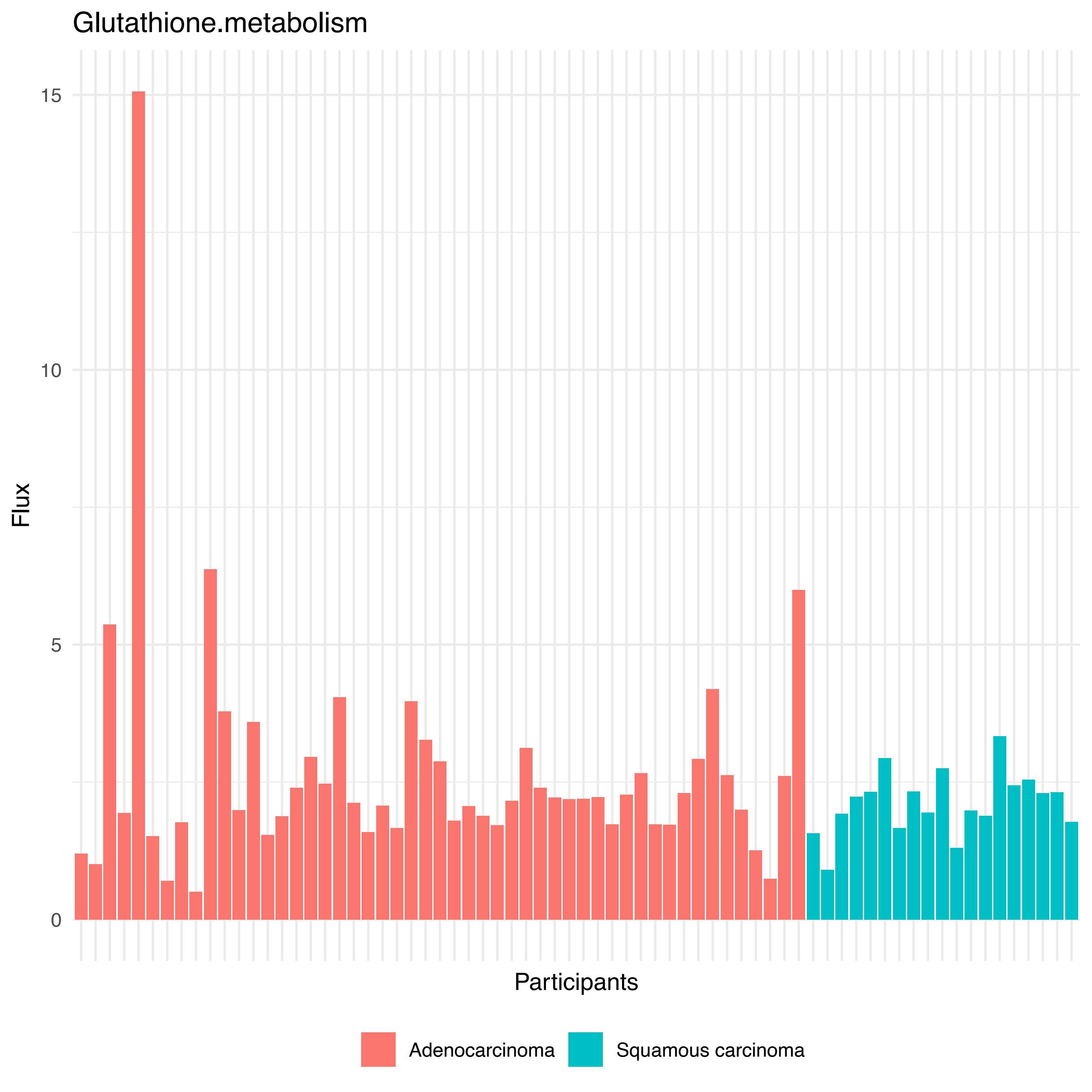
Glutathione.metabolism
Flux
15
10
5
0
Participants
Adenocarcinoma
Squamous carcinoma

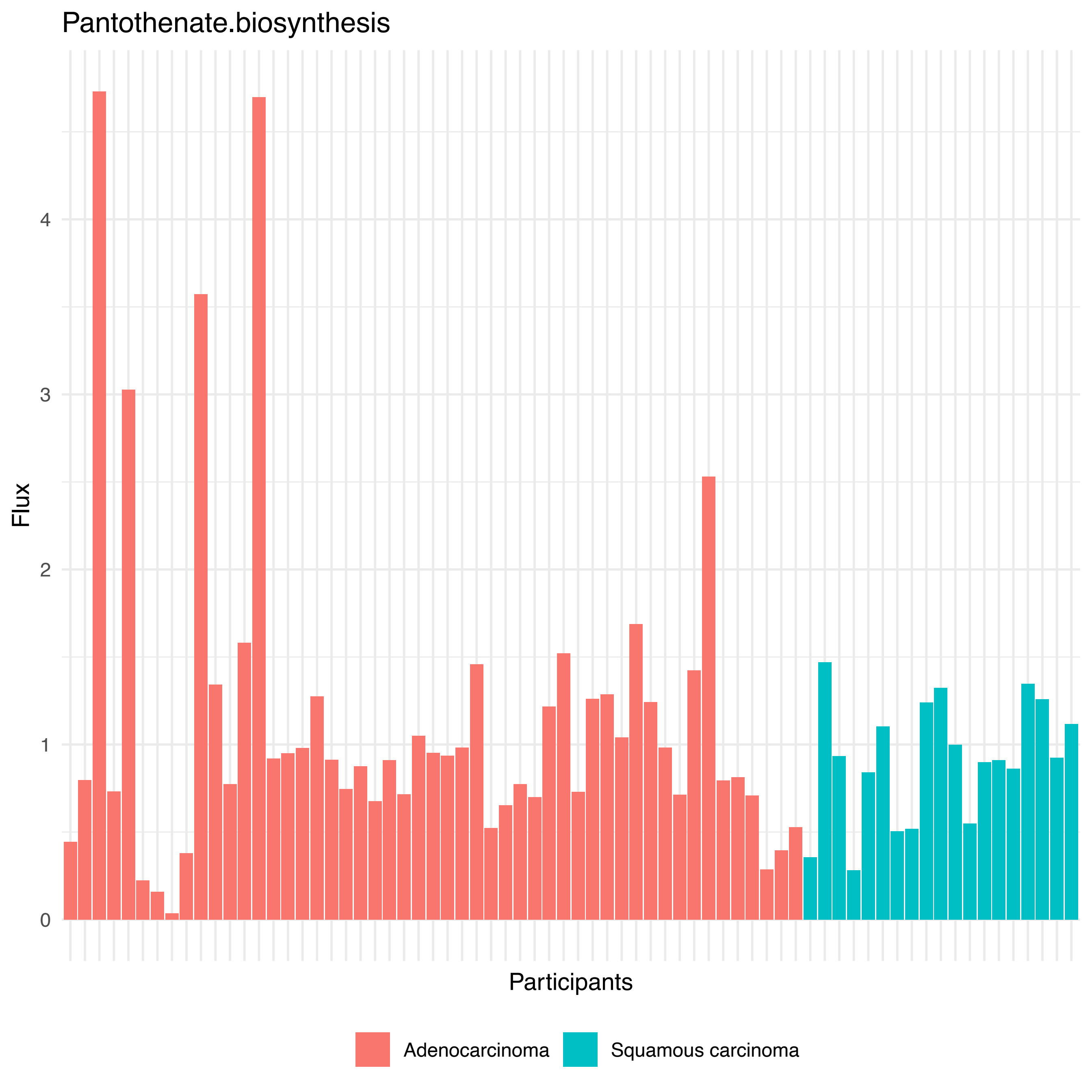

Pantothenate.biosynthesis
Flux
0
1
2
3
4
Participants
Adenocarcinoma
Squamous carcinoma

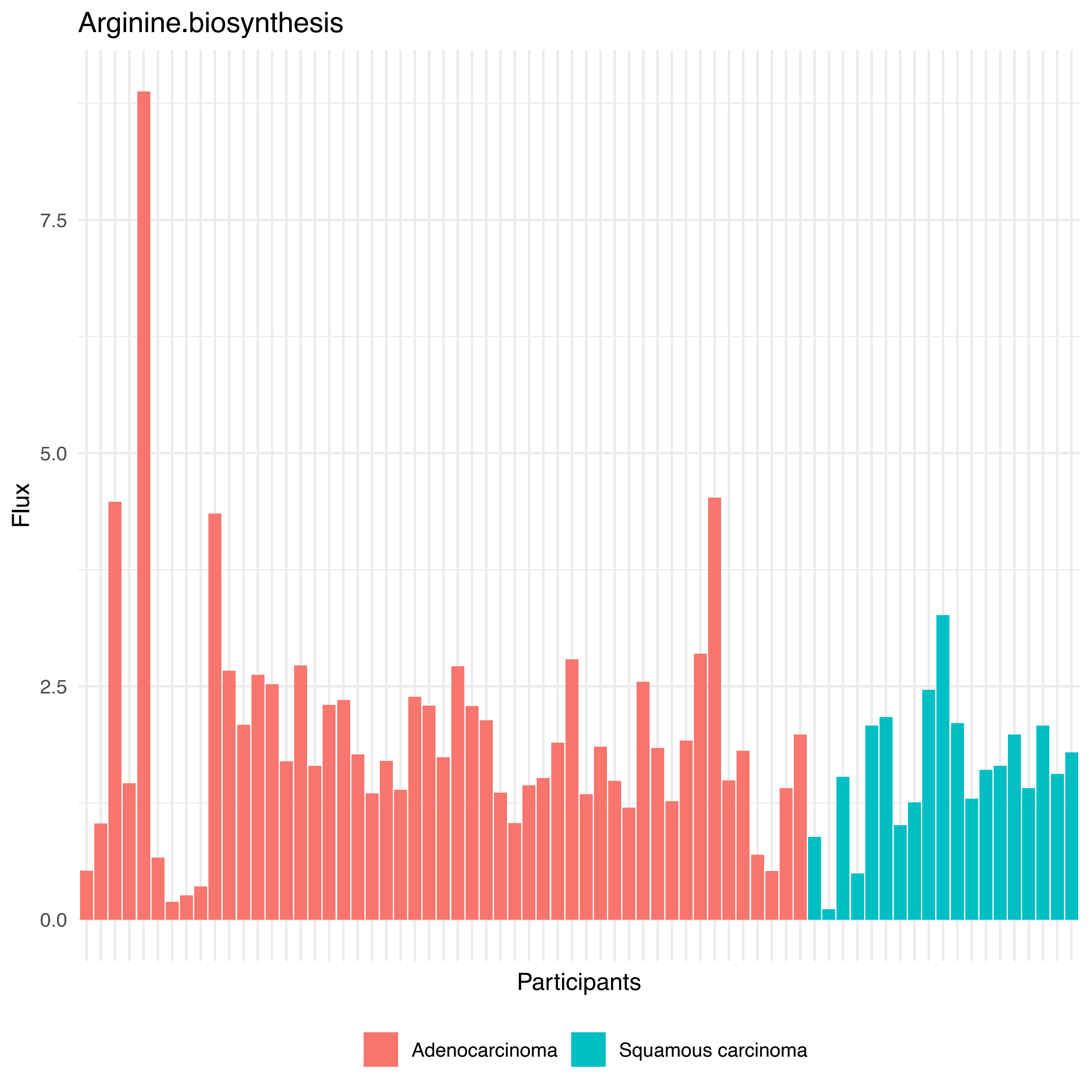

Arginine.biosynthesis
7.5
5.0
2.5
0.0
Flux
Participants
Adenocarcinoma
Squamous carcinoma

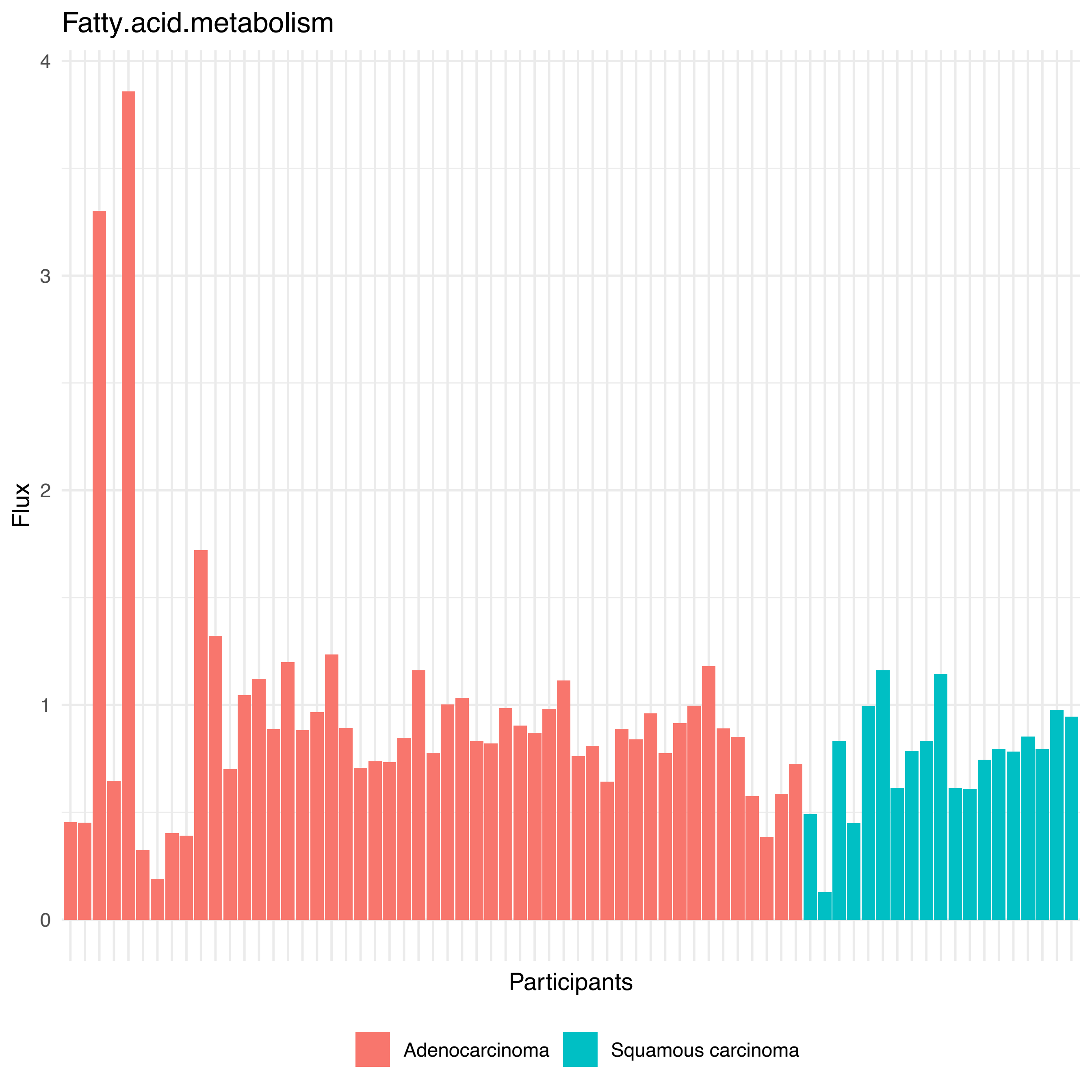

Fatty.acid.metabolism
Flux
4
3
2
1
0
Participants
Adenocarcinoma
Squamous carcinoma

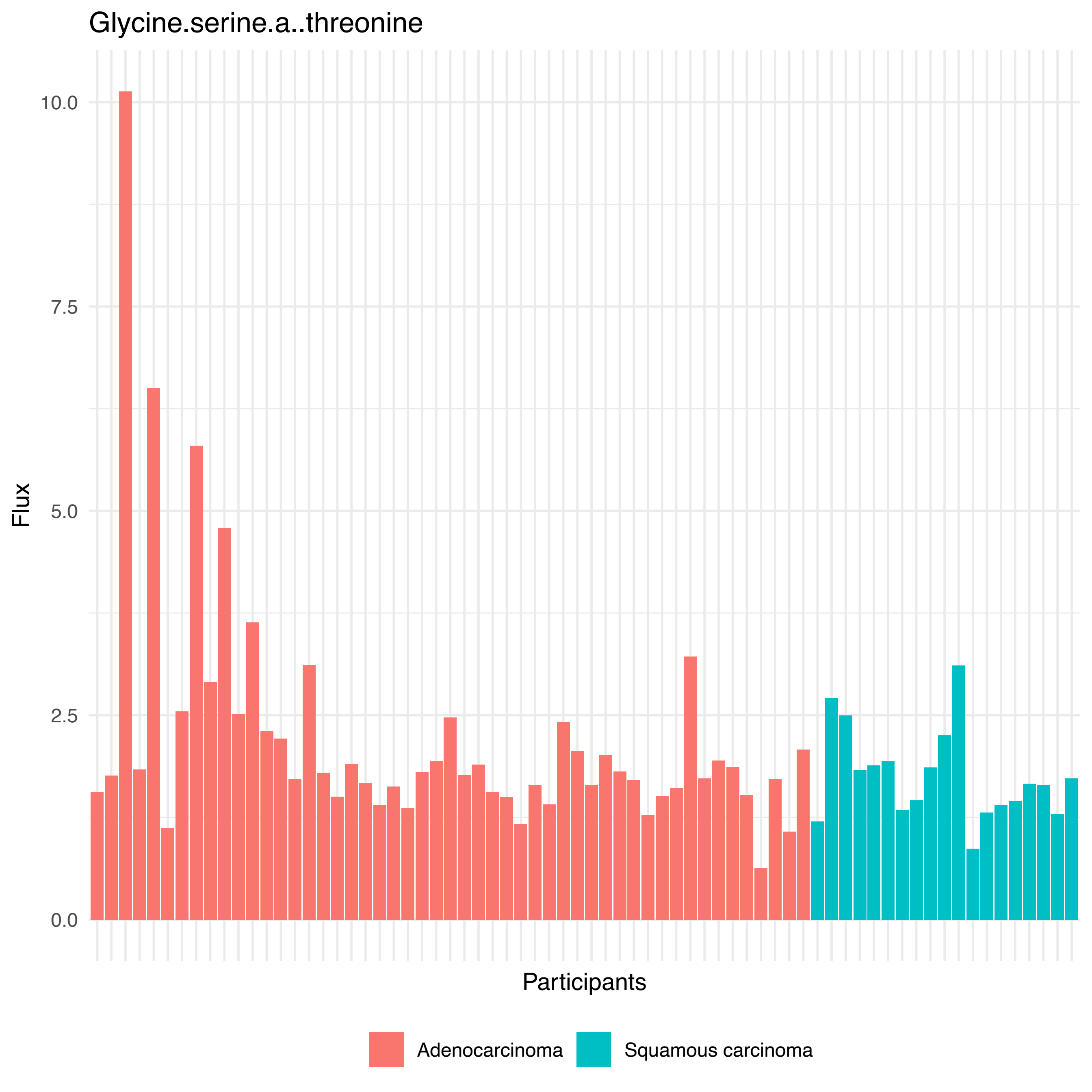
Glycine.serine.a..threonine
Flux
10.0
7.5
5.0
2.5
0.0
Participants
Adenocarcinoma
Squamous carcinoma

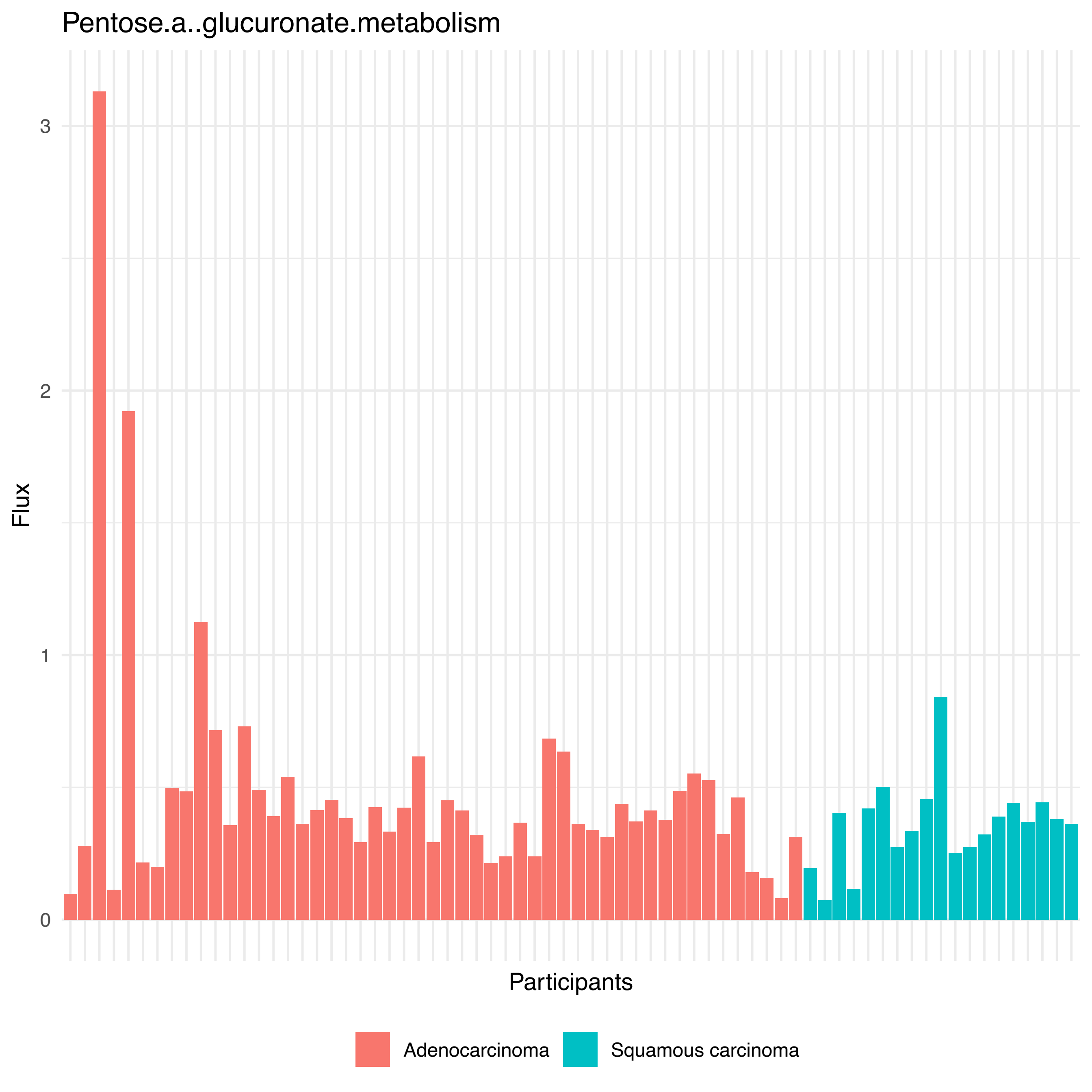

Pentose.a..glucuronate.metabolism
Flux
3
2
1
0
Participants
Adenocarcinoma
Squamous carcinoma

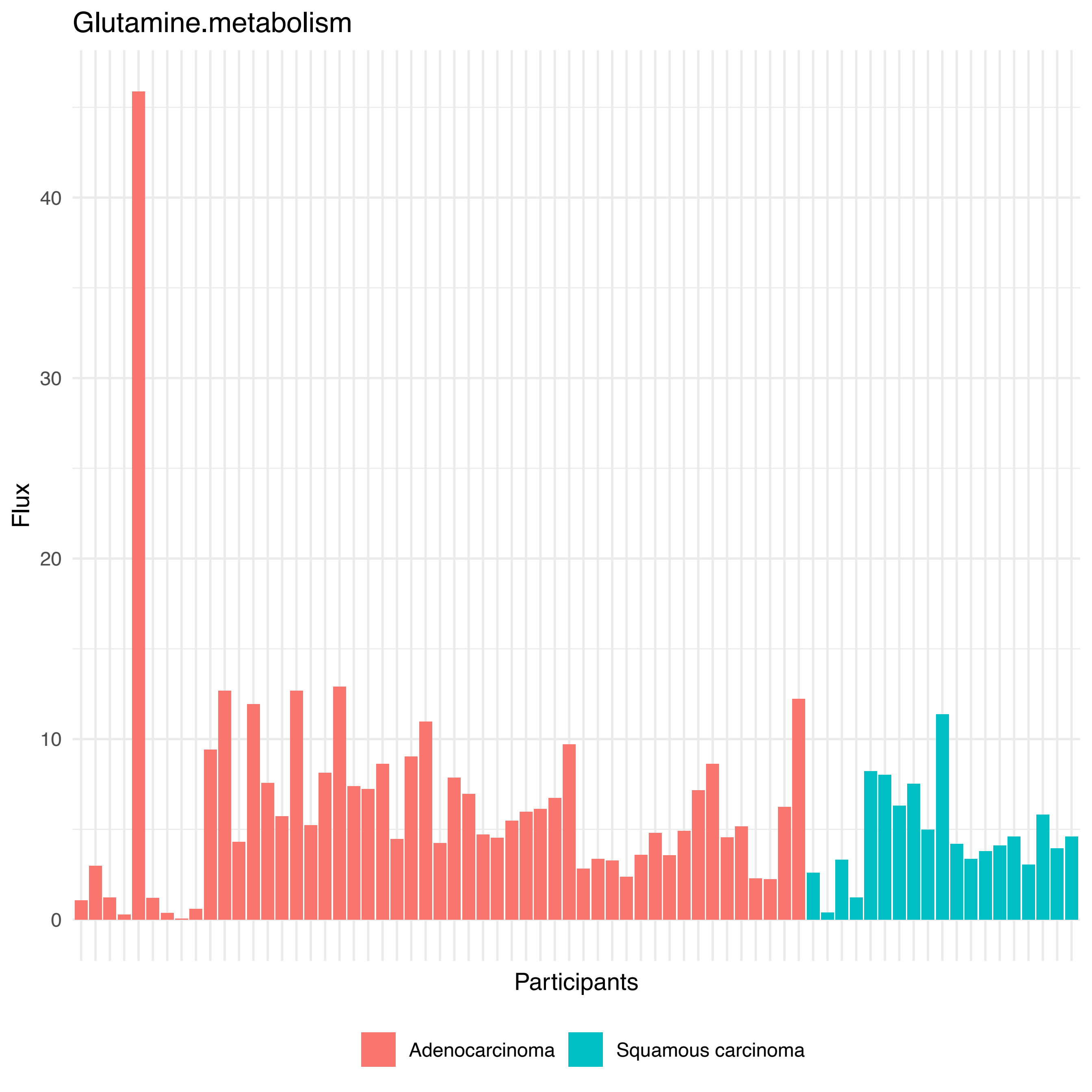

Glutamine.metabolism
Flux
0
10
20
30
40
Participants
Adenocarcinoma
Squamous carcinoma

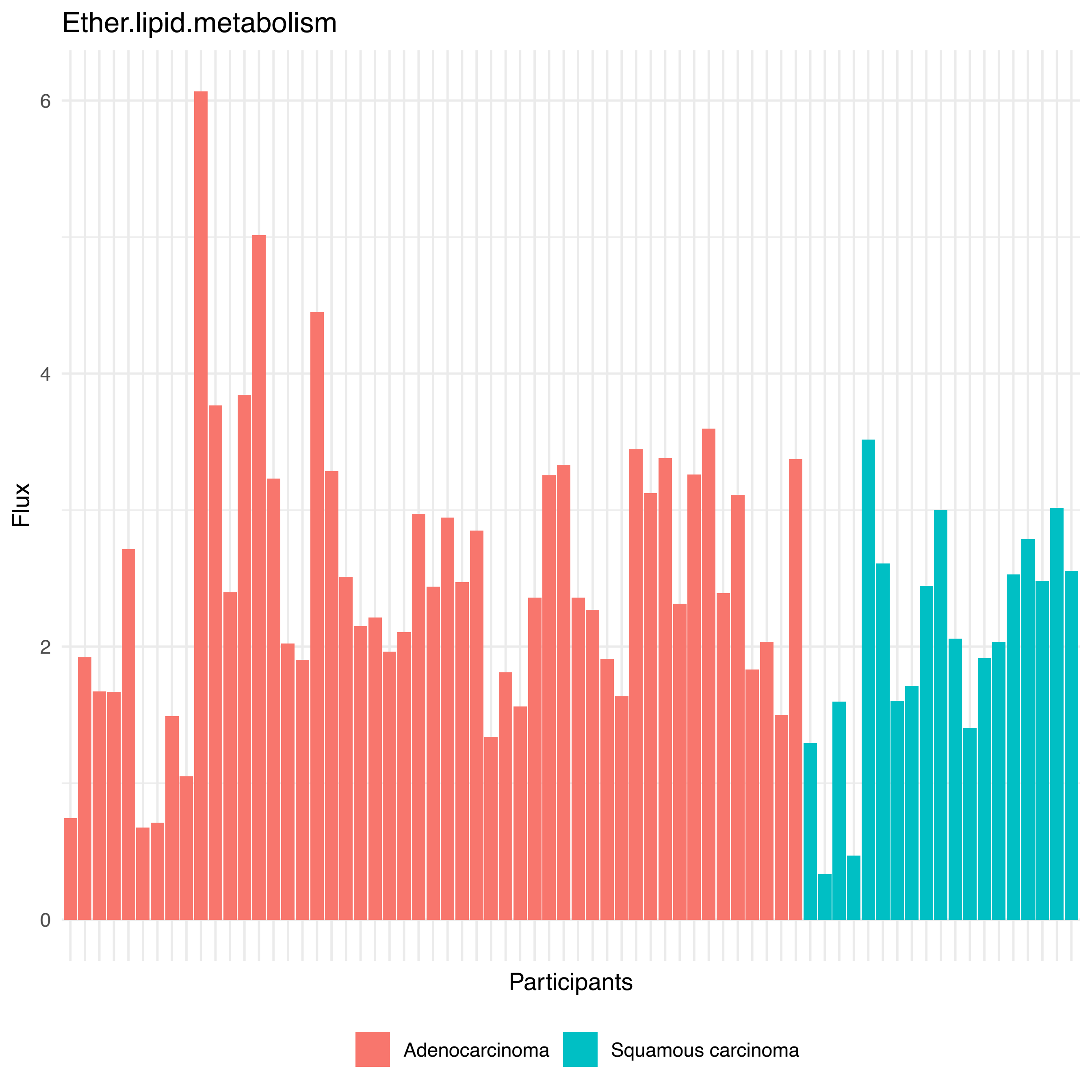
Ether.lipid.metabolism
Flux
6
4
2
0
Participants
Adenocarcinoma
Squamous carcinoma

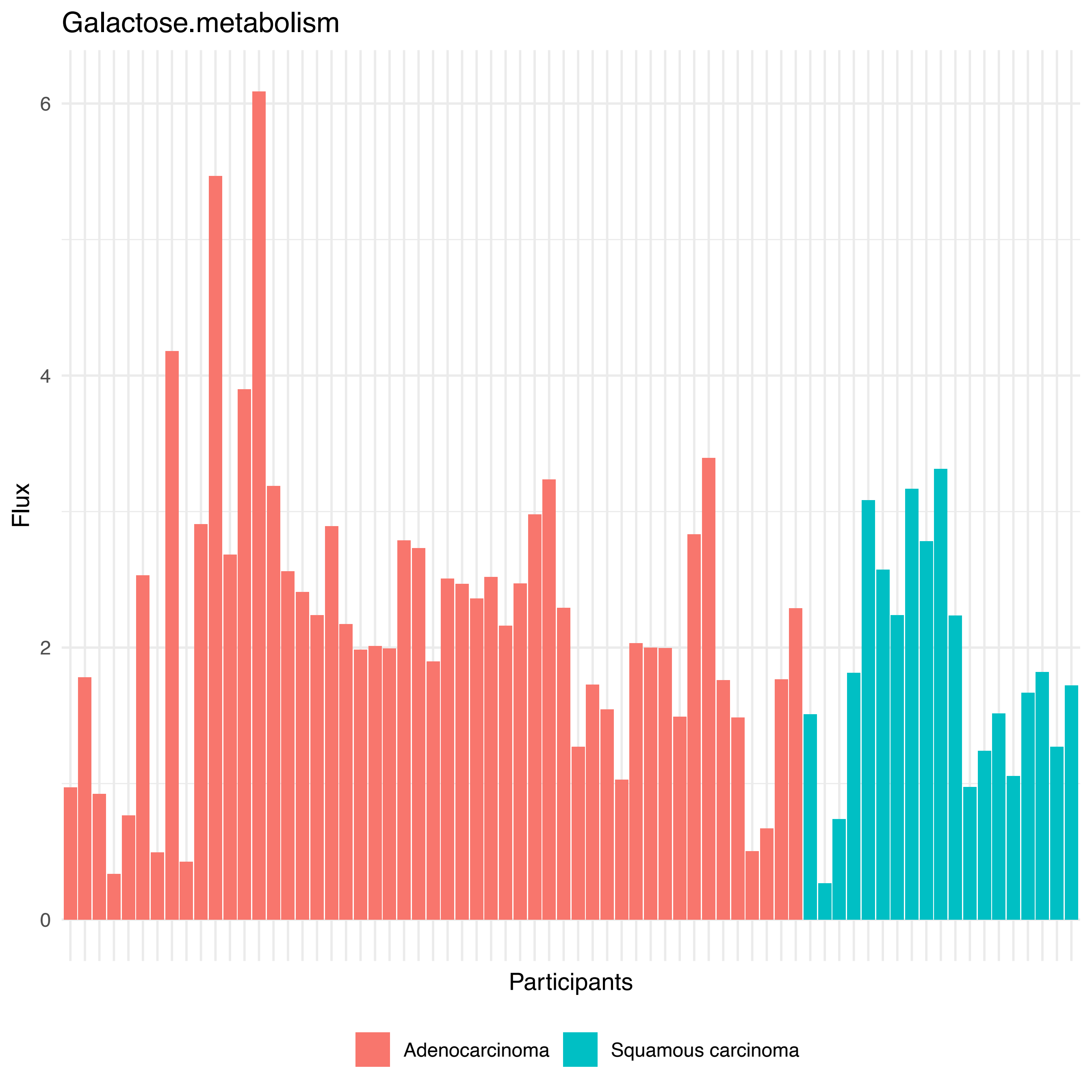

Galactose.metabolism
Flux
6
4
2
0
Participants
Adenocarcinoma
Squamous carcinoma

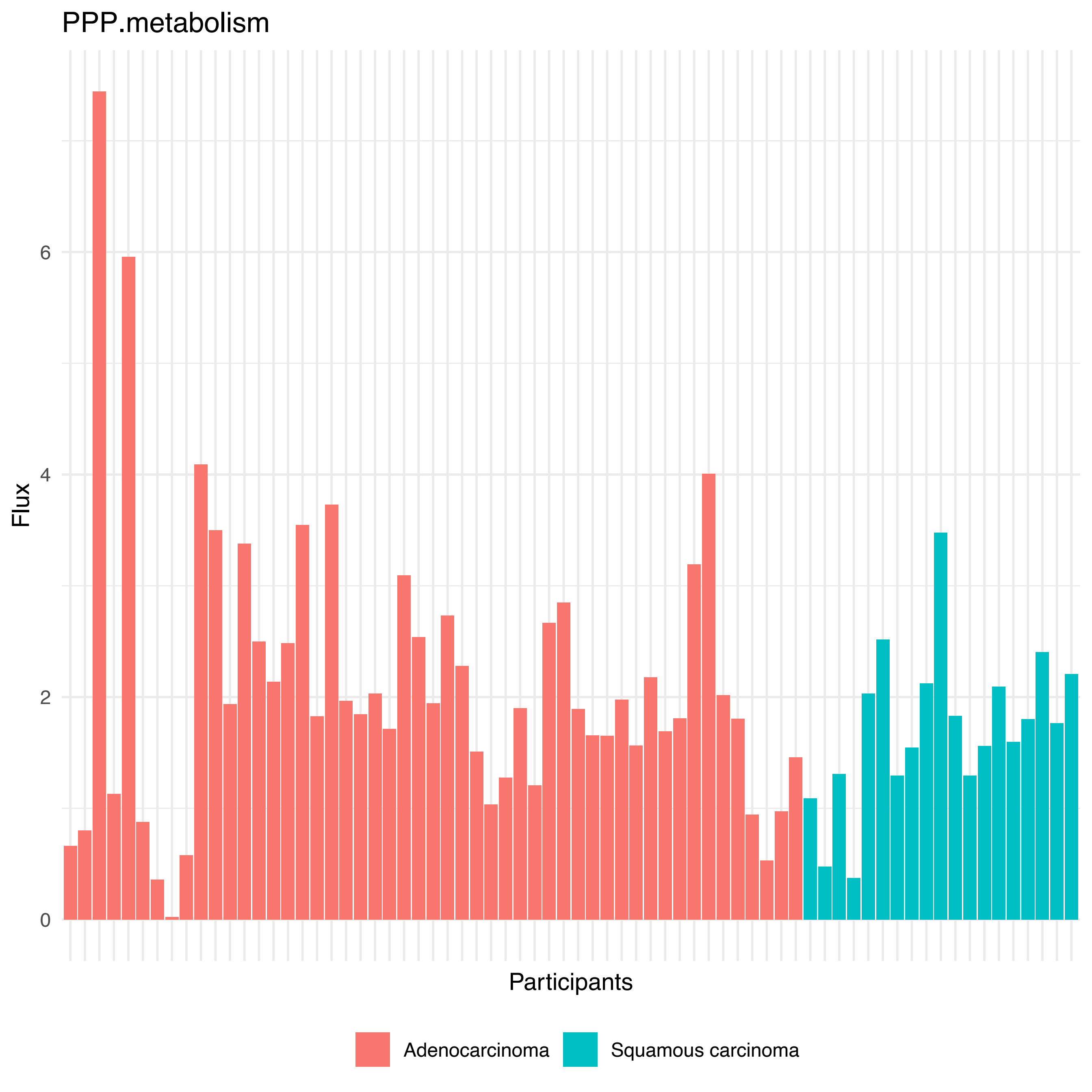
PPP.metabolism
Flux
6
4
2
0
Participants
Adenocarcinoma
Squamous carcinoma

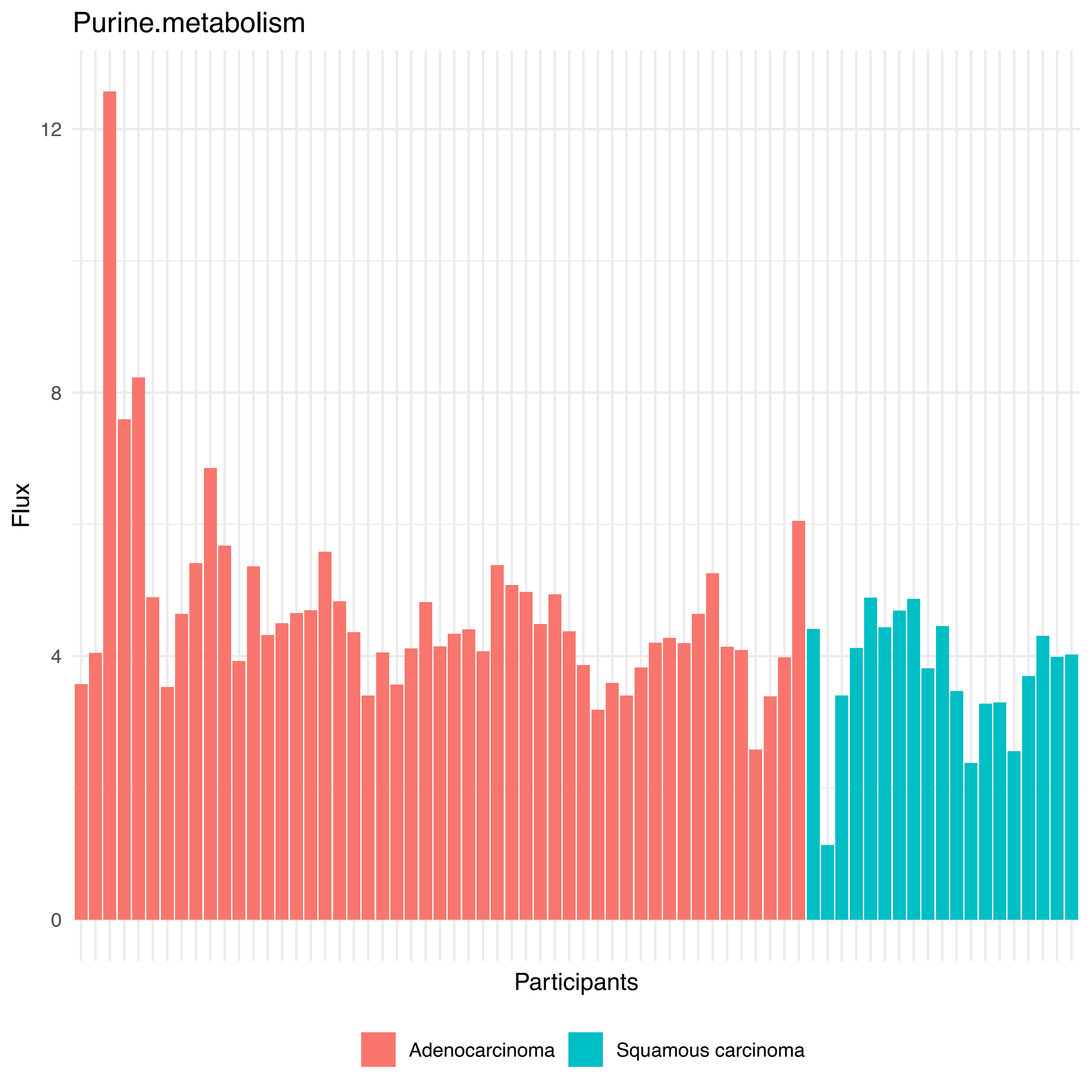

Purine.metabolism
Flux
12
8
4
0
Participants
Adenocarcinoma
Squamous carcinoma

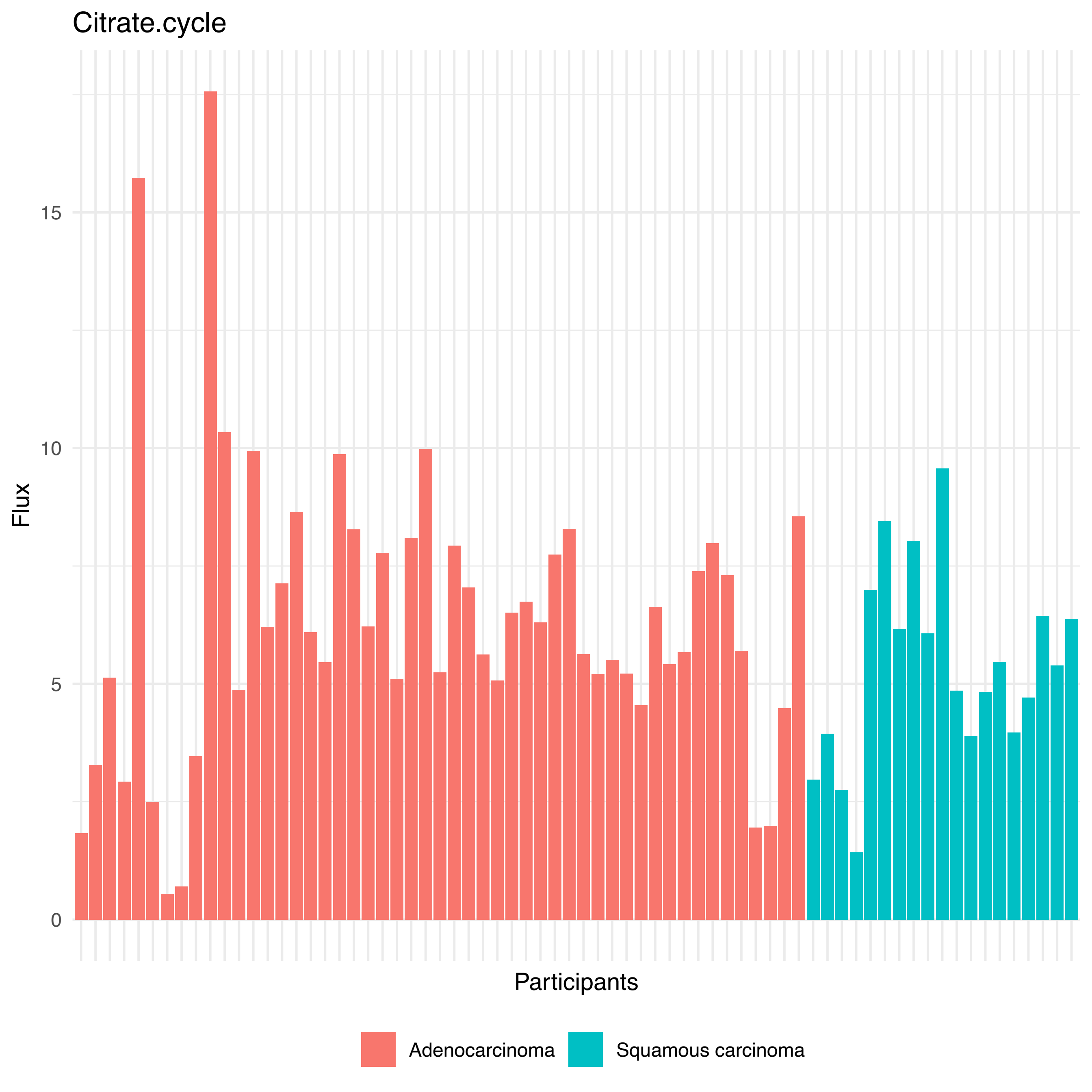

Citrate.cycle
Flux
15
10
5
0
Participants
Adenocarcinoma
Squamous carcinoma

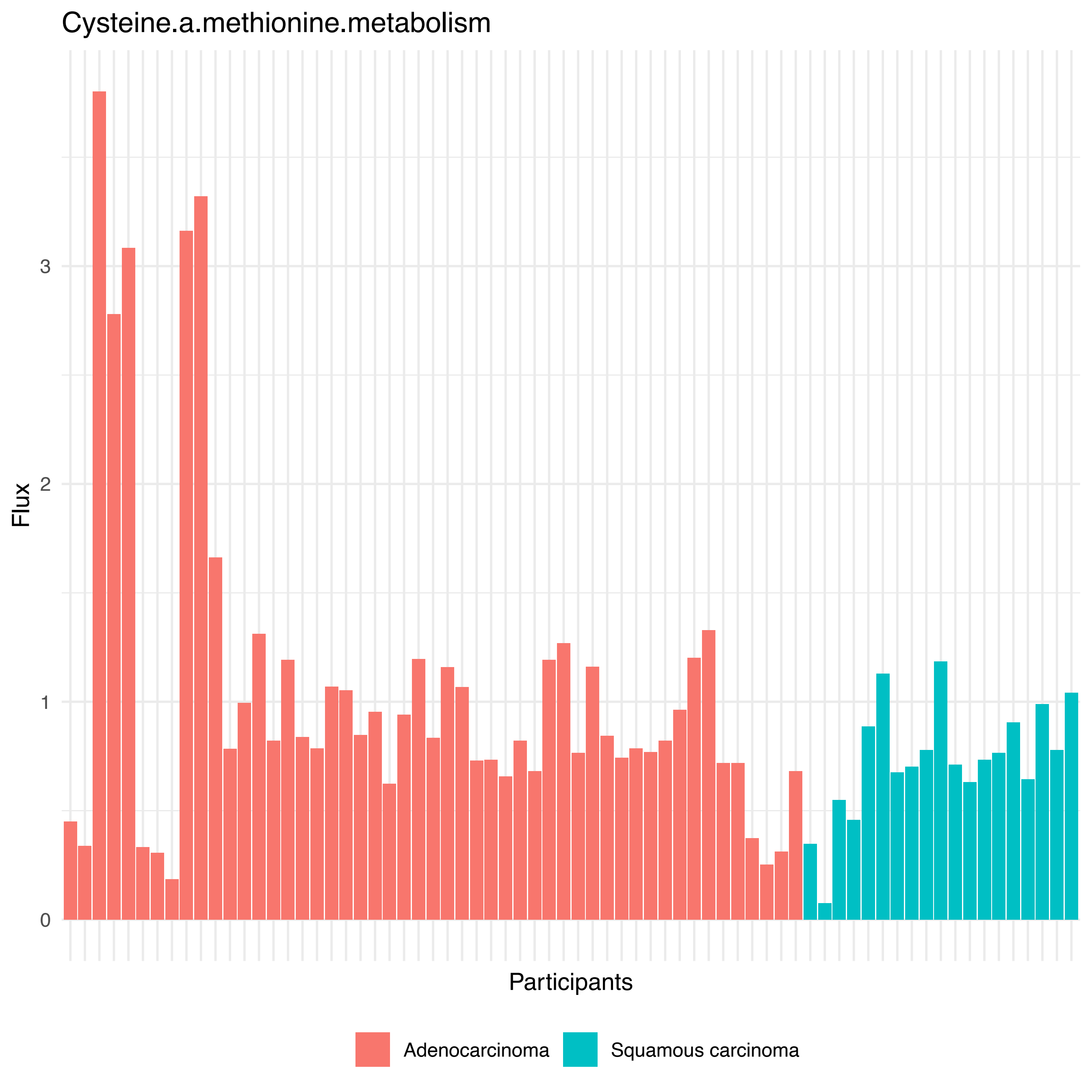

Cysteine.a.methionine.metabolism
Flux
3
2
1
0
Participants
Adenocarcinoma
Squamous carcinoma

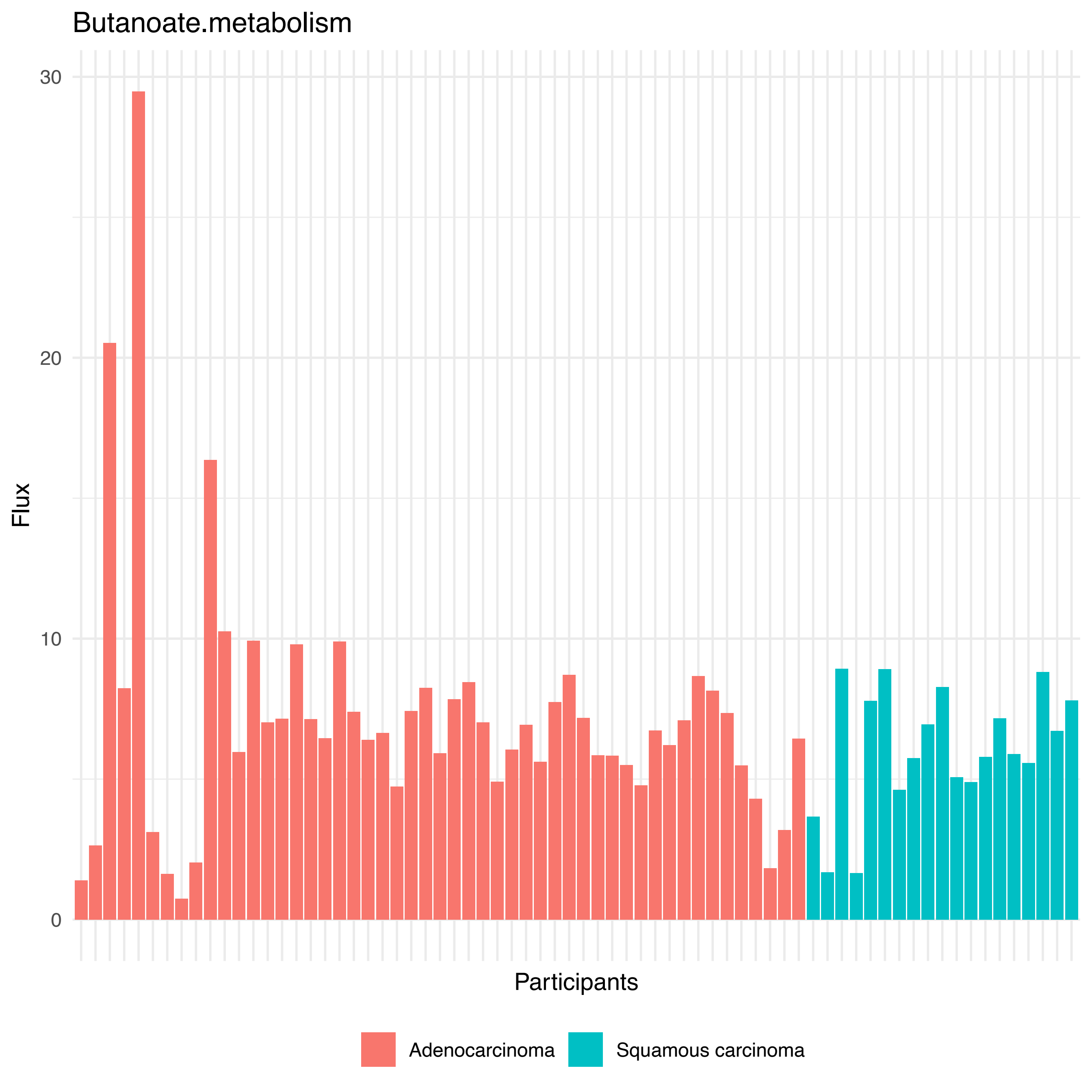

Butanoate.metabolism
30
20
10
0
Flux
Participants
Adenocarcinoma
Squamous carcinoma

Supplement Figure 4: Pathway flux comparison between host- and tumor-side.

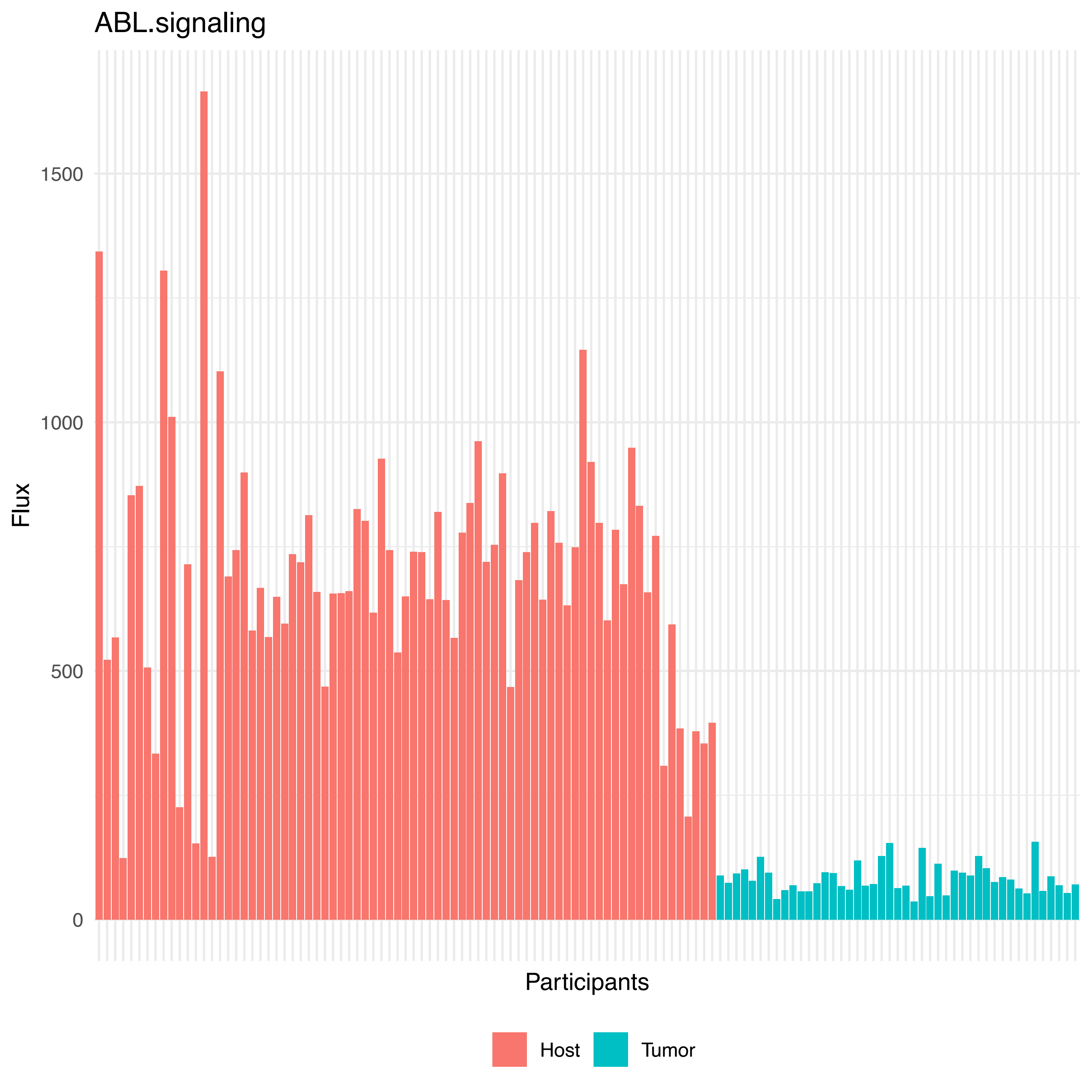
ABL.signaling
1500
1000
500
0
Flux
Participants
Host
Tumor

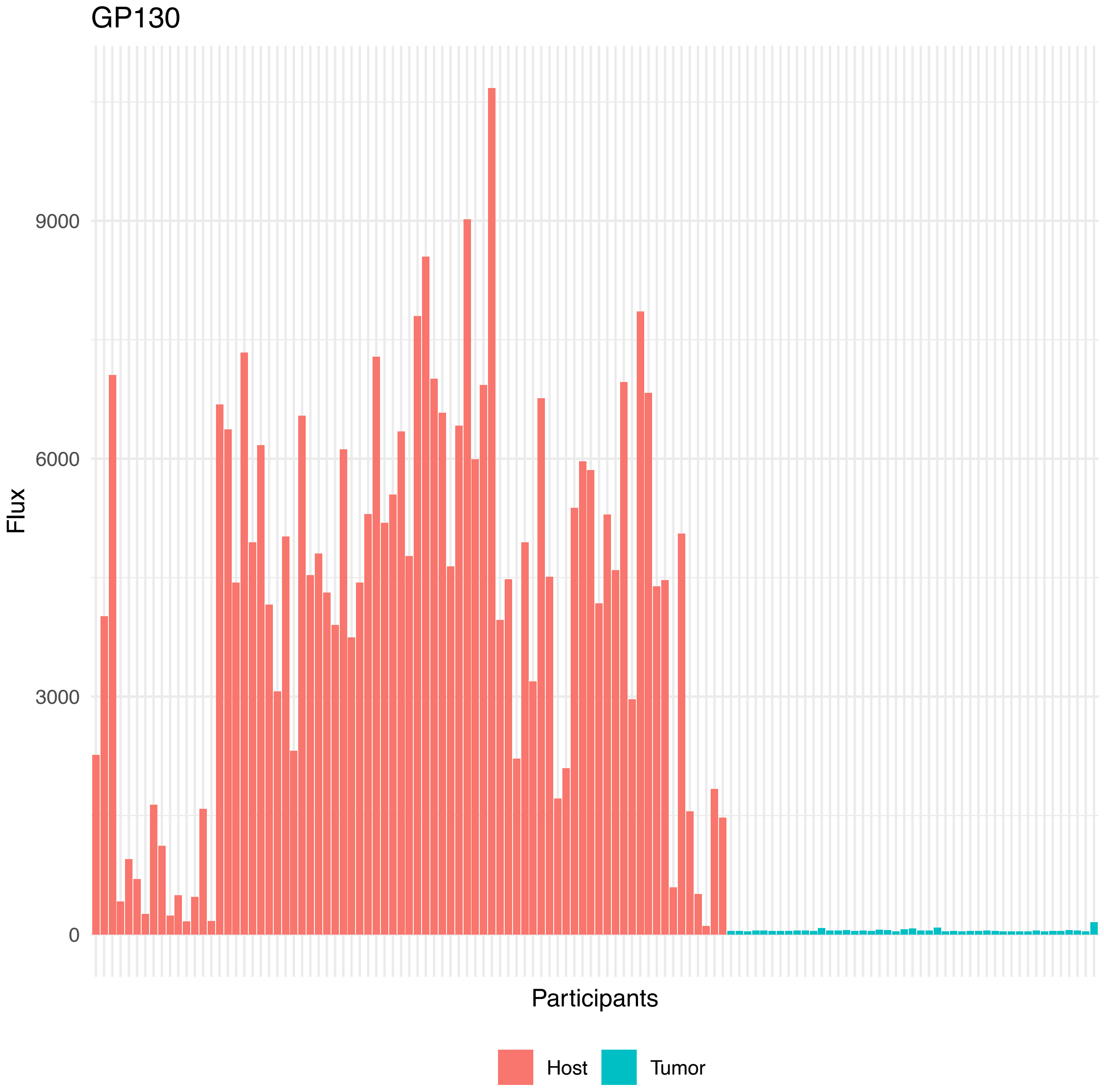
GP130
9000
6000
3000
0
Flux
Participants
Host
Tumor

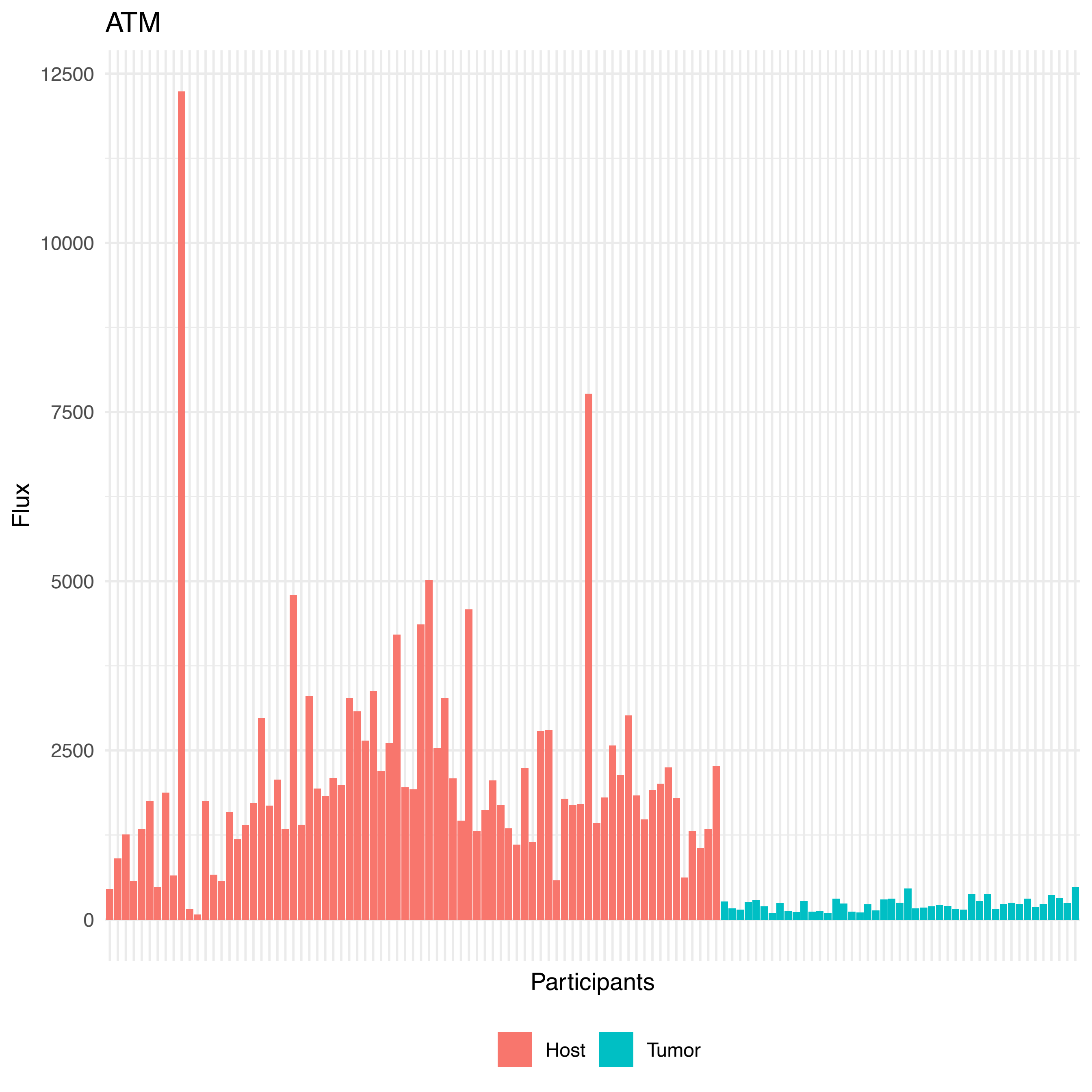

ATM
Flux
12500
10000
7500
5000
2500
0
Participants
Host
Tumor

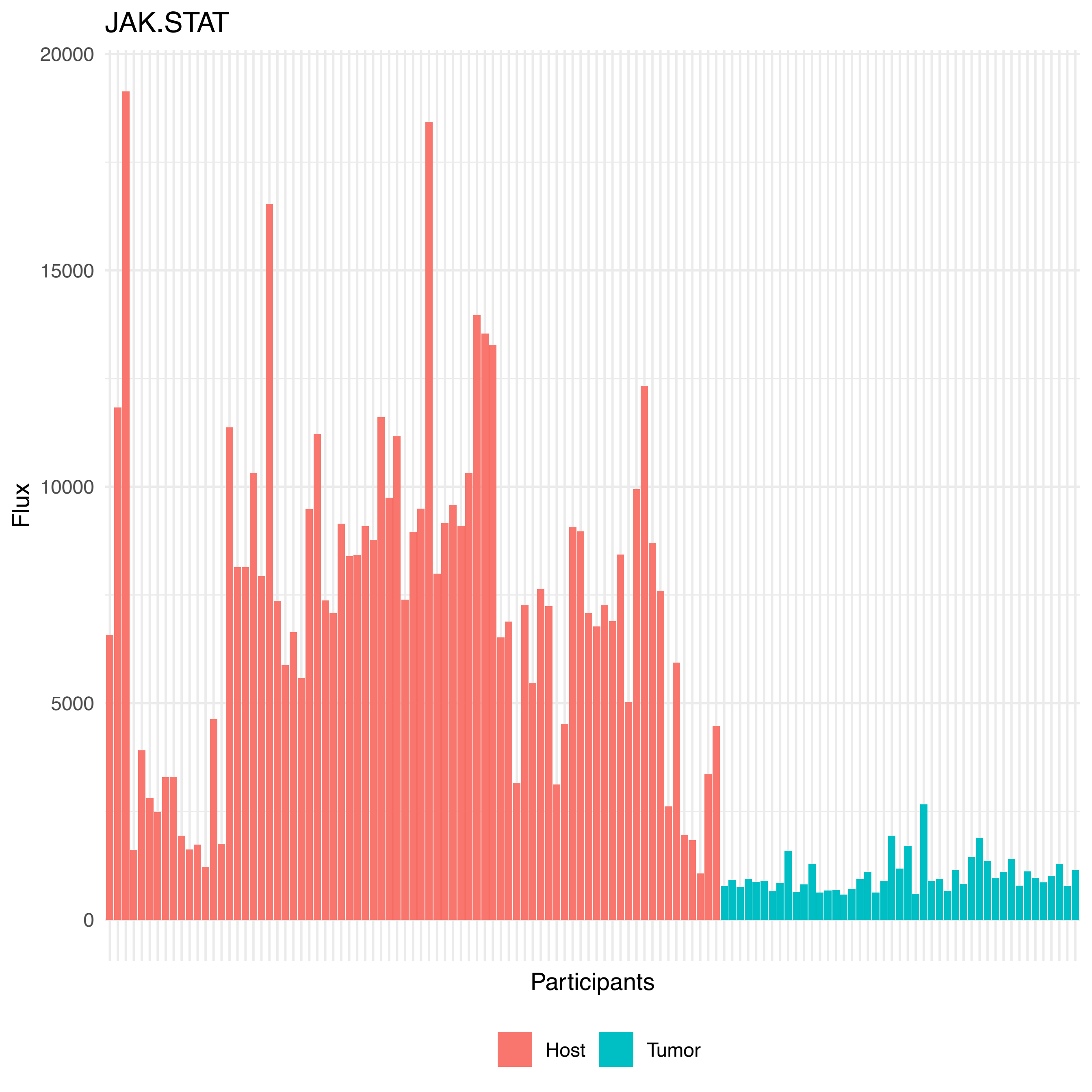

JAK.STAT
Flux
20000
15000
10000
5000
0
Participants
Host
Tumor

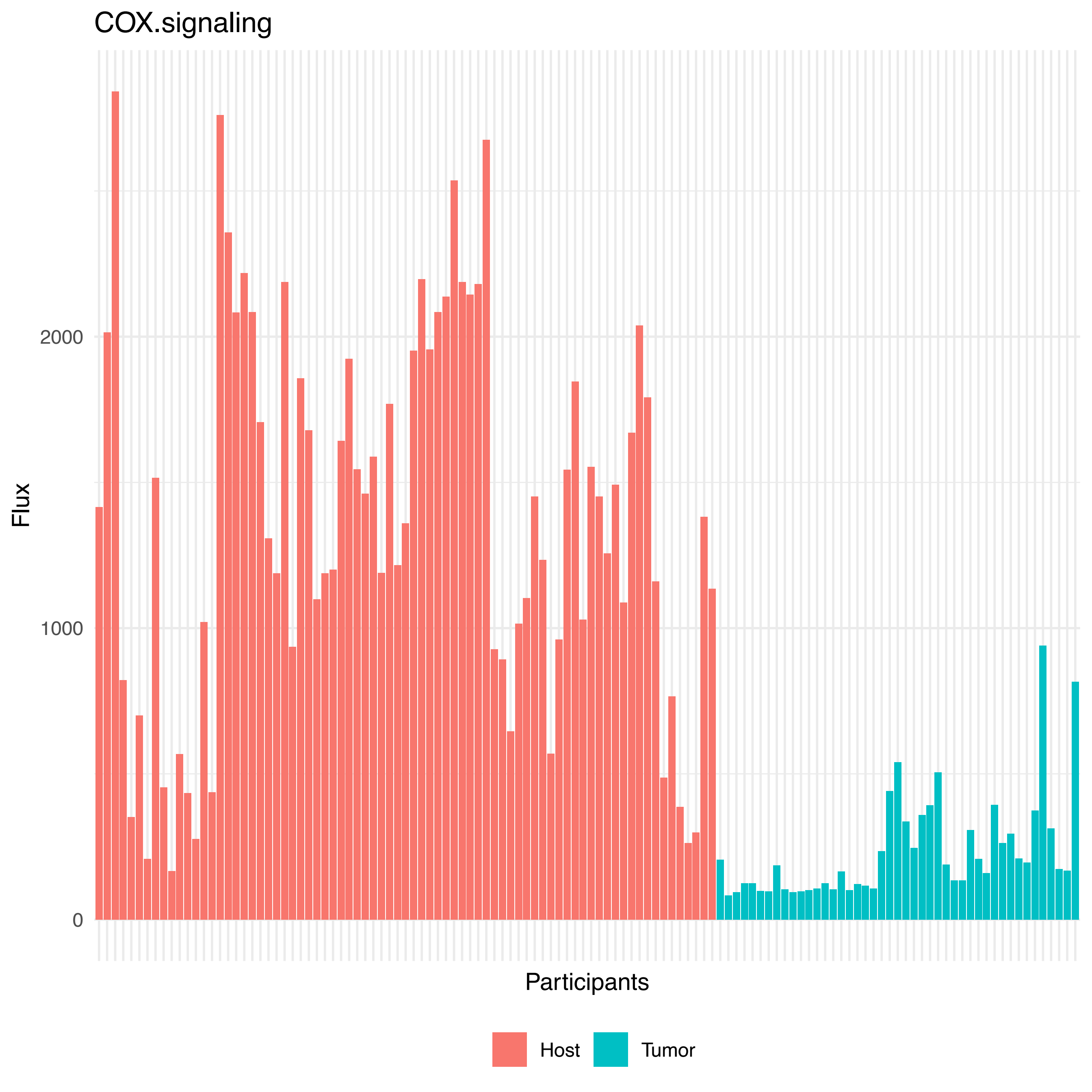

COX.signaling
Flux
2000
1000
0
Participants
Host
Tumor

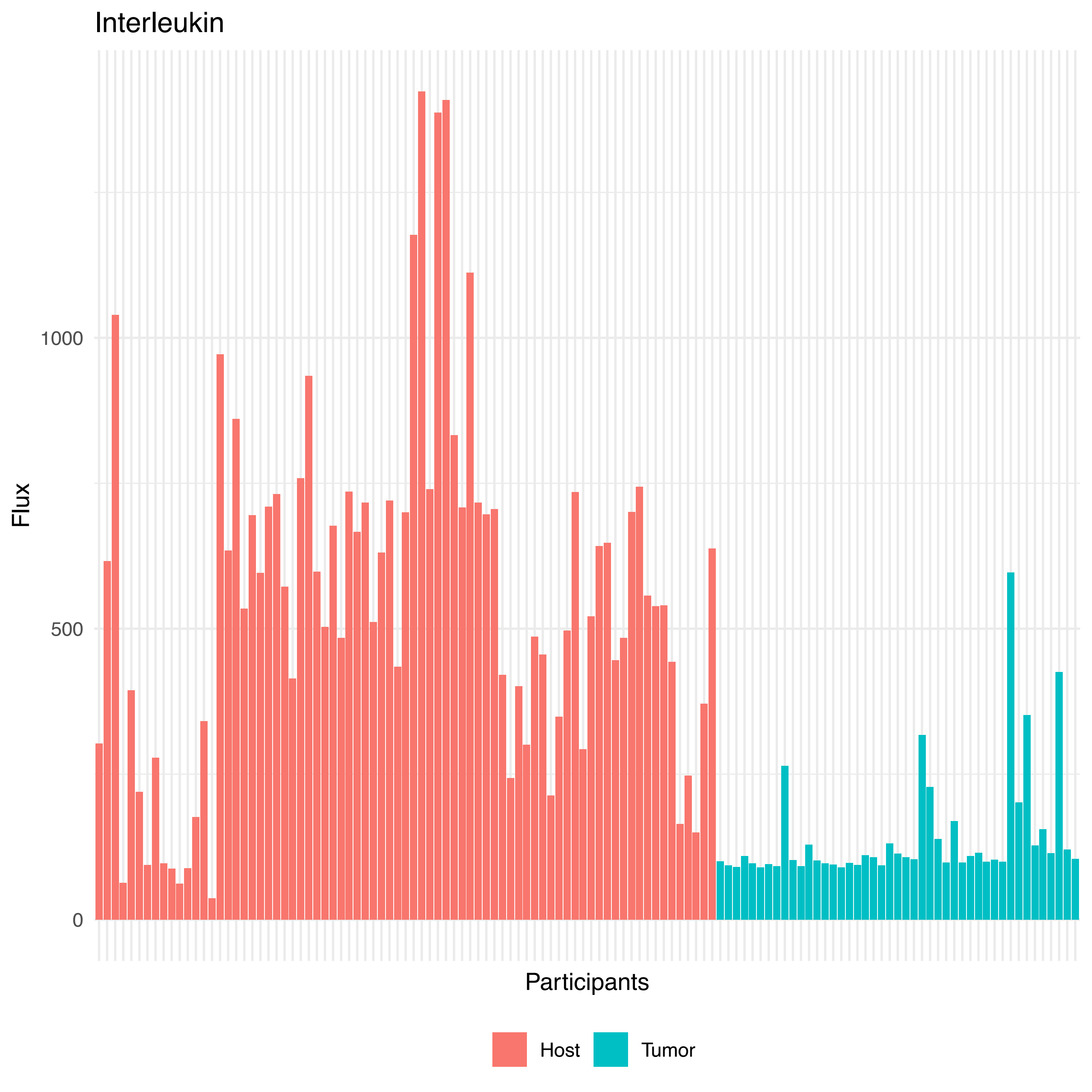

Interleukin
Flux
1000
500
0
Participants
Host
Tumor

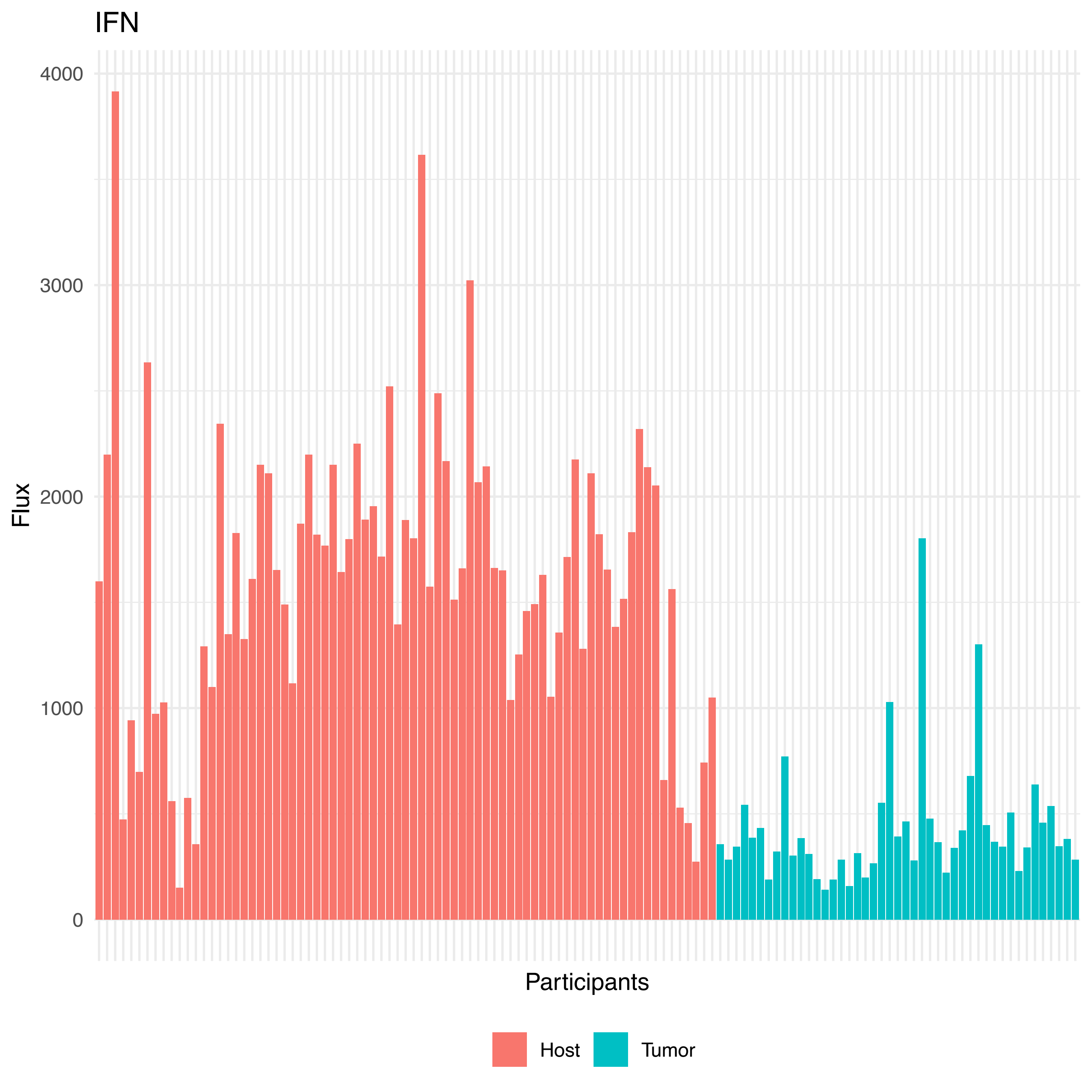
IFN
Flux
0
1000
2000
3000
4000
Participants
Host
Tumor

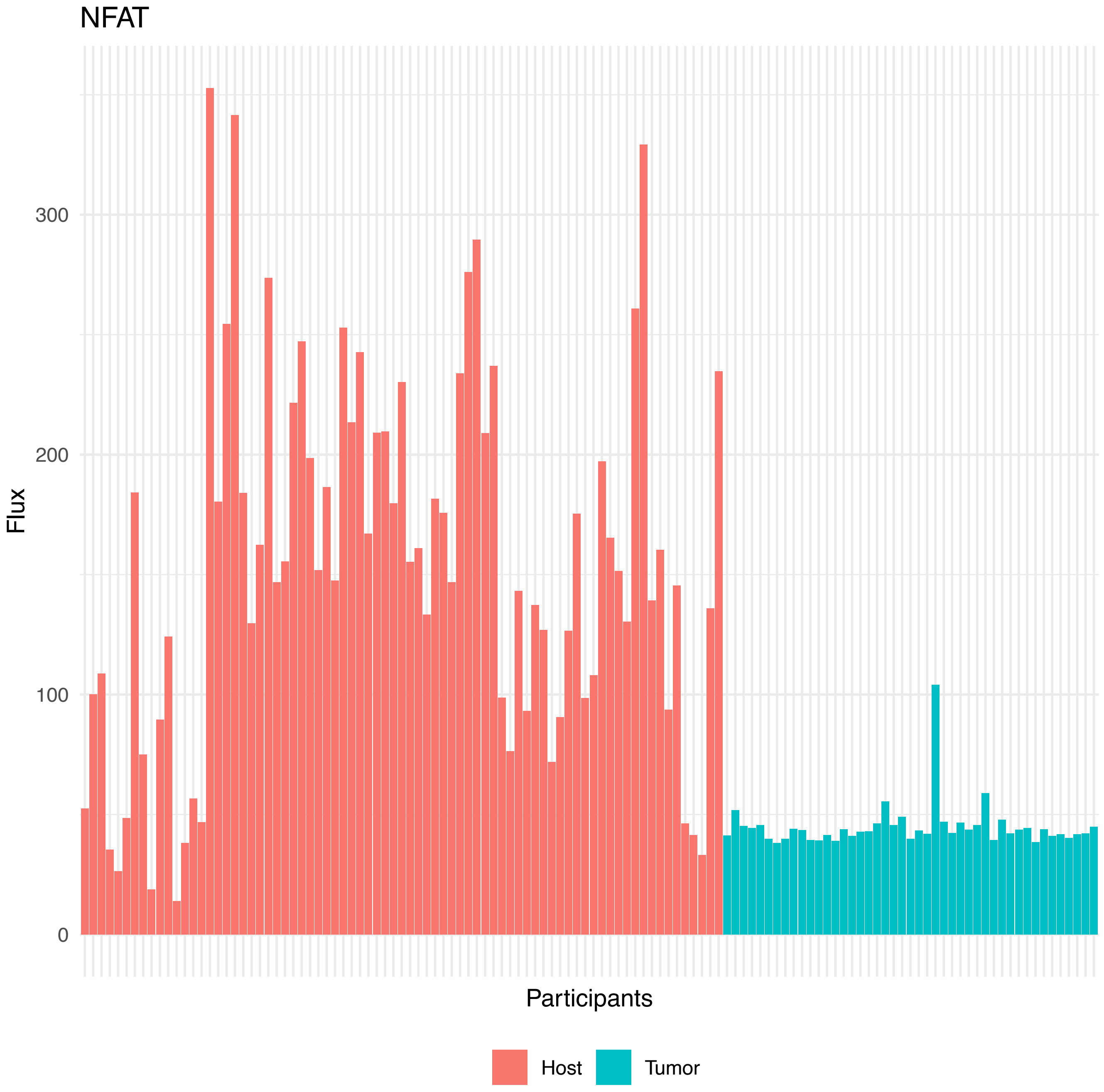

NFAT
300
200
100
0
Flux
Participants
Host
Tumor

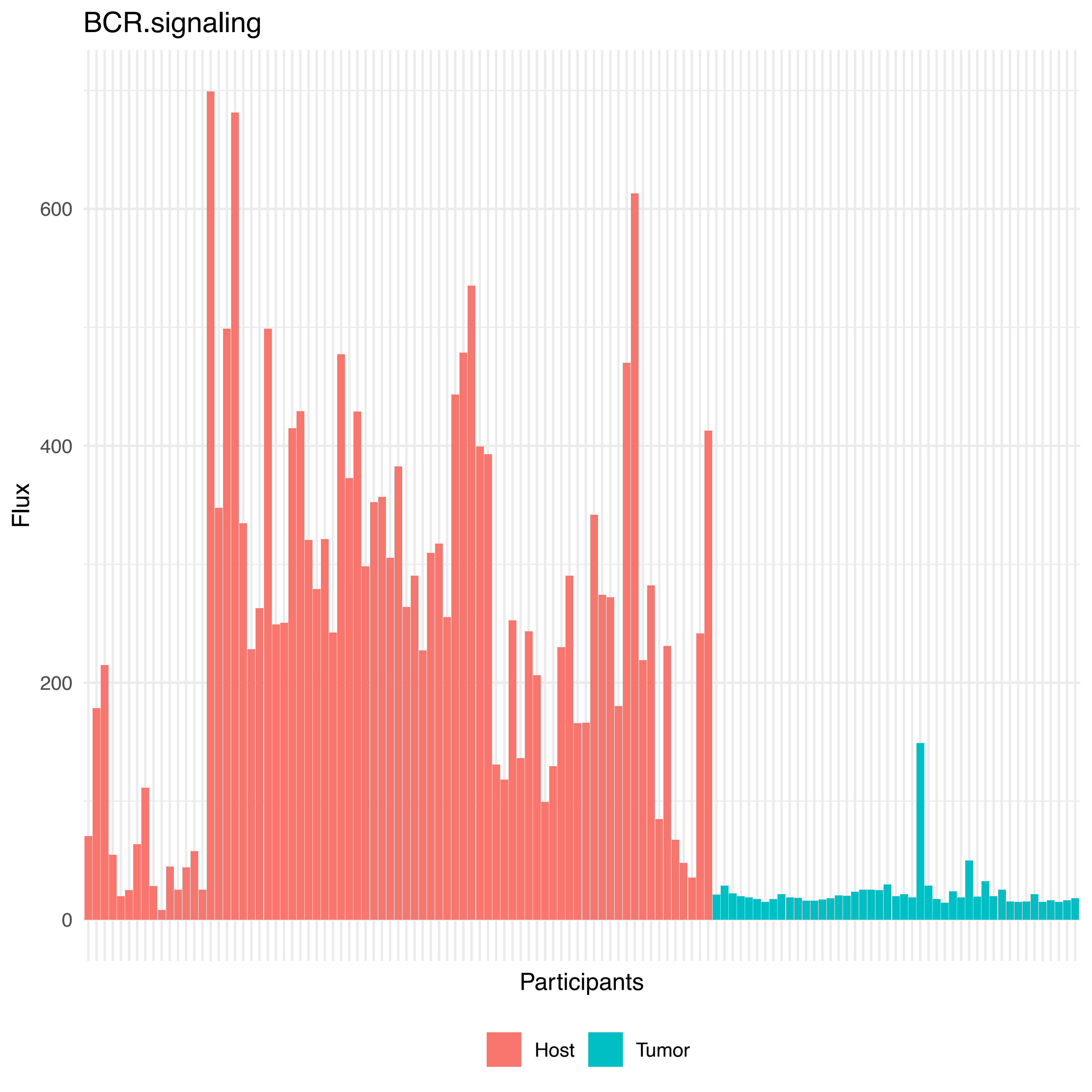
BCR.signaling
Flux
600
400
200
0
Participants
Host
Tumor

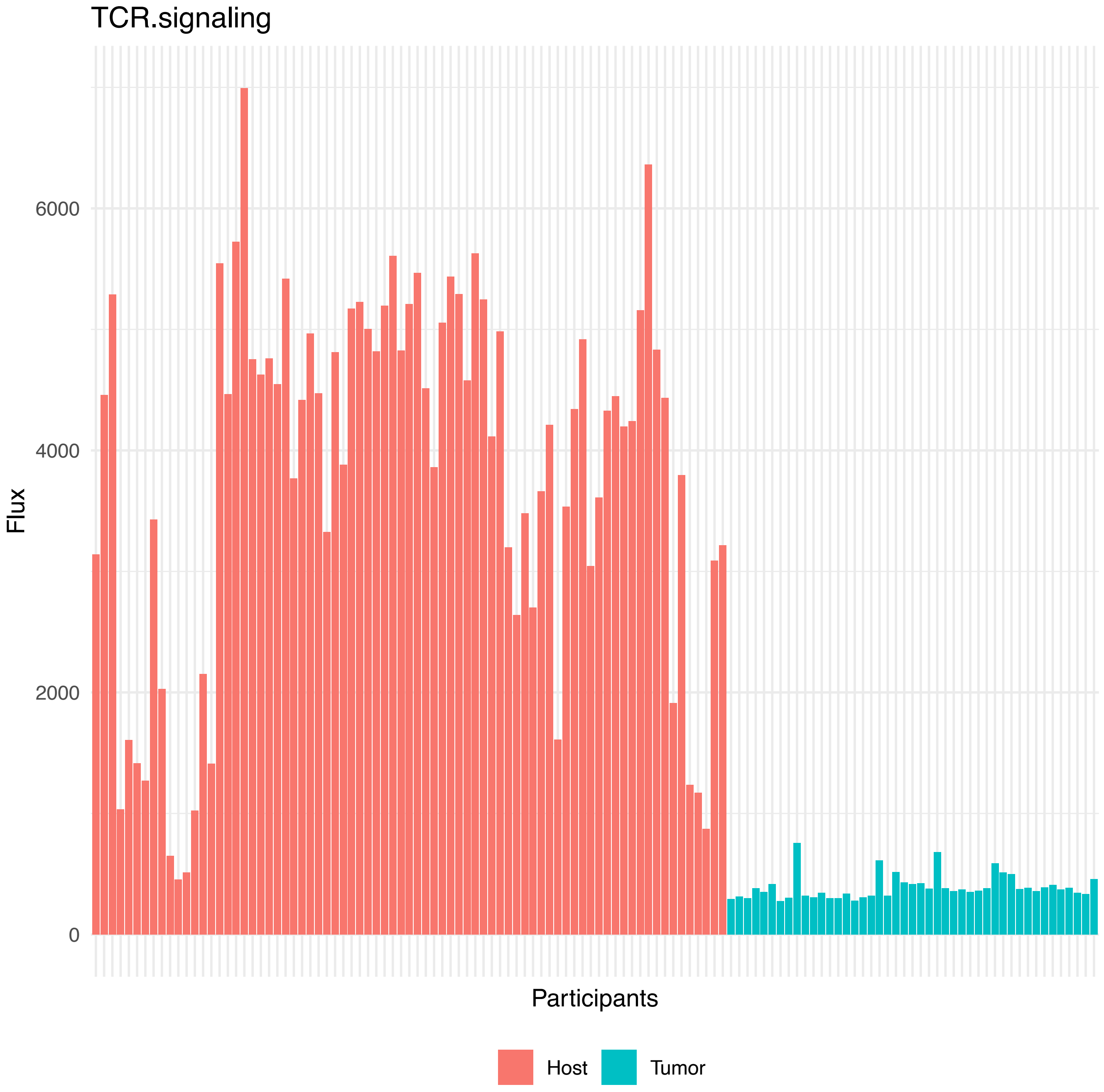
TCR.signaling
6000
4000
2000
0
Flux
Participants
Host
Tumor

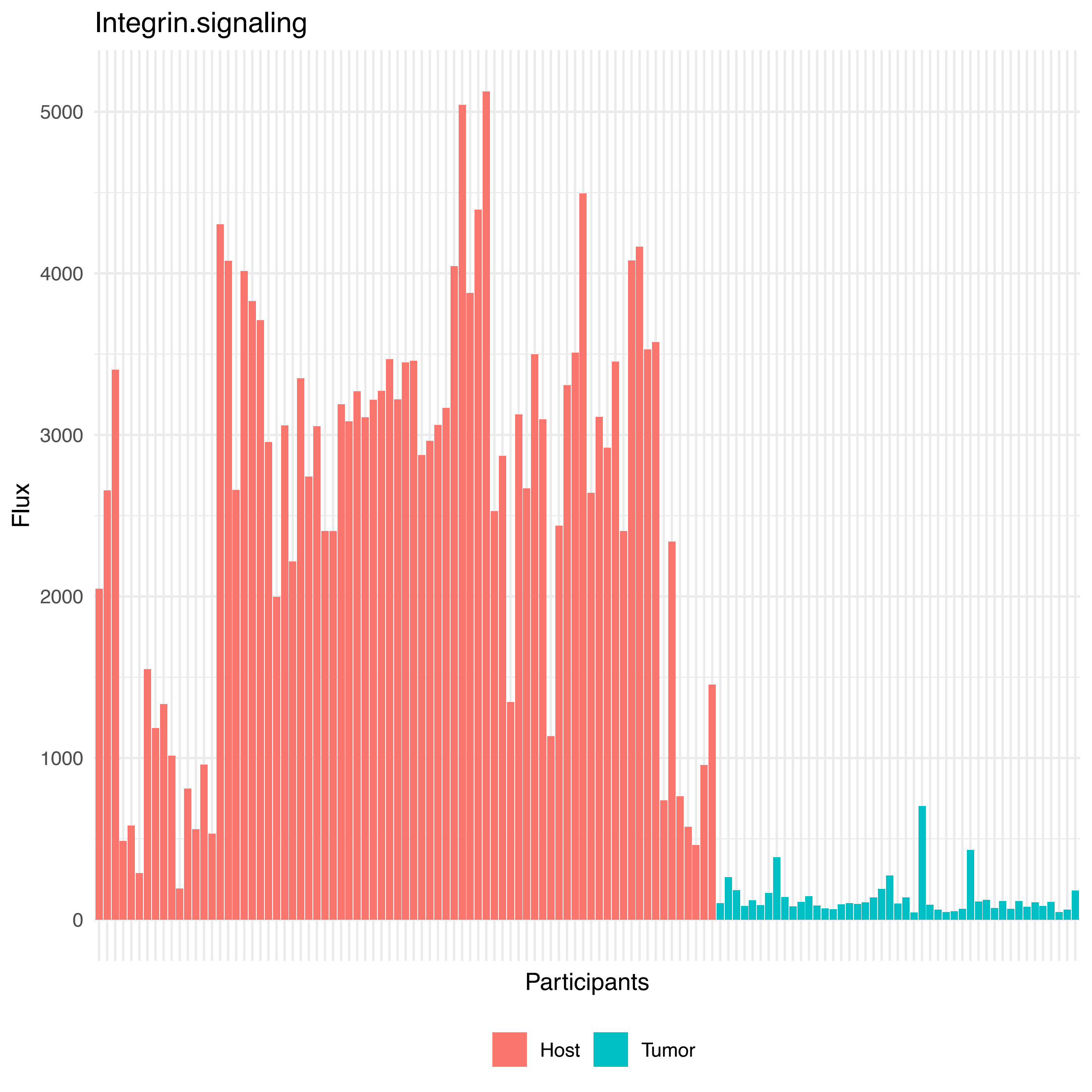
Integrin.signaling
Flux
5000
4000
3000
2000
1000
0
Participants
Host
Tumor

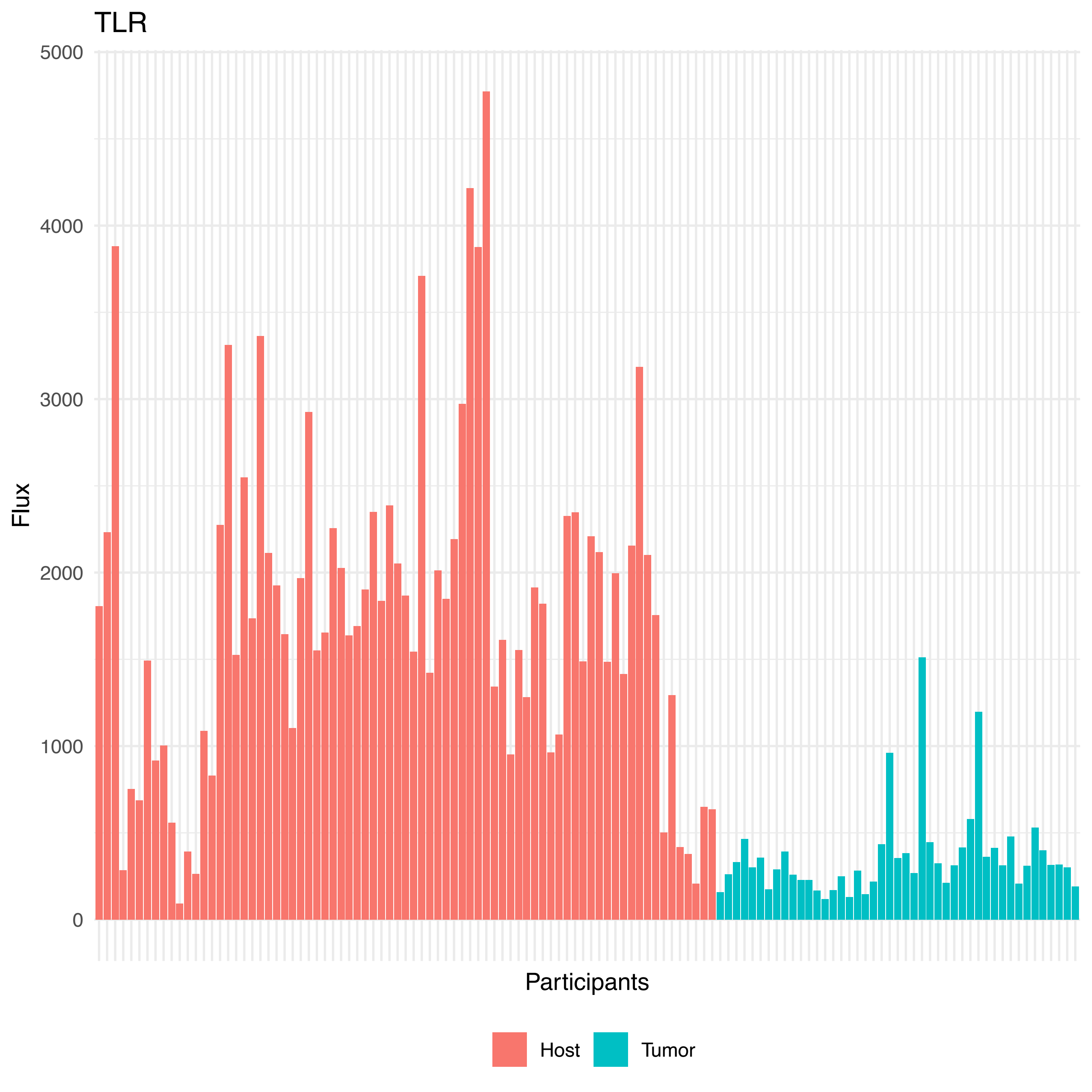
TLR
Flux
5000
4000
3000
2000
1000
0
Participants
Host
Tumor

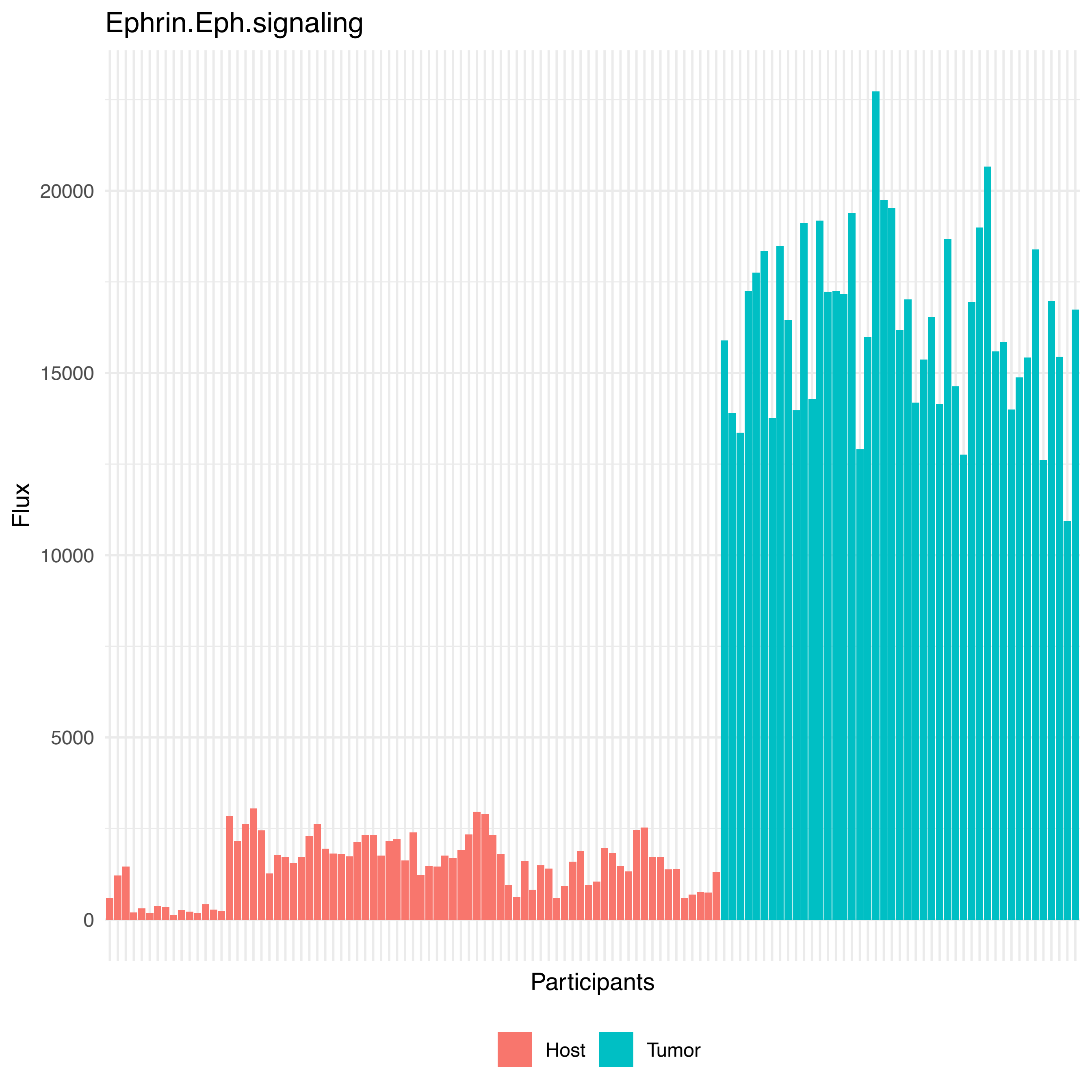

Ephrin.Eph.signaling
Flux
20000
15000
10000
5000
0
Participants
Host
Tumor

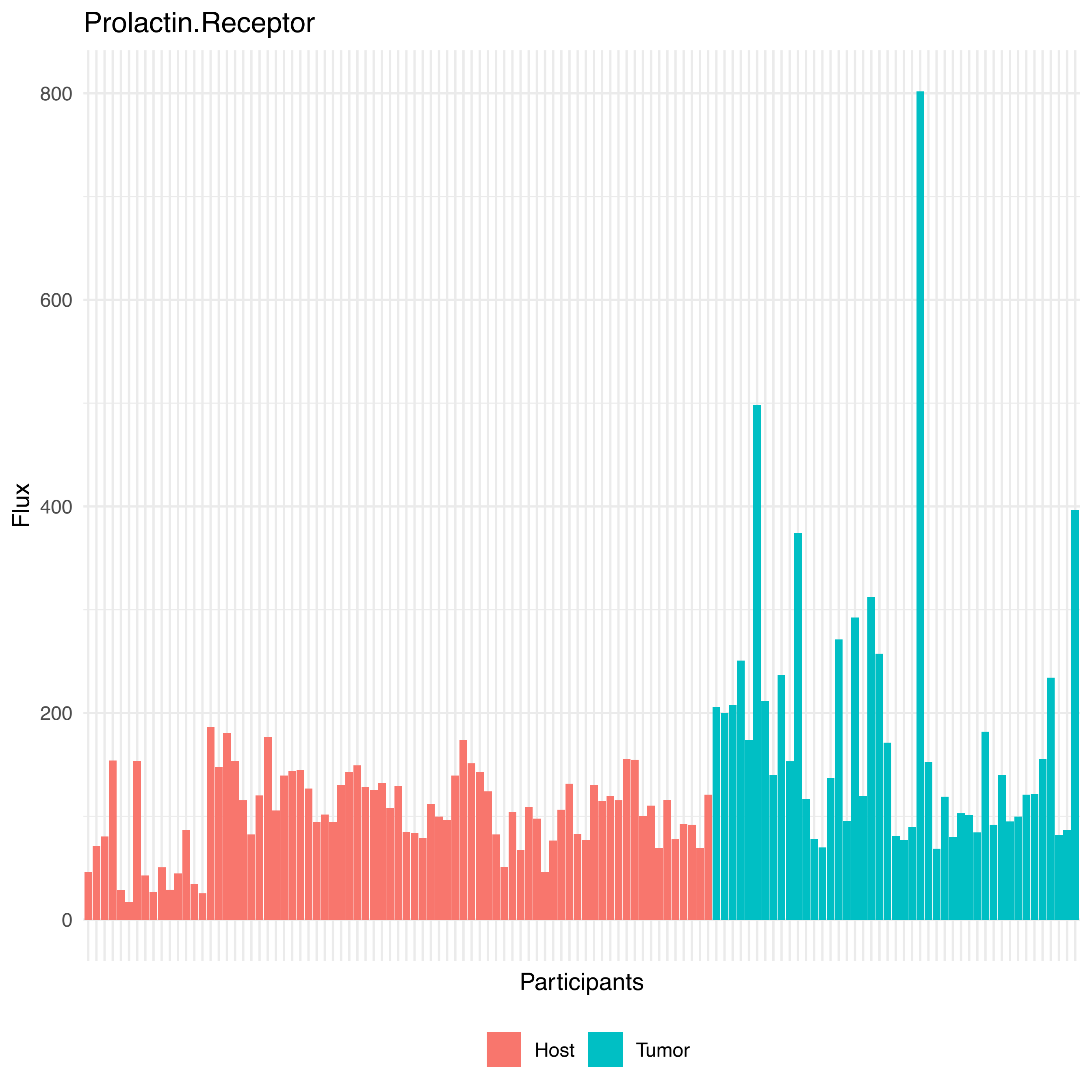

Prolactin.Receptor
800
600
400
200
0
Flux
Participants
Host
Tumor

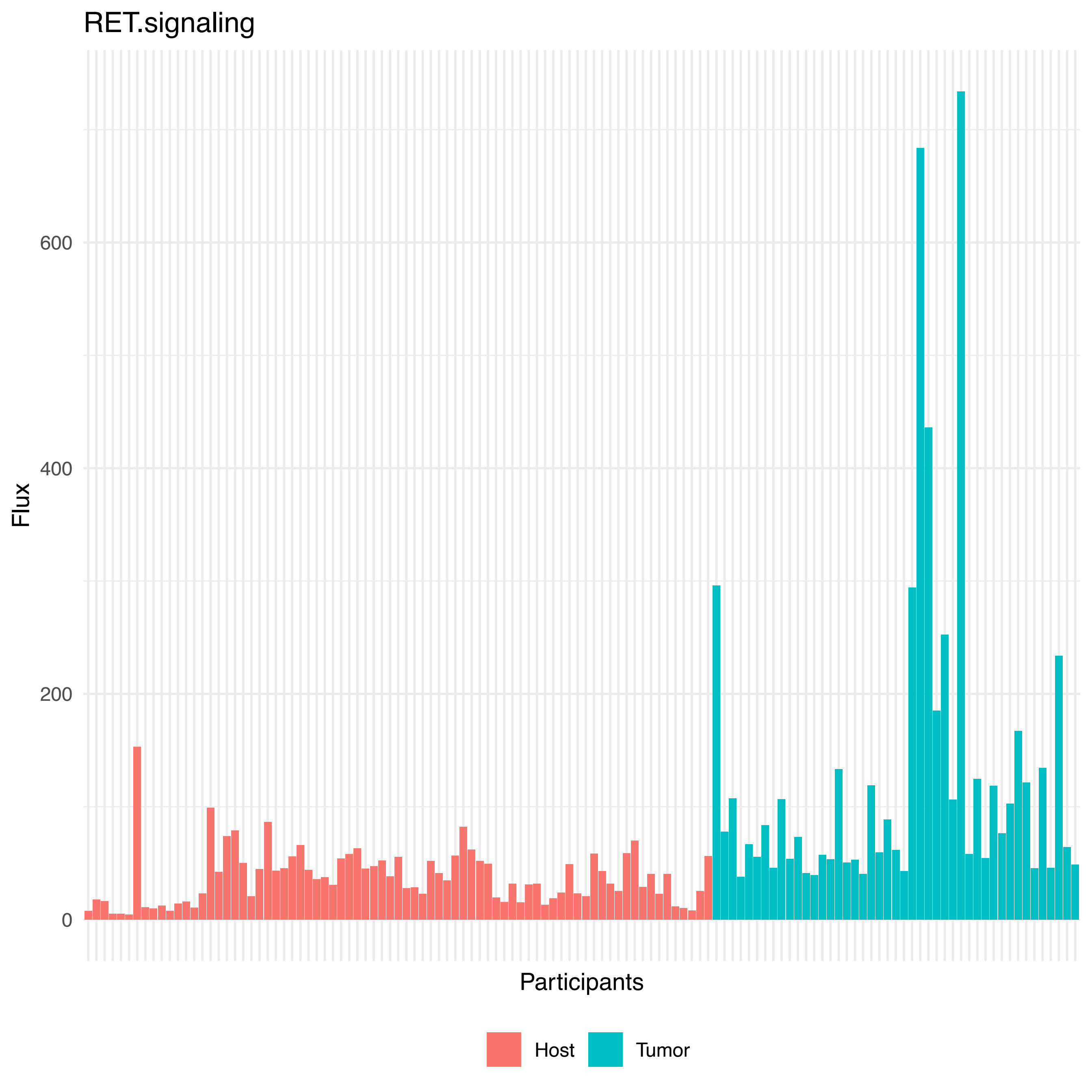

RET.signaling
Flux
600
400
200
0
Participants
Host
Tumor

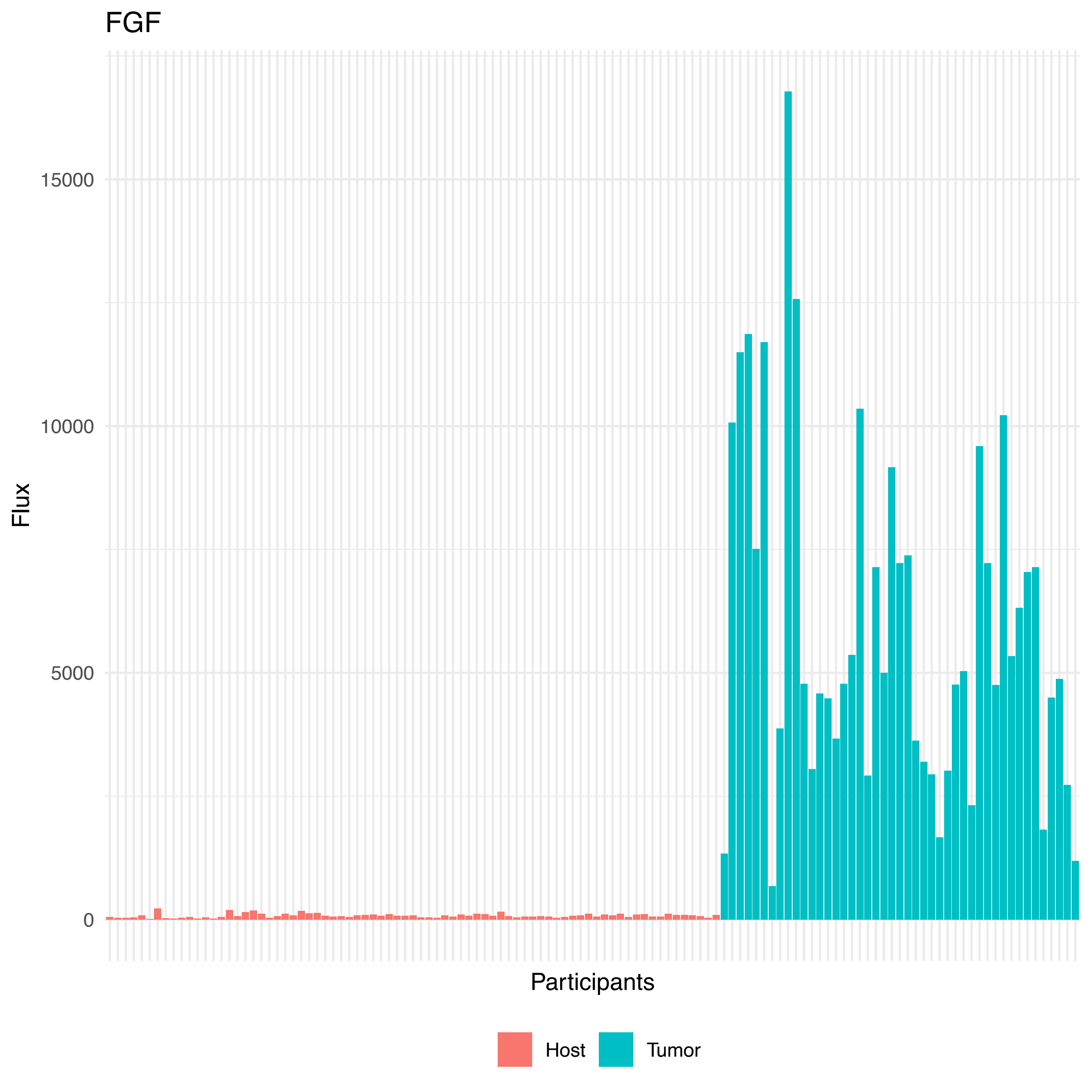

FGF
15000
10000
5000
0
Flux
Participants
Host
Tumor

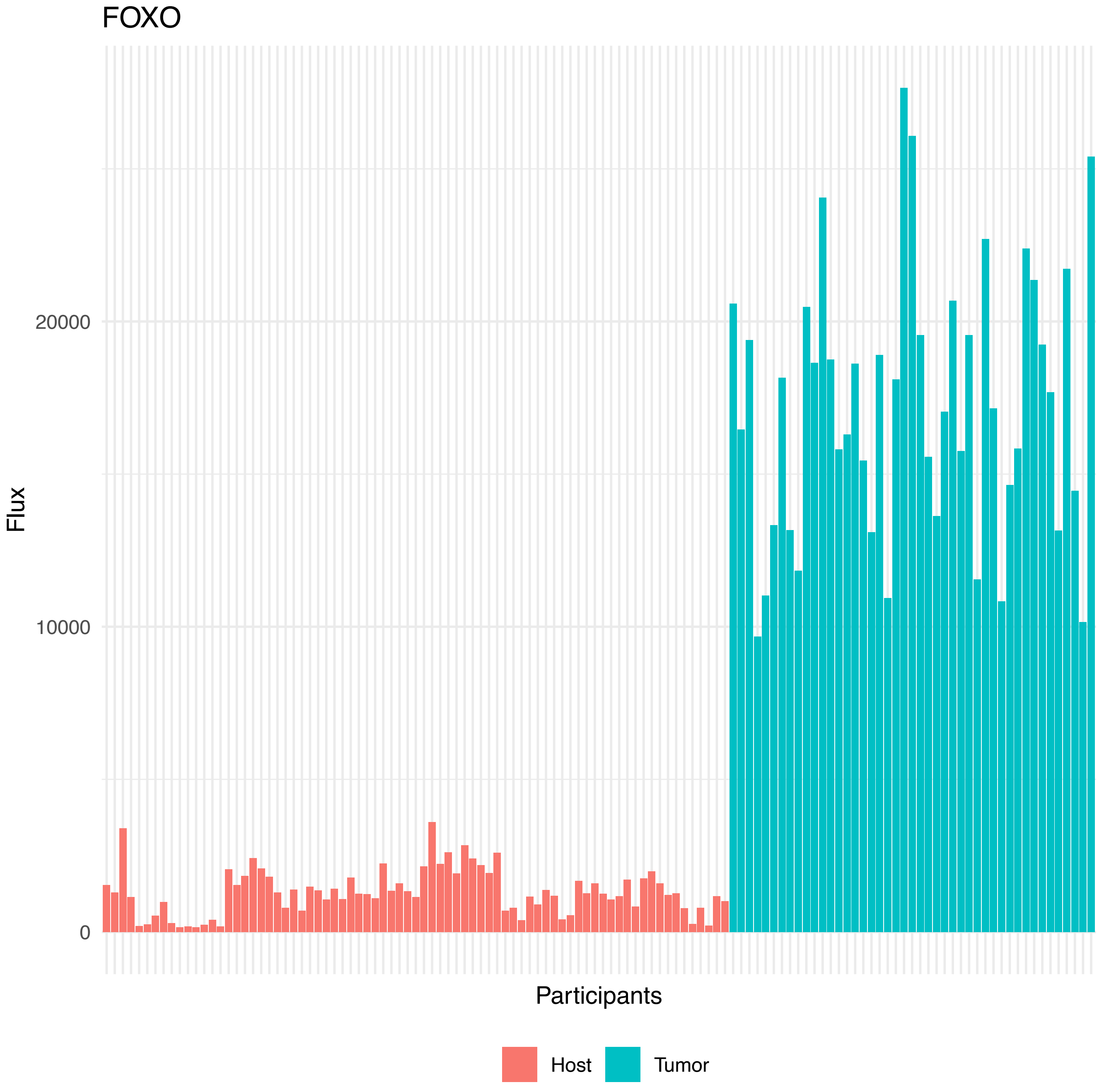

FOXO
Flux
20000
10000
0
Participants
Host
Tumor

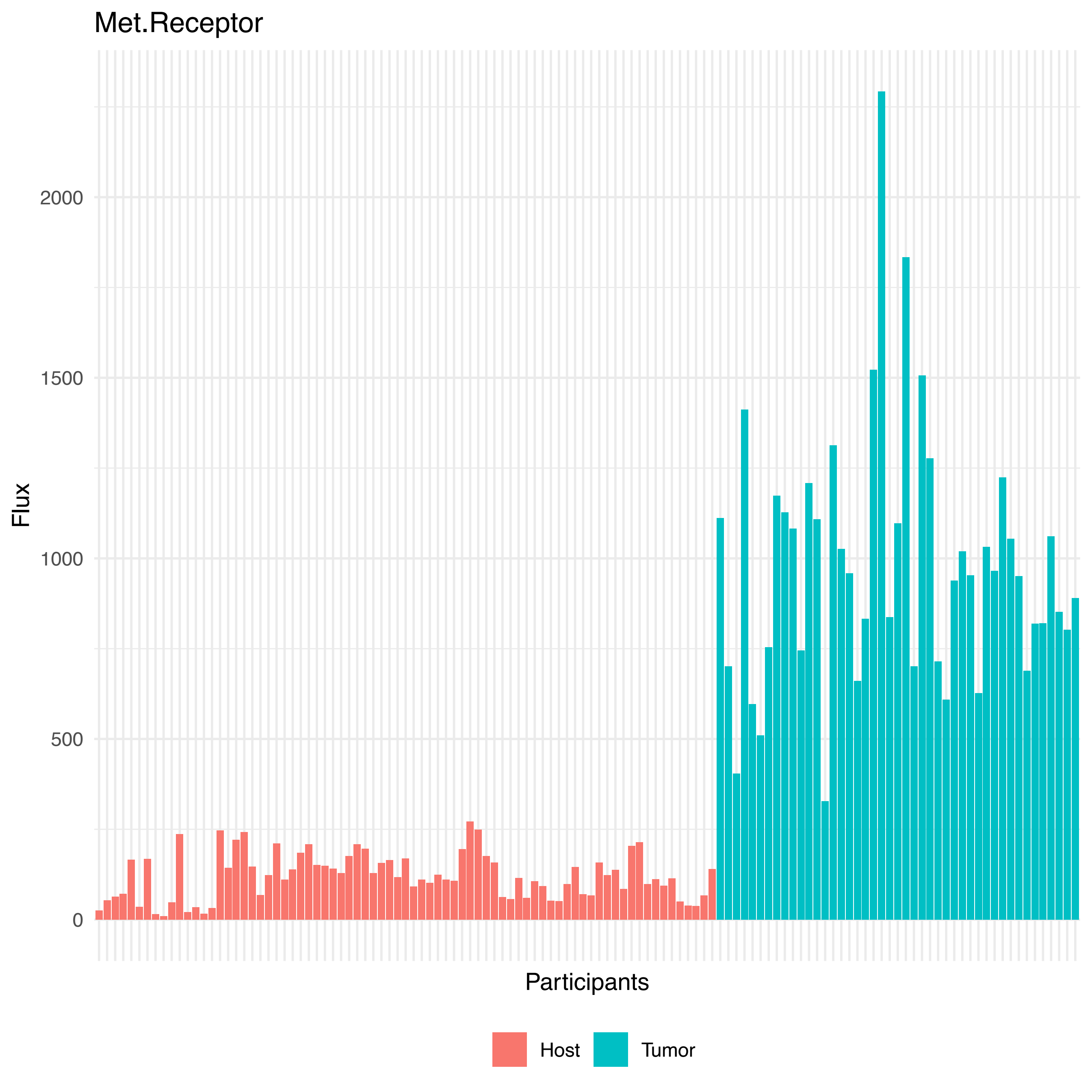
Met.Receptor
Flux
2000
1500
1000
500
0
Participants
Host
Tumor

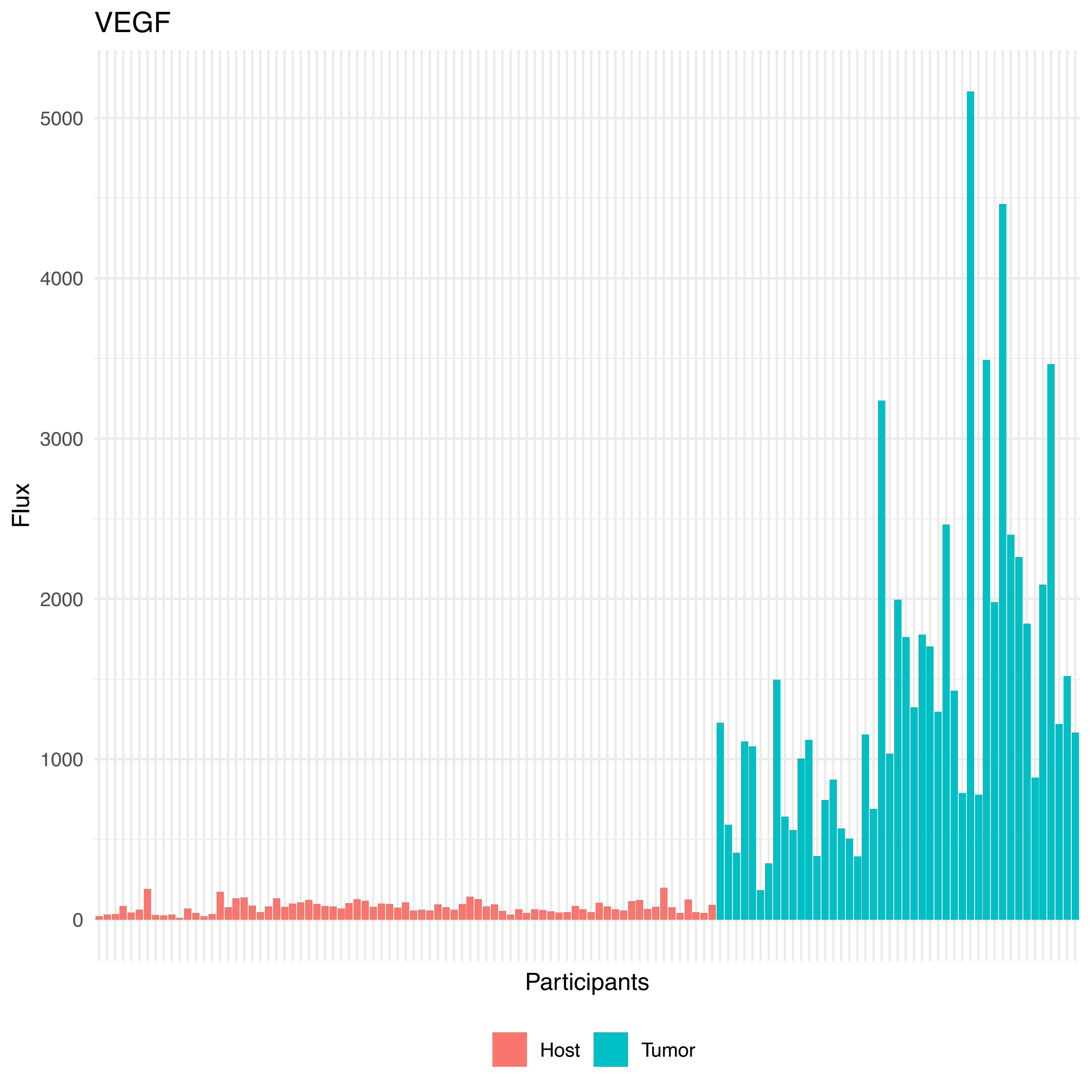

VEGF
Flux
5000
4000
3000
2000
1000
0
Participants
Host
Tumor

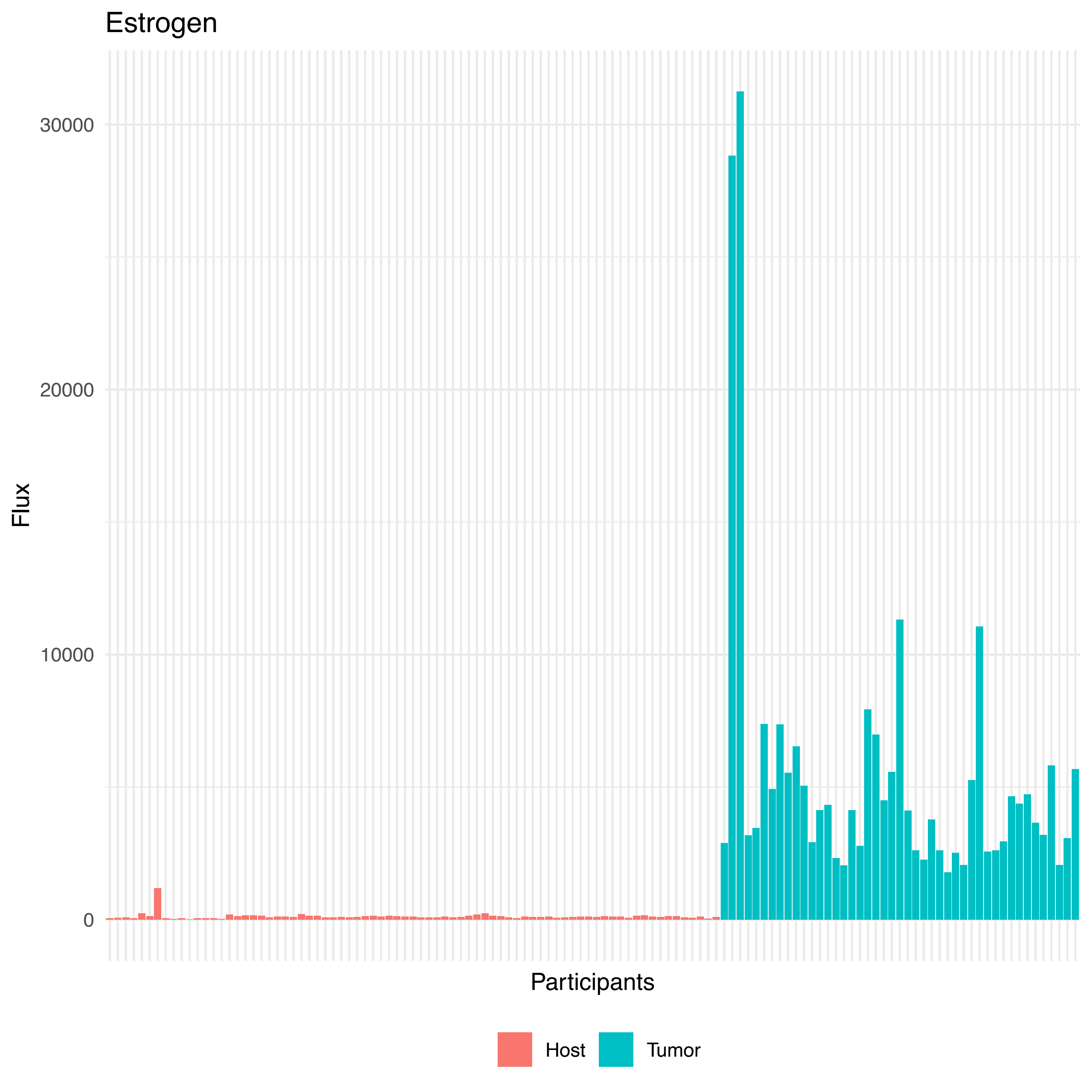
Estrogen
30000
20000
10000
0
Flux
Participants
Host
Tumor

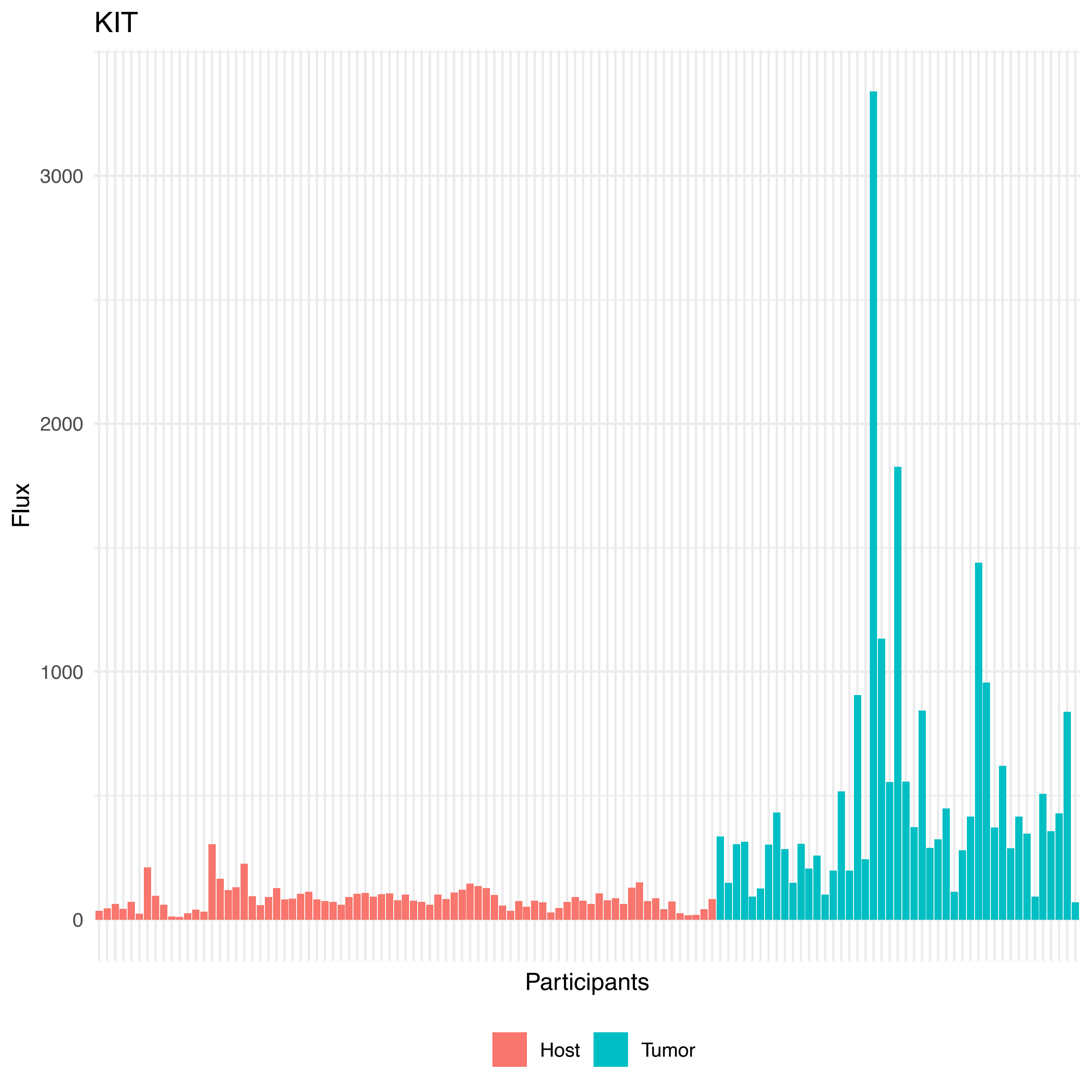
KIT
3000
2000
1000
0
Flux
Participants
Host
Tumor

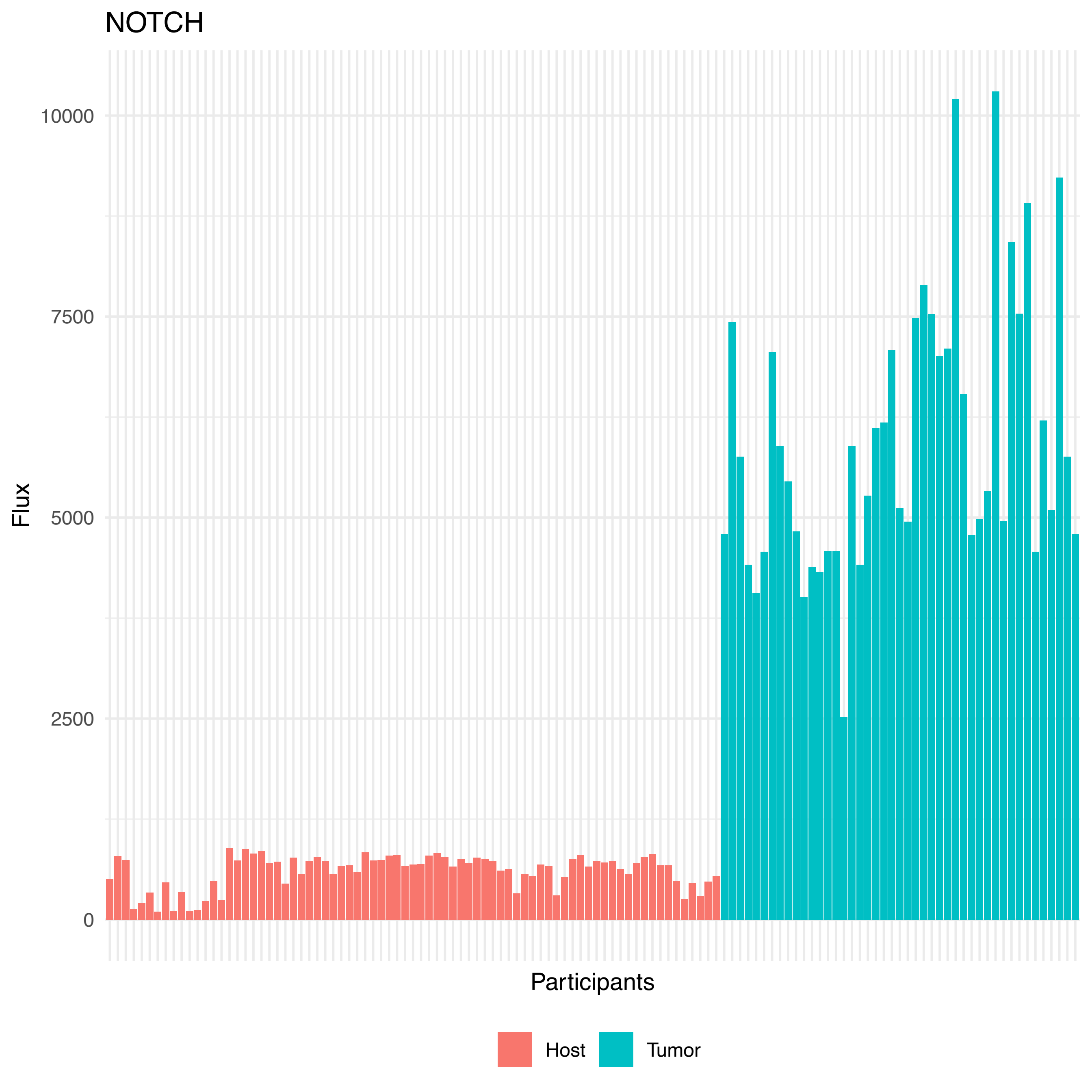

NOTCH
10000
7500
5000
2500
0
Flux
Participants
Host
Tumor

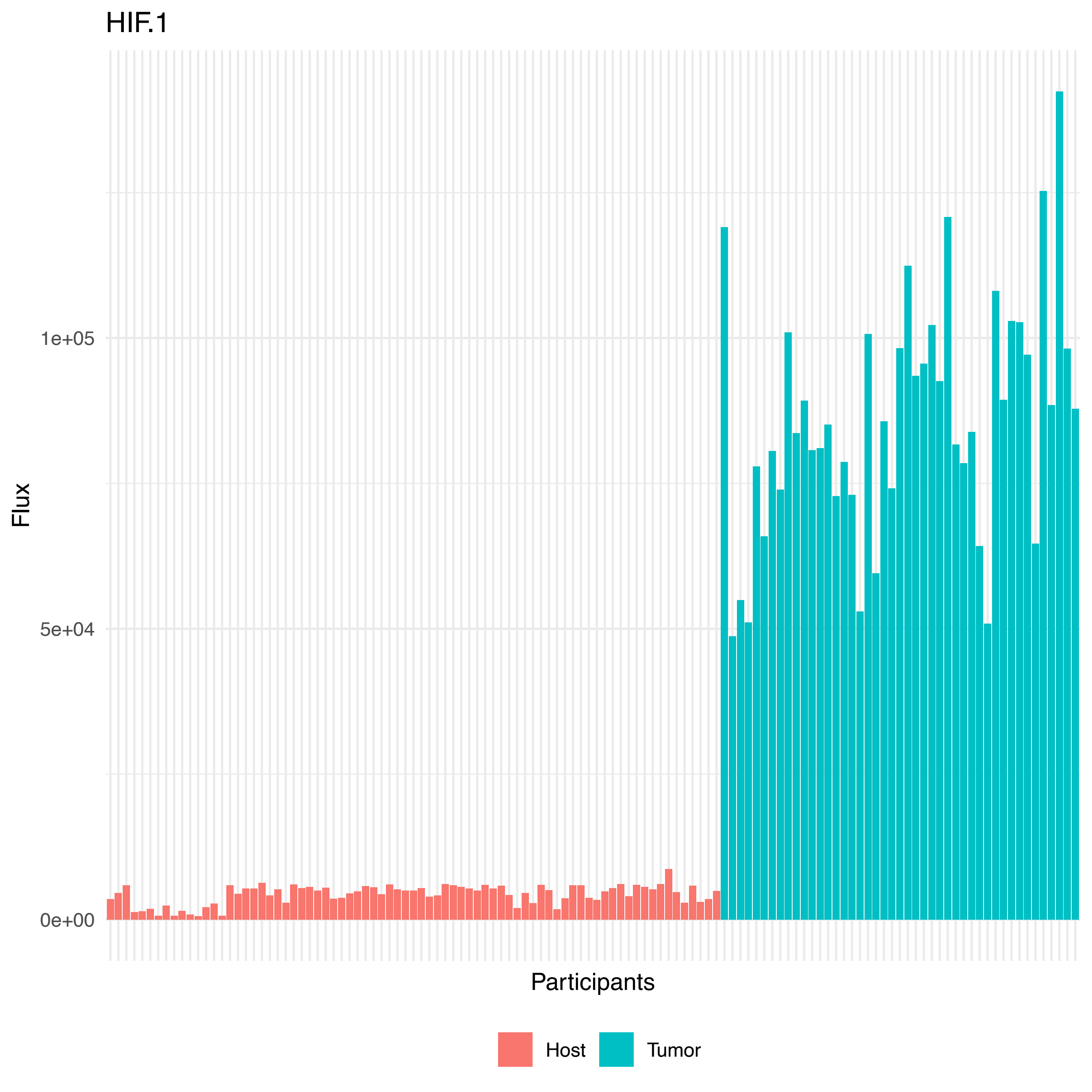

HIF.1
Flux
1e+05
5e+04
0e+00
Participants
Host
Tumor

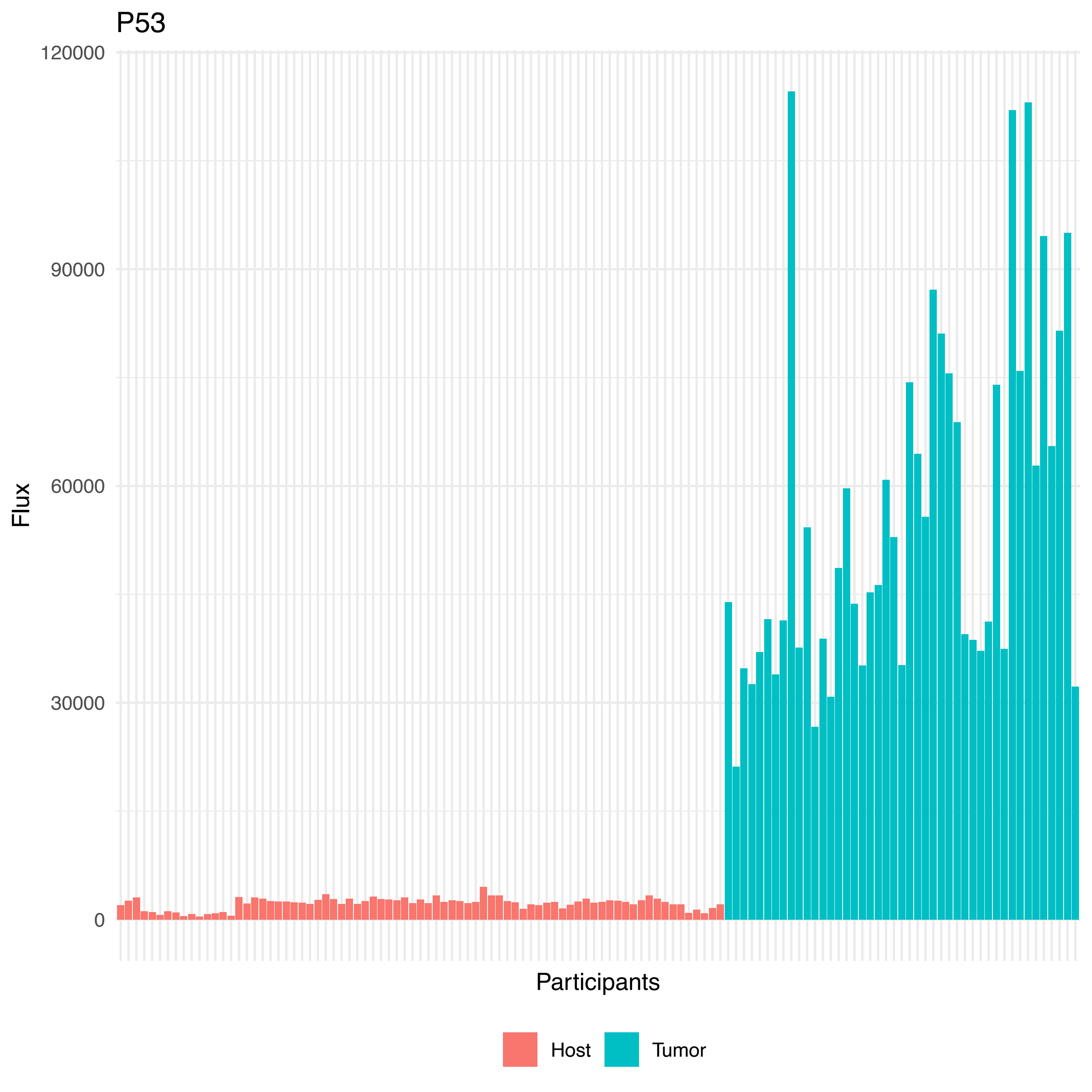

P53
Flux
120000
90000
60000
30000
0
Participants
Host
Tumor

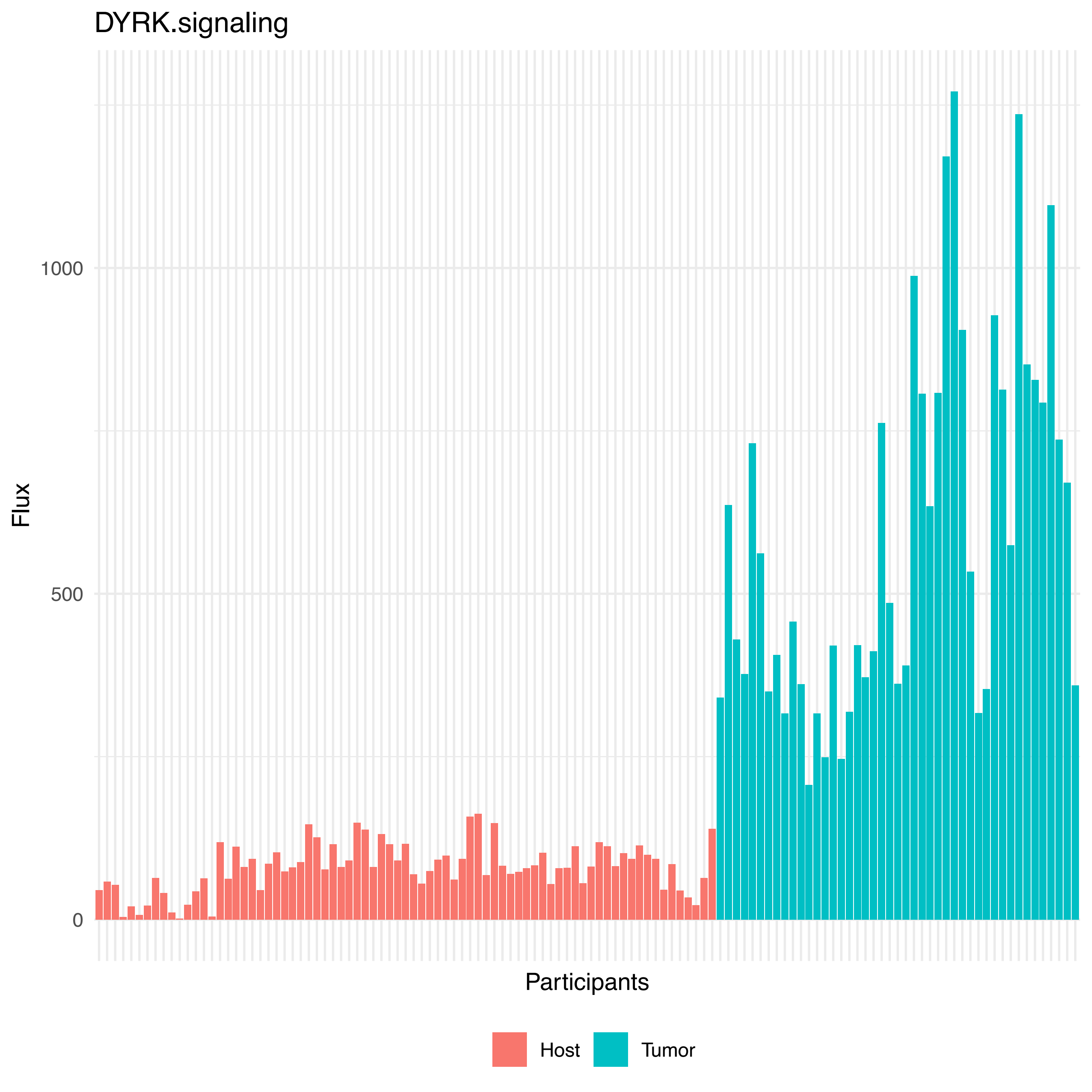
DYRK.signaling
Flux
1000
500
0
Participants
Host
Tumor

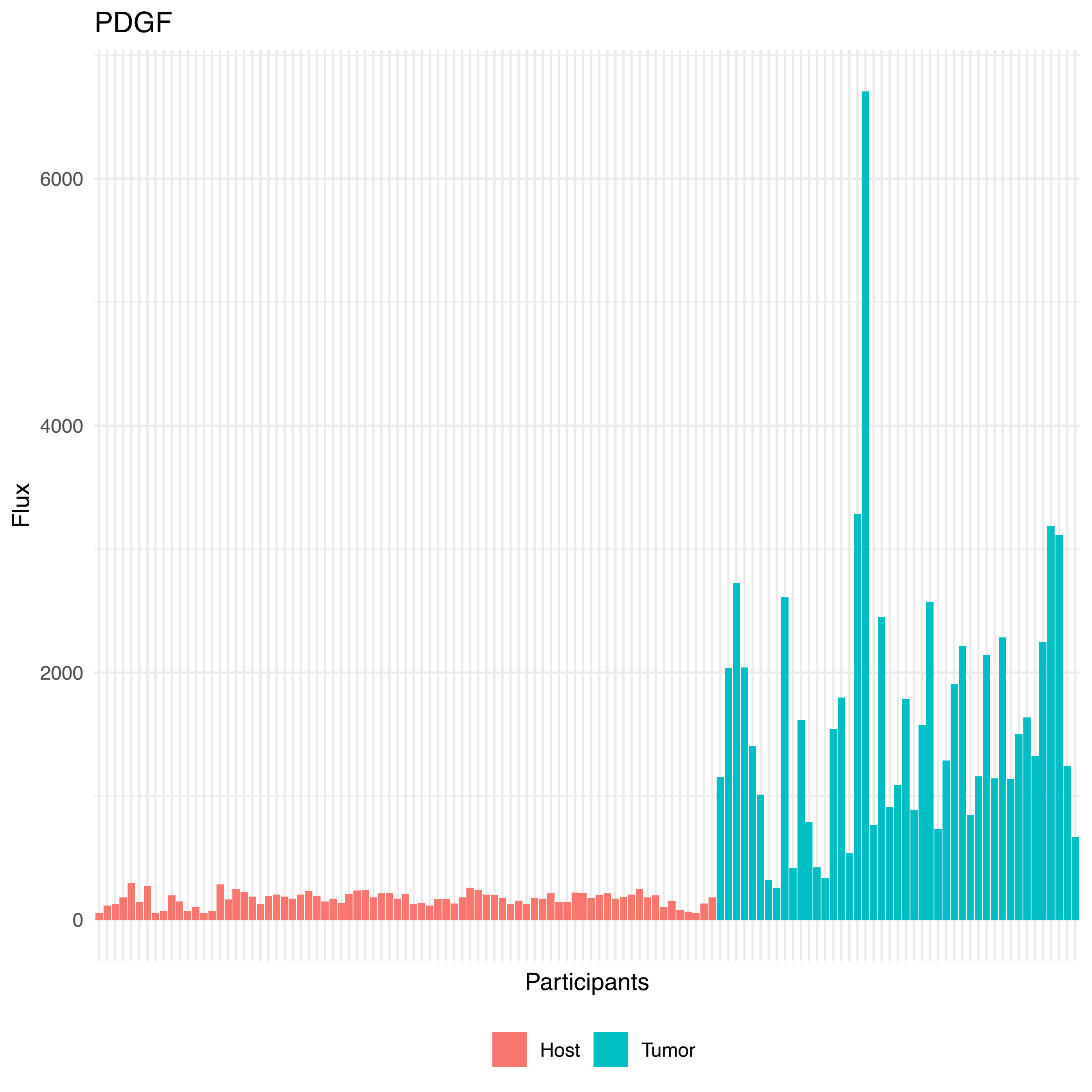

PDGF
6000
4000
2000
0
Flux
Participants
Host
Tumor

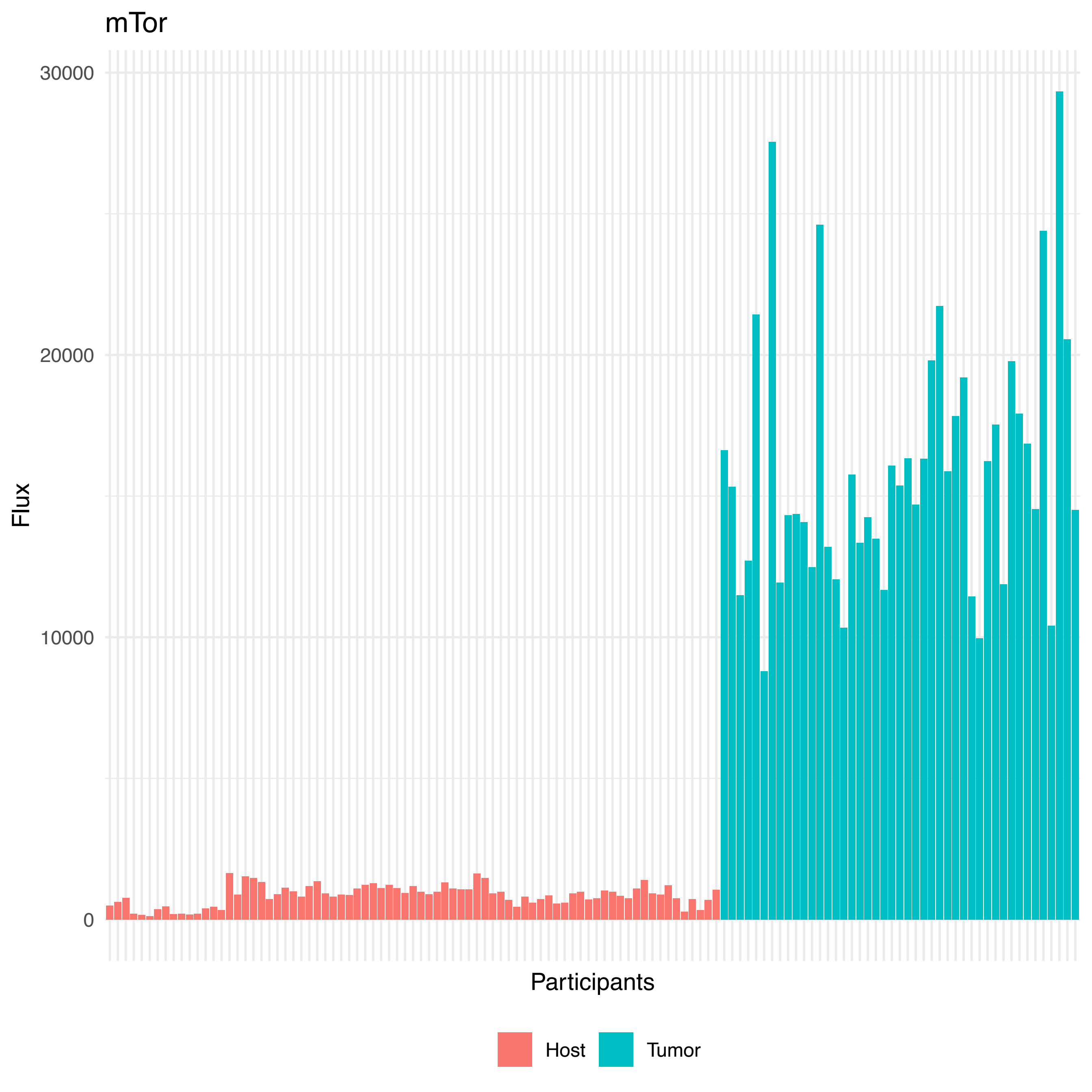

mTor
30000
20000
10000
0
Flux
Participants
Host
Tumor

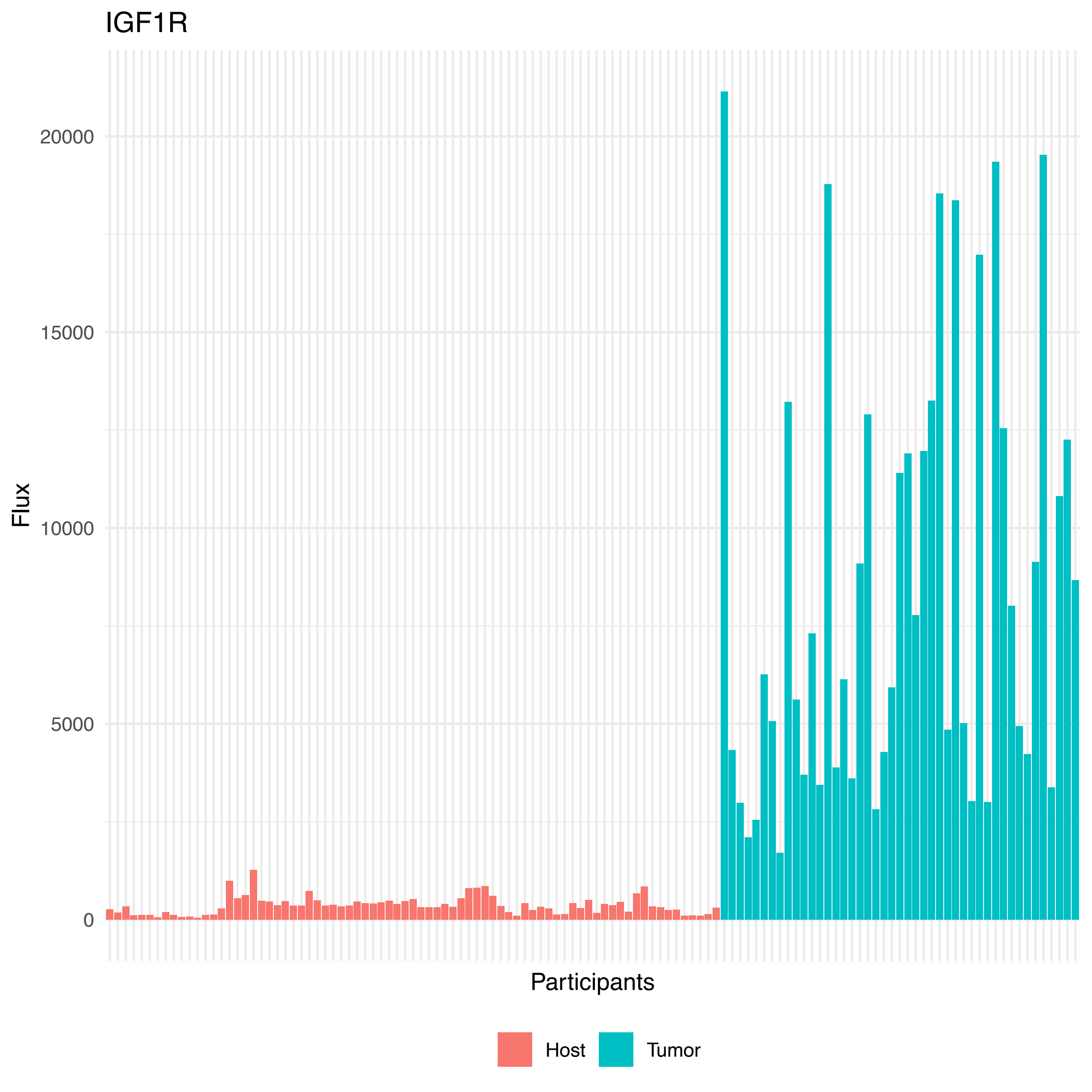
IGF1R
Flux
20000
15000
10000
5000
0
Participants
Host
Tumor

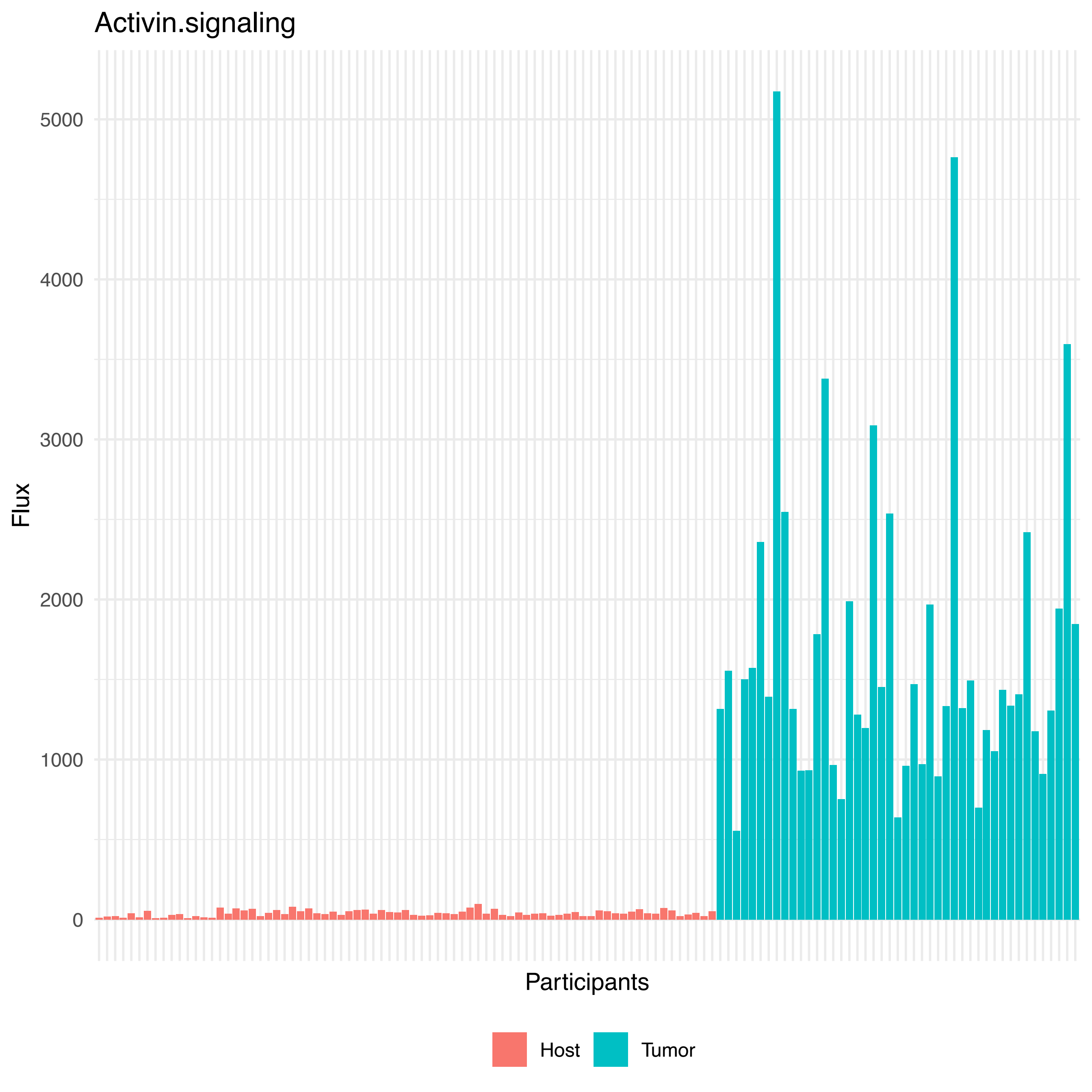
Activin.signaling
Flux
5000
4000
3000
2000
1000
0
Participants
Host
Tumor

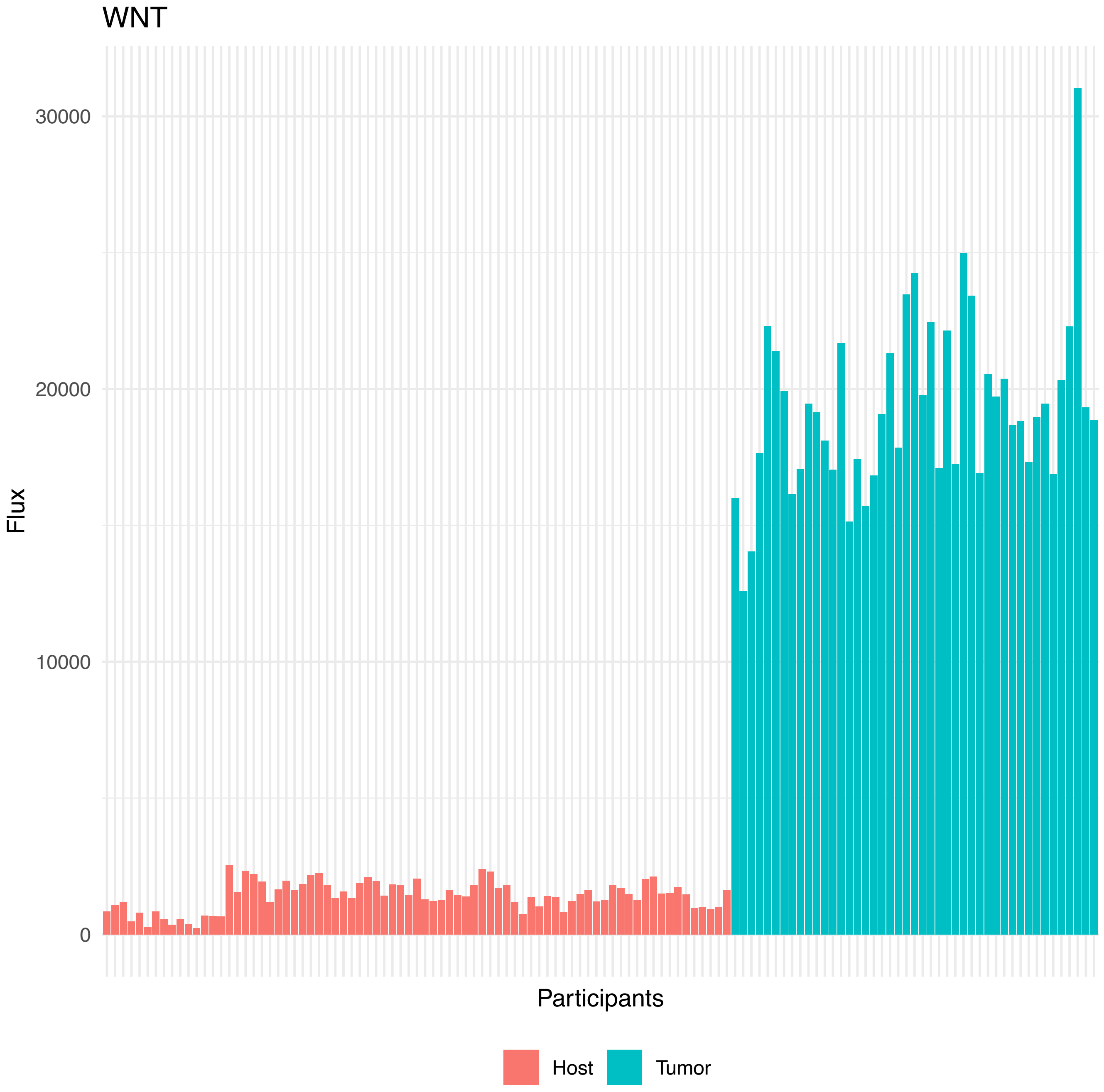

WNT
Flux
30000
20000
10000
0
Participants
Host
Tumor

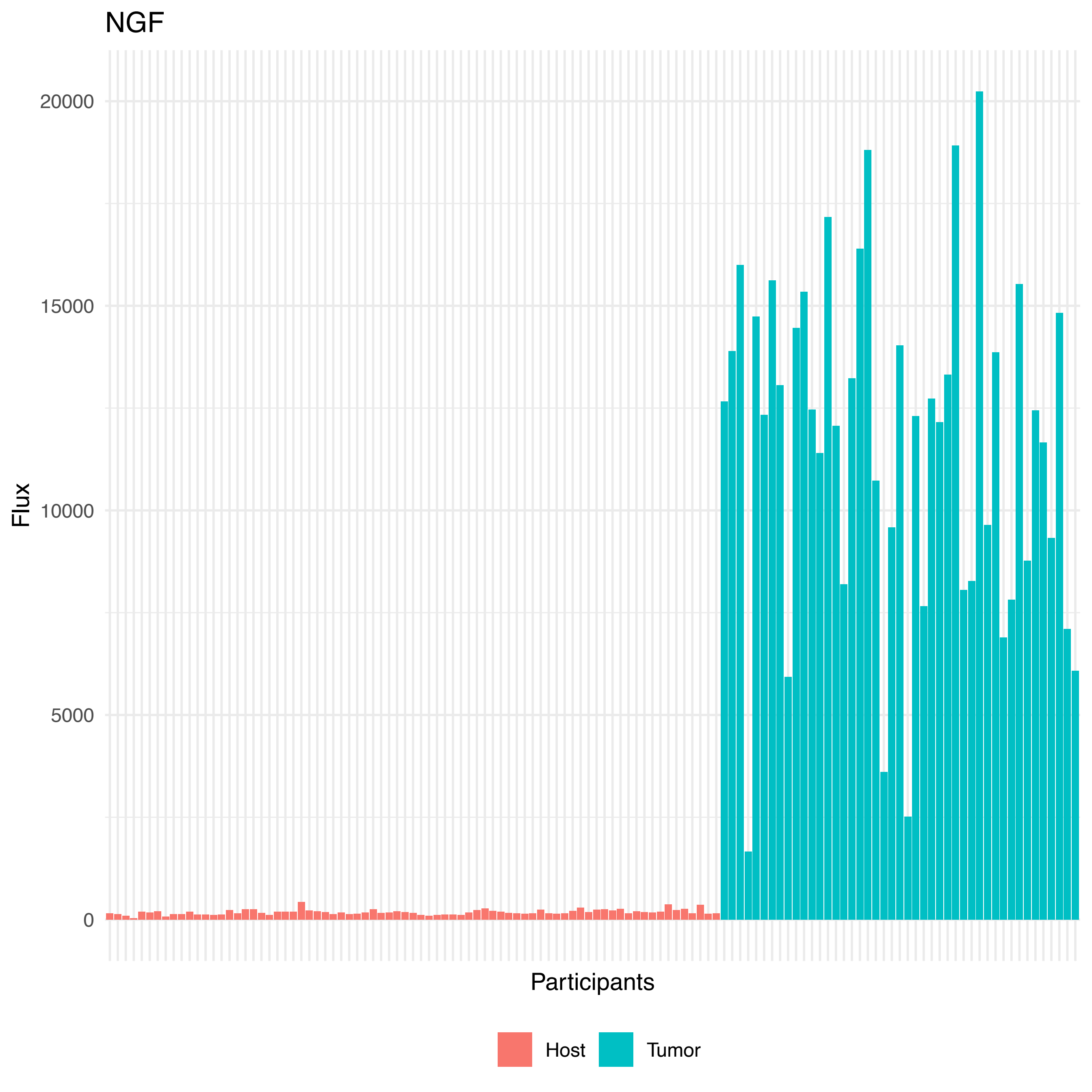

NGF
Flux
20000
15000
10000
5000
0
Participants
Host
Tumor

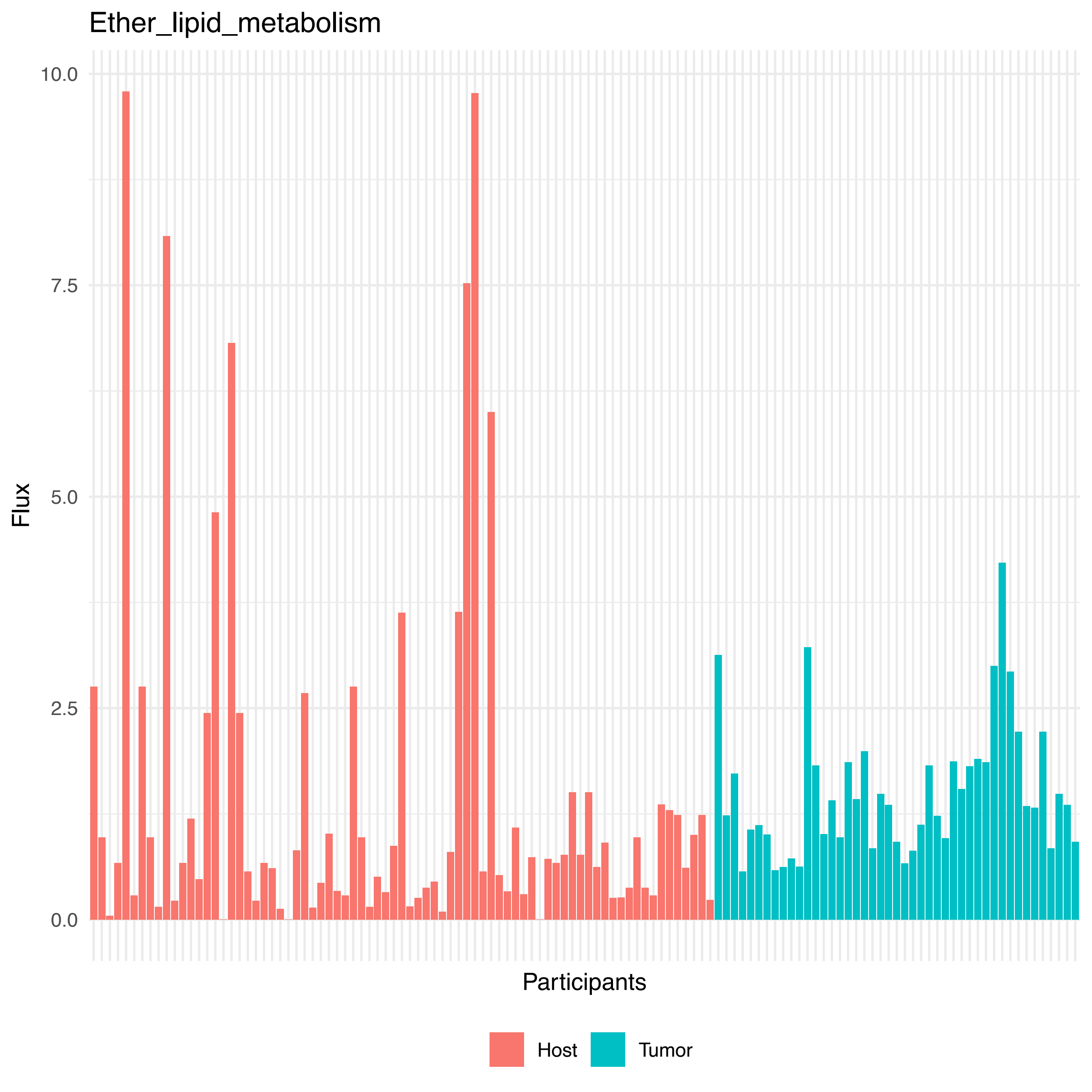

Ether_lipid_metabolism
10.0
7.5
5.0
2.5
0.0
Flux
Participants
Host
Tumor

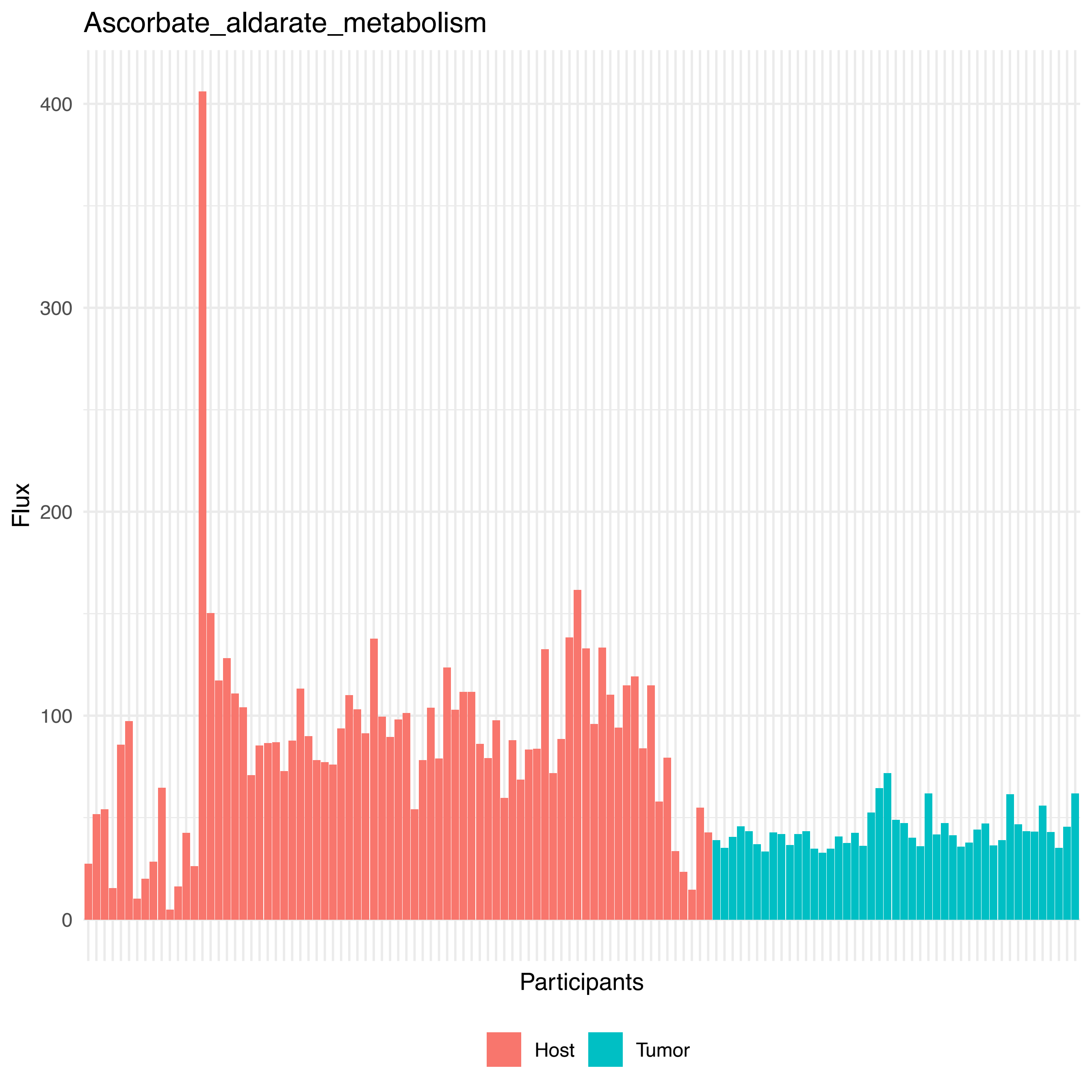

Ascorbate_aldarate_metabolism
400
300
200
100
0
Flux
Participants
Host
Tumor

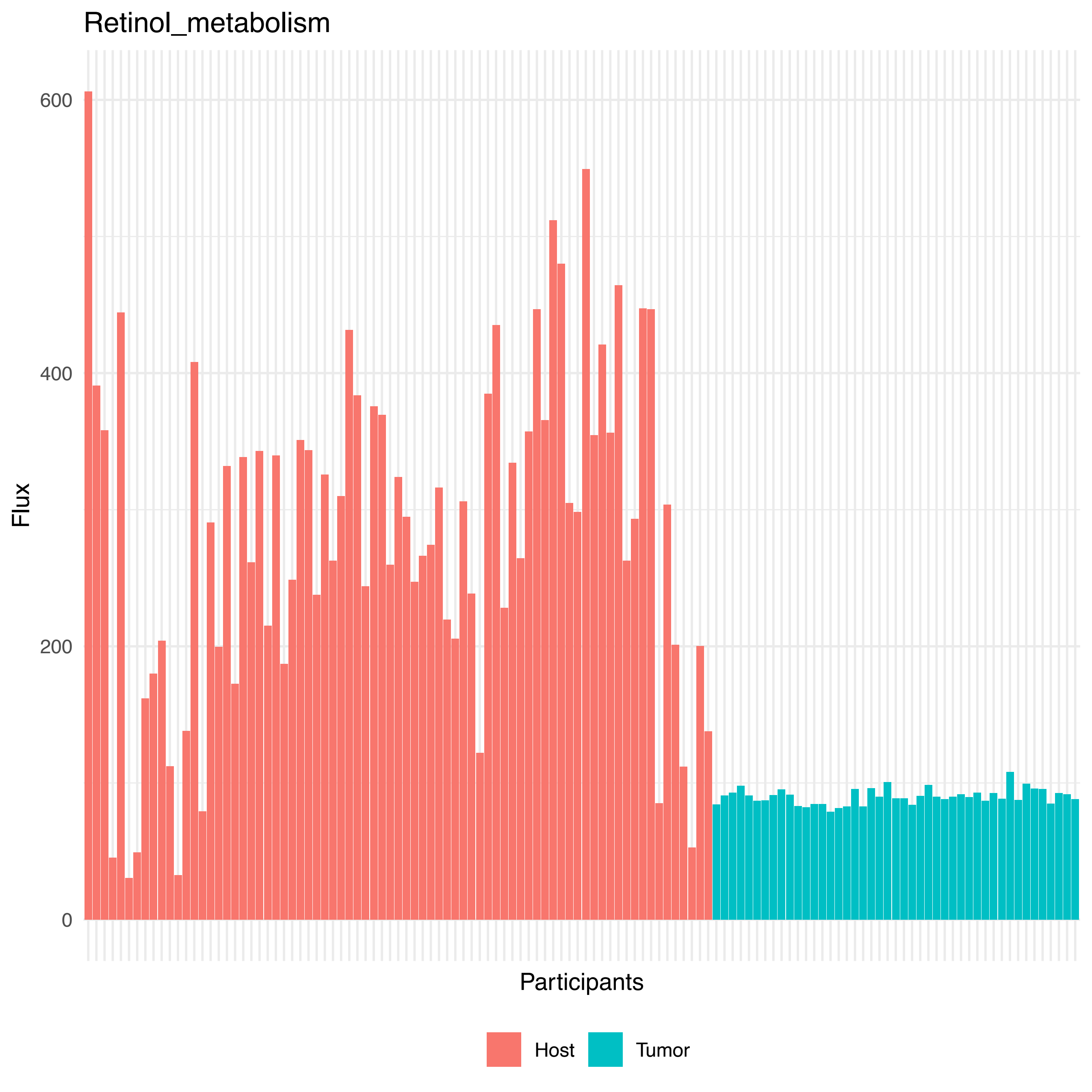

Retinol_metabolism
Flux
600
400
200
0
Participants
Host
Tumor

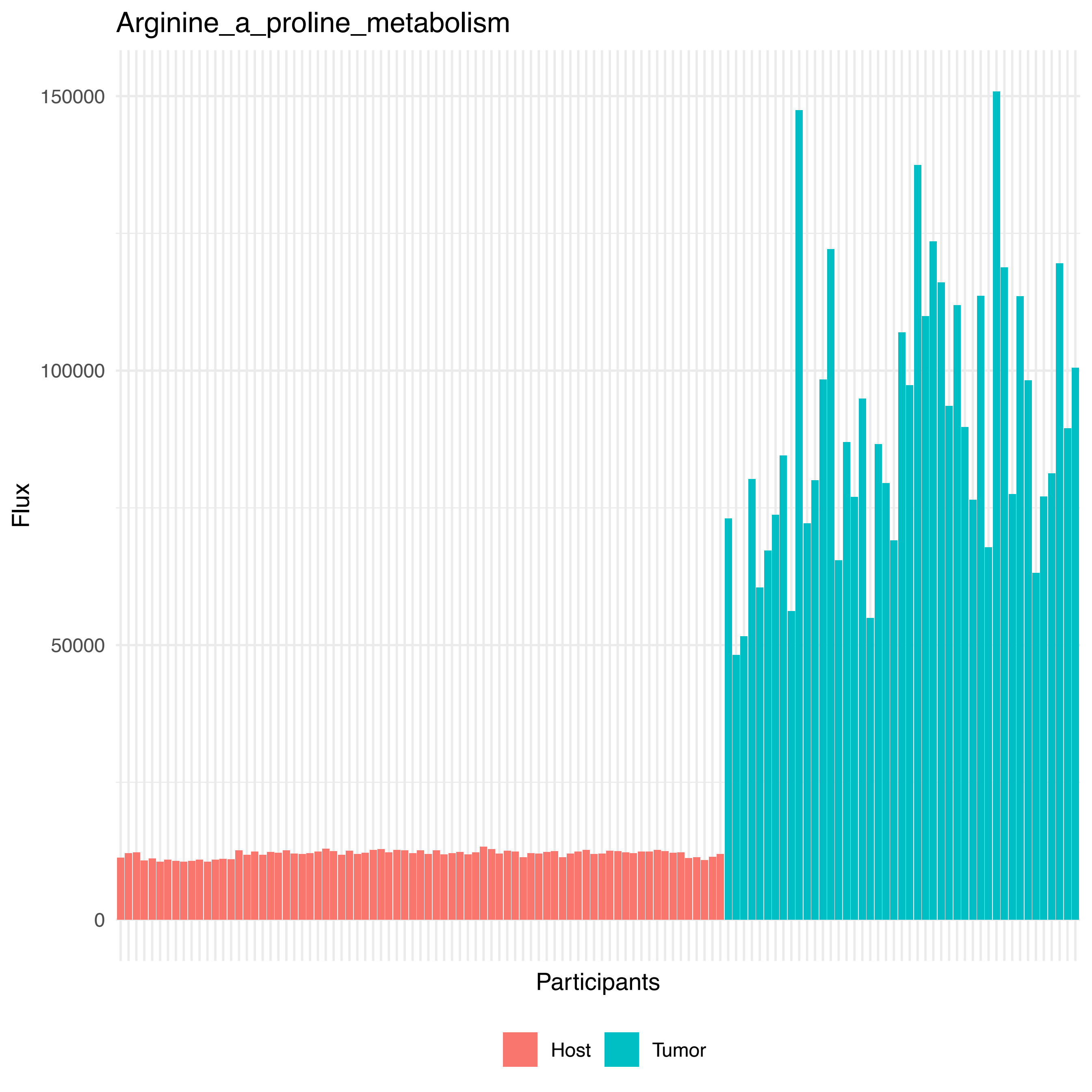

Arginine_a_proline_metabolism
Flux
150000
100000
50000
0
Participants
Host
Tumor

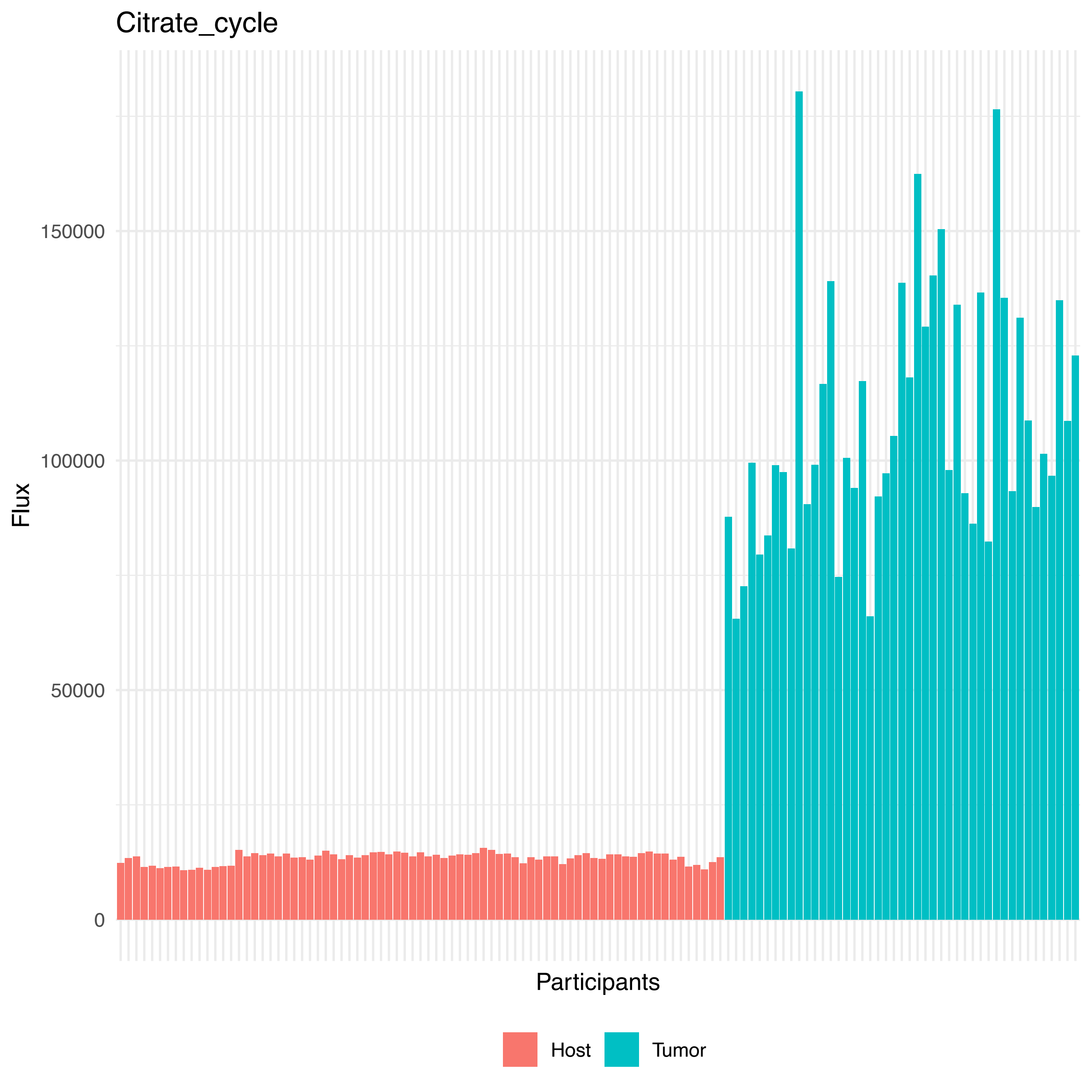
Citrate_cycle
Flux
150000
100000
50000
0
Participants
Host
Tumor

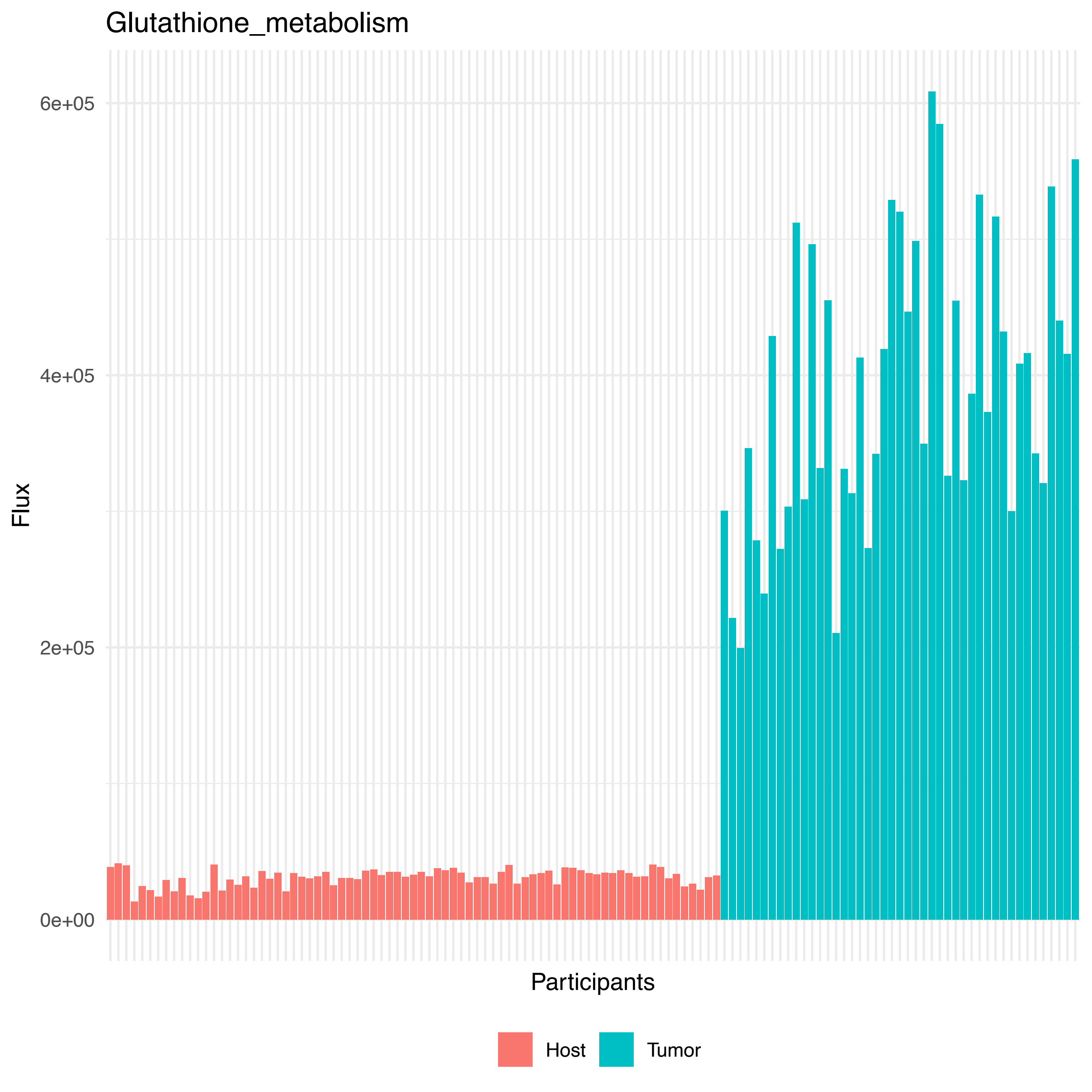
Glutathione_metabolism
Flux
6e+05
4e+05
2e+05
0e+00
Participants
Host
Tumor

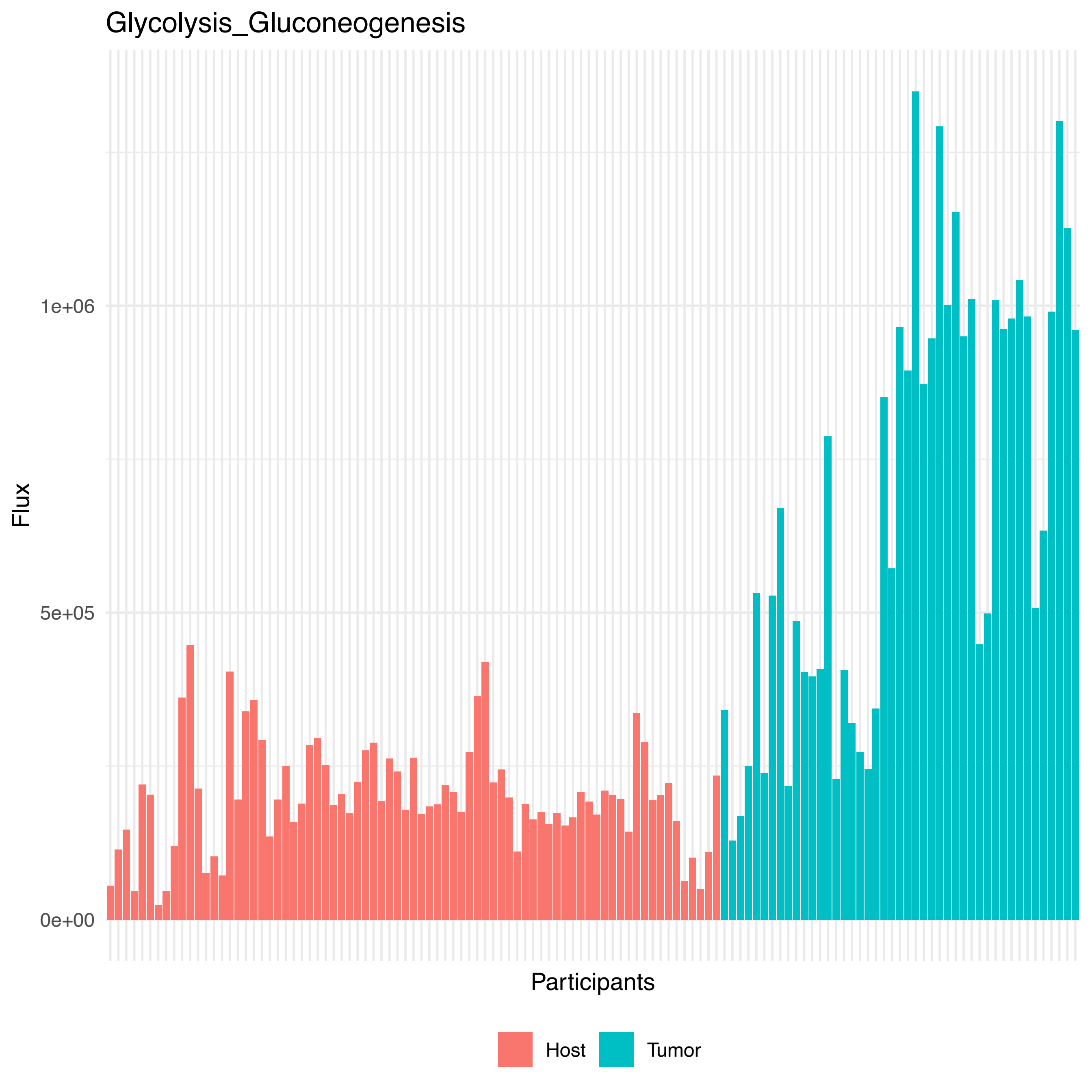

Glycolysis_Gluconeogenesis
Flux
1e+06
5e+05
0e+00
Participants
Host
Tumor

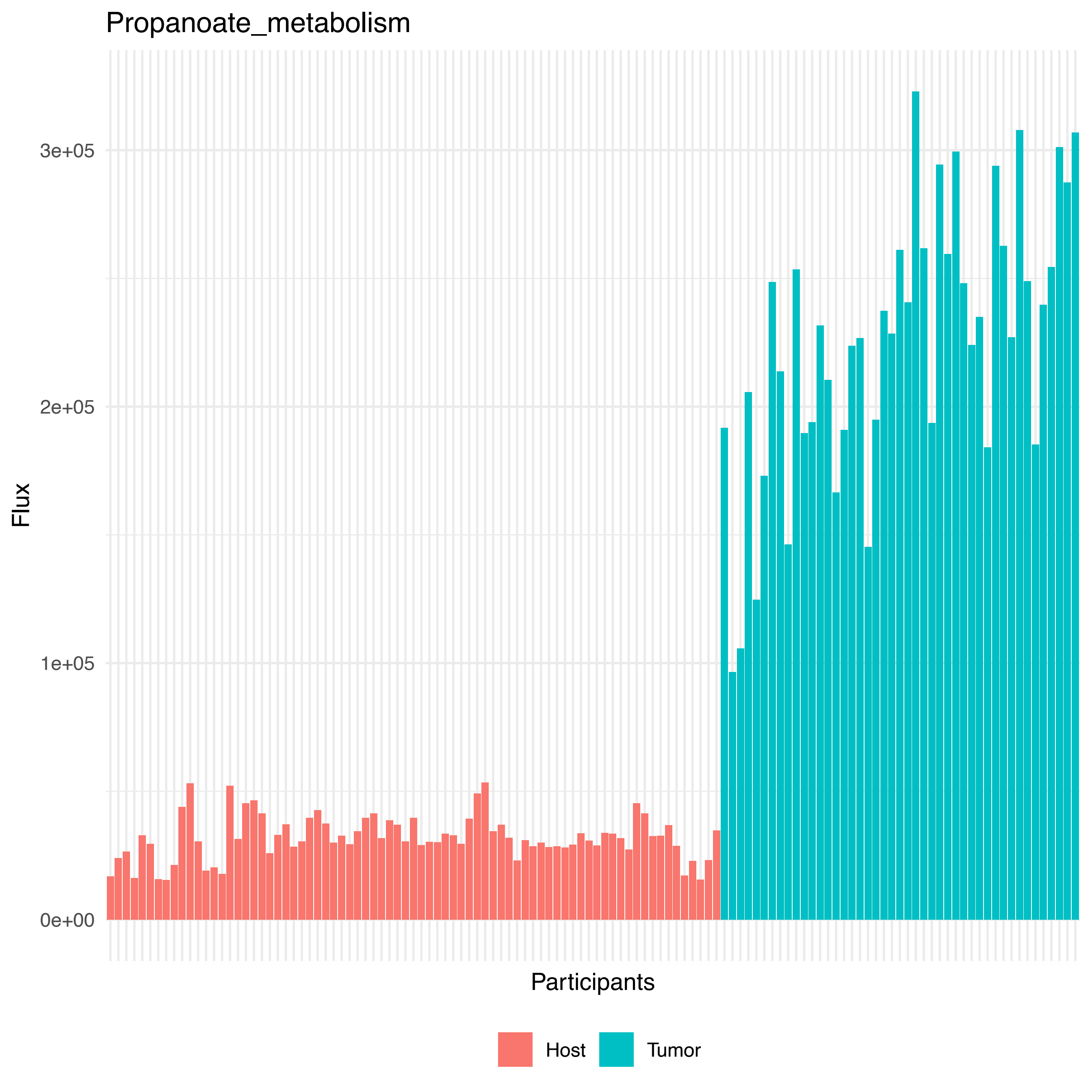

Propanoate_metabolism
3e+05
2e+05
1e+05
0e+00
Flux
Participants
Host
Tumor

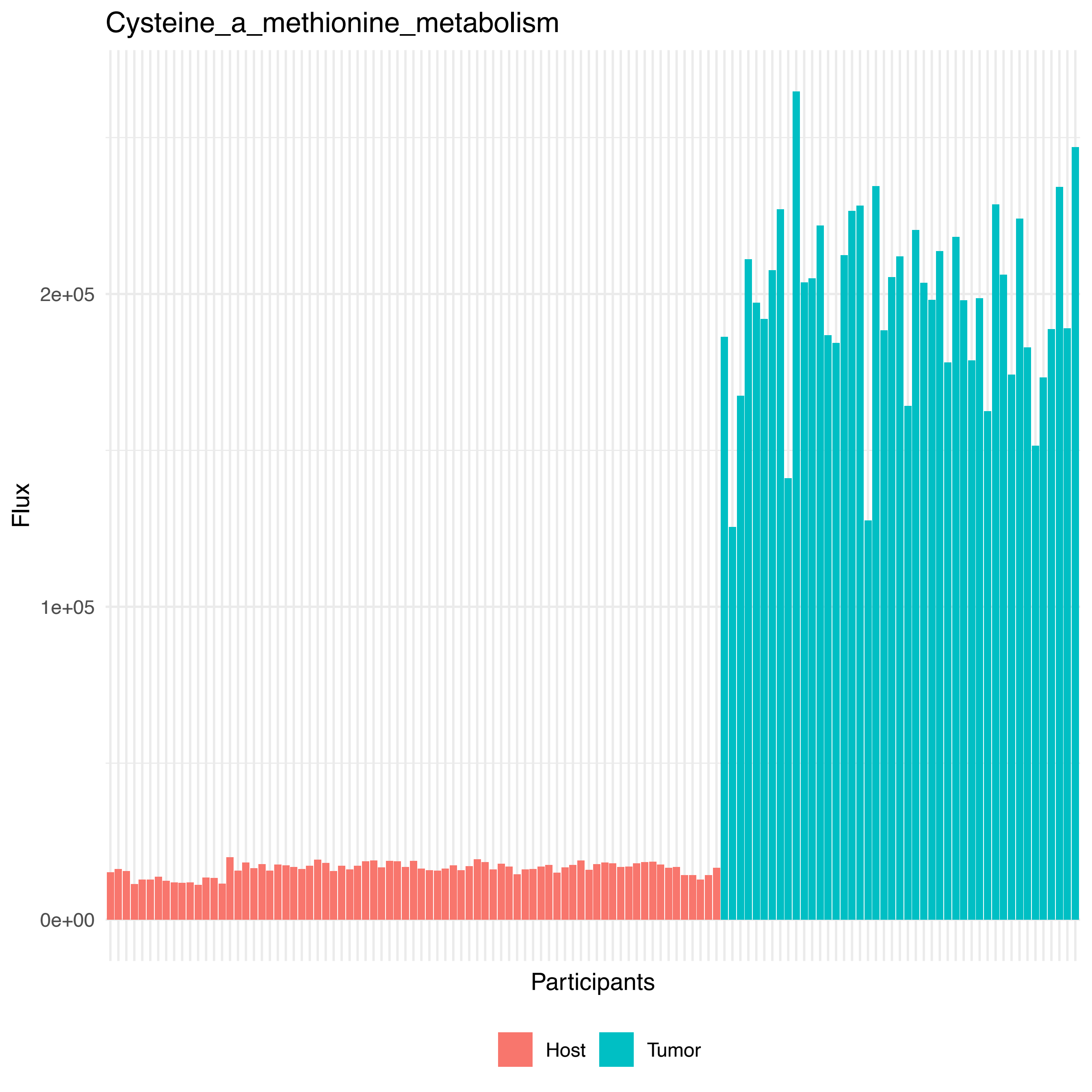

Cysteine_a_methionine_metabolism
2e+05
1e+05
0e+00
Flux
Participants
Host
Tumor

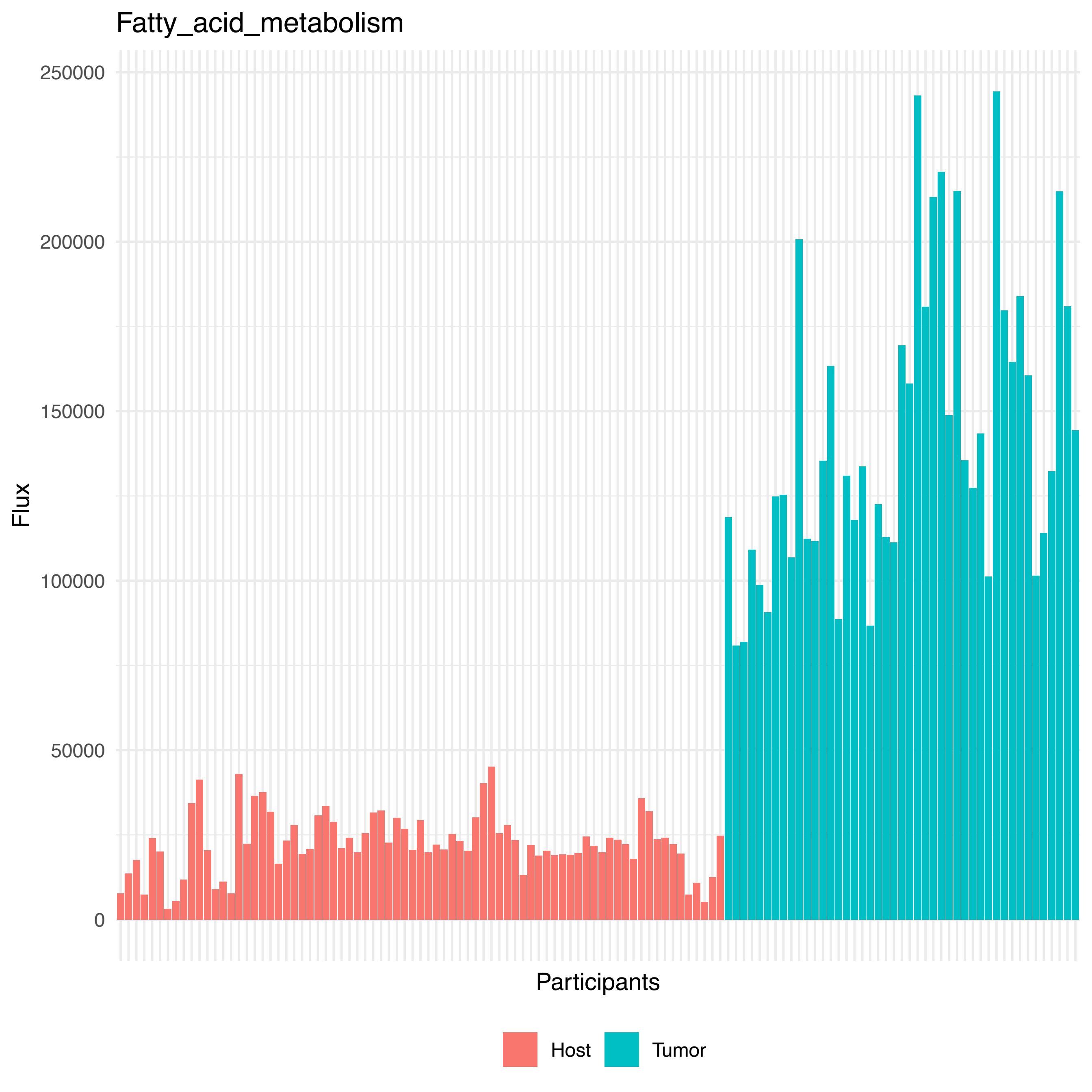

Fatty_acid_metabolism
250000
200000
150000
100000
50000
0
Flux
Participants
Host
Tumor

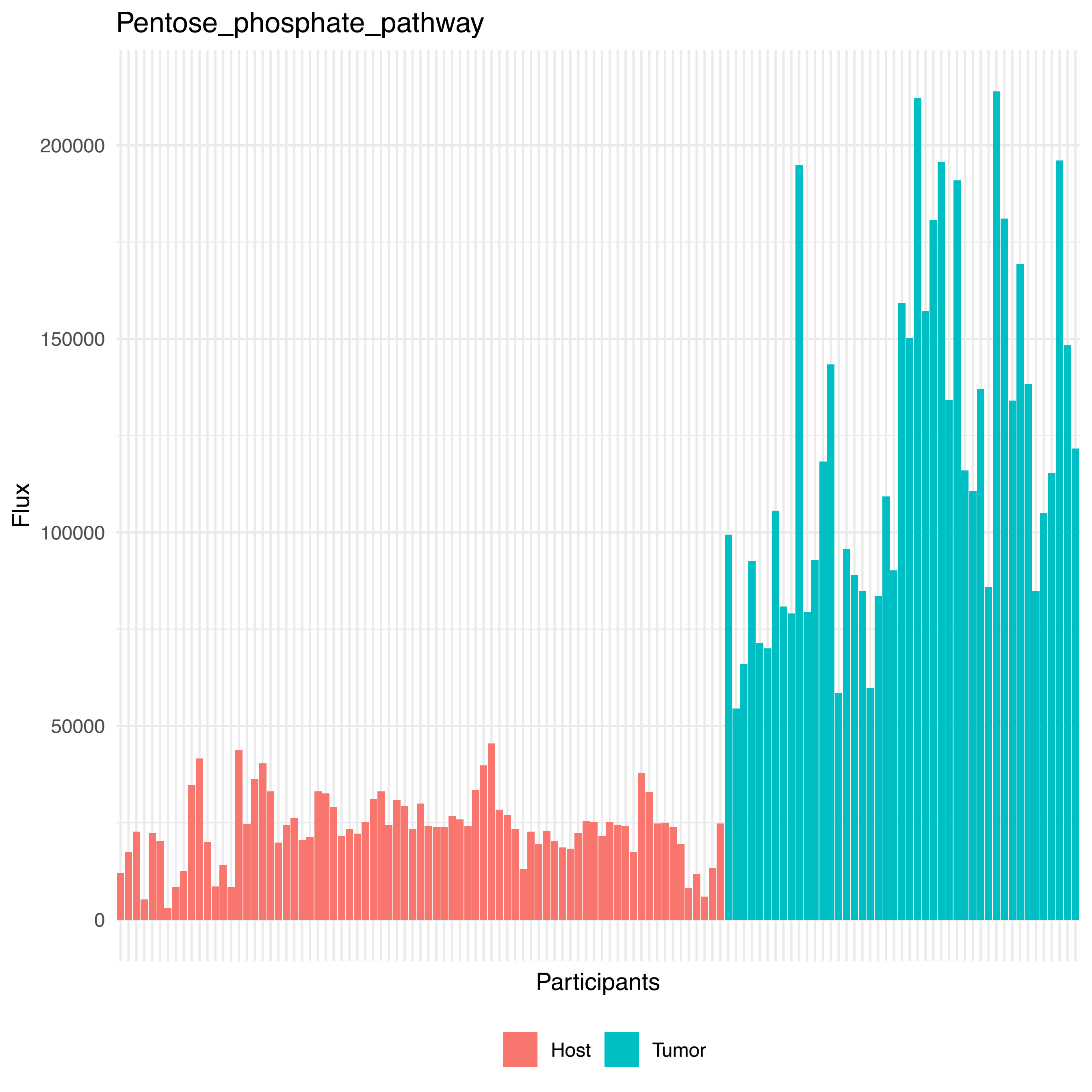

Pentose_phosphate_pathway
Flux
200000
150000
100000
50000
0
Participants
Host
Tumor

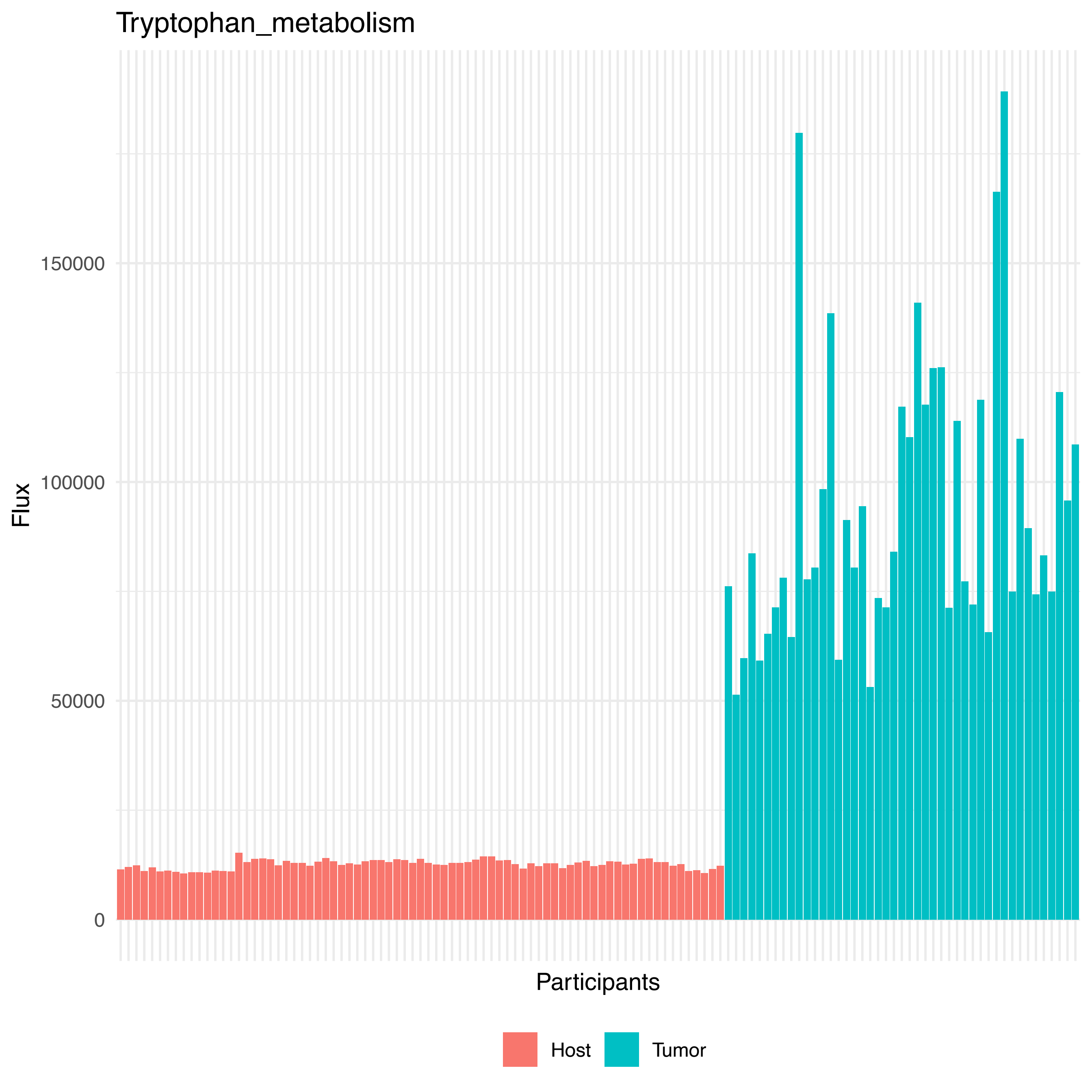

Tryptophan_metabolism
Flux
150000
100000
50000
0
Participants
Host
Tumor

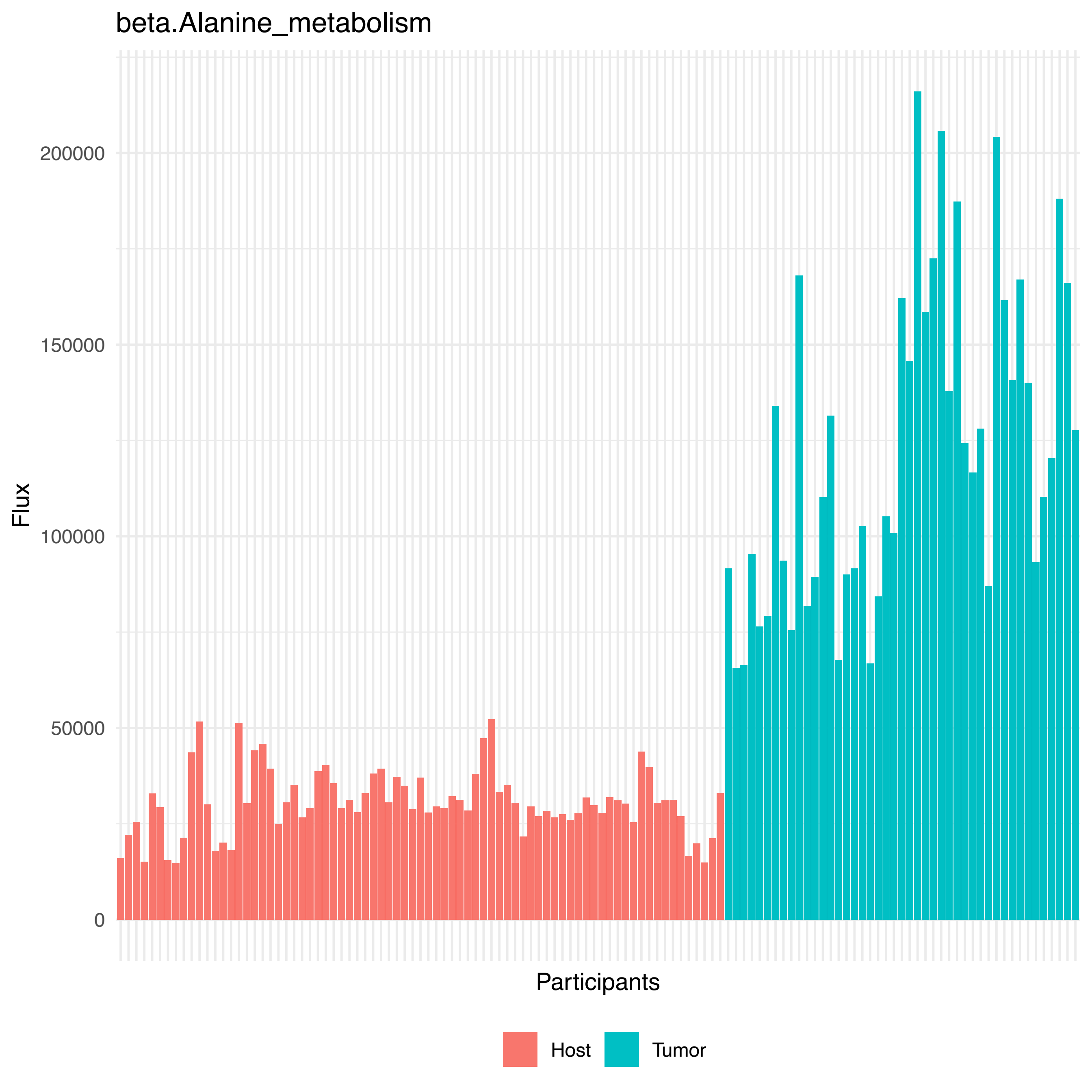

beta.Alanine_metabolism
Flux
200000
150000
100000
50000
0
Participants
Host
Tumor

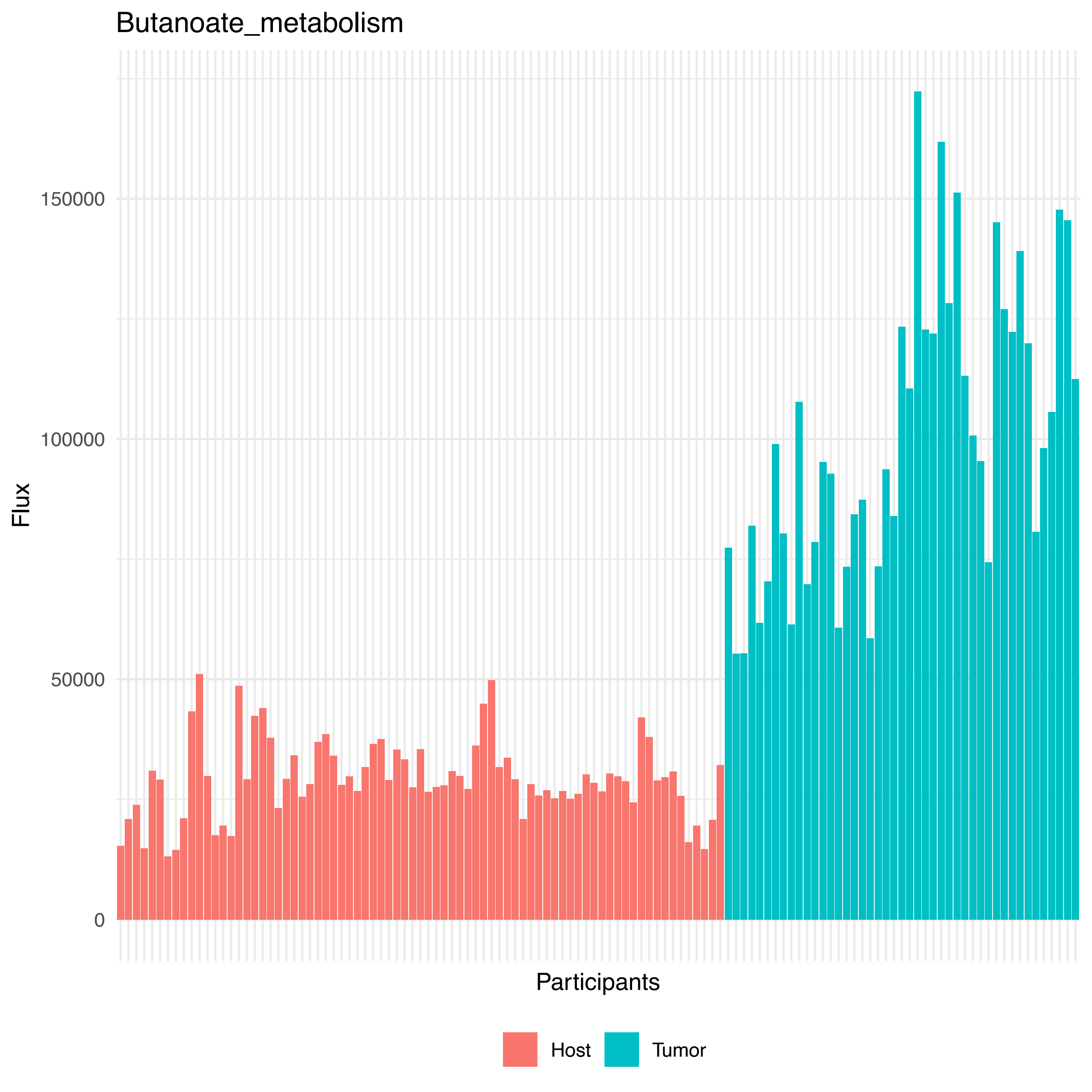
Butanoate_metabolism
Flux
150000
100000
50000
0
Participants
Host
Tumor

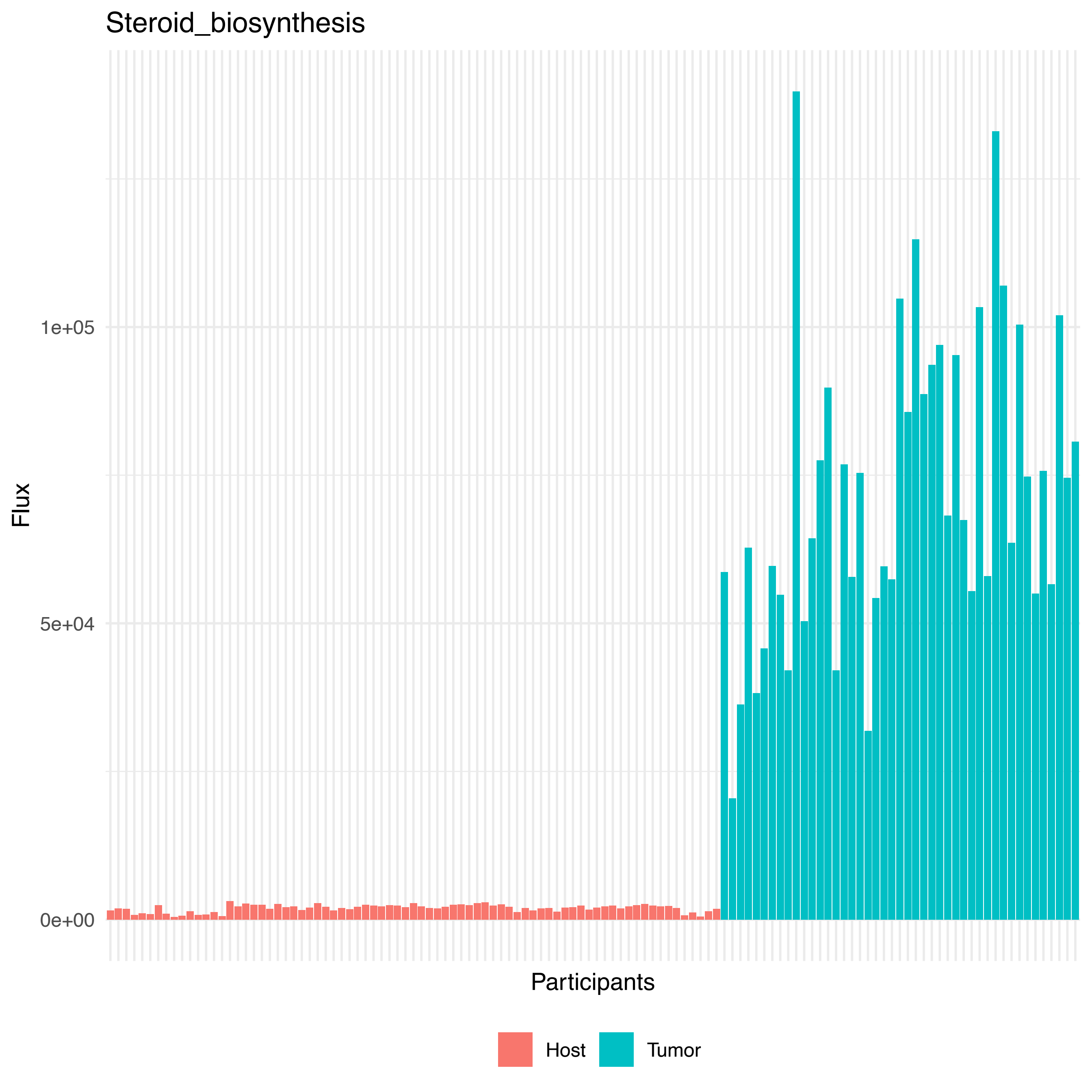
Steroid_biosynthesis
Flux
1e+05
5e+04
0e+00
Participants
Host
Tumor

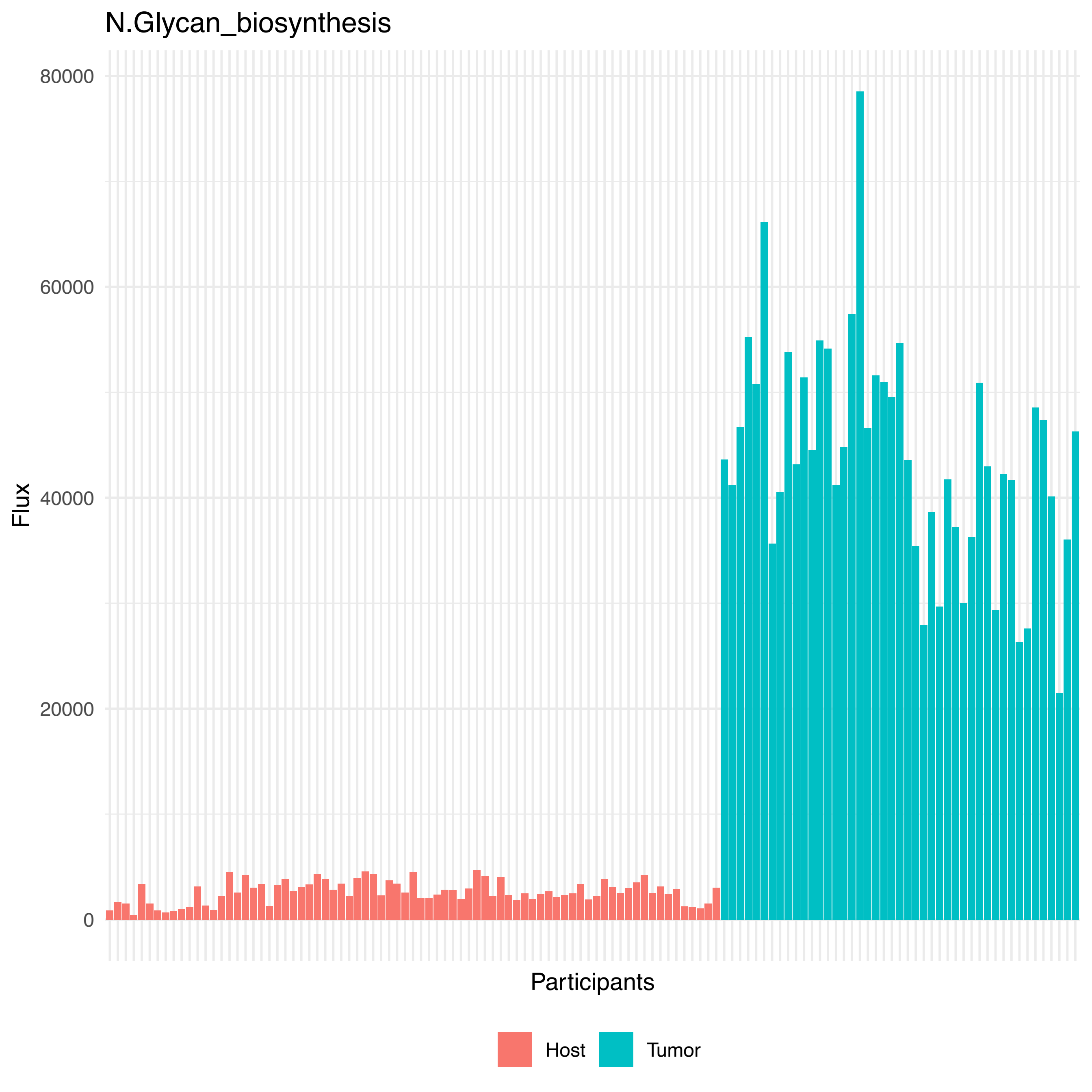

N.Glycan_biosynthesis
Flux
80000
60000
40000
20000
0
Participants
Host
Tumor

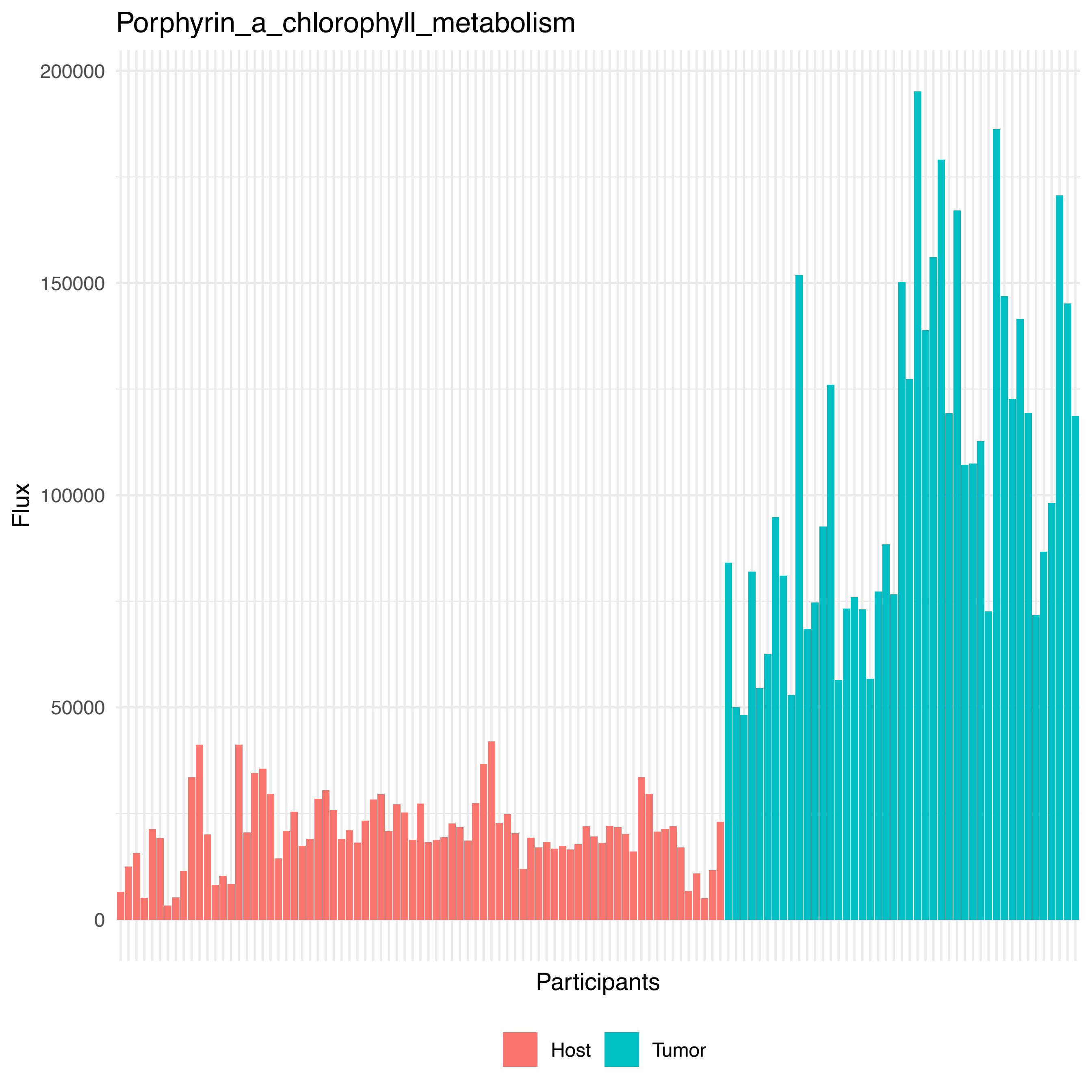
Porphyrin_a_chlorophyll_metabolism
200000
150000
100000
50000
0
Flux
Participants
Host
Tumor

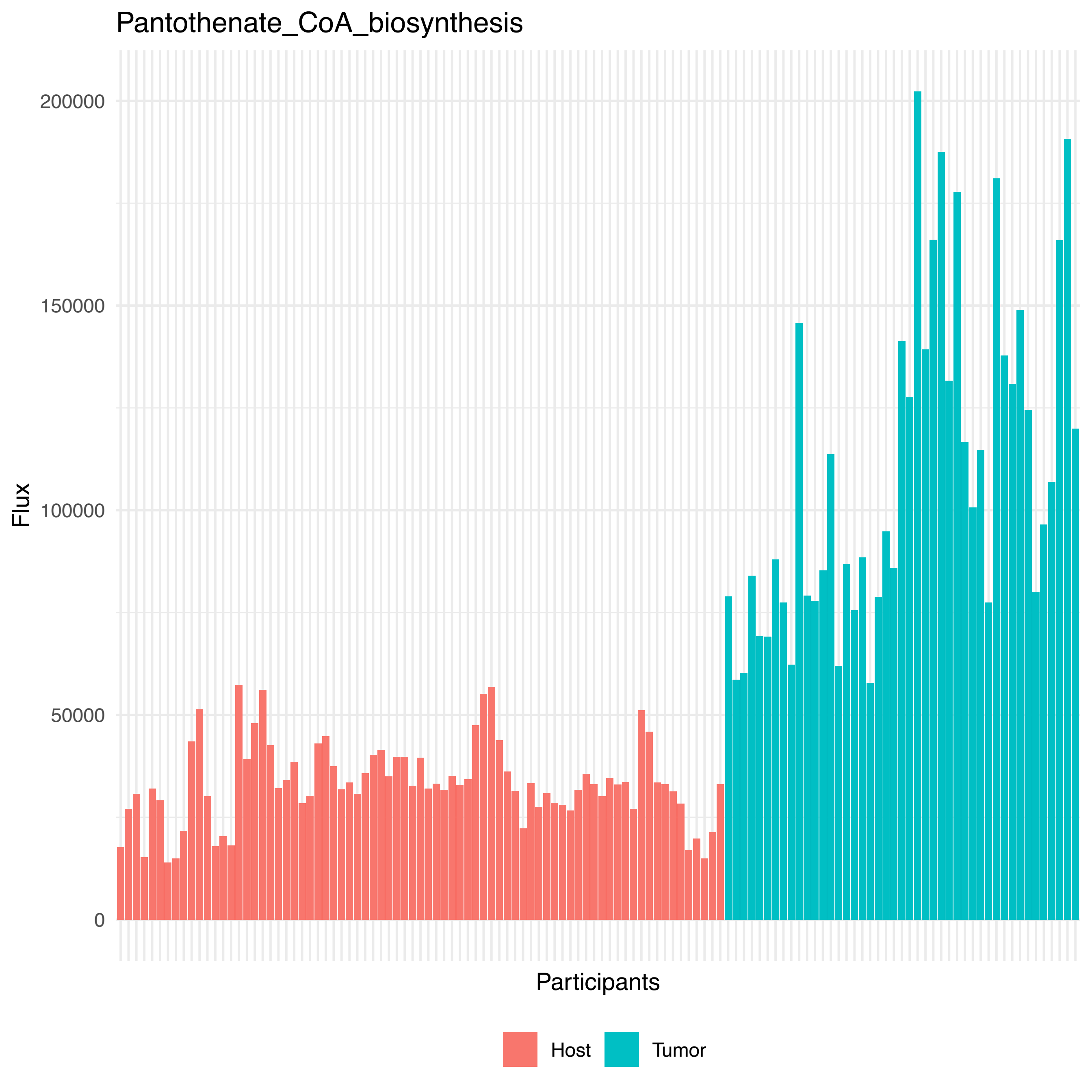

Pantothenate_CoA_biosynthesis
Flux
200000
150000
100000
50000
0
Participants
Host
Tumor

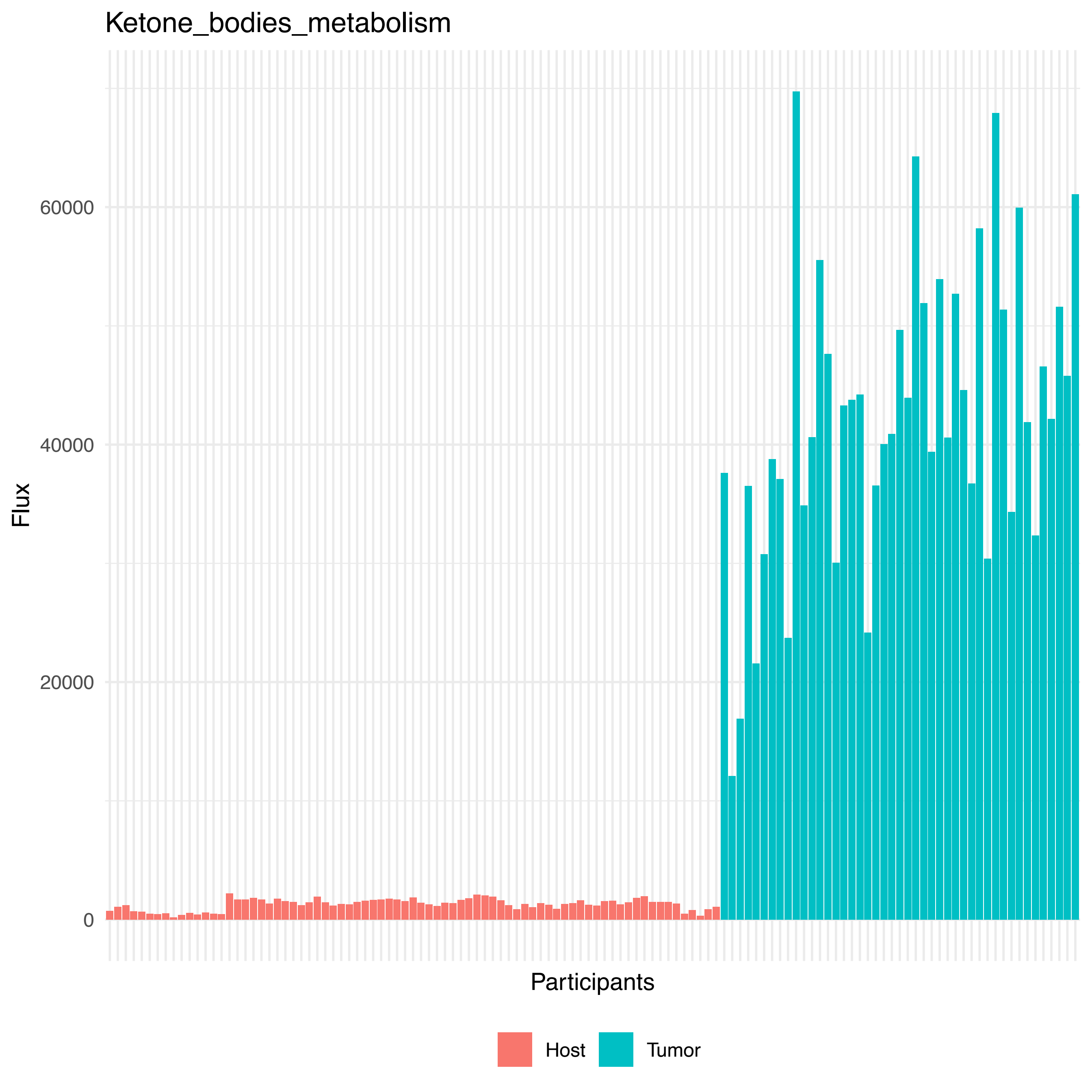

Ketone_bodies_metabolism
Flux
60000
40000
20000
0
Participants
Host
Tumor

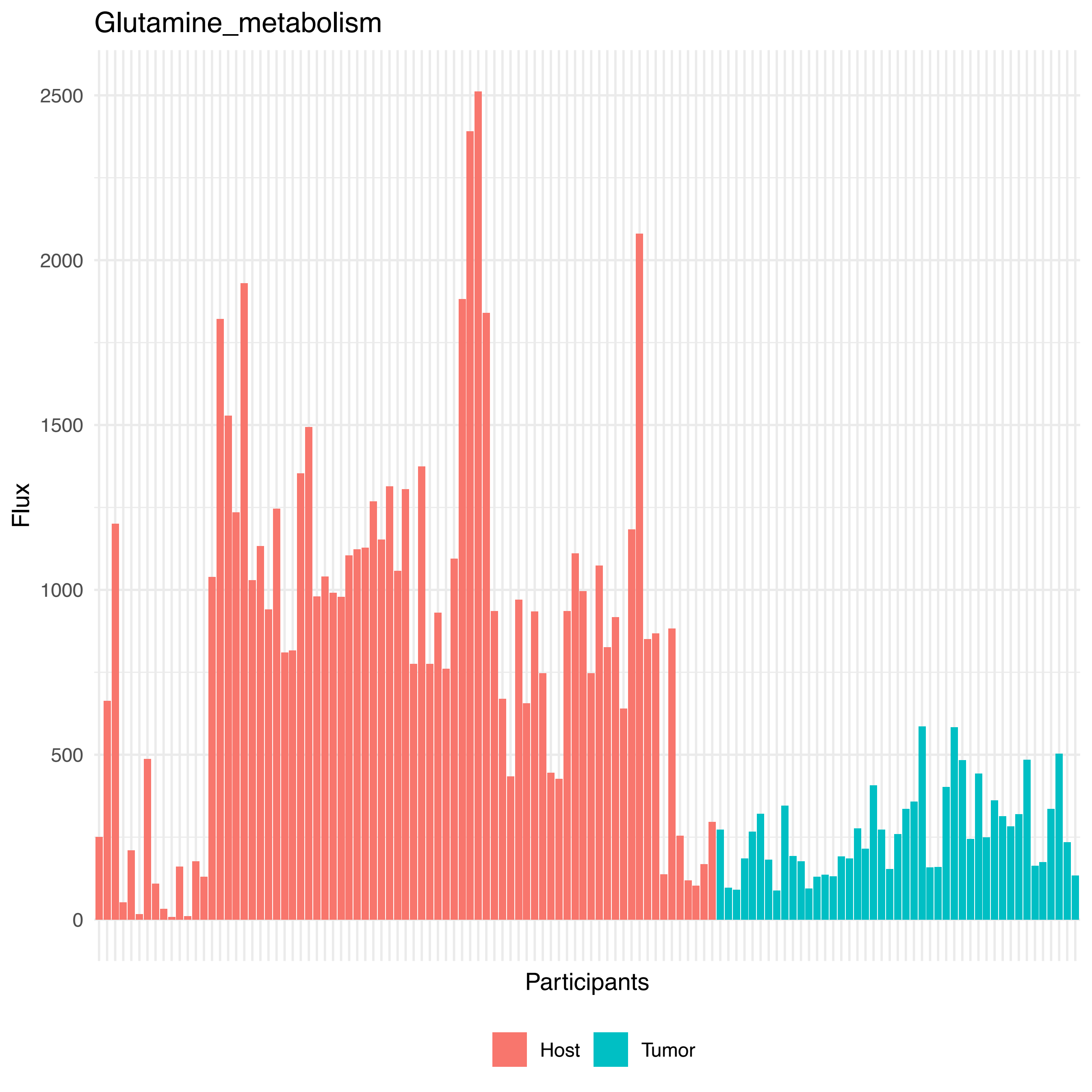

Glutamine_metabolism
2500
2000
1500
1000
500
0
Flux
Participants
Host
Tumor

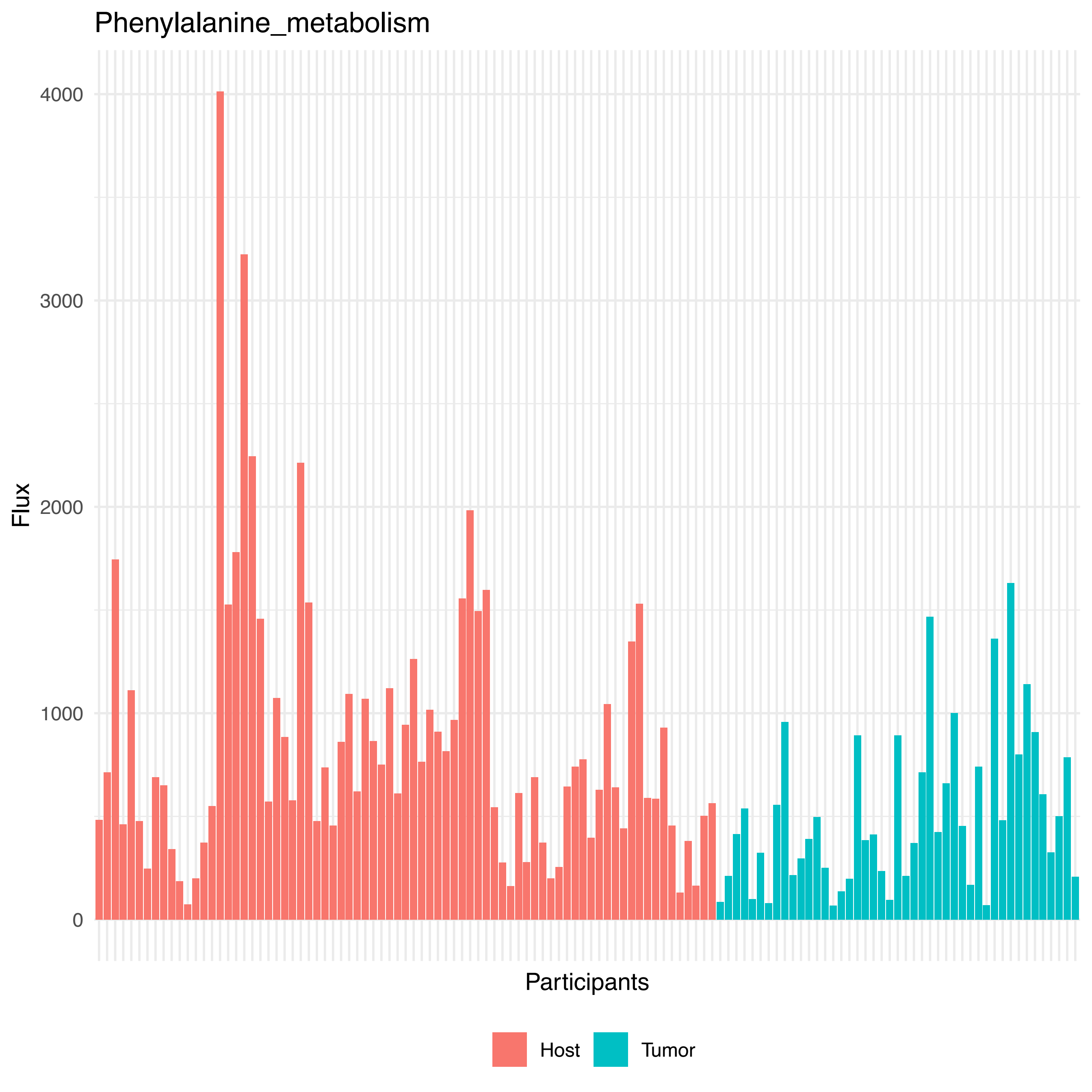

Phenylalanine_metabolism
Flux
4000
3000
2000
1000
0
Participants
Host
Tumor

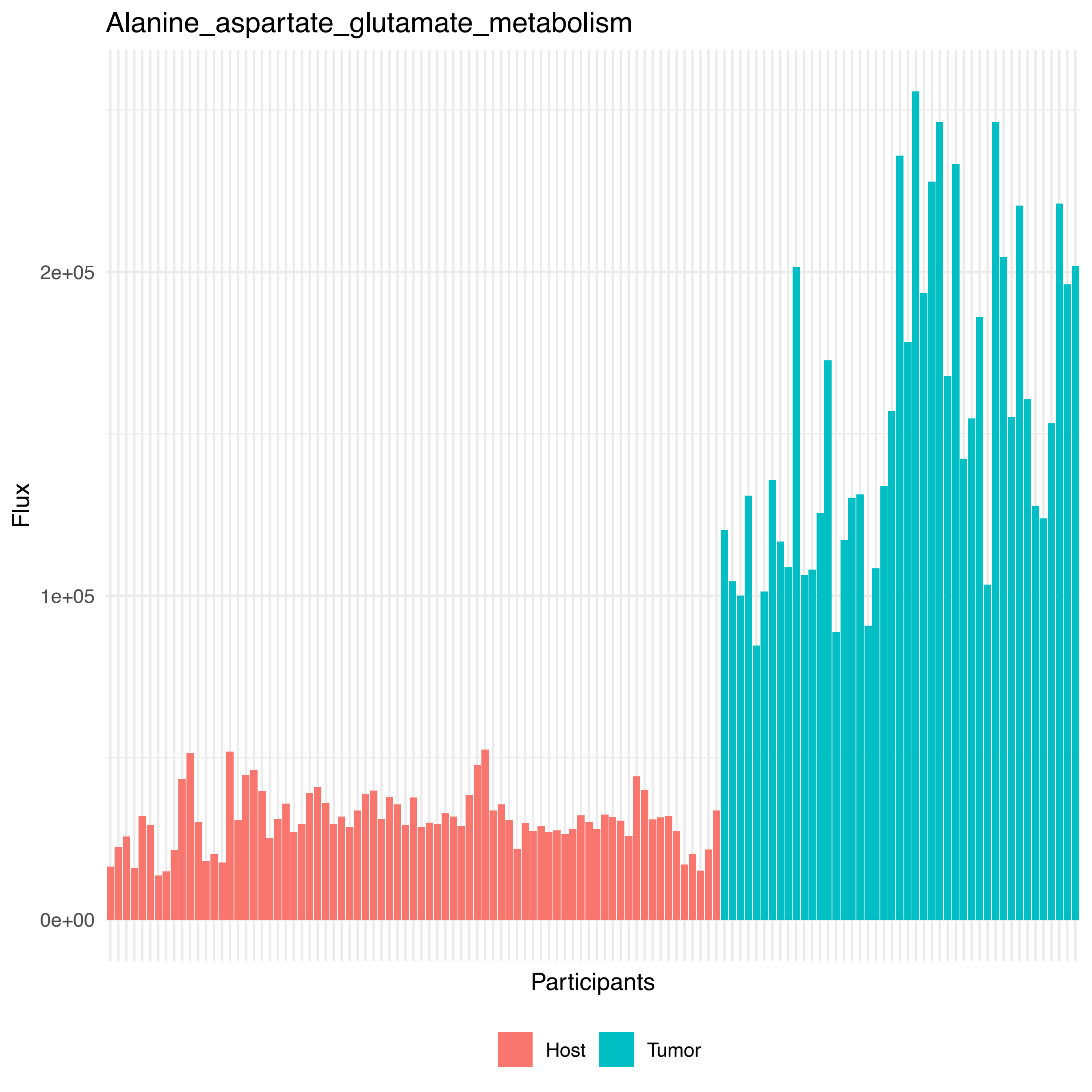
Alanine_aspartate_glutamate_metabolism
Flux
2e+05
1e+05
0e+00
Participants
Host
Tumor

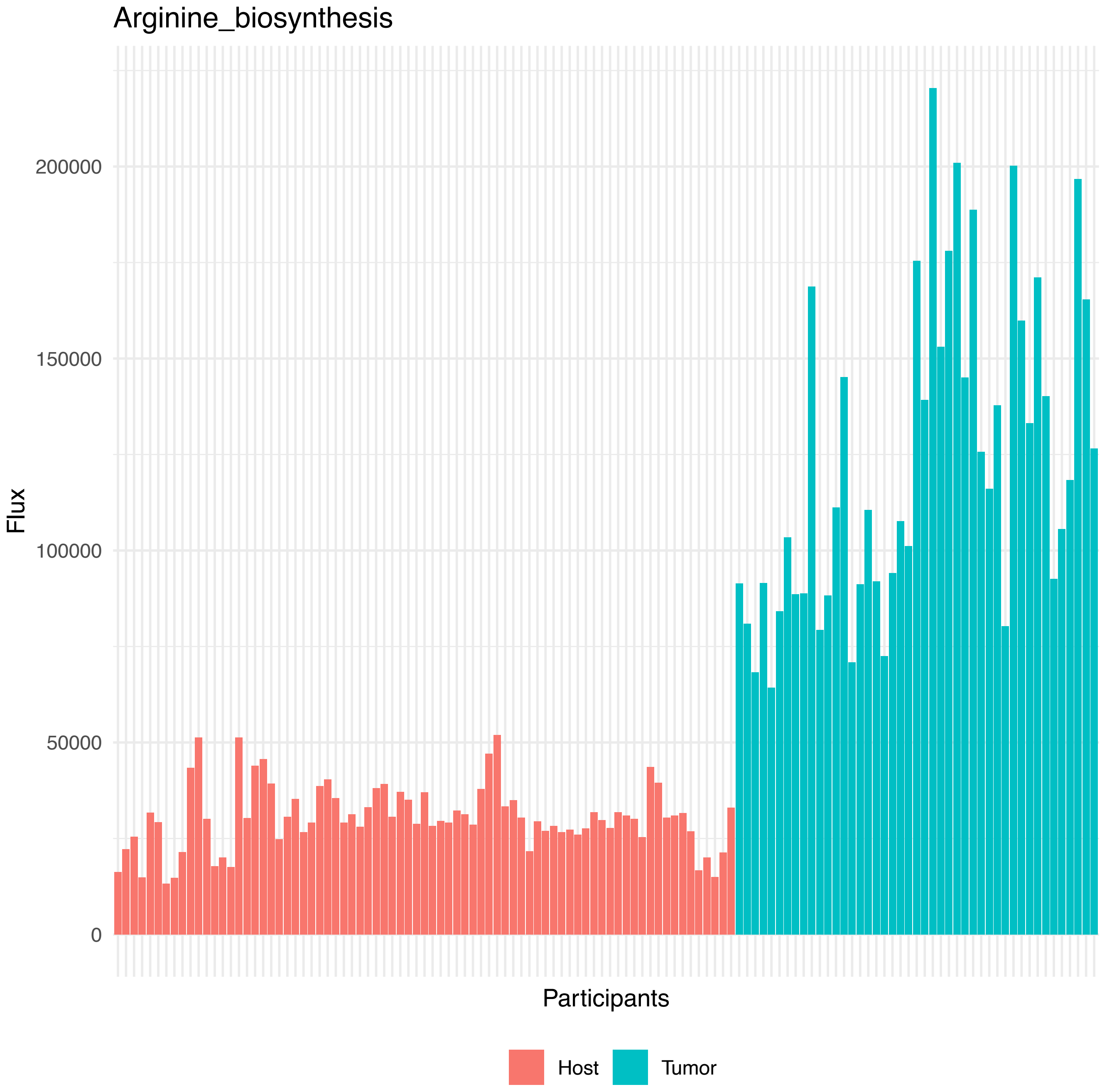

Arginine_biosynthesis
Flux
200000
150000
100000
50000
0
Participants
Host
Tumor

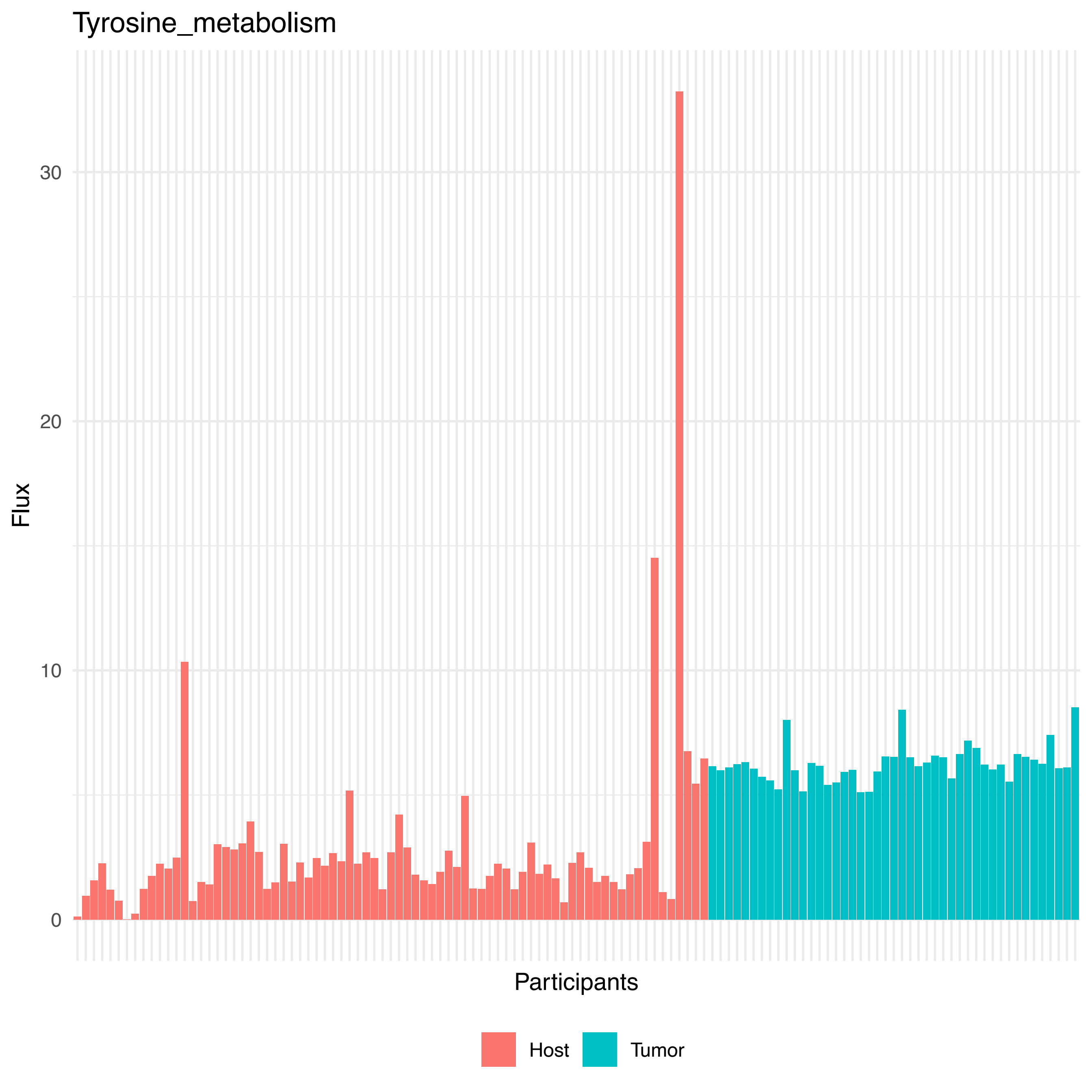
Tyrosine_metabolism
30
20
10
0
Flux
Participants
Host
Tumor

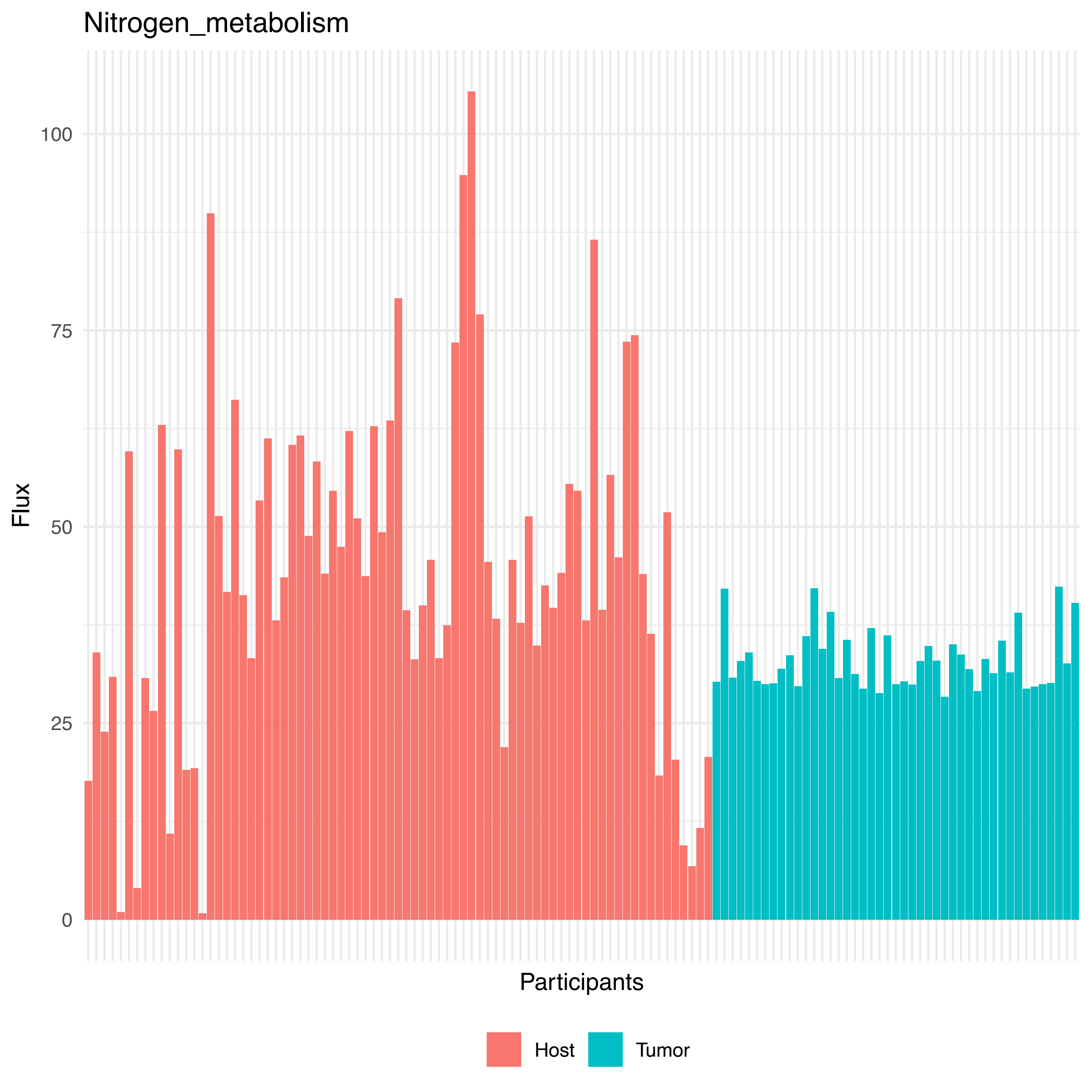
Nitrogen_metabolism
Flux
100
75
50
25
0
Participants
Host
Tumor
