# Supplementary material for: Differential early diagnosis of benign versus malignant lung cancer using systematic pathway flux analysis of peripheral blood leukocytes
Source: Sci Rep. 2022 Mar 24;12:5070. doi: 10.1038/s41598-022-08890-x (PMC8948197; doi:10.1038/s41598-022-08890-x)
Supplement: Supplementary file 2 — Supplementary Information 2. [file 41598_2022_8890_MOESM2_ESM.pdf]

Supplement Legends

Supplement Figure 1: **A**: the internal database diagram of the artificial intelligent (AI) model; **B**: the miRNA modeling pattern in the AI model. **C**: two array digital structure of the AI model;

Supplement Figure 2: IM-Index related pathway flux plot in the three participant groups.

Supplement Figure 3: Pathway flux comparison between adenocarcinoma and squamous carcinoma sub-groups.

Supplement Figure 4: Pathway flux comparison between host- and tumor-side.

Supplement Table 1. The clinical information and IM-Index of 109 participants

Supplement Table 2. The statistics overview of the model-components

Supplement Information 1. Function of the immune system consisting of signaling transduction and energy metabolism

Supplement Information / Figures / Tables

Differential Early Diagnosis of Benign vs Malignant Lung Cancer using Systematic Pathway Flux Analysis of Peripheral Blood Leukocytes

Jian Li[1,+], Xiaoyu Li[2,+], Ming Li[3,+], Hong Qiu[2], Christian Saad[4], Bo Zhao[5], Fan Li[5], Xiaowei Wu[5], Dong Kuang[6,7], Fengjuan Tang[6,7], Yaobing Chen[6,7], Hongge Shu[8], Jing Zhang[8], Qiuxia Wang[8], He Huang[9], Shankang Qi[9], Changkun Ye[10], Amy Bryant[11], Xianglin Yuan[2], Christian Kurts[1], Guangyuan Hu[2,*] , Weiting Cheng[12,*], Qi Mei[2,*]

[1] Institute of Molecular Medicine and Experimental Immunology, University Clinic of Rheinische Friedrich-Wilhelms-University, Bonn, Germany
[2] Department of Oncology, Tongji Hospital, Tongji Medical College, Huazhong University of Science and Technology, Wuhan, Hubei, People's Republic of China
[3] Department of Oncology, Wuhan Pulmonary Hospital, Wuhan, Hubei, People's Republic of China
[4] Department of Computer Science, University of Augsburg, Augsburg, Germany
[5] Department of thoracic surgery, Tongji Hospital, Tongji Medical College, Huazhong University of Science and Technology, Wuhan, Hubei, People's Republic of China
[6] Institute of Pathology, Tongji Hospital, Tongji Medical College, Huazhong University of Science and Technology, Wuhan, Hubei, People's Republic of China
[7] Department of Pathology, School of Basic Medicine, Tongji Medical College, Huazhong University of Science and Technology, Wuhan, Hubei, People's Republic of China
[8] Radiology department, Tongji Hospital, Tongji Medical College, Huazhong University of Science and Technology, Wuhan, Hubei, People's Republic of China
[9] Shanghai Institute of Materia Medica, Chinese Academy of Sciences, Shanghai, People's Republic of China
[10] Medical Research Center of Yu Huang Hospital, Yu Huang, Zhejiang, People's Republic of China
[11] Department of Biochemical and Pharmaceutical Sciences, College of Pharmacy, Idaho State University
[12] Department of Oncology, Wuhan No. 1 Hospital, Wuhan, Hubei, People's Republic of China
[*] Corresponding Authors: h.g.y.121@163.com, joycvt@126.com, borismq@163.com
[+] Contributing equally authors

Function of the immune system consists of two parts: signaling transduction and energy metabolism

At the beginning of an immune response, naive T-cells recognize antigen with proper co-stimulation (e.g CD45) and become activated by starting the signal from TCR receptor. This leads to signal propagation via Ca+ channel, PI3K-AKT, NF-kB, MAPK and other pathways, among others which subsequently promote related transcriptional programs. Some changed transcription patterns turn on the signal of JAK-STAT, mTOR and AMPK pathways to initiate metabolic energetic remodeling via glycolysis, fatty-acid and amino acid metabolism in order to support T-cell proliferation, growth and function. Other altered transcription patterns lead to the activation of pathways such as WNT, TLR, IFN, and NFAT, which result in the maturation and differentiation of immune cells including B-cells and NK-cells. The different types of immune cells collaborate and crosstalk based on proper pathway interactions to enable complex function of host immunity that is facilitated with systematical requirement of energy and nutrient. Thus, a subtle change in nutrient conditions would trigger signaling pathways such as the AMPK pathway, an important metabolic sensor in T-cells, to send signal and communicate with different pathways for a corresponding adjustment in the level of oxidative metabolism for the durable effector T-cell responses. Moreover, these activated signalings further activate downstream transcriptional regulators such as HIF-1, TP53, Myc, FOXO, AP-1 deeply involve in glycolysis, fatty-acid metabolism and glutaminolysis and thereby broadly affects metabolic gene expression and energy metabolism. Further, quantitative and temporal changes of ion flux related signaling events affect the development and function of immune cells significantly. All of these observations illustrate that the signaling process and energy metabolism are interconnected in regulating immune cells to ensure the proper functionality of entire the immune system. Recent studies have demonstrated that an increased glycolysis and glucose uptake is highly associated with augmented effector T-cell functions; diminished availability of nutrients such as glucose, fatty acids and other nutrients within the tumor microenvironment leads directly to a decrease anti-tumor immune response. More importantly, fluctuations in the concentrations of nutrients or intensity of signaling transduction can considerably alter the functions of immune cells. Therefore, it is plausible that the functionality and strength of the immune system could be reflect through the intensity of signaling transduction and the metabolic flux from energetic metabolism.

Innate immune

Toll-like receptor signaling pathway and metabolism[1-16]
Complement and coagulation cascades[17-26]
NOD-like receptor signaling pathway[27-51]
Cytosolic DNA-sensing and transcription pathway[52-71]
NK cell mediated cytotoxicity and metabolism[72-87]
C-type lectin receptor signaling pathway[88-99]
Fc gamma R-mediated phagocytosis and cell cycle[100-109]
Fc epsilon RI and PI3K signaling[110-123]
Neutrophil degranulation [124,125]

Cytokine interaction and metabolism[126-146]

Adaptive immune

T cell receptor signaling pathway and metabolism [147-209]
B cell receptor signaling pathway and metabolism [210-233]
Antigen processing and related signaling and metabolism [234-280]
Cell adhesion molecules (CAMs): [281-300]
Adherens junction [301-309]
Leukocyte migration and metabolism [203,310-313]
MAPK related signaling pathway [314-331]

The definition of **immune-related index** (IM-Index):

$$\text{IM-Index} = \alpha * \sum_{p \in \text{signaling transduction}} flux(P) + \beta * \sum_{p \in \text{energy metabolism}} \text{flux}(P)$$

where the default values of α, β are set to 1 in this study;

Signaling transduction includes: ABL.signaling, Cell Cycle, COX.signaling, Estrogen.signaling, InsulinR.signaling, WNT.signaling, BMP.signaling, RAR.signaling, TEK.signaling, VEGF.signaling, DYRK.signaling, ERK5.signaling, IGF1R.signaling, Ephrin.signaling, Growth.Hormone, Hedgehog.signaling, NOTCH.signaling, Interleukin.signaling, TCR.signaling, IFN.signaling, NFAT.signaling, BCR.signaling;

Energy metabolism includes: Alanine aspartate and glutamate metabolism, Arginine biosynthesis, Cysteine and methionine metabolism, Pyruvate metabolism, Nitrogen metabolism, beta.Alanine metabolism, Butanoate metabolism, Histidine metabolism, Lysine degradation, Citrate cycle .Tcycle., Tyrosine metabolism, Propanoate metabolism, Fatty acid degradation, Tryptophan metabolism, Ether lipid metabolism, Retinol metabolism, Fatty acid metabolism, Pantothenate and CoA biosynthesis, Valine leucine and isoleucine degradation, Fructose and mannose metabolism, Valine leucine and isoleucine biosynthesis.

References:

1 Schaefer, L. Complexity of danger: the diverse nature of damage-associated molecular patterns. *J Biol Chem* **289**, 35237-35245, doi:10.1074/jbc.R114.619304 (2014).

2 Gibbard, R. J., Morley, P. J. & Gay, N. J. Conserved features in the extracellular domain of human toll-like receptor 8 are essential for pH-dependent signaling. *J Biol Chem* **281**, 27503-27511, doi:10.1074/jbc.M605003200 (2006).

3 Ewald, S. E. *et al.* The ectodomain of Toll-like receptor 9 is cleaved to generate a functional receptor. *Nature* **456**, 658-662, doi:10.1038/nature07405 (2008).

4 O'Neill, L. A. & Bowie, A. G. The family of five: TIR-domain-containing adaptors in Toll-like receptor signalling. *Nat Rev Immunol* **7**, 353-364, doi:10.1038/nri2079 (2007).

5 Barton, G. M., Kagan, J. C. & Medzhitov, R. Intracellular localization of Toll-like receptor 9 prevents recognition of self DNA but facilitates access to viral DNA. *Nat Immunol* **7**, 49-56, doi:10.1038/ni1280 (2006).

6 Akira, S., Uematsu, S. & Takeuchi, O. Pathogen recognition and innate immunity. *Cell* **124**, 783-801, doi:10.1016/j.cell.2006.02.015 (2006).

7 Kawai, T. & Akira, S. Pathogen recognition with Toll-like receptors. *Curr Opin Immunol* **17**, 338-344, doi:10.1016/j.coi.2005.02.007 (2005).

8 de Bouteiller, O. *et al.* Recognition of double-stranded RNA by human toll-like receptor 3 and downstream receptor signaling requires multimerization and an acidic pH. *J Biol Chem* **280**, 38133-38145, doi:10.1074/jbc.M507163200 (2005).

9 Liu, L. *et al.* Structural basis of toll-like receptor 3 signaling with double-stranded RNA. *Science* **320**, 379-381, doi:10.1126/science.1155406 (2008).

10 Aderem, A. & Smith, K. D. A systems approach to dissecting immunity and inflammation. *Semin Immunol* **16**, 55-67 (2004).

11 Kawai, T. & Akira, S. TLR signaling. *Semin Immunol* **19**, 24-32, doi:10.1016/j.smim.2006.12.004 (2007).

12 Takeda, K. & Akira, S. TLR signaling pathways. *Semin Immunol* **16**, 3-9 (2004).

13 Arbibe, L. *et al.* Toll-like receptor 2-mediated NF-kappa B activation requires a Rac1-dependent pathway. *Nat Immunol* **1**, 533-540, doi:10.1038/82797 (2000).

14 Means, T. K., Hayashi, F., Smith, K. D., Aderem, A. & Luster, A. D. The Toll-like receptor 5 stimulus bacterial flagellin induces maturation and chemokine production in human dendritic cells. *J Immunol* **170**, 5165-5175, doi:10.4049/jimmunol.170.10.5165 (2003).

15 Uematsu, S. & Akira, S. Toll-like receptors and innate immunity. *J Mol Med (Berl)* **84**, 712-725, doi:10.1007/s00109-006-0084-y (2006).

16 Uematsu, S. & Akira, S. Toll-like receptors and Type I interferons. *J Biol Chem* **282**, 15319-15323, doi:10.1074/jbc.R700009200 (2007).

17 Bajic, G., Degn, S. E., Thiel, S. & Andersen, G. R. Complement activation, regulation, and molecular basis for complement-related diseases. *Embo j* **34**, 2735-2757, doi:10.15252/embj.201591881 (2015).

18 Ricklin, D. & Lambris, J. D. Complement in immune and inflammatory disorders: therapeutic interventions. *J Immunol* **190**, 3839-3847,

doi:10.4049/jimmunol.1203200 (2013).

19 Zipfel, P. F. & Skerka, C. Complement regulators and inhibitory proteins. *Nat Rev Immunol* **9**, 729-740, doi:10.1038/nri2620 (2009).

20 Merle, N. S., Church, S. E., Fremeaux-Bacchi, V. & Roumenina, L. T. Complement System Part I - Molecular Mechanisms of Activation and Regulation. *Front Immunol* **6**, 262, doi:10.3389/fimmu.2015.00262 (2015).

21 Schmidt, B. Z. & Colten, H. R. Complement: a critical test of its biological importance. *Immunol Rev* **178**, 166-176, doi:10.1034/j.1600-065x.2000.17801.x (2000).

22 Ricklin, D., Hajishengallis, G., Yang, K. & Lambris, J. D. Complement: a key system for immune surveillance and homeostasis. *Nat Immunol* **11**, 785-797, doi:10.1038/ni.1923 (2010).

23 Oikonomopoulou, K., Ricklin, D., Ward, P. A. & Lambris, J. D. Interactions between coagulation and complement--their role in inflammation. *Semin Immunopathol* **34**, 151-165, doi:10.1007/s00281-011-0280-x (2012).

24 Hillmeister, P. & Persson, P. B. The Kallikrein-Kinin system. *Acta Physiol (Oxf)* **206**, 215-219, doi:10.1111/apha.12007 (2012).

25 Fujita, T., Matsushita, M. & Endo, Y. The lectin-complement pathway--its role in innate immunity and evolution. *Immunol Rev* **198**, 185-202, doi:10.1111/j.0105-2896.2004.0123.x (2004).

26 Smith, H. W. & Marshall, C. J. Regulation of cell signalling by uPAR. *Nat Rev Mol Cell Biol* **11**, 23-36, doi:10.1038/nrm2821 (2010).

27 Latz, E., Xiao, T. S. & Stutz, A. Activation and regulation of the inflammasomes. *Nat Rev Immunol* **13**, 397-411, doi:10.1038/nri3452 (2013).

28 Storek, K. M. & Monack, D. M. Bacterial recognition pathways that lead to inflammasome activation. *Immunol Rev* **265**, 112-129, doi:10.1111/imr.12289 (2015).

29 Murray, P. J. Beyond peptidoglycan for Nod2. *Nat Immunol* **10**, 1053-1054, doi:10.1038/ni1009-1053 (2009).

30 Man, S. M. & Kanneganti, T. D. Converging roles of caspases in inflammasome activation, cell death and innate immunity. *Nat Rev Immunol* **16**, 7-21, doi:10.1038/nri.2015.7 (2016).

31 Man, S. M. & Kanneganti, T. D. Gasdermin D: the long-awaited executioner of pyroptosis. *Cell Res* **25**, 1183-1184, doi:10.1038/cr.2015.124 (2015).

32 Estornes, Y. & Bertrand, M. J. IAPs, regulators of innate immunity and inflammation. *Semin Cell Dev Biol* **39**, 106-114, doi:10.1016/j.semcdb.2014.03.035 (2015).

33 Kerur, N. *et al.* IFI16 acts as a nuclear pathogen sensor to induce the inflammasome in response to Kaposi Sarcoma-associated herpesvirus infection. *Cell Host Microbe* **9**, 363-375, doi:10.1016/j.chom.2011.04.008 (2011).

34 Schroder, K. & Tschopp, J. The inflammasomes. *Cell* **140**, 821-832, doi:10.1016/j.cell.2010.01.040 (2010).

35 Lamkanfi, M. & Dixit, V. M. Inflammasomes: guardians of cytosolic sanctity. *Immunol Rev* **227**, 95-105, doi:10.1111/j.1600-065X.2008.00730.x (2009).

36 Guo, H., Callaway, J. B. & Ting, J. P. Inflammasomes: mechanism of action, role in disease, and therapeutics. *Nat Med* **21**, 677-687, doi:10.1038/nm.3893 (2015).

37 Broz, P. & Dixit, V. M. Inflammasomes: mechanism of assembly, regulation and signalling. *Nat Rev Immunol* **16**, 407-420, doi:10.1038/nri.2016.58 (2016).
38 Blander, J. M. A long-awaited merger of the pathways mediating host defence and programmed cell death. *Nat Rev Immunol* **14**, 601-618, doi:10.1038/nri3720 (2014).
39 Jo, E. K., Kim, J. K., Shin, D. M. & Sasakawa, C. Molecular mechanisms regulating NLRP3 inflammasome activation. *Cell Mol Immunol* **13**, 148-159, doi:10.1038/cmi.2015.95 (2016).
40 Broz, P. & Monack, D. M. Newly described pattern recognition receptors team up against intracellular pathogens. *Nat Rev Immunol* **13**, 551-565, doi:10.1038/nri3479 (2013).
41 Shaw, M. H., Reimer, T., Kim, Y. G. & Nunez, G. NOD-like receptors (NLRs): bona fide intracellular microbial sensors. *Curr Opin Immunol* **20**, 377-382, doi:10.1016/j.coi.2008.06.001 (2008).
42 Chen, G., Shaw, M. H., Kim, Y. G. & Nunez, G. NOD-like receptors: role in innate immunity and inflammatory disease. *Annu Rev Pathol* **4**, 365-398, doi:10.1146/annurev.pathol.4.110807.092239 (2009).
43 Jakopin, Z. Nucleotide-binding oligomerization domain (NOD) inhibitors: a rational approach toward inhibition of NOD signaling pathway. *J Med Chem* **57**, 6897-6918, doi:10.1021/jm401841p (2014).
44 von Moltke, J., Ayres, J. S., Kofoed, E. M., Chavarria-Smith, J. & Vance, R. E. Recognition of bacteria by inflammasomes. *Annu Rev Immunol* **31**, 73-106, doi:10.1146/annurev-immunol-032712-095944 (2013).
45 Abais, J. M., Xia, M., Zhang, Y., Boini, K. M. & Li, P. L. Redox regulation of NLRP3 inflammasomes: ROS as trigger or effector? *Antioxid Redox Signal* **22**, 1111-1129, doi:10.1089/ars.2014.5994 (2015).
46 Man, S. M. & Kanneganti, T. D. Regulation of inflammasome activation. *Immunol Rev* **265**, 6-21, doi:10.1111/imr.12296 (2015).
47 Rathinam, V. A., Vanaja, S. K. & Fitzgerald, K. A. Regulation of inflammasome signaling. *Nat Immunol* **13**, 333-342, doi:10.1038/ni.2237 (2012).
48 Vandenabeele, P. & Bertrand, M. J. The role of the IAP E3 ubiquitin ligases in regulating pattern-recognition receptor signalling. *Nat Rev Immunol* **12**, 833-844, doi:10.1038/nri3325 (2012).
49 Strober, W., Murray, P. J., Kitani, A. & Watanabe, T. Signalling pathways and molecular interactions of NOD1 and NOD2. *Nat Rev Immunol* **6**, 9-20, doi:10.1038/nri1747 (2006).
50 Rathinam, V. A. *et al.* TRIF licenses caspase-11-dependent NLRP3 inflammasome activation by gram-negative bacteria. *Cell* **150**, 606-619, doi:10.1016/j.cell.2012.07.007 (2012).
51 Hu, H. & Sun, S. C. Ubiquitin signaling in immune responses. *Cell Res* **26**, 457-483, doi:10.1038/cr.2016.40 (2016).
52 Krieg, A. M. AIMing 2 detect foreign DNA. *Sci Signal* **2**, pe39, doi:10.1126/scisignal.277pe39 (2009).
53 Rothenburg, S., Schwartz, T., Koch-Nolte, F. & Haag, F. Complex regulation of the human gene for the Z-DNA binding protein DLM-1. *Nucleic Acids Res* **30**, 993-1000, doi:10.1093/nar/30.4.993 (2002).

54 Takaoka, A. & Taniguchi, T. Cytosolic DNA recognition for triggering innate immune responses. *Adv Drug Deliv Rev* **60**, 847-857, doi:10.1016/j.addr.2007.12.002 (2008).
55 Goubau, D., Deddouche, S. & Reis e Sousa, C. Cytosolic sensing of viruses. *Immunity* **38**, 855-869, doi:10.1016/j.immuni.2013.05.007 (2013).
56 Takaoka, A. *et al.* DAI (DLM-1/ZBP1) is a cytosolic DNA sensor and an activator of innate immune response. *Nature* **448**, 501-505, doi:10.1038/nature06013 (2007).
57 Rebsamen, M. *et al.* DAI/ZBP1 recruits RIP1 and RIP3 through RIP homotypic interaction motifs to activate NF-kappaB. *EMBO Rep* **10**, 916-922, doi:10.1038/embor.2009.109 (2009).
58 O'Neill, L. A. DNA makes RNA makes innate immunity. *Cell* **138**, 428-430, doi:10.1016/j.cell.2009.07.021 (2009).
59 Takaoka, A. & Shinohara, S. DNA sensors in innate immune system. *Uirusu* **58**, 37-46 (2008).
60 Paludan, S. R. & Bowie, A. G. Immune sensing of DNA. *Immunity* **38**, 870-880, doi:10.1016/j.immuni.2013.05.004 (2013).
61 Vilaysane, A. & Muruve, D. A. The innate immune response to DNA. *Semin Immunol* **21**, 208-214, doi:10.1016/j.smim.2009.05.006 (2009).
62 Sharma, S. & Fitzgerald, K. A. Innate immune sensing of DNA. *PLoS Pathog* **7**, e1001310, doi:10.1371/journal.ppat.1001310 (2011).
63 Schroder, K., Muruve, D. A. & Tschopp, J. Innate immunity: cytoplasmic DNA sensing by the AIM2 inflammasome. *Curr Biol* **19**, R262-265, doi:10.1016/j.cub.2009.02.011 (2009).
64 Takeshita, F. & Ishii, K. J. Intracellular DNA sensors in immunity. *Curr Opin Immunol* **20**, 383-388, doi:10.1016/j.coi.2008.05.009 (2008).
65 Cao, X. New DNA-sensing pathway feeds RIG-I with RNA. *Nat Immunol* **10**, 1049-1051, doi:10.1038/ni1009-1049 (2009).
66 Kaiser, W. J., Upton, J. W. & Mocarski, E. S. Receptor-interacting protein homotypic interaction motif-dependent control of NF-kappa B activation via the DNA-dependent activator of IFN regulatory factors. *J Immunol* **181**, 6427-6434, doi:10.4049/jimmunol.181.9.6427 (2008).
67 Wang, Z. *et al.* Regulation of innate immune responses by DAI (DLM-1/ZBP1) and other DNA-sensing molecules. *Proc Natl Acad Sci U S A* **105**, 5477-5482, doi:10.1073/pnas.0801295105 (2008).
68 Yanai, H., Savitsky, D., Tamura, T. & Taniguchi, T. Regulation of the cytosolic DNA-sensing system in innate immunity: a current view. *Curr Opin Immunol* **21**, 17-22, doi:10.1016/j.coi.2009.01.005 (2009).
69 Ishikawa, H., Ma, Z. & Barber, G. N. STING regulates intracellular DNA-mediated, type I interferon-dependent innate immunity. *Nature* **461**, 788-792, doi:10.1038/nature08476 (2009).
70 Ishii, K. J. *et al.* A Toll-like receptor-independent antiviral response induced by double-stranded B-form DNA. *Nat Immunol* **7**, 40-48, doi:10.1038/ni1282 (2006).
71 Stetson, D. B., Ko, J. S., Heidmann, T. & Medzhitov, R. Trex1 prevents cell-intrinsic initiation of autoimmunity. *Cell* **134**, 587-598, doi:10.1016/j.cell.2008.06.032 (2008).
72 Lanier, L. L. DAP10- and DAP12-associated receptors in innate immunity. *Immunol Rev* **227**, 150-160, doi:10.1111/j.1600-065X.2008.00720.x (2009).

73 Liu, D. *et al.* Rapid biogenesis and sensitization of secretory lysosomes in NK cells mediated by target-cell recognition. *Proc Natl Acad Sci U S A* **102**, 123-127, doi:10.1073/pnas.0405737102 (2005).
74 Lieberman, J. The ABCs of granule-mediated cytotoxicity: new weapons in the arsenal. *Nat Rev Immunol* **3**, 361-370, doi:10.1038/nri1083 (2003).
75 Colonna, M. Fine-tuning NK cell responses: it's a family affair. *Nat Immunol* **6**, 961-962, doi:10.1038/ni1005-961 (2005).
76 Lord, S. J., Rajotte, R. V., Korbutt, G. S. & Bleackley, R. C. Granzyme B: a natural born killer. *Immunol Rev* **193**, 31-38, doi:10.1034/j.1600-065x.2003.00044.x (2003).
77 Maghazachi, A. A. Insights into seven and single transmembrane-spanning domain receptors and their signaling pathways in human natural killer cells. *Pharmacol Rev* **57**, 339-357, doi:10.1124/pr.57.3.5 (2005).
78 Colucci, F., Di Santo, J. P. & Leibson, P. J. Natural killer cell activation in mice and men: different triggers for similar weapons? *Nat Immunol* **3**, 807-813, doi:10.1038/ni0902-807 (2002).
79 Vivier, E., Nunes, J. A. & Vely, F. Natural killer cell signaling pathways. *Science* **306**, 1517-1519, doi:10.1126/science.1103478 (2004).
80 Wu, J. & Lanier, L. L. Natural killer cells and cancer. *Adv Cancer Res* **90**, 127-156 (2003).
81 Cerwenka, A. & Lanier, L. L. Natural killer cells, viruses and cancer. *Nat Rev Immunol* **1**, 41-49, doi:10.1038/35095564 (2001).
82 Smyth, M. J. *et al.* Nature's TRAIL--on a path to cancer immunotherapy. *Immunity* **18**, 1-6, doi:10.1016/s1074-7613(02)00502-2 (2003).
83 Tassi, I. *et al.* Phospholipase C-gamma 2 is a critical signaling mediator for murine NK cell activating receptors. *J Immunol* **175**, 749-754, doi:10.4049/jimmunol.175.2.749 (2005).
84 Jevremovic, D., Billadeau, D. D., Schoon, R. A., Dick, C. J. & Leibson, P. J. Regulation of NK cell-mediated cytotoxicity by the adaptor protein 3BP2. *J Immunol* **166**, 7219-7228, doi:10.4049/jimmunol.166.12.7219 (2001).
85 Blott, E. J. & Griffiths, G. M. Secretory lysosomes. *Nat Rev Mol Cell Biol* **3**, 122-131, doi:10.1038/nrm732 (2002).
86 Perussia, B. Signaling for cytotoxicity. *Nat Immunol* **1**, 372-374, doi:10.1038/80808 (2000).
87 Djeu, J. Y., Jiang, K. & Wei, S. A view to a kill: signals triggering cytotoxicity. *Clin Cancer Res* **8**, 636-640 (2002).
88 Bendickova, K., Tidu, F. & Fric, J. Calcineurin-NFAT signalling in myeloid leucocytes: new prospects and pitfalls in immunosuppressive therapy. *EMBO Mol Med* **9**, 990-999, doi:10.15252/emmm.201707698 (2017).
89 Gringhuis, S. I. *et al.* C-type lectin DC-SIGN modulates Toll-like receptor signaling via Raf-1 kinase-dependent acetylation of transcription factor NF-kappaB. *Immunity* **26**, 605-616, doi:10.1016/j.immuni.2007.03.012 (2007).
90 Geijtenbeek, T. B. & Gringhuis, S. I. C-type lectin receptors in the control of T helper cell differentiation. *Nat Rev Immunol* **16**, 433-448, doi:10.1038/nri.2016.55 (2016).
91 Gringhuis, S. I. *et al.* Dectin-1 directs T helper cell differentiation by controlling noncanonical NF-kappaB activation through Raf-1 and Syk. *Nat Immunol* **10**, 203-

213, doi:10.1038/ni.1692 (2009).
92 Brown, G. D. *et al.* Dectin-1 mediates the biological effects of beta-glucans. *J Exp Med* **197**, 1119-1124, doi:10.1084/jem.20021890 (2003).
93 Gringhuis, S. I., Kaptein, T. M., Wevers, B. A., Mesman, A. W. & Geijtenbeek, T. B. Fucose-specific DC-SIGN signalling directs T helper cell type-2 responses via IKKepsilon- and CYLD-dependent Bcl3 activation. *Nat Commun* **5**, 3898, doi:10.1038/ncomms4898 (2014).
94 Iliev, I. D. & Leonardi, I. Fungal dysbiosis: immunity and interactions at mucosal barriers. *Nat Rev Immunol* **17**, 635-646, doi:10.1038/nri.2017.55 (2017).
95 Hajishengallis, G. & Lambris, J. D. Microbial manipulation of receptor crosstalk in innate immunity. *Nat Rev Immunol* **11**, 187-200, doi:10.1038/nri2918 (2011).
96 Dupaul-Chicoine, J. & Saleh, M. A new path to IL-1beta production controlled by caspase-8. *Nat Immunol* **13**, 211-212, doi:10.1038/ni.2241 (2012).
97 Reid, D. M., Gow, N. A. & Brown, G. D. Pattern recognition: recent insights from Dectin-1. *Curr Opin Immunol* **21**, 30-37, doi:10.1016/j.coi.2009.01.003 (2009).
98 Marakalala, M. J. & Ndlovu, H. Signaling C-type lectin receptors in antimycobacterial immunity. *PLoS Pathog* **13**, e1006333, doi:10.1371/journal.ppat.1006333 (2017).
99 Geijtenbeek, T. B. & Gringhuis, S. I. Signalling through C-type lectin receptors: shaping immune responses. *Nat Rev Immunol* **9**, 465-479, doi:10.1038/nri2569 (2009).
100 Gold, E. S. *et al.* Amphiphysin IIm, a novel amphiphysin II isoform, is required for macrophage phagocytosis. *Immunity* **12**, 285-292, doi:10.1016/s1074-7613(00)80181-8 (2000).
101 Gu, H., Botelho, R. J., Yu, M., Grinstein, S. & Neel, B. G. Critical role for scaffolding adapter Gab2 in Fc gamma R-mediated phagocytosis. *J Cell Biol* **161**, 1151-1161, doi:10.1083/jcb.200212158 (2003).
102 Nimmerjahn, F. & Ravetch, J. V. Fcgamma receptors: old friends and new family members. *Immunity* **24**, 19-28, doi:10.1016/j.immuni.2005.11.010 (2006).
103 Ravetch, J. V. & Bolland, S. IgG Fc receptors. *Annu Rev Immunol* **19**, 275-290, doi:10.1146/annurev.immunol.19.1.275 (2001).
104 Groves, E., Dart, A. E., Covarelli, V. & Caron, E. Molecular mechanisms of phagocytic uptake in mammalian cells. *Cell Mol Life Sci* **65**, 1957-1976, doi:10.1007/s00018-008-7578-4 (2008).
105 Uchida, H., Kondo, A., Yoshimura, Y., Mazaki, Y. & Sabe, H. PAG3/Papalpha/KIAA0400, a GTPase-activating protein for ADP-ribosylation factor (ARF), regulates ARF6 in Fcgamma receptor-mediated phagocytosis of macrophages. *J Exp Med* **193**, 955-966, doi:10.1084/jem.193.8.955 (2001).
106 Cox, D. & Greenberg, S. Phagocytic signaling strategies: Fc(gamma)receptor-mediated phagocytosis as a model system. *Semin Immunol* **13**, 339-345, doi:10.1006/smim.2001.0330 (2001).
107 Bompard, G. & Caron, E. Regulation of WASP/WAVE proteins: making a long story short. *J Cell Biol* **166**, 957-962, doi:10.1083/jcb.200403127 (2004).
108 Lee, W. L., Cosio, G., Ireton, K. & Grinstein, S. Role of CrkII in Fcgamma receptor-mediated phagocytosis. *J Biol Chem* **282**, 11135-11143, doi:10.1074/jbc.M700823200 (2007).

109 Ganesan, L. P. *et al.* The serine/threonine kinase Akt Promotes Fc gamma receptor-mediated phagocytosis in murine macrophages through the activation of p70S6 kinase. *J Biol Chem* **279**, 54416-54425, doi:10.1074/jbc.M408188200 (2004).

110 Alvarez-Errico, D., Lessmann, E. & Rivera, J. Adapters in the organization of mast cell signaling. *Immunol Rev* **232**, 195-217, doi:10.1111/j.1600-065X.2009.00834.x (2009).

111 Klemm, S. *et al.* The Bcl10-Malt1 complex segregates Fc epsilon RI-mediated nuclear factor kappa B activation and cytokine production from mast cell degranulation. *J Exp Med* **203**, 337-347, doi:10.1084/jem.20051982 (2006).

112 Gu, H. *et al.* Essential role for Gab2 in the allergic response. *Nature* **412**, 186-190, doi:10.1038/35084076 (2001).

113 Wu, L. C. Immunoglobulin E receptor signaling and asthma. *J Biol Chem* **286**, 32891-32897, doi:10.1074/jbc.R110.205104 (2011).

114 Blank, U. & Rivera, J. The ins and outs of IgE-dependent mast-cell exocytosis. *Trends Immunol* **25**, 266-273, doi:10.1016/j.it.2004.03.005 (2004).

115 Gilfillan, A. M. & Tkaczyk, C. Integrated signalling pathways for mast-cell activation. *Nat Rev Immunol* **6**, 218-230, doi:10.1038/nri1782 (2006).

116 Siraganian, R. P. Mast cell signal transduction from the high-affinity IgE receptor. *Curr Opin Immunol* **15**, 639-646, doi:10.1016/j.coi.2003.09.010 (2003).

117 Kambayashi, T. & Koretzky, G. A. Proximal signaling events in Fc epsilon RI-mediated mast cell activation. *J Allergy Clin Immunol* **119**, 544-552; quiz 553-544, doi:10.1016/j.jaci.2007.01.017 (2007).

118 Ebinu, J. O. *et al.* RasGRP links T-cell receptor signaling to Ras. *Blood* **95**, 3199-3203 (2000).

119 Kawakami, T. & Galli, S. J. Regulation of mast-cell and basophil function and survival by IgE. *Nat Rev Immunol* **2**, 773-786, doi:10.1038/nri914 (2002).

120 Nadler, M. J., Matthews, S. A., Turner, H. & Kinet, J. P. Signal transduction by the high-affinity immunoglobulin E receptor Fc epsilon RI: coupling form to function. *Adv Immunol* **76**, 325-355, doi:10.1016/s0065-2776(01)76022-1 (2000).

121 Turner, H. & Kinet, J. P. Signalling through the high-affinity IgE receptor Fc epsilonRI. *Nature* **402**, B24-30, doi:10.1038/35037021 (1999).

122 Gilfillan, A. M. & Rivera, J. The tyrosine kinase network regulating mast cell activation. *Immunol Rev* **228**, 149-169, doi:10.1111/j.1600-065X.2008.00742.x (2009).

123 Nadler, M. J. & Kinet, J. P. Uncovering new complexities in mast cell signaling. *Nat Immunol* **3**, 707-708, doi:10.1038/ni0802-707 (2002).

124 Wright, H. L., Moots, R. J., Bucknall, R. C. & Edwards, S. W. Neutrophil function in inflammation and inflammatory diseases. *Rheumatology (Oxford)* **49**, 1618-1631, doi:10.1093/rheumatology/keq045 (2010).

125 Borregaard, N., Sorensen, O. E. & Theilgaard-Monch, K. Neutrophil granules: a library of innate immunity proteins. *Trends Immunol* **28**, 340-345, doi:10.1016/j.it.2007.06.002 (2007).

126 Zlotnik, A. & Yoshie, O. Chemokines: a new classification system and their role in immunity. *Immunity* **12**, 121-127, doi:10.1016/s1074-7613(00)80165-x (2000).

127 Ozaki, K. & Leonard, W. J. Cytokine and cytokine receptor pleiotropy and

redundancy. *J Biol Chem* **277**, 29355-29358, doi:10.1074/jbc.R200003200 (2002).

128 Brooks, A. J. & Waters, M. J. The growth hormone receptor: mechanism of activation and clinical implications. *Nat Rev Endocrinol* **6**, 515-525, doi:10.1038/nrendo.2010.123 (2010).

129 Massague, J. How cells read TGF-beta signals. *Nat Rev Mol Cell Biol* **1**, 169-178, doi:10.1038/35043051 (2000).

130 Gough, D. J., Levy, D. E., Johnstone, R. W. & Clarke, C. J. IFNgamma signaling-does it mean JAK-STAT? *Cytokine Growth Factor Rev* **19**, 383-394, doi:10.1016/j.cytogfr.2008.08.004 (2008).

131 Dinarello, C. A. Immunological and inflammatory functions of the interleukin-1 family. *Annu Rev Immunol* **27**, 519-550, doi:10.1146/annurev.immunol.021908.132612 (2009).

132 Pestka, S. *et al.* Interleukin-10 and related cytokines and receptors. *Annu Rev Immunol* **22**, 929-979, doi:10.1146/annurev.immunol.22.012703.104622 (2004).

133 Moseley, T. A., Haudenschild, D. R., Rose, L. & Reddi, A. H. Interleukin-17 family and IL-17 receptors. *Cytokine Growth Factor Rev* **14**, 155-174 (2003).

134 Vosshenrich, C. A. & Di Santo, J. P. Interleukin signaling. *Curr Biol* **12**, R760-763, doi:10.1016/s0960-9822(02)01286-1 (2002).

135 Akdis, M. *et al.* Interleukins (from IL-1 to IL-38), interferons, transforming growth factor beta, and TNF-alpha: Receptors, functions, and roles in diseases. *J Allergy Clin Immunol* **138**, 984-1010, doi:10.1016/j.jaci.2016.06.033 (2016).

136 Platanias, L. C. Mechanisms of type-I- and type-II-interferon-mediated signalling. *Nat Rev Immunol* **5**, 375-386, doi:10.1038/nri1604 (2005).

137 Bodmer, J. L., Schneider, P. & Tschopp, J. The molecular architecture of the TNF superfamily. *Trends Biochem Sci* **27**, 19-26, doi:10.1016/s0968-0004(01)01995-8 (2002).

138 Razani, B., Reichardt, A. D. & Cheng, G. Non-canonical NF-kappaB signaling activation and regulation: principles and perspectives. *Immunol Rev* **244**, 44-54, doi:10.1111/j.1600-065X.2011.01059.x (2011).

139 Sun, S. C. Non-canonical NF-kappaB signaling pathway. *Cell Res* **21**, 71-85, doi:10.1038/cr.2010.177 (2011).

140 Li, X. & Eriksson, U. Novel PDGF family members: PDGF-C and PDGF-D. *Cytokine Growth Factor Rev* **14**, 91-98 (2003).

141 Bole-Feysot, C., Goffin, V., Edery, M., Binart, N. & Kelly, P. A. Prolactin (PRL) and its receptor: actions, signal transduction pathways and phenotypes observed in PRL receptor knockout mice. *Endocr Rev* **19**, 225-268, doi:10.1210/edrv.19.3.0334 (1998).

142 Goffin, V., Binart, N., Touraine, P. & Kelly, P. A. Prolactin: the new biology of an old hormone. *Annu Rev Physiol* **64**, 47-67, doi:10.1146/annurev.physiol.64.081501.131049 (2002).

143 Silke, J. & Brink, R. Regulation of TNFRSF and innate immune signalling complexes by TRAFs and cIAPs. *Cell Death Differ* **17**, 35-45, doi:10.1038/cdd.2009.114 (2010).

144 Zachary, I. & Gliki, G. Signaling transduction mechanisms mediating biological actions of the vascular endothelial growth factor family. *Cardiovasc Res* **49**, 568-581, doi:10.1016/s0008-6363(00)00268-6 (2001).

145 Martin, M. U. & Wesche, H. Summary and comparison of the signaling mechanisms of the Toll/interleukin-1 receptor family. *Biochim Biophys Acta* **1592**, 265-280, doi:10.1016/s0167-4889(02)00320-8 (2002).
146 Faustman, D. & Davis, M. TNF receptor 2 pathway: drug target for autoimmune diseases. *Nat Rev Drug Discov* **9**, 482-493, doi:10.1038/nrd3030 (2010).
147 de Jong, A. Activation of human T cells by CD1 and self-lipids. *Immunol. Rev.* **267**, 16-29, doi:10.1111/imr.12322 (2015).
148 Moody, D. B., Zajonc, D. M. & Wilson, I. A. Anatomy of CD1-lipid antigen complexes. *Nat. Rev. Immunol.* **5**, 387-399, doi:10.1038/nri1605 (2005).
149 Murphy, K. M., Nelson, C. A. & Sedy, J. R. Balancing co-stimulation and inhibition with BTLA and HVEM. *Nat Rev Immunol* **6**, 671-681, doi:10.1038/nri1917 (2006).
150 Watanabe, N. *et al.* BTLA is a lymphocyte inhibitory receptor with similarities to CTLA-4 and PD-1. *Nat Immunol* **4**, 670-679, doi:10.1038/ni944 (2003).
151 Abeler-Dorner, L., Swamy, M., Williams, G., Hayday, A. C. & Bas, A. Butyrophilins: an emerging family of immune regulators. *Trends Immunol* **33**, 34-41, doi:10.1016/j.it.2011.09.007 (2012).
152 Thome, M. CARMA1, BCL-10 and MALT1 in lymphocyte development and activation. *Nat Rev Immunol* **4**, 348-359, doi:10.1038/nri1352 (2004).
153 Blonska, M. & Lin, X. CARMA1-mediated NF-kappaB and JNK activation in lymphocytes. *Immunol. Rev.* **228**, 199-211, doi:10.1111/j.1600-065X.2008.00749.x (2009).
154 Duan, L., Reddi, A. L., Ghosh, A., Dimri, M. & Band, H. The Cbl family and other ubiquitin ligases: destructive forces in control of antigen receptor signaling. *Immunity* **21**, 7-17, doi:10.1016/j.immuni.2004.06.012 (2004).
155 Van Rhijn, I. & Moody, D. B. CD1 and mycobacterial lipids activate human T cells. *Immunol. Rev.* **264**, 138-153, doi:10.1111/imr.12253 (2015).
156 Barral, D. C. & Brenner, M. B. CD1 antigen presentation: how it works. *Nat. Rev. Immunol.* **7**, 929-941, doi:10.1038/nri2191 (2007).
157 Sieling, P. A. *et al.* CD1-restricted T cell recognition of microbial lipoglycan antigens. *Science* **269**, 227-230, doi:10.1126/science.7542404 (1995).
158 Michel, F. & Acuto, O. CD28 costimulation: a source of Vav-1 for TCR signaling with the help of SLP-76? *Sci STKE* **2002**, pe35, doi:10.1126/stke.2002.144.pe35 (2002).
159 Zikherman, J. *et al.* CD45-Csk phosphatase-kinase titration uncouples basal and inducible T cell receptor signaling during thymic development. *Immunity* **32**, 342-354, doi:10.1016/j.immuni.2010.03.006 (2010).
160 Chen, L. Co-inhibitory molecules of the B7-CD28 family in the control of T-cell immunity. *Nat. Rev. Immunol.* **4**, 336-347, doi:10.1038/nri1349 (2004).
161 Fife, B. T. & Bluestone, J. A. Control of peripheral T-cell tolerance and autoimmunity via the CTLA-4 and PD-1 pathways. *Immunol Rev* **224**, 166-182, doi:10.1111/j.1600-065X.2008.00662.x (2008).
162 Rebeaud, F., Hailfinger, S. & Thome, M. Dlgh1 and Carma1 MAGUK proteins contribute to signal specificity downstream of TCR activation. *Trends Immunol.* **28**, 196-200, doi:10.1016/j.it.2007.03.004 (2007).
163 Ogg, S. L., Weldon, A. K., Dobbie, L., Smith, A. J. & Mather, I. H. Expression of butyrophilin (Btn1a1) in lactating mammary gland is essential for the regulated

secretion of milk-lipid droplets. *Proc Natl Acad Sci U S A* **101**, 10084-10089, doi:10.1073/pnas.0402930101 (2004).

164 Cronin, S. J. & Penninger, J. M. From T-cell activation signals to signaling control of anti-cancer immunity. *Immunol Rev* **220**, 151-168, doi:10.1111/j.1600-065X.2007.00570.x (2007).

165 Diehn, M. *et al.* Genomic expression programs and the integration of the CD28 costimulatory signal in T cell activation. *Proc. Natl. Acad. Sci. U.S.A.* **99**, 11796-11801, doi:10.1073/pnas.092284399 (2002).

166 Arnett, H. A. & Viney, J. L. Immune modulation by butyrophilins. *Nat Rev Immunol* **14**, 559-569, doi:10.1038/nri3715 (2014).

167 Sechi, A. S. & Wehland, J. Interplay between TCR signalling and actin cytoskeleton dynamics. *Trends Immunol.* **25**, 257-265, doi:10.1016/j.it.2004.03.003 (2004).

168 Gilmore, T. D. Introduction to NF-kappaB: players, pathways, perspectives. *Oncogene* **25**, 6680-6684, doi:10.1038/sj.onc.1209954 (2006).

169 Kane, L. P., Lin, J. & Weiss, A. It's all Rel-ative: NF-kappaB and CD28 costimulation of T-cell activation. *Trends Immunol.* **23**, 413-420 (2002).

170 Qi, Q. & August, A. Keeping the (kinase) party going: SLP-76 and ITK dance to the beat. *Sci STKE* **2007**, pe39, doi:10.1126/stke.3962007pe39 (2007).

171 Cordoba, S. P. *et al.* The large ectodomains of CD45 and CD148 regulate their segregation from and inhibition of ligated T-cell receptor. *Blood* **121**, 4295-4302, doi:10.1182/blood-2012-07-442251 (2013).

172 Wange, R. L. LAT, the linker for activation of T cells: a bridge between T cell-specific and general signaling pathways. *Sci STKE* **2000**, re1, doi:10.1126/stke.2000.63.re1 (2000).

173 Vang, T. *et al.* LYP inhibits T-cell activation when dissociated from CSK. *Nat Chem Biol* **8**, 437-446, doi:10.1038/nchembio.916 (2012).

174 Ashwell, J. D. The many paths to p38 mitogen-activated protein kinase activation in the immune system. *Nat. Rev. Immunol.* **6**, 532-540, doi:10.1038/nri1865 (2006).

175 Das, S., Dixon, J. E. & Cho, W. Membrane-binding and activation mechanism of PTEN. *Proc Natl Acad Sci U S A* **100**, 7491-7496, doi:10.1073/pnas.0932835100 (2003).

176 Teft, W. A., Kirchhof, M. G. & Madrenas, J. A molecular perspective of CTLA-4 function. *Annu Rev Immunol* **24**, 65-97, doi:10.1146/annurev.immunol.24.021605.090535 (2006).

177 Schmitz, M. L., Bacher, S. & Dienz, O. NF-kappaB activation pathways induced by T cell costimulation. *Faseb j.* **17**, 2187-2193, doi:10.1096/fj.02-1100rev (2003).

178 Keir, M. E., Butte, M. J., Freeman, G. J. & Sharpe, A. H. PD-1 and its ligands in tolerance and immunity. *Annu Rev Immunol* **26**, 677-704, doi:10.1146/annurev.immunol.26.021607.090331 (2008).

179 Brdicka, T. *et al.* Phosphoprotein associated with glycosphingolipid-enriched microdomains (PAG), a novel ubiquitously expressed transmembrane adaptor protein, binds the protein tyrosine kinase csk and is involved in regulation of T cell activation. *J Exp Med* **191**, 1591-1604, doi:10.1084/jem.191.9.1591 (2000).

180 Shambharkar, P. B. *et al.* Phosphorylation and ubiquitination of the IkappaB kinase complex by two distinct signaling pathways. *Embo j* **26**, 1794-1805, doi:10.1038/sj.emboj.7601622 (2007).

181 Rueda, D. & Thome, M. Phosphorylation of CARMA1: the link(er) to NF-kappaB activation. *Immunity* **23**, 551-553, doi:10.1016/j.immuni.2005.11.007 (2005).
182 Okkenhaug, K. & Vanhaesebroeck, B. PI3K in lymphocyte development, differentiation and activation. *Nat. Rev. Immunol.* **3**, 317-330, doi:10.1038/nri1056 (2003).
183 Thome, M. & Weil, R. Post-translational modifications regulate distinct functions of CARMA1 and BCL10. *Trends Immunol* **28**, 281-288, doi:10.1016/j.it.2007.04.004 (2007).
184 Zajonc, D. M. & Girardi, E. Recognition of Microbial Glycolipids by Natural Killer T Cells. *Front Immunol* **6**, 400, doi:10.3389/fimmu.2015.00400 (2015).
185 Hacker, H. & Karin, M. Regulation and function of IKK and IKK-related kinases. *Sci STKE* **2006**, re13, doi:10.1126/stke.3572006re13 (2006).
186 Rhodes, D. A., Reith, W. & Trowsdale, J. Regulation of Immunity by Butyrophilins. *Annu Rev Immunol* **34**, 151-172, doi:10.1146/annurev-immunol-041015-055435 (2016).
187 Stepanek, O. *et al.* Regulation of Src family kinases involved in T cell receptor signaling by protein-tyrosine phosphatase CD148. *J Biol Chem* **286**, 22101-22112, doi:10.1074/jbc.M110.196733 (2011).
188 March, M. E. & Ravichandran, K. Regulation of the immune response by SHIP. *Semin Immunol* **14**, 37-47, doi:10.1006/smim.2001.0340 (2002).
189 Liu, S. K., Berry, D. M. & McGlade, C. J. The role of Gads in hematopoietic cell signalling. *Oncogene* **20**, 6284-6290, doi:10.1038/sj.onc.1204771 (2001).
190 Koyasu, S. The role of PI3K in immune cells. *Nat Immunol* **4**, 313-319, doi:10.1038/ni0403-313 (2003).
191 Lin, X. & Wang, D. The roles of CARMA1, Bcl10, and MALT1 in antigen receptor signaling. *Semin Immunol* **16**, 429-435, doi:10.1016/j.smim.2004.08.022 (2004).
192 Round, J. L. *et al.* Scaffold protein Dlgh1 coordinates alternative p38 kinase activation, directing T cell receptor signals toward NFAT but not NF-kappaB transcription factors. *Nat. Immunol.* **8**, 154-161, doi:10.1038/ni1422 (2007).
193 Manicassamy, S., Gupta, S. & Sun, Z. Selective function of PKC-theta in T cells. *Cell Mol Immunol* **3**, 263-270 (2006).
194 Hayden, M. S. & Ghosh, S. Signaling to NF-kappaB. *Genes Dev* **18**, 2195-2224, doi:10.1101/gad.1228704 (2004).
195 Harada, Y. *et al.* A single amino acid alteration in cytoplasmic domain determines IL-2 promoter activation by ligation of CD28 but not inducible costimulator (ICOS). *J Exp Med* **197**, 257-262, doi:10.1084/jem.20021305 (2003).
196 Silk, J. D., Salio, M., Brown, J., Jones, E. Y. & Cerundolo, V. Structural and functional aspects of lipid binding by CD1 molecules. *Annu. Rev. Cell Dev. Biol.* **24**, 369-395, doi:10.1146/annurev.cellbio.24.110707.175359 (2008).
197 Rohrschneider, L. R., Fuller, J. F., Wolf, I., Liu, Y. & Lucas, D. M. Structure, function, and biology of SHIP proteins. *Genes Dev* **14**, 505-520 (2000).
198 Schrum, A. G., Turka, L. A. & Palmer, E. Surface T-cell antigen receptor expression and availability for long-term antigenic signaling. *Immunol. Rev.* **196**, 7-24, doi:10.1046/j.1600-065x.2003.00083.x (2003).
199 Smith-Garvin, J. E., Koretzky, G. A. & Jordan, M. S. T cell activation. *Annu. Rev.*

Immunol. **27**, 591-619, doi:10.1146/annurev.immunol.021908.132706 (2009).
200 Huang, Y. & Wange, R. L. T cell receptor signaling: beyond complex complexes. *J. Biol. Chem.* **279**, 28827-28830, doi:10.1074/jbc.R400012200 (2004).
201 Alegre, M. L., Frauwirth, K. A. & Thompson, C. B. T-cell regulation by CD28 and CTLA-4. *Nat Rev Immunol* **1**, 220-228, doi:10.1038/35105024 (2001).
202 Baniyash, M. TCR zeta-chain downregulation: curtailing an excessive inflammatory immune response. *Nat. Rev. Immunol.* **4**, 675-687, doi:10.1038/nri1434 (2004).
203 Berg, L. J., Finkelstein, L. D., Lucas, J. A. & Schwartzberg, P. L. Tec family kinases in T lymphocyte development and function. *Annu. Rev. Immunol.* **23**, 549-600, doi:10.1146/annurev.immunol.22.012703.104743 (2005).
204 Finkelstein, L. D. & Schwartzberg, P. L. Tec kinases: shaping T-cell activation through actin. *Trends Cell Biol.* **14**, 443-451, doi:10.1016/j.tcb.2003.07.001 (2004).
205 Sun, L., Deng, L., Ea, C. K., Xia, Z. P. & Chen, Z. J. The TRAF6 ubiquitin ligase and TAK1 kinase mediate IKK activation by BCL10 and MALT1 in T lymphocytes. *Mol Cell* **14**, 289-301, doi:10.1016/s1097-2765(04)00236-9 (2004).
206 Horejsí, V., Zhang, W. & Schraven, B. Transmembrane adaptor proteins: organizers of immunoreceptor signalling. *Nat. Rev. Immunol.* **4**, 603-616, doi:10.1038/nri1414 (2004).
207 Bonizzi, G. & Karin, M. The two NF-kappaB activation pathways and their role in innate and adaptive immunity. *Trends Immunol* **25**, 280-288, doi:10.1016/j.it.2004.03.008 (2004).
208 Adhikari, A., Xu, M. & Chen, Z. J. Ubiquitin-mediated activation of TAK1 and IKK. *Oncogene* **26**, 3214-3226, doi:10.1038/sj.onc.1210413 (2007).
209 Rudd, C. E. & Schneider, H. Unifying concepts in CD28, ICOS and CTLA4 co-receptor signalling. *Nat Rev Immunol* **3**, 544-556, doi:10.1038/nri1131 (2003).
210 Jumaa, H., Hendriks, R. W. & Reth, M. B cell signaling and tumorigenesis. *Annu Rev Immunol* **23**, 415-445, doi:10.1146/annurev.immunol.23.021704.115606 (2005).
211 Okada, T., Maeda, A., Iwamatsu, A., Gotoh, K. & Kurosaki, T. BCAP: the tyrosine kinase substrate that connects B cell receptor to phosphoinositide 3-kinase activation. *Immunity* **13**, 817-827, doi:10.1016/s1074-7613(00)00079-0 (2000).
212 Baba, Y. *et al.* BLNK mediates Syk-dependent Btk activation. *Proc Natl Acad Sci U S A* **98**, 2582-2586, doi:10.1073/pnas.051626198 (2001).
213 Poe, J. C. *et al.* CD22 regulates B lymphocyte function in vivo through both ligand-dependent and ligand-independent mechanisms. *Nat Immunol* **5**, 1078-1087, doi:10.1038/ni1121 (2004).
214 Shinohara, H. & Kurosaki, T. Comprehending the complex connection between PKCbeta, TAK1, and IKK in BCR signaling. *Immunol Rev* **232**, 300-318, doi:10.1111/j.1600-065X.2009.00836.x (2009).
215 Harwood, N. E. & Batista, F. D. Early events in B cell activation. *Annu Rev Immunol* **28**, 185-210, doi:10.1146/annurev-immunol-030409-101216 (2010).
216 Shinohara, H., Maeda, S., Watarai, H. & Kurosaki, T. IkappaB kinase beta-induced phosphorylation of CARMA1 contributes to CARMA1 Bcl10 MALT1 complex formation in B cells. *J Exp Med* **204**, 3285-3293, doi:10.1084/jem.20070379 (2007).
217 Teixeira, C., Stang, S. L., Zheng, Y., Beswick, N. S. & Stone, J. C. Integration of DAG signaling systems mediated by PKC-dependent phosphorylation of RasGRP3. *Blood*

102, 1414-1420, doi:10.1182/blood-2002-11-3621 (2003).
218 Nitschke, L. & Tsubata, T. Molecular interactions regulate BCR signal inhibition by CD22 and CD72. *Trends Immunol* **25**, 543-550, doi:10.1016/j.it.2004.08.002 (2004).
219 Marshall, A. J. *et al.* A novel B lymphocyte-associated adaptor protein, Bam32, regulates antigen receptor signaling downstream of phosphatidylinositol 3-kinase. *J Exp Med* **191**, 1319-1332, doi:10.1084/jem.191.8.1319 (2000).
220 Luik, R. M., Wang, B., Prakriya, M., Wu, M. M. & Lewis, R. S. Oligomerization of STIM1 couples ER calcium depletion to CRAC channel activation. *Nature* **454**, 538-542, doi:10.1038/nature07065 (2008).
221 Lorenzo, P. S. *et al.* Phorbol esters modulate the Ras exchange factor RasGRP3. *Cancer Res* **61**, 943-949 (2001).
222 Zheng, Y. *et al.* Phosphorylation of RasGRP3 on threonine 133 provides a mechanistic link between PKC and Ras signaling systems in B cells. *Blood* **105**, 3648-3654, doi:10.1182/blood-2004-10-3916 (2005).
223 Sommer, K. *et al.* Phosphorylation of the CARMA1 linker controls NF-kappaB activation. *Immunity* **23**, 561-574, doi:10.1016/j.immuni.2005.09.014 (2005).
224 Shinohara, H. *et al.* PKC beta regulates BCR-mediated IKK activation by facilitating the interaction between TAK1 and CARMA1. *J Exp Med* **202**, 1423-1431, doi:10.1084/jem.20051591 (2005).
225 Rickert, R. C. Regulation of B lymphocyte activation by complement C3 and the B cell coreceptor complex. *Curr Opin Immunol* **17**, 237-243, doi:10.1016/j.coi.2005.03.001 (2005).
226 Niiro, H. & Clark, E. A. Regulation of B-cell fate by antigen-receptor signals. *Nat Rev Immunol* **2**, 945-956, doi:10.1038/nri955 (2002).
227 Kurosaki, T. Regulation of B-cell signal transduction by adaptor proteins. *Nat Rev Immunol* **2**, 354-363, doi:10.1038/nri801 (2002).
228 Nitschke, L. The role of CD22 and other inhibitory co-receptors in B-cell activation. *Curr Opin Immunol* **17**, 290-297, doi:10.1016/j.coi.2005.03.005 (2005).
229 Gauld, S. B. & Cambier, J. C. Src-family kinases in B-cell development and signaling. *Oncogene* **23**, 8001-8006, doi:10.1038/sj.onc.1208075 (2004).
230 Park, C. Y. *et al.* STIM1 clusters and activates CRAC channels via direct binding of a cytosolic domain to Orai1. *Cell* **136**, 876-890, doi:10.1016/j.cell.2009.02.014 (2009).
231 Wang, C. *et al.* TAK1 is a ubiquitin-dependent kinase of MKK and IKK. *Nature* **412**, 346-351, doi:10.1038/35085597 (2001).
232 Gold, M. R. To make antibodies or not: signaling by the B-cell antigen receptor. *Trends Pharmacol Sci* **23**, 316-324, doi:10.1016/s0165-6147(02)02045-x (2002).
233 Mori, Y. *et al.* Transient receptor potential 1 regulates capacitative Ca(2+) entry and Ca(2+) release from endoplasmic reticulum in B lymphocytes. *J Exp Med* **195**, 673-681, doi:10.1084/jem.20011758 (2002).
234 Shiratori, I., Ogasawara, K., Saito, T., Lanier, L. L. & Arase, H. Activation of natural killer cells and dendritic cells upon recognition of a novel CD99-like ligand by paired immunoglobulin-like type 2 receptor. *J Exp Med* **199**, 525-533, doi:10.1084/jem.20031885 (2004).
235 Kaufmann, S. H. & Schaible, U. E. Antigen presentation and recognition in bacterial infections. *Curr Opin Immunol* **17**, 79-87, doi:10.1016/j.coi.2004.12.004 (2005).

236 Loureiro, J. & Ploegh, H. L. Antigen presentation and the ubiquitin-proteasome system in host-pathogen interactions. *Adv Immunol* **92**, 225-305, doi:10.1016/s0065-2776(06)92006-9 (2006).
237 Antoniou, A. N., Powis, S. J. & Elliott, T. Assembly and export of MHC class I peptide ligands. *Curr Opin Immunol* **15**, 75-81, doi:10.1016/s0952-7915(02)00010-9 (2003).
238 Kaifu, T., Escaliere, B., Gastinel, L. N., Vivier, E. & Baratin, M. B7-H6/NKp30 interaction: a mechanism of alerting NK cells against tumors. *Cell Mol Life Sci* **68**, 3531-3539, doi:10.1007/s00018-011-0802-7 (2011).
239 Binici, J. & Koch, J. BAG-6, a jack of all trades in health and disease. *Cell Mol Life Sci* **71**, 1829-1837, doi:10.1007/s00018-013-1522-y (2014).
240 Reith, W. & Mach, B. The bare lymphocyte syndrome and the regulation of MHC expression. *Annu Rev Immunol* **19**, 331-373, doi:10.1146/annurev.immunol.19.1.331 (2001).
241 Tabata, S. *et al.* Biophysical characterization of O-glycosylated CD99 recognition by paired Ig-like type 2 receptors. *J Biol Chem* **283**, 8893-8901, doi:10.1074/jbc.M709793200 (2008).
242 Chen, K. *et al.* CD40/CD40L dyad in the inflammatory and immune responses in the central nervous system. *Cell Mol Immunol* **3**, 163-169 (2006).
243 Clark, G. J., Ju, X., Tate, C. & Hart, D. N. The CD300 family of molecules are evolutionarily significant regulators of leukocyte functions. *Trends Immunol* **30**, 209-217, doi:10.1016/j.it.2009.02.003 (2009).
244 Trombetta, E. S. & Mellman, I. Cell biology of antigen processing in vitro and in vivo. *Annu Rev Immunol* **23**, 975-1028, doi:10.1146/annurev.immunol.22.012703.104538 (2005).
245 Ackerman, A. L. & Cresswell, P. Cellular mechanisms governing cross-presentation of exogenous antigens. *Nat Immunol* **5**, 678-684, doi:10.1038/ni1082 (2004).
246 Lebbink, R. J. *et al.* Collagens are functional, high affinity ligands for the inhibitory immune receptor LAIR-1. *J Exp Med* **203**, 1419-1425, doi:10.1084/jem.20052554 (2006).
247 Elliott, T. & Neefjes, J. The complex route to MHC class I-peptide complexes. *Cell* **127**, 249-251, doi:10.1016/j.cell.2006.10.001 (2006).
248 Heath, W. R. & Carbone, F. R. Cross-presentation in viral immunity and self-tolerance. *Nat Rev Immunol* **1**, 126-134, doi:10.1038/35100512 (2001).
249 Rock, K. L. & Shen, L. Cross-presentation: underlying mechanisms and role in immune surveillance. *Immunol Rev* **207**, 166-183, doi:10.1111/j.0105-2896.2005.00301.x (2005).
250 Monu, N. & Trombetta, E. S. Cross-talk between the endocytic pathway and the endoplasmic reticulum in cross-presentation by MHC class I molecules. *Curr Opin Immunol* **19**, 66-72, doi:10.1016/j.coi.2006.11.017 (2007).
251 Delamarre, L., Pack, M., Chang, H., Mellman, I. & Trombetta, E. S. Differential lysosomal proteolysis in antigen-presenting cells determines antigen fate. *Science* **307**, 1630-1634, doi:10.1126/science.1108003 (2005).
252 Zika, E. & Ting, J. P. Epigenetic control of MHC-II: interplay between CIITA and histone-modifying enzymes. *Curr Opin Immunol* **17**, 58-64, doi:10.1016/j.coi.2004.11.008 (2005).

253 Sun, Y. *et al.* Evolutionarily conserved paired immunoglobulin-like receptor alpha (PILRalpha) domain mediates its interaction with diverse sialylated ligands. *J Biol Chem* **287**, 15837-15850, doi:10.1074/jbc.M111.286633 (2012).
254 Watts, C. The exogenous pathway for antigen presentation on major histocompatibility complex class II and CD1 molecules. *Nat Immunol* **5**, 685-692, doi:10.1038/ni1088 (2004).
255 Griewank, K. *et al.* Homotypic interactions mediated by Slamf1 and Slamf6 receptors control NKT cell lineage development. *Immunity* **27**, 751-762, doi:10.1016/j.immuni.2007.08.020 (2007).
256 Pogge von Strandmann, E. *et al.* Human leukocyte antigen-B-associated transcript 3 is released from tumor cells and engages the NKp30 receptor on natural killer cells. *Immunity* **27**, 965-974, doi:10.1016/j.immuni.2007.10.010 (2007).
257 Amigorena, S. & Savina, A. Intracellular mechanisms of antigen cross presentation in dendritic cells. *Curr Opin Immunol* **22**, 109-117, doi:10.1016/j.coi.2010.01.022 (2010).
258 Vilches, C. & Parham, P. KIR: diverse, rapidly evolving receptors of innate and adaptive immunity. *Annu Rev Immunol* **20**, 217-251, doi:10.1146/annurev.immunol.20.092501.134942 (2002).
259 Vyas, J. M., Van der Veen, A. G. & Ploegh, H. L. The known unknowns of antigen processing and presentation. *Nat Rev Immunol* **8**, 607-618, doi:10.1038/nri2368 (2008).
260 Honey, K. & Rudensky, A. Y. Lysosomal cysteine proteases regulate antigen presentation. *Nat Rev Immunol* **3**, 472-482, doi:10.1038/nri1110 (2003).
261 Cresswell, P., Ackerman, A. L., Giodini, A., Peaper, D. R. & Wearsch, P. A. Mechanisms of MHC class I-restricted antigen processing and cross-presentation. *Immunol Rev* **207**, 145-157, doi:10.1111/j.0105-2896.2005.00316.x (2005).
262 Ramachandra, L., Simmons, D. & Harding, C. V. MHC molecules and microbial antigen processing in phagosomes. *Curr Opin Immunol* **21**, 98-104, doi:10.1016/j.coi.2009.01.001 (2009).
263 Sercarz, E. E. & Maverakis, E. Mhc-guided processing: binding of large antigen fragments. *Nat Rev Immunol* **3**, 621-629, doi:10.1038/nri1149 (2003).
264 Purcell, A. W. & Elliott, T. Molecular machinations of the MHC-I peptide loading complex. *Curr Opin Immunol* **20**, 75-81, doi:10.1016/j.coi.2007.12.005 (2008).
265 Kim, Y. *et al.* Molecular mechanisms of MHC class I-antigen processing: redox considerations. *Antioxid Redox Signal* **11**, 907-936, doi:10.1089/ars.2008.2316 (2009).
266 Bottino, C. *et al.* NTB-A [correction of GNTB-A], a novel SH2D1A-associated surface molecule contributing to the inability of natural killer cells to kill Epstein-Barr virus-infected B cells in X-linked lymphoproliferative disease. *J Exp Med* **194**, 235-246, doi:10.1084/jem.194.3.235 (2001).
267 Cao, E. *et al.* NTB-A receptor crystal structure: insights into homophilic interactions in the signaling lymphocytic activation molecule receptor family. *Immunity* **25**, 559-570, doi:10.1016/j.immuni.2006.06.020 (2006).
268 Barrow, A. D. *et al.* OSCAR is a collagen receptor that costimulates osteoclastogenesis in DAP12-deficient humans and mice. *J Clin Invest* **121**, 3505-

3516, doi:10.1172/jci45913 (2011).
269 Merck, E. *et al.* OSCAR is an FcRgamma-associated receptor that is expressed by myeloid cells and is involved in antigen presentation and activation of human dendritic cells. *Blood* **104**, 1386-1395, doi:10.1182/blood-2004-03-0850 (2004).
270 Rock, K. L., York, I. A. & Goldberg, A. L. Post-proteasomal antigen processing for major histocompatibility complex class I presentation. *Nat Immunol* **5**, 670-677, doi:10.1038/ni1089 (2004).
271 Kloetzel, P. M. & Ossendorp, F. Proteasome and peptidase function in MHC-class-I-mediated antigen presentation. *Curr Opin Immunol* **16**, 76-81, doi:10.1016/j.coi.2003.11.004 (2004).
272 Groettrup, M., Kirk, C. J. & Basler, M. Proteasomes in immune cells: more than peptide producers? *Nat Rev Immunol* **10**, 73-78, doi:10.1038/nri2687 (2010).
273 Wearsch, P. A. & Cresswell, P. The quality control of MHC class I peptide loading. *Curr Opin Cell Biol* **20**, 624-631, doi:10.1016/j.ceb.2008.09.005 (2008).
274 Princiotta, M. F. *et al.* Quantitating protein synthesis, degradation, and endogenous antigen processing. *Immunity* **18**, 343-354, doi:10.1016/s1074-7613(03)00051-7 (2003).
275 Giodini, A., Rahner, C. & Cresswell, P. Receptor-mediated phagocytosis elicits cross-presentation in nonprofessional antigen-presenting cells. *Proc Natl Acad Sci U S A* **106**, 3324-3329, doi:10.1073/pnas.0813305106 (2009).
276 Reith, W., LeibundGut-Landmann, S. & Waldburger, J. M. Regulation of MHC class II gene expression by the class II transactivator. *Nat Rev Immunol* **5**, 793-806, doi:10.1038/nri1708 (2005).
277 Deshaies, R. J. & Joazeiro, C. A. RING domain E3 ubiquitin ligases. *Annu Rev Biochem* **78**, 399-434, doi:10.1146/annurev.biochem.78.101807.093809 (2009).
278 Deng, L. & Mariuzza, R. A. Structural basis for recognition of MHC and MHC-like ligands by natural killer cell receptors. *Semin Immunol* **18**, 159-166, doi:10.1016/j.smim.2006.03.004 (2006).
279 Sawicki, M. W. *et al.* Structural basis of MHC class I recognition by natural killer cell receptors. *Immunol Rev* **181**, 52-65, doi:10.1034/j.1600-065x.2001.1810104.x (2001).
280 Lizee, G., Basha, G. & Jefferies, W. A. Tails of wonder: endocytic-sorting motifs key for exogenous antigen presentation. *Trends Immunol* **26**, 141-149, doi:10.1016/j.it.2005.01.005 (2005).
281 Sharpe, A. H. & Freeman, G. J. The B7-CD28 superfamily. *Nat Rev Immunol* **2**, 116-126, doi:10.1038/nri727 (2002).
282 Carrasco, Y. R. & Batista, F. D. B cell recognition of membrane-bound antigen: an exquisite way of sensing ligands. *Curr Opin Immunol* **18**, 286-291, doi:10.1016/j.coi.2006.03.013 (2006).
283 Montoya, M. C., Sancho, D., Vicente-Manzanares, M. & Sanchez-Madrid, F. Cell adhesion and polarity during immune interactions. *Immunol Rev* **186**, 68-82, doi:10.1034/j.1600-065x.2002.18607.x (2002).
284 Arnett, K. L., Harrison, S. C. & Wiley, D. C. Crystal structure of a human CD3-epsilon/delta dimer in complex with a UCHT1 single-chain antibody fragment. *Proc Natl Acad Sci U S A* **101**, 16268-16273, doi:10.1073/pnas.0407359101 (2004).

285 Kjer-Nielsen, L. *et al.* Crystal structure of the human T cell receptor CD3 epsilon gamma heterodimer complexed to the therapeutic mAb OKT3. *Proc Natl Acad Sci U S A* **101**, 7675-7680, doi:10.1073/pnas.0402295101 (2004).
286 Dejana, E. Endothelial cell-cell junctions: happy together. *Nat Rev Mol Cell Biol* **5**, 261-270, doi:10.1038/nrm1357 (2004).
287 Vivier, E., Tomasello, E., Baratin, M., Walzer, T. & Ugolini, S. Functions of natural killer cells. *Nat Immunol* **9**, 503-510, doi:10.1038/ni1582 (2008).
288 Rudolph, M. G., Stanfield, R. L. & Wilson, I. A. How TCRs bind MHCs, peptides, and coreceptors. *Annu Rev Immunol* **24**, 419-466, doi:10.1146/annurev.immunol.23.021704.115658 (2006).
289 Cemerski, S. & Shaw, A. Immune synapses in T-cell activation. *Curr Opin Immunol* **18**, 298-304, doi:10.1016/j.coi.2006.03.011 (2006).
290 Bromley, S. K. *et al.* The immunological synapse. *Annu Rev Immunol* **19**, 375-396, doi:10.1146/annurev.immunol.19.1.375 (2001).
291 Yusuf-Makagiansar, H., Anderson, M. E., Yakovleva, T. V., Murray, J. S. & Siahaan, T. J. Inhibition of LFA-1/ICAM-1 and VLA-4/VCAM-1 as a therapeutic approach to inflammation and autoimmune diseases. *Med Res Rev* **22**, 146-167 (2002).
292 Bazzoni, G. The JAM family of junctional adhesion molecules. *Curr Opin Cell Biol* **15**, 525-530, doi:10.1016/s0955-0674(03)00104-2 (2003).
293 Muller, W. A. Leukocyte-endothelial-cell interactions in leukocyte transmigration and the inflammatory response. *Trends Immunol* **24**, 327-334 (2003).
294 Benson, D. L., Schnapp, L. M., Shapiro, L. & Huntley, G. W. Making memories stick: cell-adhesion molecules in synaptic plasticity. *Trends Cell Biol* **10**, 473-482, doi:10.1016/s0962-8924(00)01838-9 (2000).
295 Barclay, A. N. Membrane proteins with immunoglobulin-like domains--a master superfamily of interaction molecules. *Semin Immunol* **15**, 215-223 (2003).
296 Ethell, I. M. & Pasquale, E. B. Molecular mechanisms of dendritic spine development and remodeling. *Prog Neurobiol* **75**, 161-205, doi:10.1016/j.pneurobio.2005.02.003 (2005).
297 Salzer, J. L. Polarized domains of myelinated axons. *Neuron* **40**, 297-318, doi:10.1016/s0896-6273(03)00628-7 (2003).
298 Nedvetzki, S. *et al.* Reciprocal regulation of human natural killer cells and macrophages associated with distinct immune synapses. *Blood* **109**, 3776-3785, doi:10.1182/blood-2006-10-052977 (2007).
299 Fuchs, A. & Colonna, M. The role of NK cell recognition of nectin and nectin-like proteins in tumor immunosurveillance. *Semin Cancer Biol* **16**, 359-366, doi:10.1016/j.semcancer.2006.07.002 (2006).
300 Yamagata, M., Sanes, J. R. & Weiner, J. A. Synaptic adhesion molecules. *Curr Opin Cell Biol* **15**, 621-632, doi:10.1016/s0955-0674(03)00107-8 (2003).
301 Labbe, E., Letamendia, A. & Attisano, L. Association of Smads with lymphoid enhancer binding factor 1/T cell-specific factor mediates cooperative signaling by the transforming growth factor-beta and wnt pathways. *Proc Natl Acad Sci U S A* **97**, 8358-8363, doi:10.1073/pnas.150152697 (2000).
302 Conacci-Sorrell, M. *et al.* Autoregulation of E-cadherin expression by cadherin-cadherin interactions: the roles of beta-catenin signaling, Slug, and MAPK. *J Cell Biol*

163, 847-857, doi:10.1083/jcb.200308162 (2003).
303 Angst, B. D., Marcozzi, C. & Magee, A. I. The cadherin superfamily: diversity in form and function. *J Cell Sci* **114**, 629-641 (2001).
304 Nelson, W. J. & Nusse, R. Convergence of Wnt, beta-catenin, and cadherin pathways. *Science* **303**, 1483-1487, doi:10.1126/science.1094291 (2004).
305 Beavon, I. R. The E-cadherin-catenin complex in tumour metastasis: structure, function and regulation. *Eur J Cancer* **36**, 1607-1620, doi:10.1016/s0959-8049(00)00158-1 (2000).
306 Reynolds, A. B. & Roczniak-Ferguson, A. Emerging roles for p120-catenin in cell adhesion and cancer. *Oncogene* **23**, 7947-7956, doi:10.1038/sj.onc.1208161 (2004).
307 Ciruna, B. & Rossant, J. FGF signaling regulates mesoderm cell fate specification and morphogenetic movement at the primitive streak. *Dev Cell* **1**, 37-49 (2001).
308 Irie, K., Shimizu, K., Sakisaka, T., Ikeda, W. & Takai, Y. Roles and modes of action of nectins in cell-cell adhesion. *Semin Cell Dev Biol* **15**, 643-656, doi:10.1016/j.semcdb.2004.09.002 (2004).
309 Peinado, H., Quintanilla, M. & Cano, A. Transforming growth factor beta-1 induces snail transcription factor in epithelial cell lines: mechanisms for epithelial mesenchymal transitions. *J Biol Chem* **278**, 21113-21123, doi:10.1074/jbc.M211304200 (2003).
310 Imhof, B. A. & Aurrand-Lions, M. Adhesion mechanisms regulating the migration of monocytes. *Nat Rev Immunol* **4**, 432-444, doi:10.1038/nri1375 (2004).
311 Etienne-Manneville, S. *et al.* ICAM-1-coupled cytoskeletal rearrangements and transendothelial lymphocyte migration involve intracellular calcium signaling in brain endothelial cell lines. *J Immunol* **165**, 3375-3383, doi:10.4049/jimmunol.165.6.3375 (2000).
312 van Buul, J. D. & Hordijk, P. L. Signaling in leukocyte transendothelial migration. *Arterioscler Thromb Vasc Biol* **24**, 824-833, doi:10.1161/01.ATV.0000122854.76267.5c (2004).
313 Deem, T. L. & Cook-Mills, J. M. Vascular cell adhesion molecule 1 (VCAM-1) activation of endothelial cell matrix metalloproteinases: role of reactive oxygen species. *Blood* **104**, 2385-2393, doi:10.1182/blood-2004-02-0665 (2004).
314 Crittenden, J. R. *et al.* CalDAG-GEFI integrates signaling for platelet aggregation and thrombus formation. *Nat Med* **10**, 982-986, doi:10.1038/nm1098 (2004).
315 Pannekoek, W. J., Kooistra, M. R., Zwartkruis, F. J. & Bos, J. L. Cell-cell junction formation: the role of Rap1 and Rap1 guanine nucleotide exchange factors. *Biochim Biophys Acta* **1788**, 790-796, doi:10.1016/j.bbamem.2008.12.010 (2009).
316 Caron, E. Cellular functions of the Rap1 GTP-binding protein: a pattern emerges. *J Cell Sci* **116**, 435-440, doi:10.1242/jcs.00238 (2003).
317 Boettner, B. & Van Aelst, L. Control of cell adhesion dynamics by Rap1 signaling. *Curr Opin Cell Biol* **21**, 684-693, doi:10.1016/j.ceb.2009.06.004 (2009).
318 Hoffmeister, M. *et al.* Cyclic nucleotide-dependent protein kinases inhibit binding of 14-3-3 to the GTPase-activating protein Rap1GAP2 in platelets. *J Biol Chem* **283**, 2297-2306, doi:10.1074/jbc.M706825200 (2008).
319 Stork, P. J. Does Rap1 deserve a bad Rap? *Trends Biochem Sci* **28**, 267-275, doi:10.1016/s0968-0004(03)00087-2 (2003).

320 Bos, J. L. Linking Rap to cell adhesion. *Curr Opin Cell Biol* **17**, 123-128, doi:10.1016/j.ceb.2005.02.009 (2005).

321 Stork, P. J. & Dillon, T. J. Multiple roles of Rap1 in hematopoietic cells: complementary versus antagonistic functions. *Blood* **106**, 2952-2961, doi:10.1182/blood-2005-03-1062 (2005).

322 Doebele, R. C. *et al.* A novel interplay between Epac/Rap1 and mitogen-activated protein kinase kinase 5/extracellular signal-regulated kinase 5 (MEK5/ERK5) regulates thrombospondin to control angiogenesis. *Blood* **114**, 4592-4600, doi:10.1182/blood-2009-04-217042 (2009).

323 Polakis, P. G., Rubinfeld, B., Evans, T. & McCormick, F. Purification of a plasma membrane-associated GTPase-activating protein specific for rap1/Krev-1 from HL60 cells. *Proc Natl Acad Sci U S A* **88**, 239-243, doi:10.1073/pnas.88.1.239 (1991).

324 Bos, J. L., de Rooij, J. & Reedquist, K. A. Rap1 signalling: adhering to new models. *Nat Rev Mol Cell Biol* **2**, 369-377, doi:10.1038/35073073 (2001).

325 Kooistra, M. R., Dube, N. & Bos, J. L. Rap1: a key regulator in cell-cell junction formation. *J Cell Sci* **120**, 17-22, doi:10.1242/jcs.03306 (2007).

326 Schultess, J., Danielewski, O. & Smolenski, A. P. Rap1GAP2 is a new GTPase-activating protein of Rap1 expressed in human platelets. *Blood* **105**, 3185-3192, doi:10.1182/blood-2004-09-3605 (2005).

327 Minato, N., Kometani, K. & Hattori, M. Regulation of immune responses and hematopoiesis by the Rap1 signal. *Adv Immunol* **93**, 229-264, doi:10.1016/s0065-2776(06)93006-5 (2007).

328 Lafuente, E. M. *et al.* RIAM, an Ena/VASP and Profilin ligand, interacts with Rap1-GTP and mediates Rap1-induced adhesion. *Dev Cell* **7**, 585-595, doi:10.1016/j.devcel.2004.07.021 (2004).

329 Xia, Z. & Storm, D. R. The role of calmodulin as a signal integrator for synaptic plasticity. *Nat Rev Neurosci* **6**, 267-276, doi:10.1038/nrn1647 (2005).

330 Mor, A., Dustin, M. L. & Philips, M. R. Small GTPases and LFA-1 reciprocally modulate adhesion and signaling. *Immunol Rev* **218**, 114-125, doi:10.1111/j.1600-065X.2007.00538.x (2007).

331 Burbach, B. J., Medeiros, R. B., Mueller, K. L. & Shimizu, Y. T-cell receptor signaling to integrins. *Immunol Rev* **218**, 65-81, doi:10.1111/j.1600-065X.2007.00527.x (2007).
