# Supplementary material for: Differential early diagnosis of benign versus malignant lung cancer using systematic pathway flux analysis of peripheral blood leukocytes
Source: Sci Rep. 2022 Mar 24;12:5070. doi: 10.1038/s41598-022-08890-x (PMC8948197; doi:10.1038/s41598-022-08890-x)
Supplement: Supplementary file 3 — Supplementary Information 3. [file 41598_2022_8890_MOESM3_ESM.pdf]

Supplement Information / Figures / Tables

Differential Early Diagnosis of Benign vs Malignant Lung Cancer using Systematic Pathway Flux Analysis of Peripheral Blood Leukocytes

Jian Li[1,+], Xiaoyu Li[2,+], Ming Li[3,+], Hong Qiu[2], Christian Saad[4], Bo Zhao[5], Fan Li[5], Xiaowei Wu[5], Dong Kuang[6,7], Fengjuan Tang[6,7], Yaobing Chen[6,7], Hongge Shu[8], Jing Zhang[8], Qiuxia Wang[8], He Huang[9], Shankang Qi[9], Changkun Ye[10], Amy Bryant[11], Xianglin Yuan[2], Christian Kurts[1], Guangyuan Hu[2,*] , Weiting Cheng[12,*], Qi Mei[2,*]

[1] Institute of Molecular Medicine and Experimental Immunology, University Clinic of Rheinische Friedrich-Wilhelms-University, Bonn, Germany
[2] Department of Oncology, Tongji Hospital, Tongji Medical College, Huazhong University of Science and Technology, Wuhan, Hubei, People's Republic of China
[3] Department of Oncology, Wuhan Pulmonary Hospital, Wuhan, Hubei, People's Republic of China
[4] Department of Computer Science, University of Augsburg, Augsburg, Germany
[5] Department of thoracic surgery, Tongji Hospital, Tongji Medical College, Huazhong University of Science and Technology, Wuhan, Hubei, People's Republic of China
[6] Institute of Pathology, Tongji Hospital, Tongji Medical College, Huazhong University of Science and Technology, Wuhan, Hubei, People's Republic of China
[7] Department of Pathology, School of Basic Medicine, Tongji Medical College, Huazhong University of Science and Technology, Wuhan, Hubei, People's Republic of China
[8] Radiology department, Tongji Hospital, Tongji Medical College, Huazhong University of Science and Technology, Wuhan, Hubei, People's Republic of China
[9] Shanghai Institute of Materia Medica, Chinese Academy of Sciences, Shanghai, People's Republic of China
[10] Medical Research Center of Yu Huang Hospital, Yu Huang, Zhejiang, People's Republic of China
[11] Department of Biochemical and Pharmaceutical Sciences, College of Pharmacy, Idaho State University
[12] Department of Oncology, Wuhan No. 1 Hospital, Wuhan, Hubei, People's Republic of China
[*] Corresponding Authors: h.g.y.121@163.com, joycvt@126.com, borismq@163.com
[+] Contributing equally authors

**Supplement Table 1.** Clinical characteristics and laboratory findings in the validation cohort				
	**Overall**	**Benign**	**Malignant**	***P*-value**
Number of patients	40	10	30	…
Age, years	55.6 [47.3-62.7]	52.0 [41.0-61.2]	57.0 [52.2-63.2]	0.035
Sex	…	…	…	0.836
Male	27 (67.5)	5 (55.6)	18 (60.0)	…
Female	13 (32.5)	4 (44.4)	12 (40.0)	…
Clinical stages	...	...	...	...
I	23 (57.5)	...	23 (76.7)	...
II	4 (10.0)	...	4 (13.3)	...
III	3 (7.5)	...	3 (10.0)	...
IV	0 (0.0)	...	0 (0.0)	...
T stage	...	...	...	...
1	14 (35.0)	...	14 (46.7)	...
2	13 (32.5)	...	13 (43.3)	...
3	2 (5.0)	...	2 (6.7)	...
4	1 (2.5)	...	1 (3.3)	...
N stage	...	...	...	...
0	25 (52.5)	...	25 (83.3)	...
1	2 (5.0)	...	2 (6.7)	...
2	2 (5.0)	...	2 (6.7)	...
3	1 (2.5)	...	1 (3.3)	...
M stage	...	...	...	...
0	28 (70.0)	...	28 (93.3)	...
1	2 (5.0)	...	2 (6.7)	...
Pathological type				...
Adenocarcinoma	20 (50.0)	...	20 (66.7)	...
Squamous carcinoma	7 (17.5)	...	7 (23.3)	...
Other carcinoma	3 (7.5)	...	3 (10.0)	...
Invasive depth	...	...	...	...
0	2 (5.0)	...	2 (6.7)	...
1	2 (5.0)	...	2 (6.7)	...
2	26 (65.0)	...	26 (86.6)	...
Differentiation grade	...	...	...	...
1	4 (10.0)	...	4 (13.3)	...
2	24 (60.0)	...	24 (80.0)	...
3	2 (5.0)	...	2 (6.7)	...
No. of pulmonary nodules	...	...	...	0.306
1	35 (62.5)	10 (100.0)	25 (83.3)	...
2	4 (10.0)	0 (0.0)	4 (13.3)	...
3	1 (2.5)	0 (0.0)	1 (3.3)	...
TIL grade	...	...	...	...
0	22 (55.0)	...	22 (73.3)	...
1	7 (17.5)	...	7 (23.3)	...
2	1 (2.5)	...	1 (3.3)	...
TII	10.0 [6.0-15.0]	...	10.0 [6.0-15.0]	...
CD8	1.0 [1.0-1.0]	1.0 [1.0-1.0]	1.0 [1.0-2.5]	0.236
PD-L1	3.0 [2.5-4.25]	1.0 [1.0-1.0]	3.0 [3.0-8.0]	<.001
CPS	4.0 [1.75-11.0]	3.0 [3.0-3.0]	7.0 [3.3-11.0]	<.001
TIMIT	...	...	...	...
I	11 (27.5)	...	11 (36.7)	...
II	8 (20.0)	...	8 (26.7)	...
III	3 (7.5)	...	3 (10.0)	...

IV	8 (20.0)	...	8 (26.7)	...
Smoking	17 (42.5)	5 (50.0)	12 (40.0)	0.717
Macrophage	...	...	...	...
≤5	24 (60.0)	...	24 (80.0)	...
5-10	4 (10.0)	...	4 (13.3)	...
≥10	2 (5.0)	...	2 (6.7)	...
Ki67	0.3 [0.1-0.6]	…	0.3 [0.1-0.6]	...
Tumor diameter, cm	...	...	...	...
Imaging	2.5 [1.2-3.2]	2.5 [1.6-3.5]	2.4 [1.1-3.0]	0.751
Surgical	2.5 [1.5-3.8]	2.3 [1.6-3.8]	2.5 [1.5-3.5]	0.710
Pathological	2.2 [1.5-3.8]	2.5 [1.0-3.0]	2.2 [1.5-4.0]	0.431
CEA, ng/mL	34.0 [10.8-55.3]	16.5 [8.5-23.8]	44.5 [14.5-61.8]	<.001
NSE, ug/L	42.0 [15.3-70.3]	37.5 [9.3-44.8]	46.0 [22.5-71.8]	0.323
CYFRA19, ug/L	32.0 [11.8-53.0]	25.0 [13.5-44.0]	33.5 [10.0-55.0]	0.649
SCC , ng/mL	8.0 [6.0-10.3]	8.0 [6.3-10.0]	8.5 [6.3-10.0]	0.384
ProGRP, pg/mL	32.5 [10.3-58.5]	36.5 [21.0-51.8]	30.0 [6.5-60.8]	0.856
WBC count, × 10^9/L	5.9 [4.6-7.6]	6.0 [4.7-6.7]	5.9 [4.7-8.1]	0.460
Neutrophil count, × 10^9/L	3.9 [2.7-5.1]	3.5 [2.7-4.1]	3.9 [2.8-5.6]	0.209
Lymphocyte count, × 10^9/L	1.5 [1.3-1.8]	1.7 [1.5-1.9]	1.5 [1.3-1.8]	0.139
Hemoglobin, g/L	138.0 [124.0-151.0]	151.0 [146.2-156.8]	132.0 [121.0-141.0]	0.022
PLT count, × 10^9/L	213.0 [172.0-254.5]	217.0 [207.5-260.2]	206.0 [170.0-252.0]	0.710

Supplement Table 2. The statistics overview of the model-components. Complex includes protein-protein complex, protein-metabolite complex, protein-mRNA and protein-gene complex. Compound is metabolite. PseudoObj includes protein-inhibitors (EGFR inhibitor, Abl inhibitor and others) and hallmarks (Tumorigenesis, Proliferation, Apoptosis, etc).

(a.)

Component	Amount
Gene	3643
mRNA	5198
miRNA	1048
Protein	3082
Complex	1165
Compound	1011
PseudoObj	106
In total	15253
Pathway	115

(b.)

Reactions	Amount
Transcription	3813
Translation	2322
Degradation	7143
Complex-formation	580
Translocation	4142
Phosphorylation	1384
dephosphorylation	451
Activation	275
Ubiquitination	58
Metabolism	1168
Complex-Interaction	243
Other	53
In total	21632
